# Discovery of a Series of Macrocycles as Potent Inhibitors of *Leishmania Infantum*

**DOI:** 10.1021/acs.jmedchem.4c01370

**Published:** 2024-10-08

**Authors:** Federico Riu, Larissa Alena Ruppitsch, Duc Duy Vo, Richard S. Hong, Mohit Tyagi, An Matheeussen, Sarah Hendrickx, Vasanthanathan Poongavanam, Guy Caljon, Ahmad Y. Sheikh, Peter Sjö, Jan Kihlberg

**Affiliations:** † Department of Chemistry − BMC, Uppsala University, 751 23 Uppsala, Sweden; ‡ Science for Life Laboratory, Department of Cell and Molecular Biology, Uppsala University, 751 24 Uppsala, Sweden; § Molecular Profiling and Drug Delivery, Research & Development, AbbVie Inc., Worcester, Massachusetts 01605, United States; ∥ Laboratory of Microbiology, Parasitology and Hygiene, University of Antwerp, Universiteitsplein 1, 2610 Wilrijk, Belgium; ⊥ Molecular Profiling and Drug Delivery, Research & Development, AbbVie Inc, North Chicago, Illinois 60064, United States; # Drugs for Neglected Diseases initiative (DNDi), 15 Chemin Camille-Vidart, 1202 Geneva, Switzerland

## Abstract

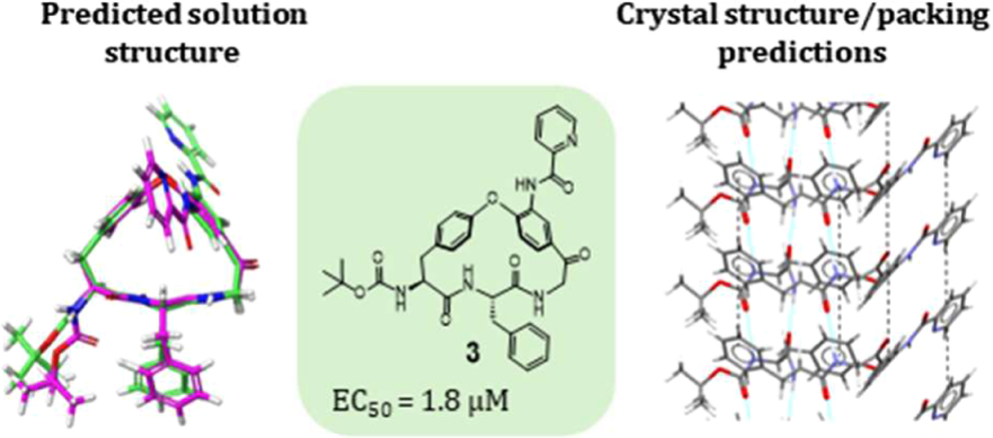

Macrocycles are prominent among drugs for treatment of infectious disease, with many originating from natural products. Herein we report on the discovery of a series of macrocycles structurally related to the natural product hymenocardine. Members of this series were found to inhibit the growth of *Plasmodium falciparum*, the parasite responsible for most malaria cases, and of four kinetoplastid parasites. Notably, macrocycles more potent than miltefosine, the only oral drug used for the treatment of the neglected tropical disease visceral leishmaniasis, were identified in a phenotypic screen of *Leishmania infantum*. *In vitro* profiling highlighted that potent inhibitors had satisfactory cell permeability with a low efflux ratio, indicating their potential for oral administration, but low solubility and metabolic stability. Analysis of predicted crystal structures suggests that optimization should focus on the reduction of π–π crystal packing interactions to reduce the strong crystalline interactions and improve the solubility of the most potent lead.

## Introduction

Close to 70 macrocycles had been approved as drugs by the FDA by September 2022.^1^ At the time, another 34 were in clinical trials, but the number is most likely higher as structures are not always disclosed for clinical candidates. Approximately 45% of the approved macrocyclic drugs are used for the treatment of infectious diseases with oncology being the second most frequent indication (21%). Interestingly, the order of these two indications is reversed for the clinical candidates, suggesting a broadening use of macrocycles. The versatility of macrocycles is also reflected when viewed from a target perspective.^1^ Anti-infective macrocyclic drugs and clinical candidates mainly act on a few targets, e.g., the ribosome, RNA polymerase and the NS3/4A protease of HCV. In oncology, macrocycles modulate a larger and more diverse set of targets, which include kinases, deacetylases, hormone receptors, tubulin and DNA. Three rapamycin natural product derivatives used in oncology function as molecular glues. Moreover, macrocycles are directed against a variety of targets in many other indications. It is important to note that natural products and their derivatives dominate *de novo*-designed compounds (ratio >4:1) across the FDA approved macrocyclic drugs and the macrocyclic clinical candidates.^1^ Finally, the number of publications describing the use of macrocycles in drug discovery has increased rapidly and reveals that macrocycles are being investigated for modulation of a large number of novel targets and positioned for use in a multitude of indications.^1^

The conformational preorganization obtained by macrocyclization provides unique opportunities for modulating protein targets that have difficult-to-drug flat, tunnel- or groove-shaped binding sites.^1,2^ Inspection of cocrystal structures of macrocyclic drugs bound to their targets reveals that macrocycles can modulate such targets because they adopt disc- and sphere-like conformations to a larger extent than nonmacrocyclic drugs that comply with Lipinski′s rule of 5 (Ro5).^1^ As a consequence, macrocyclic drugs are usually larger and structurally more complex than Ro5-compliant drugs, i.e., they reside in the beyond-Rule-of-5 (bRo5) chemical space.^3,4^ Despite this, macrocycles frequently possess oral bioavailability; close to 40% of all approved macrocyclic drugs are orally bioavailable. Molecular chameleonicity, i.e., the ability of compounds to undergo conformational changes that adapt their polarity to the surrounding environment, has been proposed as one property that may improve bioavailability in the bRo5 space.^5^ By judicious optimization, it may even be possible to discover orally bioavailable macrocyclic drugs that distribute into the central nervous system, with lorlatinib approved for treatment of cancer metastases in the brain being one example.^6^ However, clinical development of macrocycles, just as for other compounds that reside in the bRo5 space, is often more demanding than for traditional Ro5-compliant drugs. Reasons for this may include the scaling up of complex multistep synthetic routes and multiple solid-state polymorphs, where different crystal forms may have large differences in physical and chemical stability, solubility and oral bioavailability.^7,8^

Using inspiration from natural products is an attractive approach to discovering macrocyclic drugs for targets that are difficult to modulate with Ro5-compliant, nonmacrocyclic ligands. Unsurprisingly, several groups have already reported such approaches. For instance, libraries of macrocycles that contain motifs from stereochemically complex natural products or polyketide macrolides have been designed and then prepared by diversity-oriented synthesis.^9,10^ Additionally, natural product-derived fragments have been combined to provide pseudonatural products,^11^ or combined with short peptide epitopes to give macrocycles termed PepNats.^12^ We recently reported a novel approach that utilizes the structural diversity of macrocyclic natural products in drug discovery.^13^ Systematic *in silico* mining of natural products provided a set of macrocyclic cores for use in the discovery of new macrocyclic lead compounds, which was successfully applied to the discovery of nM macrocyclic inhibitors of the Keap1-Nrf2 protein–protein interaction.^13,14^ Approaches such as the above ones, which incorporate inspiration from natural products in the design of macrocycles, may mitigate the major drawback of natural products, i.e., that their complex structures require multistep synthetic routes, making structural modifications in lead optimization very resource-intensive.

**Figure 1:**
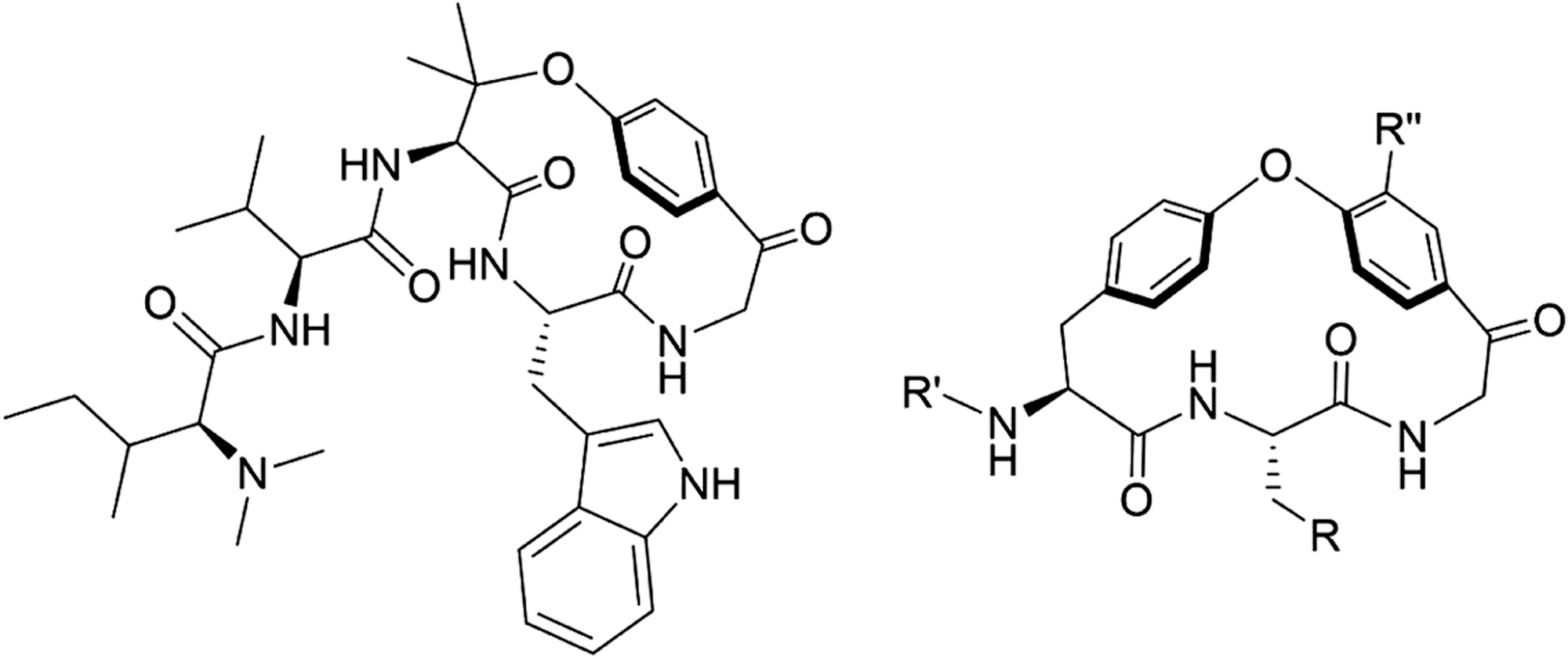
Comparison of the structures of hymenocardine (left) and the macrocyclic core of the series reported herein.

Herein, we report a series of macrocyclic compounds, inspired by the natural product hymenocardine (Figure 1),^13,15^ the core of which was found in the set obtained in our mining of macrocyclic natural products. Hymenocardine has been reported to inhibit the growth of *Plasmodium falciparum*, the parasite which is responsible for most deaths in malaria.^16^ Therefore, the series of macrocycles was evaluated as inhibitors of *P. falciparum* and four kinetoplastid parasites: *Trypanosoma cruzi*, *Leishmania infantum*, *Trypanosoma brucei brucei*, and *Trypanosoma brucei rhodesiense*. *T. cruzi* and *L. infantum* cause the neglected tropical diseases Chagas disease and visceral leishmaniasis, respectively, while *T. b. rhodesiense* causes sleeping sickness. *T. b. brucei* is known to infect cattle and rodents, but not humans.

Parasitic infections place a significant burden on individuals and society, especially in low-income communities in tropical regions. They impact not only health status of individuals but also the social and economic status of families and local communities. Malaria is a leading cause of death globally; in 2021, the number of estimated malaria cases reached 247 million, resulting in 619,000 deaths.^17^ For Chagas disease, about 6–7 million people worldwide are infected with *T. cruzi*, leading to approximately 12,000 deaths every year.^18^ For leishmaniasis the numbers are lower, with 700,000 to 1 million new cases each year.^19^ Thanks to successful surveillance programs and the introduction of efficient drug treatments, the number of new cases of sleeping sickness has been reduced dramatically from more than 40,000 recorded in 1998 to below 1000 in 2017.^20^

Screening of the hymenocardine-inspired series of compounds resulted in the identification of cell-permeable inhibitors of *L. infantum* more potent than miltefosine, the only oral drug approved for treatment of visceral leishmaniasis. Evaluation of a ring-opened analog of the most potent inhibitor, and a conformational analysis based on quantum mechanics, demonstrated the importance of the macrocyclic scaffold for the potency of the series. Determination of the lipophilicity, aqueous solubility, and metabolic stability suggested that lipophilicity should be reduced for the series to increase solubility and metabolic stability. Analysis of the predicted crystal structure and crystal packing of the most potent macrocycle indicated that strong solid-state interactions significantly contribute to the observed low solubility of many compounds in the series, and suggested approaches to reduce this liability.

## Results

### Identification of a Potent Macrocyclic Hit

**Figure 2:**
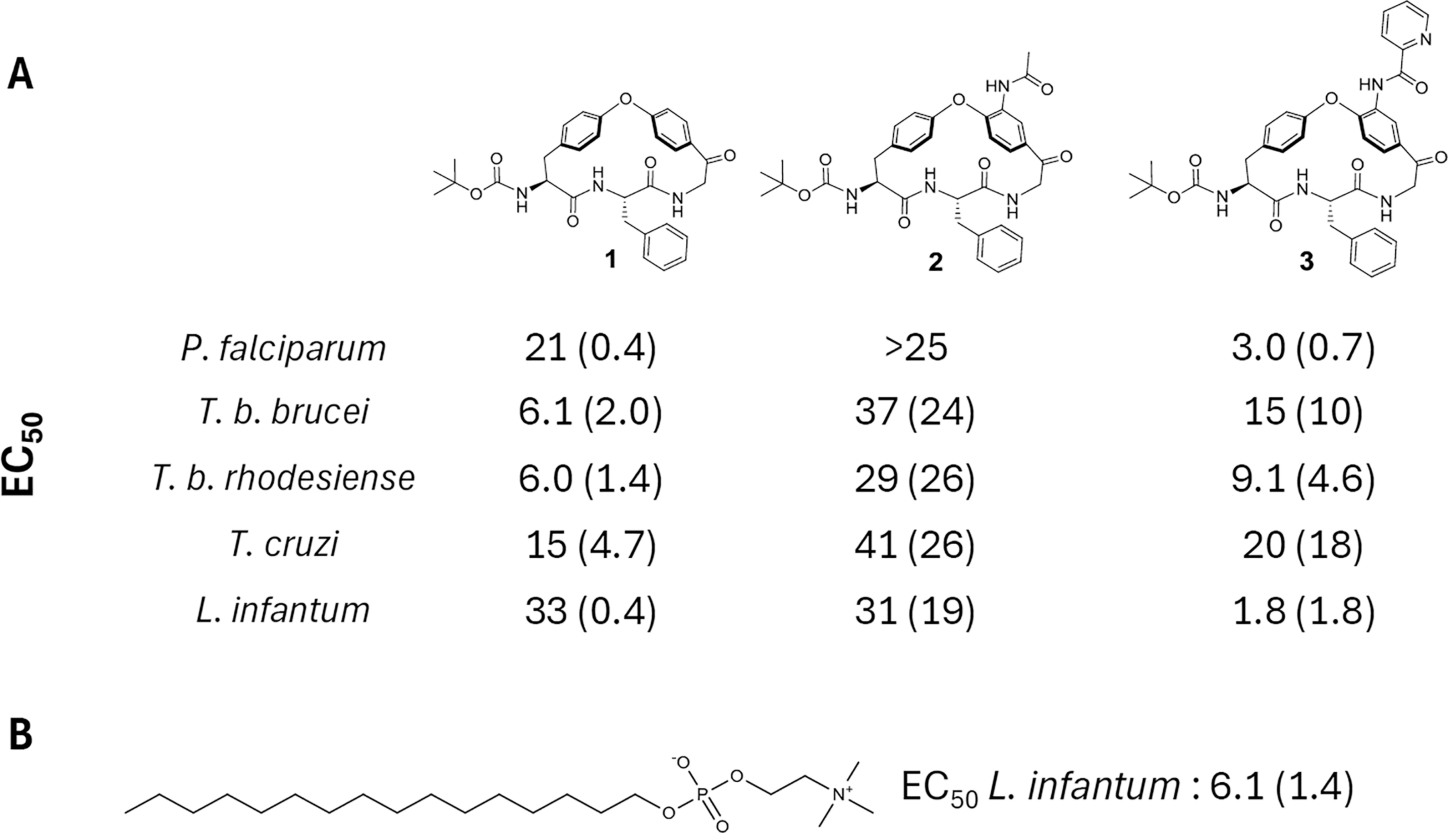
(A) Structures of macrocycles **1**–**3** and their potency as inhibitors of the growth of five parasites. Inhibitory potencies are mean values originating from 2 to 14 measurements, with the standard deviation given in parentheses. (B) Structure of miltefosine and its potency as an inhibitor of *L. infantum*, originating from 19 measurements.

We previously reported the synthesis of a series of macrocycles, including **1** (Figure 2A), designed to investigate how structural features influence molecular chameleonicity.^15,21^ Originating from the structural similarities between **1** and hymenocardine, we investigated **1** in screens for *P. falciparum* and four kinetoplastid parasites: *T. b. brucei*, *T. b. rhodesiense*, *T. cruzi*, and *L. infantum*. In the screens, we also included compounds **2** and **3** (Figure 2A), which were obtained from an intermediate late in the synthetic route to **1**. Since very few validated protein targets relevant to clearance of parasites are known, phenotypic assays were used to investigate the antiparasitic activity for all five parasites. For malaria, the *P. falciparum* blood stage 3D7 strain was used to test the three compounds. For the two *Trypanosoma brucei* species, the reduction in trypomastigote growth in culture medium was determined after the addition of **1**–**3**. For *T. cruzi*, the intracellular parasite growth in infected human fibroblasts (MRC-5 cell line) was assessed in the presence and absence of the three inhibitors. Similarly, the growth of *L. infantum* was assessed in infected primary mouse macrophages (PMMs) in the presence and absence of the inhibitors. *In vitro* host cell cytotoxicity was determined in the MRC-5 and PMM cell lines in the presence of the inhibitor; none of the three compounds displayed any host cell cytotoxicity (CC_50_ > 64 μM). The assays for *T. cruzi* and *L. infantum* are complex, i.e., they determine the degree of inhibition of parasitic replication in a mammalian cell line; this complexity may lead to a high variability for some compounds.

Macrocycles **1** and **2** had low or no potency as inhibitors of *P. falciparum*, while **3** was found to be moderately potent (Figure 2A). All three macrocycles were found to have low activity against *T. b. brucei* and *T. b. rhodesiense* (EC_50_ > 5 μM), while **1** and **3** were weak inhibitors of *T. cruzi* (10 < EC_50_ < 30 μM) (Figure 2). Most interestingly, macrocycle **3**, but not **1** and **2**, was shown to be a potent inhibitor of the growth of *L. infantum* (EC_50_ < 2 μM). In fact, **3** was more potent than miltefosine (Figure 2B), the only orally administered drug approved for the treatment of visceral leishmaniasis. Compound **3** was therefore chosen as the starting point for structure–activity relationship (SAR) studies to evaluate its potential for further optimization into a novel, oral treatment for leishmaniasis. All compounds were also evaluated in parallel as inhibitors of *T. b. brucei*, *T. b. rhodesiense*, and *T. cruzi*.

### Design of Macrocycles for SAR Exploration

**Figure 3:**
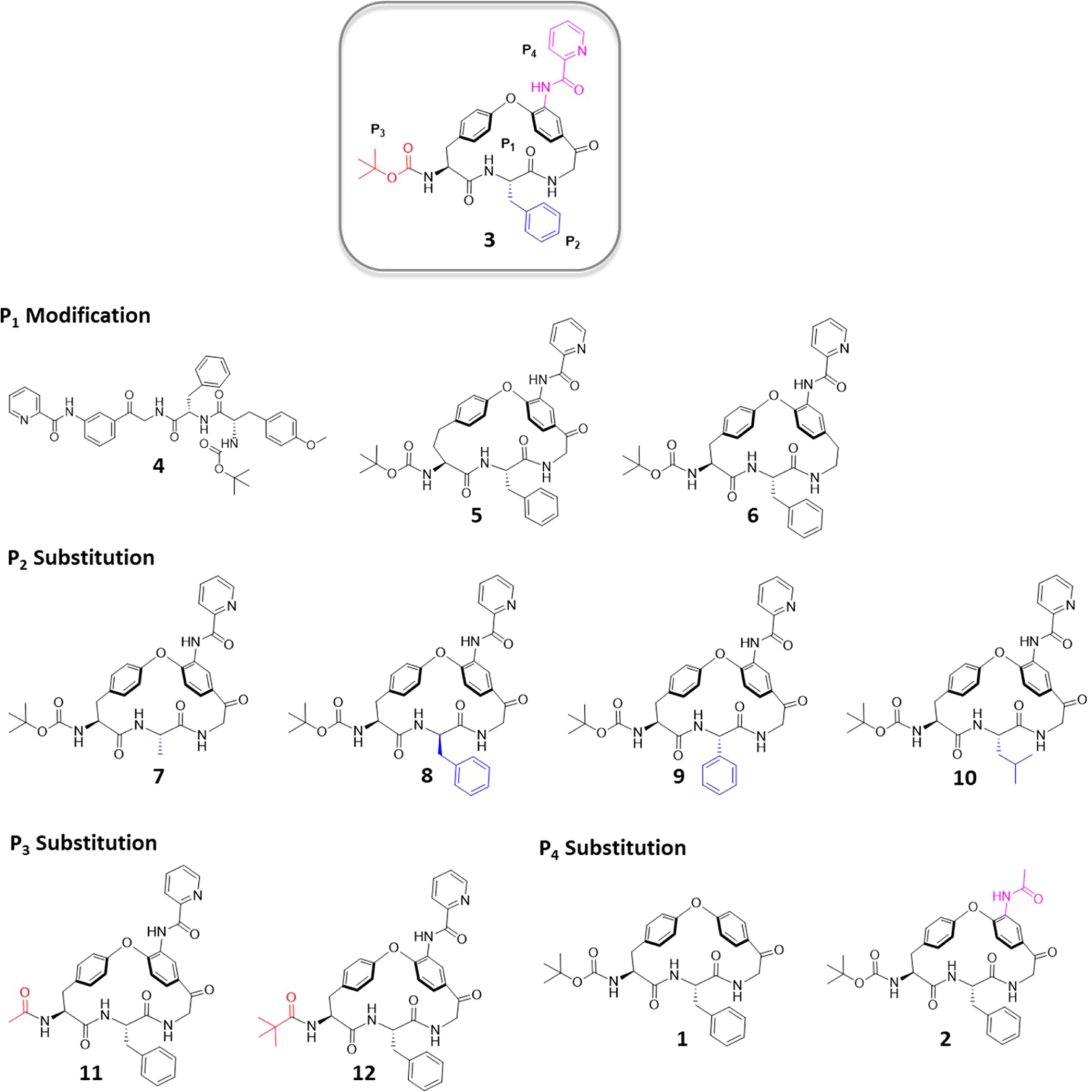
Macrocyclic (**1**-**3**, **5**–**12**) and linear (**4**) compounds designed to probe the structure–activity relationships of the series as inhibitors of the growth of *L. infantum*, as well as of *T. b. brucei*, *T. b. rhodesiense*, and *T. cruzi*.

The SAR exploration aimed to determine the role of both the macrocyclic scaffold and of its three substituents in the potency of the series (Figure 3). The macrocyclic scaffold (**P**_**1**_) and the **P**_**2**_ substituent must be chosen at the start of the synthetic route, while variations are possible for the **P**_**3**_ and **P**_**4**_ substituents toward the end of the route. Somewhat larger emphasis was, therefore, invested in exploring the macrocyclic scaffold and the **P**_**2**_ substituent than on the **P**_**3**_ and **P**_**4**_ substituents, with the aim of providing a lead compound suitable for subsequent optimization, first into a tool compound for mode of action studies and then into an oral drug.

Compound **4**, a ring-opened, non-macrocyclic analog of **3**, was chosen to probe the overall importance of the macrocyclic scaffold (**P**_**1**_) of the series (Figure 3). Compound **5**, in which the macrocyclic ring has been expanded by one methylene group, and **6**, in which the ketone has been deoxygenated, were designed to determine the influence of smaller structural variations of the scaffold on inhibitory potency. The importance of having a large and hydrophobic moiety in the **P**_**2**_ position was first investigated by substitution of the (*S*)-benzyl group of **3** with the smaller (*S*)-methyl group (**7**) and by inversion of stereochemistry [(*R*)-benzyl, **8**]. Additionally, a somewhat smaller (*S*)-phenyl group (**9**) and an (*S*)-*iso*-butyl moiety (**10**), maintaining the lipophilicity of **3**, were chosen for the **P**_**2**_ position. At the **P**_**3**_ position, the role of the acid-labile Boc group, which is also potentially sensitive to metabolic oxidation, was probed by acetyl (**11**) and pivaloyl (**12**) moieties. The importance of the **P**_**4**_ picolinoylated aniline had already been revealed by compounds **1** and **2** (Figure 2).

### Potency and Cell Permeability

The potency of macrocycles **1**–**12** to inhibit the growth of *L. infantum* was determined in a phenotypic assay in which the parasite was grown in primary peritoneal mouse macrophages (PMMs). To understand whether the SAR for inhibition of parasitic growth is impacted by low cell permeability, the permeability of compounds **1**–**12** was determined separately. Madin-Darby canine kidney (MDCK) cells transfected with the human MDR1 (multidrug resistance 1) gene (MDCK-MDR1 cells) were used to determine permeabilities in the apical to basolateral direction (*P*_app_ AB) as well as efflux ratios (ERs). Importantly, none of the compounds **1**–**12** showed any significant level of host cell toxicity (CC_50_ > 64 μM in PMMs). Lipophilicity (Log *D* at pH 7.4), aqueous kinetic solubility and mouse liver microsomal metabolism were also determined for **1**–**12**, and are discussed separately (cf. physiochemical properties and ADMET, below).

Of the 12 compounds, only the non-macrocyclic **4** had a very low (not quantifiable) cell permeability in the apical to basolateral direction across MDCK-MDR1 cells (Table 1). The permeabilities of the other compounds ranged from low for **2** and **9** (*P*_app_ AB 0.7 and 0.4 × 10^–6^ cm/s) to high for **6** (*P*_app_ AB > 5 × 10^–6^ cm/s), with most compounds having moderate permeability. Efflux ratios (ERs) were low or moderate. Except for the non-macrocyclic analog **4**, it is thus unlikely that the inhibitory potencies are significantly influenced by differences in cell permeability between the compounds.

**Table 1:** *In Vitro* Potency for Inhibition of the Growth of *L. infantum*, Cell Permeability, Physiochemical Properties, and *In Vitro* Clearance for Compounds **1**–**12**

compound	EC_50_ (μM)^a^	*P*_app_ AB (×10^–6^ cm/s)^b^	ER^c^	solubility (μM)^d^	Log *D*^e^	CL_int_^f^ (μL/min/mg)
1	33 (0.4, 2)	5.0	4.3	44	4.0	>580
2	31 (19, 7)	0.7	19	169	3.2	260
3	1.8 (1.8, 14)	3.5	6.4	<2.5	4.9	>580
4	38 (26, 2)	g		7.3	4.7	>580
5	17 (14, 4)	1.3	13	2.7	4.8	>580
6	12 (5.9, 4)	9.5	8.4	5.9	5.1	>580
7	16 (4.7, 2)	1.4	28	126	3.5	300
8	8.2 (0.1, 2)	2.9	7.6	2.9	4.8	>580
9	6.6 (1.5, 2)	0.4	9.9	16	4.4	210
10	2.1 (0.6, 2)	2.2	3.1	7.6	4.8	>580
11	38 (7.8, 4)	1.1	16	96	3.2	130
12	7.1 (1.0, 4)	1.5	21	16	4.4	>580
Miltefosine	6.1 (1.4, 7)					

a Mean values, with the standard deviation and the number of measurements in parentheses.

b Permeability across a MDCK-MDR1 cell monolayer in the apical-to-basolateral direction at pH 7.4.

c Efflux ratio (BA/AB) for the permeability across a MDCK-MDR1 cell monolayer.

d Kinetic solubility in phosphate buffered saline at pH 7.4, assay range 2.5–200 μM.

e Logarithm of the partition coefficient between 1-octanol and phosphate buffered saline at pH 7.4, determined by chromatography. Mean values from two measurements.

f Determined by incubation with mouse liver microsomes.

g Below the level of quantification.

The macrocyclic ring (**P**_**1**_) was found to be essential for potent inhibition of *L. infantum* in the cell-based phenotypic screen (Table 1). Macrocycle **4**, the ring-opened analog of **3**, was inactive in the phenotypic screen. However, as mentioned above, **4** was the only compound among **1**–**12** that had a cell permeability across MDCK-MDR1 cells below the level of quantification. This prevents us from concluding if the loss of activity was due to the very low cell permeability, a lack of potency on the target(s) or both. Ring-expanded macrocycle **5** and deoxygenated **6** were both 7- to 10-fold less potent than **3**, underscoring the importance of the macrocyclic ring present in **3** for the potency of the series. Since **5** and **6** did not display any major improvement in cell permeability or solubility over **3**, the 18-membered macrocyclic scaffold of **3** was considered as optimal for the SAR exploration of the **P**_**2**_–**P**_**4**_ positions.

Replacement of l-phenylalanine by l-alanine within the macrocyclic ring (**7**), i.e., replacing the **P**_**2**_ benzyl group by a methyl group, led to a large (close to 10-fold) drop in potency. The introduction of d-phenylalanine (**8**) and l-phenylglycine (**9**) also resulted in potency losses, whereas potency was regained by the l-leucine derivative **10**. Overall, the series of **P**_**2**_-substitutions suggested that a lipophilic substituent, which included a short flexible linker, is preferred over smaller and more rigid substituents at this position. At the **P**_**3**_-position the large drop in potency (>20 fold) of the *N*-acetyl derivative **11**, which was partly regained by *N*-pivaloyl derivative **12**, suggested a preference for a bulky lipophilic group attached via a carbamate or an amide bond to the α-amino group of the tyrosine moiety. The potency of **12** indicated that it might be possible to replace the acid-labile Boc-group with more stable groups. As already described above, the much higher potency of **3**, as compared to **1** and **2**, highlights that acylation of the aniline with an aromatic moiety is essential at the **P**_**4**_ position. We note that both **3** and **10** are more potent in the phenotypic assay than miltefosine. In addition, we note that **3** has activity against *Leishmania donovani* (EC_50_ 1.04 ± 0.25 μM) indicating potential to achieve *Leishmania* cross-species activity.

Compounds **1**–**12** were also evaluated as inhibitors of the growth of *T. b. brucei*, *T. b. rhodesiense*, and *T. cruzi*. For *T. b. brucei*, all compounds were either inactive or showed only low levels of inhibition (Table S1). Two of the macrocycles, **9** and **10**, were moderately active inhibitors of *T. b. rhodesiense*, while the other ten compounds had, at best, low activity. For *T. cruzi*, seven of the macrocycles had moderate activity, but none was highly potent. The finding that some of the compounds in this series inhibit *T. b. rhodesiense* and *T. cruzi*, as well as *L. infantum*, could suggest that they act at one or more targets common to kinetoplastid parasites. However, differences in SAR for the compounds in the series as inhibitors of the four parasites indicate differences in the structure of the target(s) between parasites, or in the ability of the compounds to reach them.

In summary, SAR investigations identified the 18-membered macrocyclic scaffold (**P**_**1**_), common to all but compounds **4**–**6** in the series, as essential for potent inhibition of the growth of *L. infantum*. Compounds with a lipophilic aromatic or aliphatic side chain in the **P**_**2**_ position were highly potent inhibitors. Moreover, a lipophilic group attached via a carbamate or an amide bond in the **P**_**3**_ position, and an aromatic amide in the **P**_**4**_ position, were tentatively concluded to be important for potency. Finally, the current lead compounds, **3** and **10**, have potencies that surpass that of miltefosine, with cell permeabilities that are favorable for oral absorption.

### Macrocycle Conformation and Potency

As revealed by loss in potency for the nonmacrocyclic **4**, ring expanded **5** and deoxygenated **6**, the presence of the macrocyclic ring, and its structural features, are essential for the potency of this series of leishmaniasis inhibitors (Table 1). The close to 10-fold loss in potency of **5**, which only differs from **3** by the insertion of a single methylene group to give a 19-membered ring, is intriguing. In order to gain insight into the reasons for this difference in potency, we performed a conformational analysis of **3** and **5**, using a protocol found to provide an accurate description of analogs of **3** which lacked the acylated aniline.^21^ Briefly, the protocol employed Monte Carlo conformational sampling in an implicit nonpolar environment, followed by clustering and quantum mechanical (QM) energy minimization of the cluster centers.^21^

**Figure 4:**
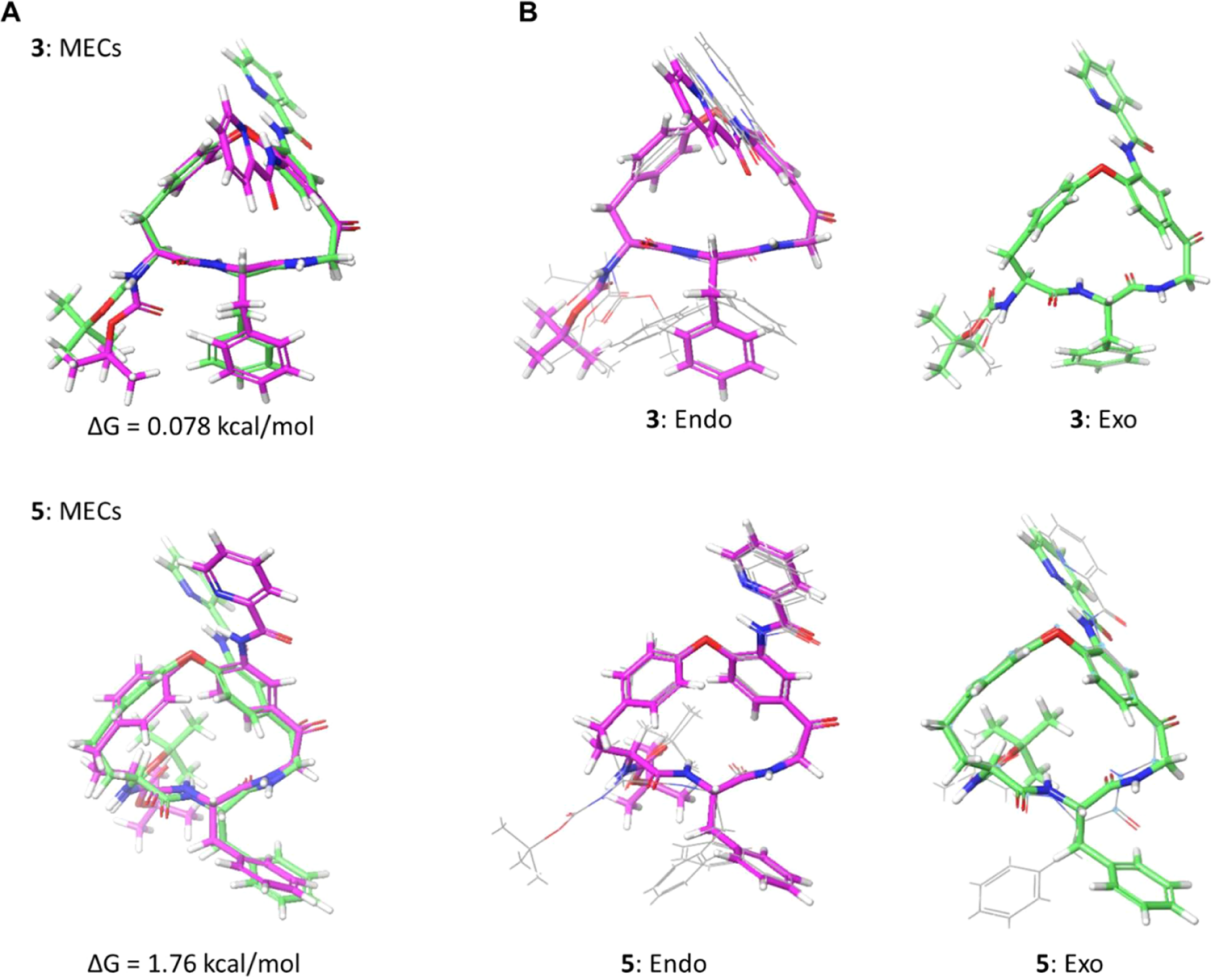
(A) Overlays of the minimum energy conformation (MEC) of the *endo* and *exo* rotamers in the predicted ensembles of **3** and **5**. (B) Overlays of the predicted MECs of the *endo* and *exo* rotamers of **3** and **5** with the conformations having QM energies within 5 kcal/mol of the corresponding MEC. *Endo* and *exo* MECs have bonds in magenta and green, respectively, while the bonds of other conformations are in gray in panel B. Figures were made by overlaying the heavy atoms of the macrocyclic core.

Conformational sampling revealed that the **P**_**4**_ picolinoylated aniline, characteristic of this series of inhibitors of *L. infantum*, can be positioned in an “*endo*” or “*exo*” orientation with regards to the **P**_**2**_ phenyl group (Figure 4A). Conformational analysis revealed that minimum energy conformations (MECs) of **3** having the picolinoylated aniline in the *endo* and *exo* orientations did not differ in energy, while the energy difference between the corresponding MECs of **5** was not significant (≤5 kcal/mol) (Figure 4A). For the 18-membered **3**, the macrocyclic backbone adopted identical conformations in the *endo* and e*xo* MECs (Figure 4A, top). Except for some minor variation for some *endo* conformations, this backbone conformation was preserved for the other low energy conformations of **3** found within 5 kcal/mol of the MECs; only the phenylalanine side chain and the Boc-protected amine were flexible (Figure 4B, top; Figure S1). In contrast, the large difference in the macrocyclic backbone between the two MECs of 19-membered **5** indicated the backbone of **5** to have significant flexibility (Figure 4A, bottom). In line with this, the backbone of **5** also differed between some of the conformations found within 5 kcal/mol of the MECs (Figure 4B, bottom; Figure S2). In addition to the difference in flexibility found between the backbones of **3** and **5**, the Boc-protected amine adopted different orientations in the two macrocycles. In **3**, all low energy conformations had this side chain oriented “equatorially” on the macrocyclic ring, while it adopted an “axial” orientation in all but one of the conformations of **5** (Figure 4B). In conclusion, conformational analysis identified an increased flexibility of the macrocyclic ring of **5**, and a different orientation of the Boc-protected amine, as causing the loss of potency compared to **3**.

### Physiochemical Properties and *In Vitro* ADMET

**Figure 5:**
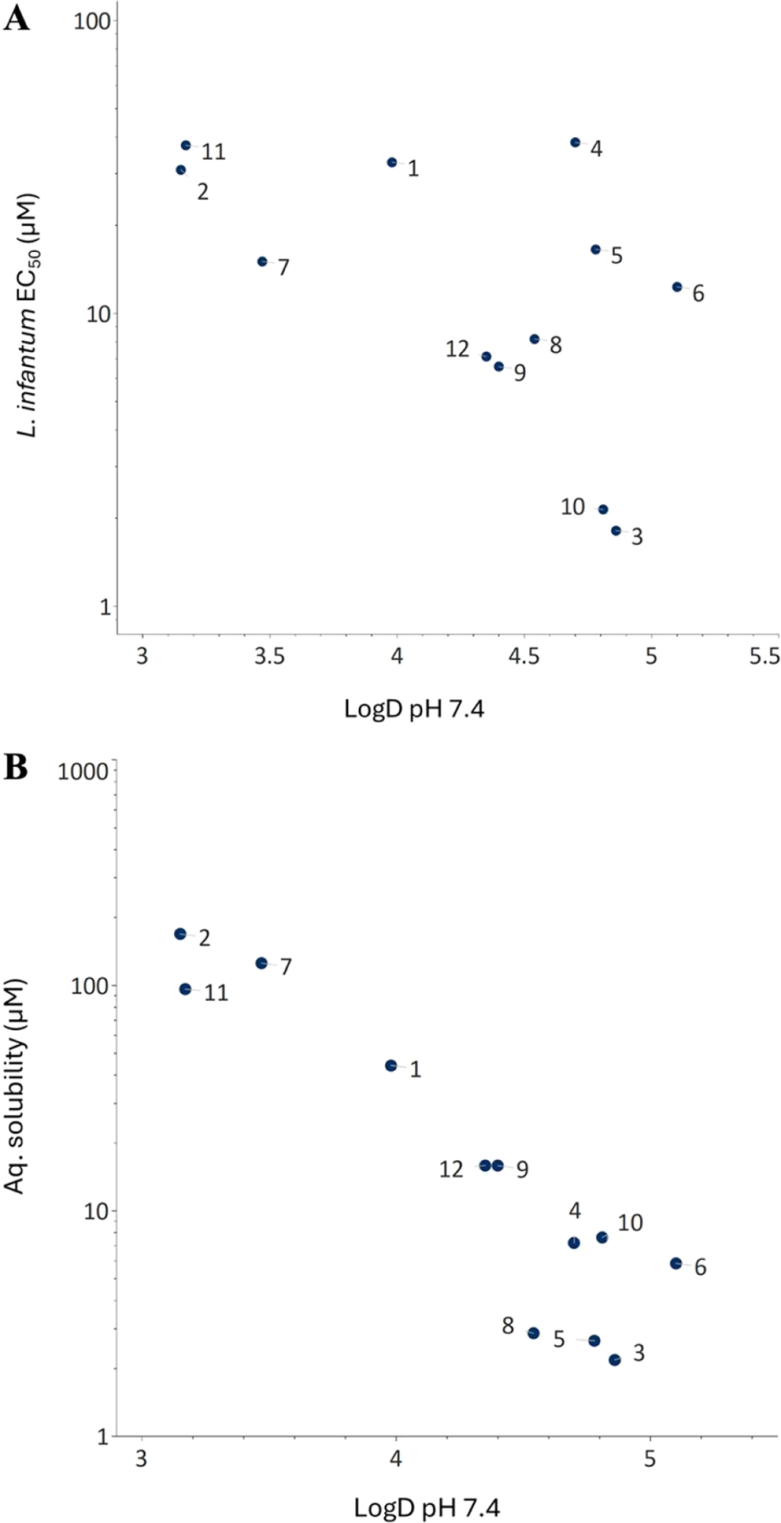
Correlations between Log *D*_7.4_ and (A) the potency of macrocycles **1**–**12** to inhibit the growth of *L. infantum* and (B) kinetic solubilities in phosphate buffered saline at pH 7.4.

The permeabilities of compounds **1**–**12** across MDCK-MDR1 cells ranged from low (*P*_app_ AB < 1 × 10^–6^ cm/s) to high (*P*_app_ AB ≥ 5 × 10^–6^ cm/s), while efflux ratios were low to moderate (<30, six compounds had ER < 10) (Table 1). Most compounds show low to moderate kinetic aqueous solubility (<50 μM), but **2** and **7** had high solubilities (>100 μM). Lipophilicities varied from druglike (Log *D* just over 3) to high for **6** (Log *D* 5.1), while clearance by mouse liver microsomes was high for all of **1**–**12** (>100 μL/min/mg, Table 1). As low solubility and high clearance often correlate with high lipophilicity, we investigated the relationship between the properties of the compounds and Log *D* to further understand the scope and limitations of the series (Figures 5, S4). As is frequently found in drug discovery projects, the series shows a “leading edge” where the potency of the compounds is correlated with Log *D* (Figure 5A). However, compounds **1**, **5**, and **6** and the linear control **4** do not adhere to this correlation. Compounds **3**, **5**, **6**, and **10** that have Log *D* values between 4.8 and 5.1, and that differ 10-fold in potency, illustrate that antileishmanial potency is also determined by specific interactions between the compounds and the target(s). As expected, aqueous solubility was inversely correlated with Log *D* (Figure 5B), while clearance was too high for most compounds to establish a meaningful correlation with Log *D*. For the most potent compound, macrocycle **3**, metabolite identification revealed oxidative metabolism occurring at the **P**_**2**_ and **P**_**3**_ positions, and at the α-carbon atom of the tyrosine moiety in the macrocyclic ring (Figure S3). Other potential correlations, such as those between permeability across MDCK-MDR1 cells and Log *D*, and between potency and cell permeability, lacked statistical significance (Figure S4). Overall, these correlations suggest reduction of compound lipophilicity as one approach to increasing solubility and lowering metabolism, while attempting to maintain or improve potency. A comparison of the lipophilicity and potency of compounds **5** and **6** to those of **8**, **9**, and **12** indicates that it could be possible to reduce lipophilicity and increase potency (Figure 5A). As discussed in the following section, reduction of crystal packing interactions is also an attractive approach to improving the solubility of potent members of the series.

### Solid State and Solubility Predictions

**Figure 6:**
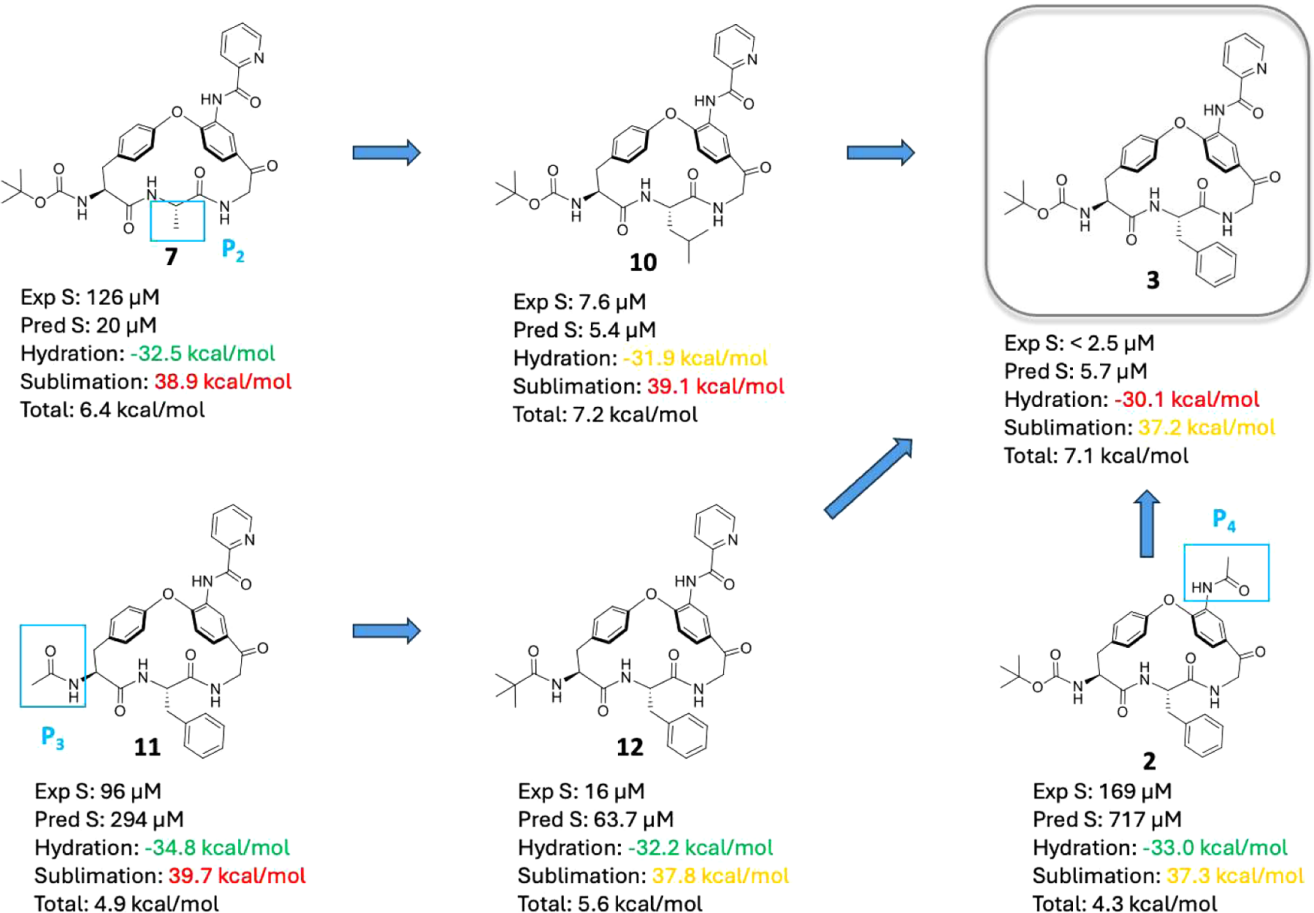
Comparison of the experimentally determined kinetic solubilities (PBS, pH 7.4) with predicted amorphous solubilities for macrocycles **2**, **3**, **7**, and **10**–**12**. Calculated hydration and sublimation free energies have been included and color coded based on their respective contributions to solubility. Red indicates less favorable energies, green more favorable ones while orange indicates an intermediate contribution. The total free energy for solubilization of amorphous material is also given for each macrocycle. The **P**_**2**_, **P**_**3**_, and **P**_**4**_ position is indicated for macrocycles **7**, **11**, and **2**, respectively.

The most potent compound, macrocycle **3**, had a low kinetic aqueous solubility (<2.5 μM) which, in part, appears to be linked to its high lipophilicity (Log *D* 4.9). Macrocycles **2**, **7**, and **10**–**12** have Log *D* values that vary from 3.2 to 4.8 due to differences in the structures of the **P**_**2**_, **P**_**3**_, and **P**_**4**_ substituents and kinetic solubilities that range from significantly higher to similar to that of **3** (Figure 6). To understand the energetic origins of how structural modifications impact the aqueous solubility, we calculated the amorphous solubility of these compounds using a physics-based free energy perturbation (FEP) approach in which an amorphous aggregate is used to model the material used for determination of the kinetic solubilities.^22−24^ This approach predicts the aqueous solubility by calculating the free energy difference between a molecule in an amorphous aggregate and water, through the respective energetic contributions of sublimation and hydration. Overall, the predictions reveal a reasonable correlation between predicted and experimentally determined solubilities for the six macrocycles (Figure 6), indicating its use in future design efforts.

A reduction in the lipophilicity and size of the **P**_**2**_, **P**_**3**_, and **P**_**4**_ substituents as compared to in **3** modulated the hydration energy so that the predicted solubility increased, with **11** displaying the largest contribution (−4.7 kcal/mol, Figure 6). Strengthened solid-state interactions in the amorphous state, as quantified by increases in the sublimation free energy, influenced the calculated solubilities less; **11** showed the largest increase (2.5 kcal/mol higher than **3**). In addition, the contributions from the sublimation free energies were balanced by the larger changes in hydration energies. We hypothesize that the bulky substituents of **3** shield neighboring polar groups from interactions with water, thereby reducing the contribution of the hydration free energy to solubility as compared to **7** (**P**_**2**_ position), **11** (**P**_**3**_), and **2** (**P**_**4**_) which all have more favorable hydration energies. For **10**, the two energies had opposite contributions, yielding an almost similar predicted solubility as for **3**. Surprisingly, we also observed an increased contribution to the hydration energy through replacing the **P**_**3**_*tert*-butyloxycarbonyl group in **3** with a pivaloyl group (cf. **12**). This was explained by analysis of the averaged solvent accessible surface area of the carbamate/carbonyl oxygen atom of the **P**_**3**_ substituent throughout the FEP MD simulation which revealed a larger solvent accessible area for **12** (19.9 Å^2^) than for **3** (15.3 Å^2^). The improved hydration of **12** is also confirmed by its lower Log *D* (4.4 as compared to 4.9 for **3**).

We attempted to crystallize **3** to understand if the thermodynamic solubility from crystalline material would be even lower than from amorphous material, and if this could pose additional developability challenges. However, with the limited amounts of material available at this stage of the project those efforts were not successful. The high MW (664 Da) and flexibility (NRotB: 9, Kier flexibility index:^25^ 7.8) of **3** are likely to have contributed to these difficulties.^26^ We therefore turned to Crystal Structure Predictions (CSPs) for **3**, since current CSP approaches have been found to provide excellent replicates of experimentally determined structures.^23,24,27^ These were followed by calculation of the crystalline thermodynamic solubility through the physics-based FEP approach, using the most energetically stable predicted crystal structure.^24^

**Figure 7:**
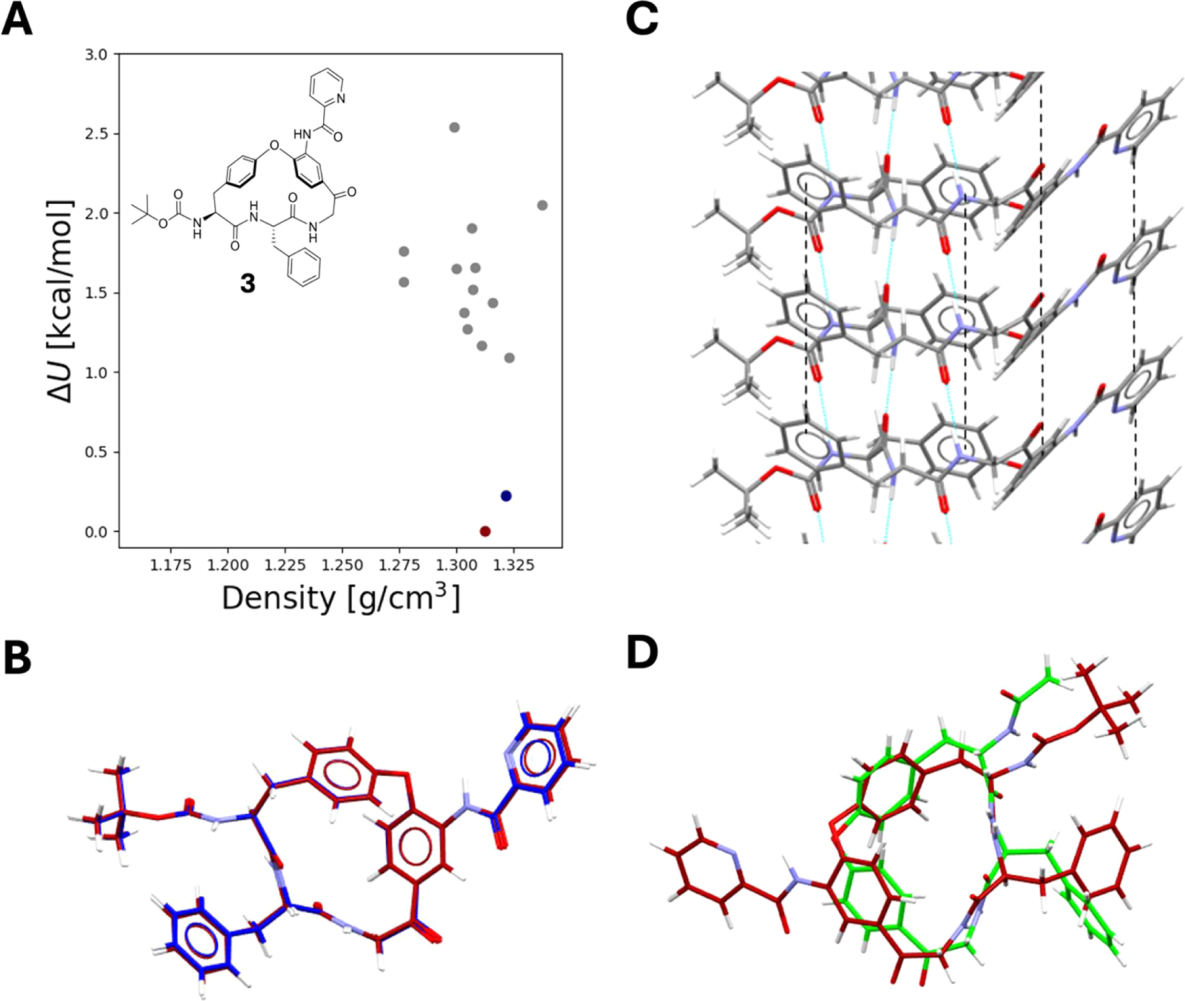
(A) CSP landscape of macrocycle **3** showing energies relative to the most stable predicted crystal structure and densities of the predicted structures. The most stable structure is indicated in dark red, the second most stable in blue and higher energy structures are in gray. (B) Conformational overlay of the two most stable crystal structures, with the most stable one in dark red. (C) Crystal packing interactions of **3**, where the dotted blue lines indicate intermolecular hydrogen bonds and the dotted black lines indicate π–π interactions between neighboring aromatic rings. (D) Conformational overlay between the crystal conformation in **3** (in dark red) and that of its structural analog **1** that lacks the **P**_**4**_ substituent (in green, CCDC REF: KIKZOW).

CSP provides not only a single predicted crystal structure, but a multitude of possible low-energy structures, describing various ways a molecule can interact in the crystalline solid state. The predicted CSP landscape of macrocycle **3** is characterized by two distinct low-energy crystal structures and a multitude of higher-energy structures (Figure 7A). While the two low energy structures pack in two distinct space groups (*P*2_1_2_1_2_1_ and *C*2, respectively), **3** exhibits identical conformations in both (Figure 7B). Moreover, the crystal packing patterns are almost identical in these two low-energy structures, involving similar hydrogen bonding and π–π interaction chains (Figure 7C). Interestingly, the higher energy crystal structures predicted also exhibit similar types of interactions. This suggests that interactions involving π–π stacking and hydrogen bonding along the same direction, will be extremely prevalent for this molecule across crystal forms.

As discussed above, the amorphous solubility of macrocycle **3** was calculated to be 5.7 μM, consistent with the observed low experimental amorphous solubility (<2.5 μM), while the crystalline thermodynamic solubility based on the most stable predicted crystal structure, was calculated to be as low as 0.03 μM. Further assessment of the crystalline solubility prediction showed that while **3** exhibited strong interactions with water, with a hydration energy of −30.0 kcal/mol, it also exhibited extremely strong intermolecular interactions in the crystalline solid state, with a sublimation energy of 40.3 kcal/mol. The very well-ordered hydrogen bonding and π–π interaction chains going in the same direction in the predicted structure (Figure 7C) should result in significant stabilization of the crystalline solid and, therefore, the high sublimation free energy.

To gather insights on how structural substituents or modifications at the **P**_**2**_–**P**_**4**_ positions may impact the crystal packing, we compared the most stable crystal structure predicted for **3** with the reported crystal structure of analog **1** that lacks the **P**_**4**_ picolinoylated aniline (CCDC REF: KIKZOW)^15^ (Figure 7D). To understand the energetic contributions of the crystal structure of **1** on its crystalline solubility, we performed FEP solubility calculations using this experimental structure. As compared to **3**, **1** had a somewhat lower hydration energy (−28.3 kcal/mol), but a significantly lower sublimation energy (33.1 kcal/mol), with an overall predicted crystalline solubility of 292 μM, i.e., four orders of magnitude higher than for **3**. The lower sublimation energy of **1** suggests a large impact of π–π interactions between the aromatic, picolinoyl groups in the **P**_**4**_ position in stabilizing the crystal lattice of **3**, resulting in the very low predicted thermodynamic solubility.

While the conformation of the macrocyclic core and the intermolecular hydrogen bonding chains appear to be very similar in **1** and **3**, the ordered π–π stacking network does not appear in the crystal structure of the analog **1**. This suggests that the additional aromatic side-chain substituent in the **P**_**4**_ position of **3** is crucial for inducing the crystal packing interactions that also include π–π stacking at the **P**_**2**_ position. Disrupting aromaticity in the **P**_**4**_ position, or in the **P**_**2**_ position, leading to weakened π–π interactions in the crystalline state thus appears as an attractive approach for increasing the solubility of the inhibitors in the series. Alternatively, weakening of the hydrogen bonding chains in the crystal could improve solubility.

### Synthetic Chemistry

**Scheme 1:**
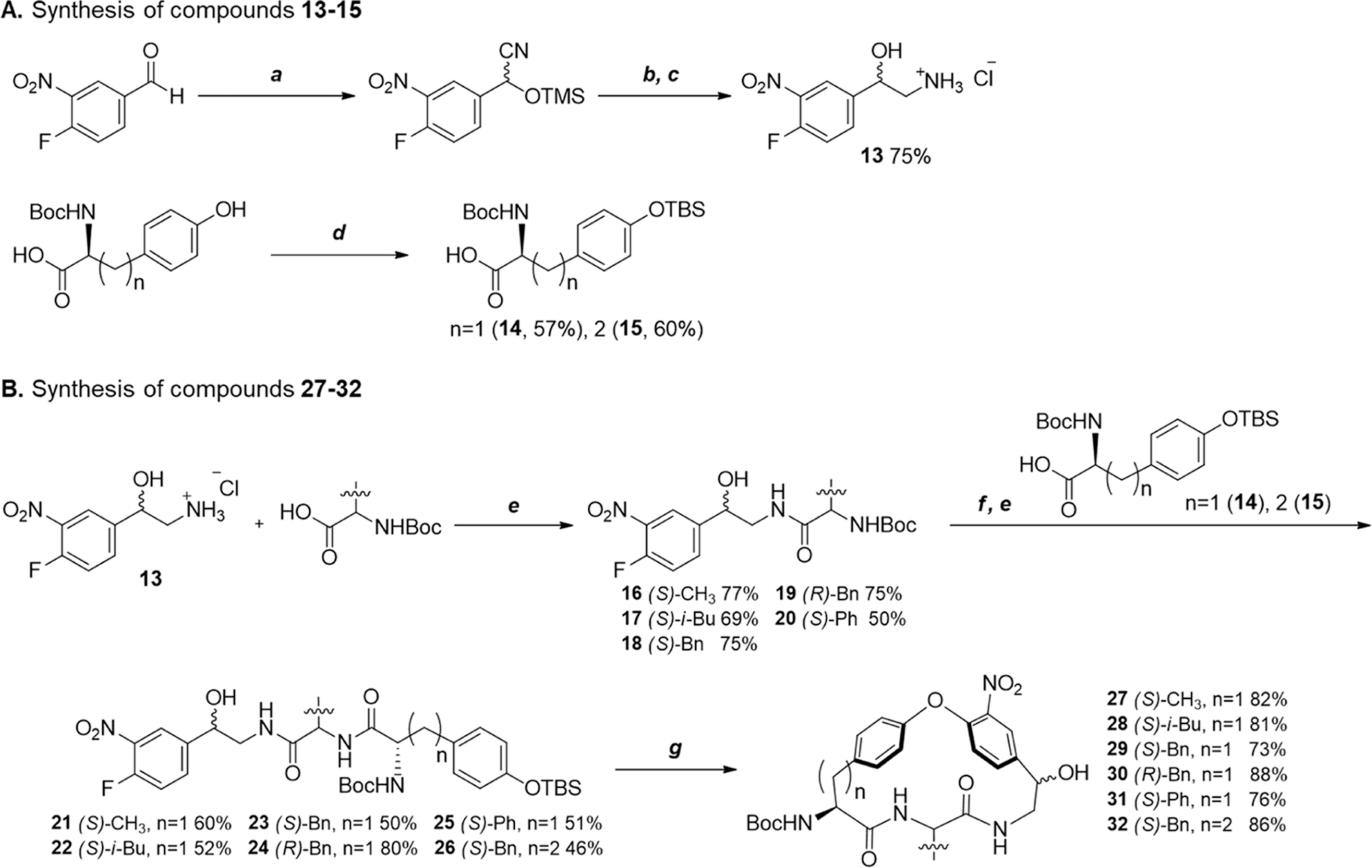
Synthesis of Compounds **27**–**32** Reagents and conditions: (a) TMSCN, ZnI_2_, CH_2_Cl_2_, rt, 1 h; (b), BH_3_ tetrahydrofuran (THF), reflux, 3 h; (c) 4 N HCl/DOX (dioxane), rt, 30 min; (d) TBSCl, 1*H*-imidazole, DCM, rt, 18 h; (e) HATU, DIPEA, CH_2_Cl_2_, rt, 30–40 min; (f) 4 N HCl/DOX, rt, 30 min; (g) CsF, DMF, 50–70 °C, 3 h.

**Scheme 2:**
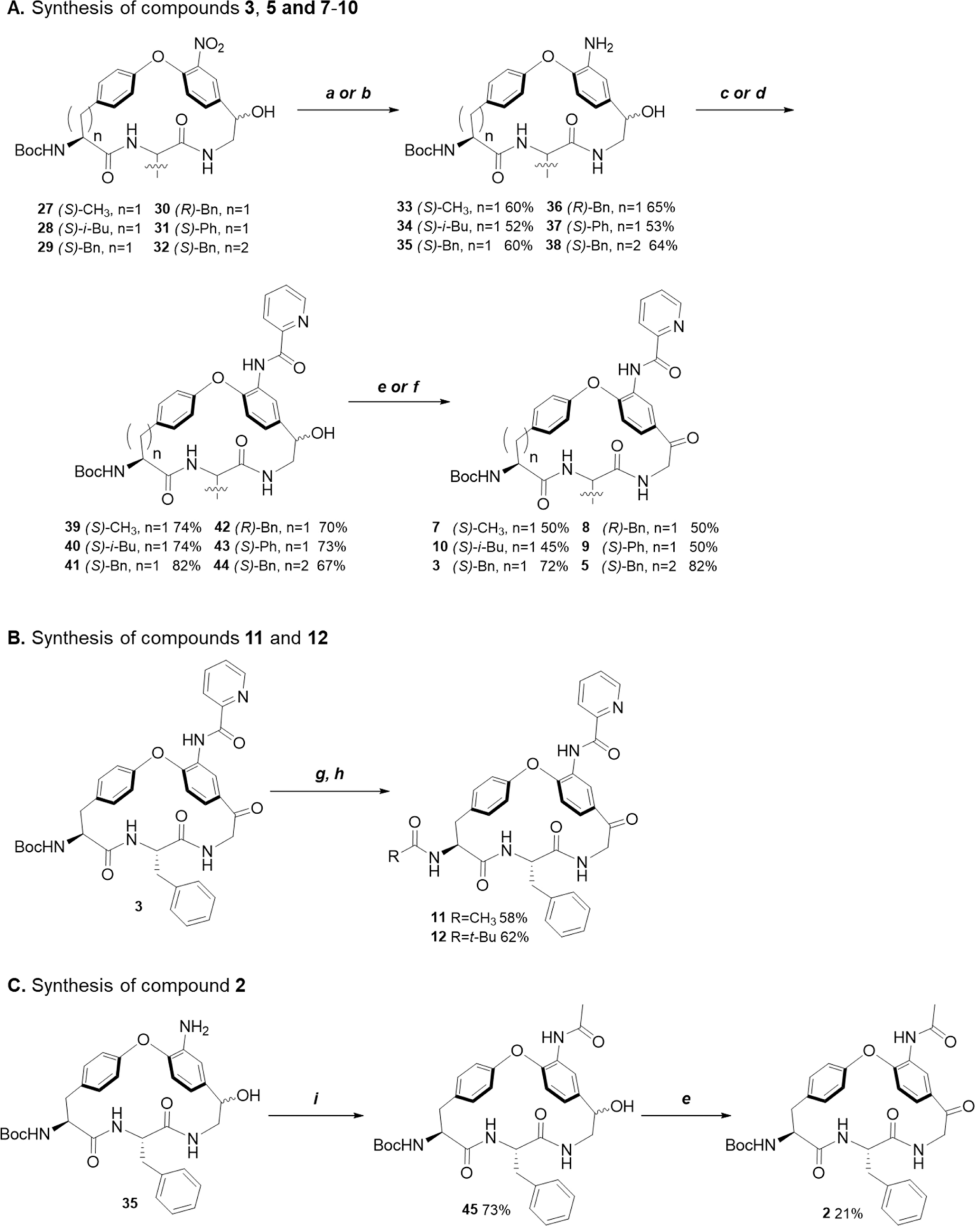
Synthesis of Compounds **2**, **3**, **5**, and **7**–**12** Reagents and conditions: (a) Fe/NH_4_Cl, EtOH/H_2_O (6:1), reflux, 3 h; (b) Pd/C, MeOH, rt, 1 h 30 min; (c) picolinoyl chloride, DIPEA, 4-dimethylaminopyridine (DMAP), DMF, rt, 30 min; (d) picolinic acid, HATU, DIPEA, rt, 30 min; (e) IBX, EtOAc-DMSO, reflux, 2–3 h; (f) Dess–Martin periodinane (DMP), DCM, rt, 2 h; (g) trifluoroacetic acid (TFA), DCM, rt, 3 h; (h) AcCl for **11** or, pivaloyl chloride for **12**, triethylamine (TEA), DCM, rt; (i), Acetic anhydride, DIPEA, DMAP, THF, rt, 2 h.

**Scheme 3:**
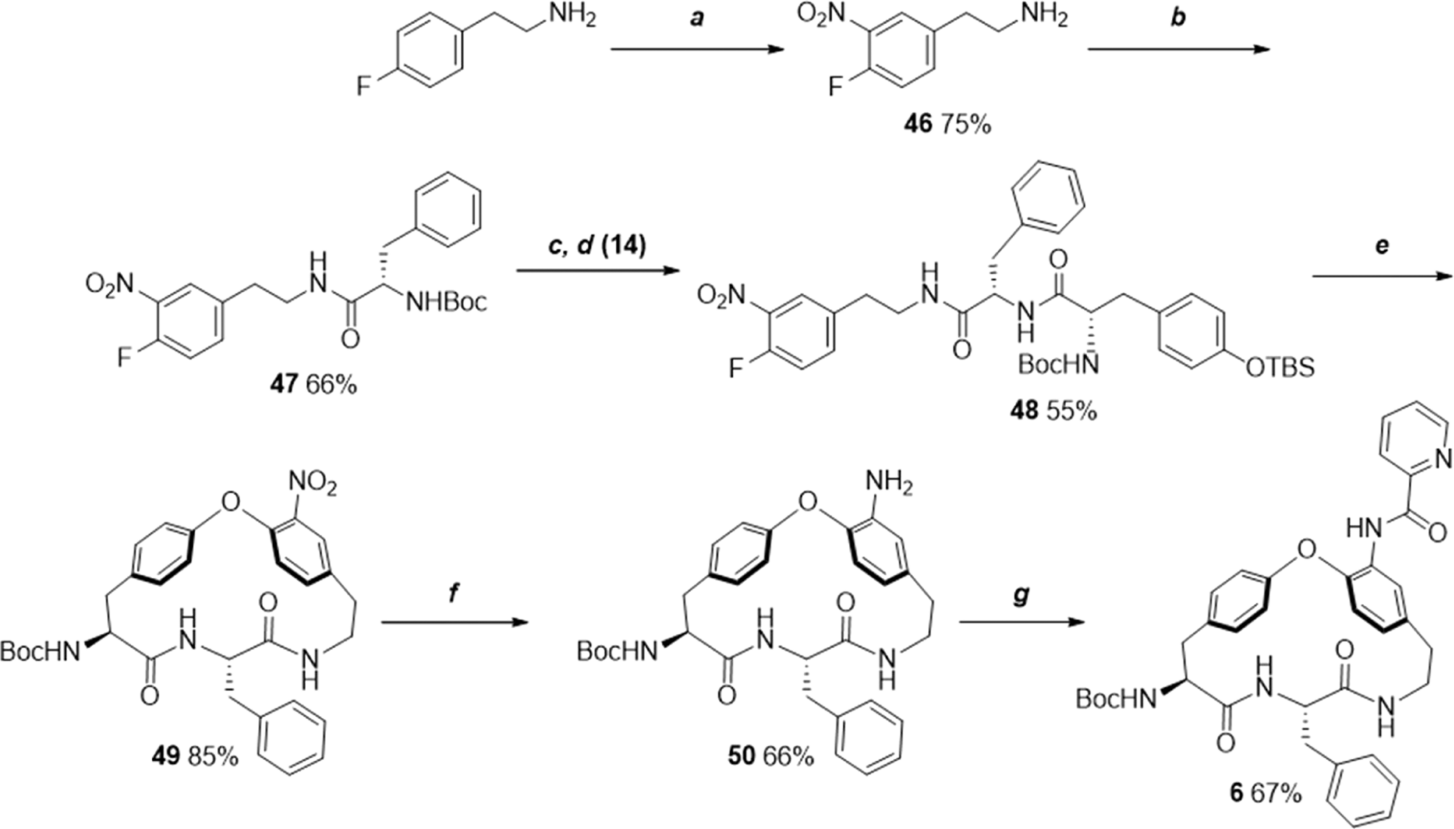
Synthesis of Compound **6** Reagents and conditions: (a) HNO_3_ fuming, H_2_SO_4_ conc.; (b) Boc-l-Phe-OH, HATU, DIPEA, CH_2_Cl_2_, rt, 40 min; (c) (1) 4 N HCl/DOX, rt, 30 min; (d) HATU, DIPEA, CH_2_Cl_2_, rt, 40 min; (e) CsF, DMF, 50 °C, 3 h, (f) Fe/NH_4_Cl, EtOH/H_2_O, reflux, 2 h; (g) picolinoyl chloride, DIPEA, DMAP, DMF, rt, 40 min.

The syntheses of the macrocyclic members of the series of *Leishmania* inhibitors relied on the preparation of nitrated macrocycles **27**–**32** as key intermediates (Scheme 1) that were then converted into **1**–**3** and **5**–1**2** (Schemes 2 and 3). The route reported^15^ for the synthesis of **29** in the preparation of macrocycle **1** also proved to be robust for **27**, **28**, and **30**–**32**, requiring only minor adjustments of some of the conditions of the different steps. First, racemic amino alcohol building block **13**(28,29) and the TBS-protected tyrosine derivative **14**(30−32) were prepared as reported, and homotyrosine **15** was obtained by TBS protection (Scheme 1A). Then building block **13** was coupled with the Boc-protected amino acids to be incorporated at the **P**_**2**_-position of the macrocycles using hexafluorophosphate azabenzotriazole tetramethyl uronium (HATU) and *N*,*N*-diisopropylethylamine (DIPEA) in dichloromethane (DCM) at room temperature (Scheme 1B). The Boc group of synthetic intermediates **16**–**20** was then deprotected by treatment with HCl in acetonitrile, followed by coupling with the TBS-protected tyrosine and homotyrosine building blocks **14** and **15**, to give the linear intermediates **21**–**26**. In general, yields were somewhat lower (50–60%) in the second coupling compared to the first coupling (70–75%), even though both were performed with HATU and DIPEA. The subsequent macrocyclization was induced by cesium fluoride-promoted TBS deprotection of the phenol of the tyrosine/homotyrosine moiety, which then engaged in an intramolecular nucleophilic attack on the fluorine atom of the nitrated phenyl ring. Nitrated macrocycles **27**–**32** were obtained in 73–88% yields through macrocyclization via an intramolecular S_N_Ar reaction,^33,34^ which by far exceeds the 20–30% often encountered in macrocyclizations,^35^ such as the one reported for vancomycin.^36,37^

Somewhat surprisingly, the three steps remaining for the conversion of intermediates **27**–**32** into the target macrocycles required tailoring reagents and reaction conditions for individual compounds (Scheme 2A). Reduction of the nitro group of **27**–**32** to an aniline was performed either with iron and ammonium chloride in a refluxing mixture of EtOH and water (to give compounds **36** and **38**), or with Pd/C in MeOH for those compounds that were not prone to being reduced with Fe/NH_4_Cl (providing **33**, **34**, **35** and **37**). The aniline intermediate was then *N*-acylated either with picolinoyl chloride (for **35** and **38**) or with picolinic acid under promotion by HATU and DIPEA (for **33**, **34**, **36**, and **37**) to give **39**–**44**. The final step was the oxidation of the alcohol in **39**–**44** to a ketone, using 2-iodoxybenzoic acid (IBX) to give macrocycles **3**, **5**, and **8**, or with Dess–Martin periodinane^38−40^ to give **7**, **9**, and **10**. Deprotection of the Boc group of **3**, and subsequent coupling with acetyl chloride or pivaloyl chloride gave macrocycles **11** and **12** (Scheme 2B). Compound **2** was obtained by acetylation of aniline **35** to provide **45** followed by oxidation of the secondary alcohol to a ketone (Scheme 2C).

Compound **6** lacks the macrocyclic ketone and its synthesis was first attempted by a Wolff–Kishner reduction of **3** with hydrazine, but the reaction was not successful. Hence, it was decided to start from the beginning of the synthetic route by preparation of building block **46** lacking the hydroxyl group of **13** (Scheme 3). The subsequent route to **6** was similar to that described above, i.e., it involved the coupling of **46** with Boc-l-phenylalanine (**47**), then with the TBS-protected tyrosine **48** followed by macrocyclization (→ **49**), reduction of the nitro group (→ **50**) and finally oxidation of the hydroxyl group to a ketone.

**Scheme 4:**
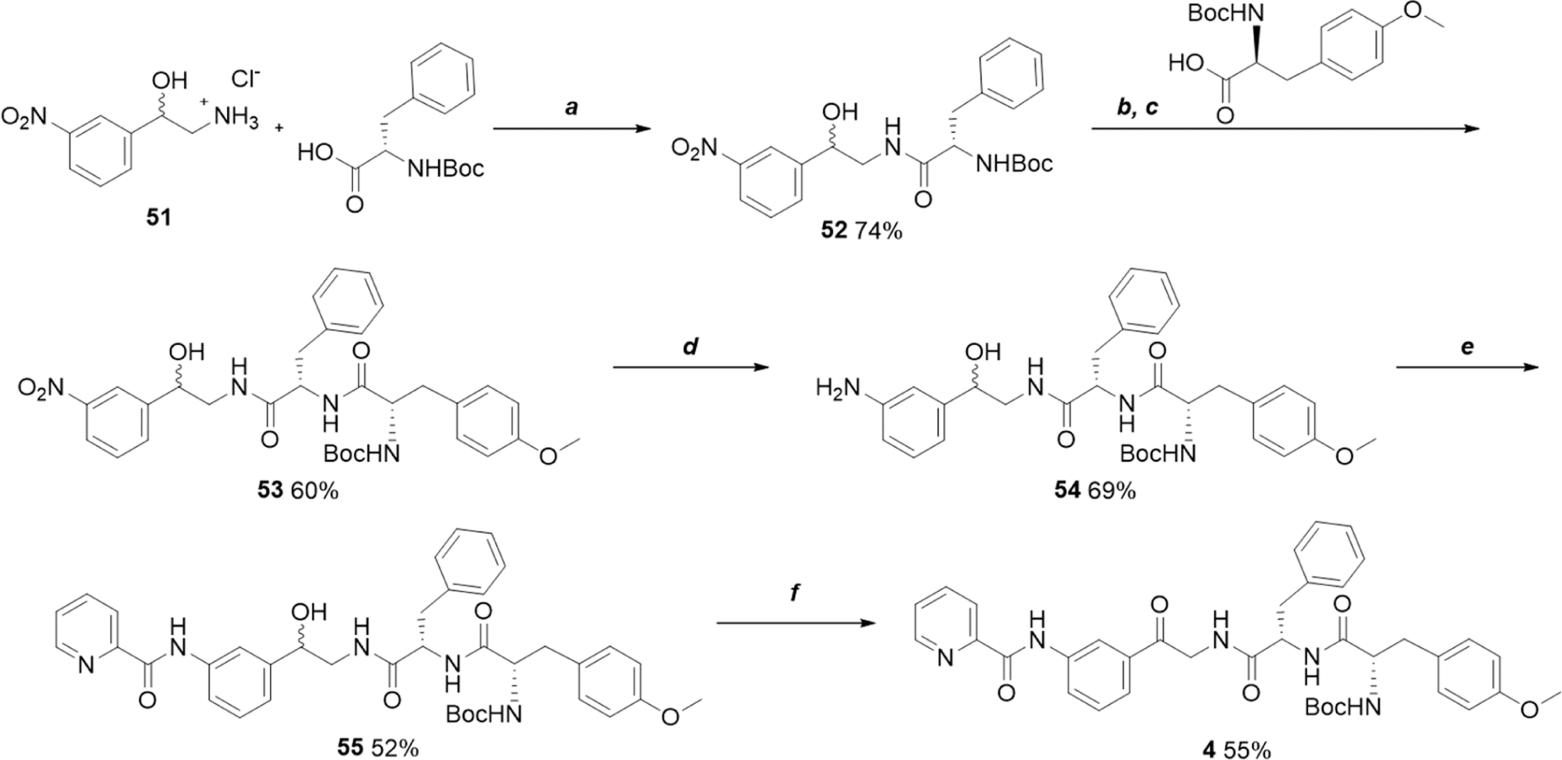
Synthesis of Compound **4** Reagents and conditions: (a) HATU, DIPEA, CH_2_Cl_2_, rt, 40 min; (b) 4 N HCl/DOX, rt, 30 min; (c) HATU, DIPEA, CH_2_Cl_2_, rt, 40 min; (d) Fe/NH_4_Cl, EtOH/H_2_O, reflux, 2 h; (e) picolinoyl chloride, DIPEA, DMAP, DMF, rt, 40 min; (f) IBX, EtOAc-DMSO, reflux, 3 h.

The linear compound **4** was synthesized starting from the amino alcohol building block **51**, lacking the fluorine atom of **13** (Scheme 4). The synthetic route involved the coupling of **51** with Boc-l-phenylalanine (→ **52**), subsequent coupling with *O*-methylated Boc-l-tyrosine (→ **53**), reduction of the nitro group to an aniline (**54**), coupling with picolinoyl chloride (→ **55**) and final oxidation of the primary alcohol to the desired ketone (**4**).

## Discussion and Conclusions

Neglected tropical diseases are a group of parasitic, bacterial, fungal and viral diseases that have been estimated to affect up to 2.7 billion people living in low and middle-income countries of Africa, Asia, and Latin America.^41,42^ Macrocyclic natural products and derivatives thereof are essential as drugs for the treatment of infectious diseases, including neglected tropical diseases.^1^ Prominent examples include the rifamycin classes of antibiotics used for the treatment of tuberculosis and leprosy, as well as vancomycin, the last resort for the treatment of serious, life-threatening infections. Amphotericin B is used for serious fungal infections and for the treatment of leishmaniasis, but requires lengthy intravenous injections. Ivermectin and moxidectin are two orally administered macrocyclic natural products that are used for the treatment of river blindness and other parasitic infections.

Herein, we explored a series of macrocycles based on their structural similarity to the natural product hymenocardine, an inhibitor of the growth of *P. falciparum* responsible for the most virulent form of human malaria.^16^ We found that macrocycles in the series inhibited *P. falciparum* and the kinetoplastid parasites *T. b. brucei*, *T. b. rhodesiense*, *T. cruzi*, and *L. infantum* in phenotypic screens, but that potencies varied between the different parasites. This cross-species activity may indicate that the macrocycles act by a similar mechanism in the parasites; kinetoplastid parasites have similar genetics and biology with cross-reactivity within compound classes frequently being observed.^43−45^ In particular, we discovered two compounds (**3** and **10**) that were more potent than the only oral drug for leishmaniasis, miltefosine, in a screen against *L. infantum*. We were hopeful that this series reported herein would show activity due to the structural similarity with hymenocardine, but had not expected to discover a highly potent compound such as **3** among the first few compounds that were evaluated in the phenotypic screen. Exploration of the SAR for the series showed that the macrocyclic ring (**P**_**1**_) was essential for the potent inhibition of *L. infantum*. Lipophilic groups such as a benzyl or isobutyl group, were preferred at the **P**_**2**_ position, while the presence of a *tert*-butyl group at **P**_**3**_ and an aromatic amide at **P**_**4**_ also appeared essential for potency. We note that the potent inhibitors **3** and **10** have satisfactory permeability across MDCK-MDR1 cell monolayers and a low efflux ratio, revealing their potential for optimization into orally bioavailable drugs. Structure–property relationships revealed that the low aqueous solubility and the low metabolic stability of potent macrocycles, such as **3** and **10**, originated, at least in part, from high lipophilicity.

Physics-based modeling of the solubility of amorphous aggregates reproduced the experimentally determined kinetic solubilities of several of the compounds in the series reasonably well, indicating that these calculations can be used to design and prioritize compounds for synthesis. In addition, these calculations highlighted an important role of hydration free energies for improving kinetic solubility. Importantly, the prediction of crystal structures of the most potent inhibitor **3**, a flexible bRo5 macrocycle, indicated a high packing efficiency resulting in a high sublimation energy contribution to the crystalline solubility. Accordingly, the crystalline solubility was estimated to be very low for **3** (0.03 μM), which would pose a major challenge in attempts to use **3** as a tool compound for mode of action studies. The predicted crystal structure suggested that future optimization of the series should also focus on the reduction of π–π interactions to reduce the sublimation energy and improve the aqueous solubility, in addition to lowering of lipophilicity.

**Figure 8:**
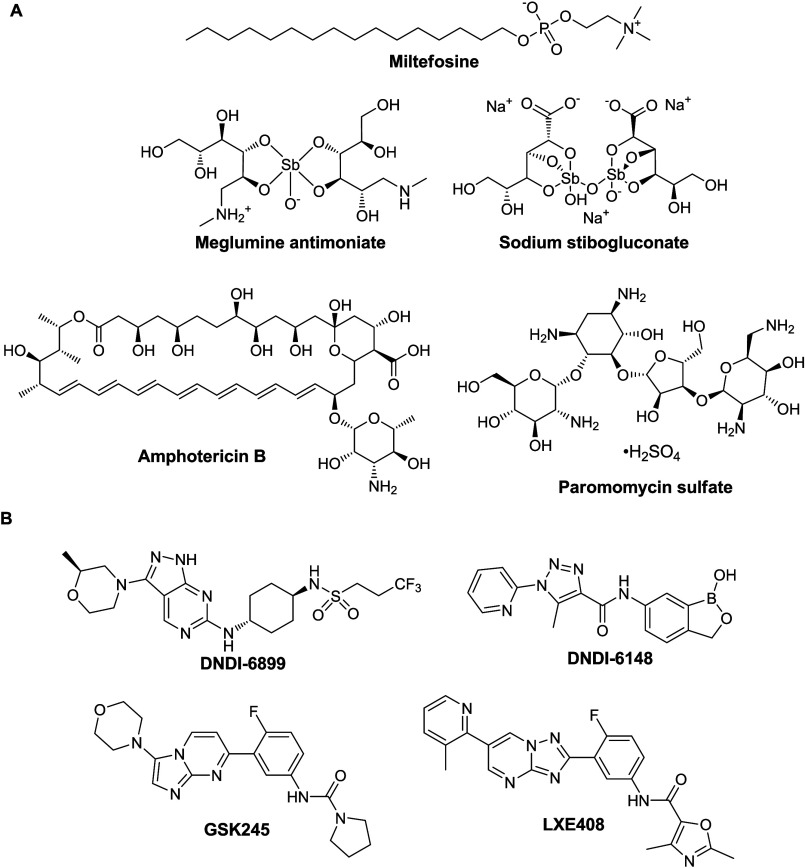
(A) Structures of the five drugs used for treatment of leishmaniasis. (B) Structures of the four drug candidates reported to be in clinical development in 2023.^47^

Leishmaniasis manifests in different forms causing symptoms ranging from benign and localized skin ulcers to systemic disease, which is fatal if left untreated.^19^ Current treatments for visceral leishmaniasis, the most severe form of leishmaniasis, depend on five drugs used either alone or in combination tailored to specific populations and regions (Figure 8A).^46^ Only miltefosine can be dosed orally, while meglumine antimoniate, sodium stibogluconate, amphotericin B and paromomycin sulfate all require lengthy and painful intramuscular or intravenous administration. Moreover, all of the five drugs are associated with significant side effects. Little understanding of the mechanism of action exists for the current drugs. Notably, all visceral leishmaniasis drug candidates currently undergoing preclinical and clinical evaluation have been discovered and optimized using phenotypic assays without knowledge of their molecular target.^47^ However, extensive mode of action studies based on the generation of resistant mutants have been successful in identifying the protein target for four compounds currently in clinical development for treatment of visceral leishmaniasis (Figure 8B).^47^ DNDI-6899 and DNDI-6148 inhibit cdc2-related kinase 12 (CRK12) and cleavage and polyadenylation specificity factor 3 (CPFS3), respectively, while GSK245 and LXE408 are proteasome inhibitors.

Macrocyclic drugs are often large; recent examples of macrocycles targeting protein–protein interactions (PPIs) that have been disclosed to be clinical studies include the peptide LUNA18^48^ (MW 1438 Da) and the nonpeptidic RMC-7977^49^ (MW 865 Da), both of which inhibit KRAS oncoproteins, as well as the polymacrocyclic peptide MK-0616^50^ (MW 1616 Da), which binds to the atherosclerosis target PCSK9. These large compounds reside in a vast chemical space, which currently can only be effectively screened using RNA display libraries of macrocyclic peptides to compensate for low hit rates.^51^ For example, only 10 hits were obtained from an RNA based library composed of 10^14^ different macrocyclic peptides in the discovery of LUNA 18. Chemical synthesis and screening of libraries of macrocycles that are just “structurally diverse” then appears futile. However, considering that most macrocyclic drugs originate from natural products, screening of macrocyclic libraries designed inspired by natural products or natural product-derived fragments may mitigate low hit rates.^9−12^ Using an alternative approach we mined the dictionary of natural products for macrocycles from which side chains were trimmed to provide cores, which can be seen as a macrocyclic equivalent to fragments.^13^ As reported herein exploration of one of these macrocyclic cores, which originated from the natural product hymenocardine, provided potent inhibitors of *L. infantum*. Previously, docking of the set of macrocyclic cores led to the discovery of a weak inhibitor of the Keap1–Nrf2 protein–protein interaction,^13^ that was subsequently optimized to double digit nM potency.^14^ Our work, and approaches reported by other groups, underscores the fact that the structural diversity and biological activity of natural products make them a rich source of drugs and leads for drug discovery.^52^

Our discovery of very potent, natural-product-derived inhibitors of *L. infantum* that have a good permeability across MDCK-MDR1 cell monolayers may pave the way for the development of a novel oral drug against leishmaniasis. The series will require further optimization to improve solubility and metabolic stability. Increased solubility, with maintained or improved potency, is required to embark on mode of action studies based on the generation of resistant mutants. To this end, optimization will first focus on the reduction of π–π interactions in the solid state, guided by crystal structure predictions, and on lowering of the lipophilicity of the series.

## Experimental Section

### General Synthetic and Analytical Methods

Reagents were purchased from BLDpharm, Sigma-Aldrich, Fluorochem, and VWR International. All organic solvents (99.9%) were purchased from VWR International. All nonaqueous reactions were performed in oven-dried glassware under inert (nitrogen) atmosphere. Solvents were concentrated *in vacuo* using a Heidolph Hei-VAP rotary evaporator system. MilliporeSigma thin-layer chromatography (TLC) silica gel plates from VWR were used for monitoring reactions and in the purification of compounds. TLCs were analyzed under UV light (254 nm) in a Scan Kemi TLC–UV-lamp SK-112005 4W. Silica gel (Chameleon, particle size 0.015–0.04 mm) was used for flash-column chromatography purification. Preparative reversed-phase high-performance liquid chromatography (HPLC) was performed on a Kromasil C8 column (250 mm × 21.2 mm, 5 μm) using a Gilson HPLC equipped with a Gilson 322 pump, UV/Visible-156 detector and 202 collector using acetonitrile–water gradients as eluents with a flow rate of 15 mL/min and detection at 210 or 254 nm.

^1^H, ^13^C, COSY, HSQC, and HMBC NMR spectra were recorded at 298 K on an Agilent Technologies 400 MR spectrometer at 400 or 100 MHz (^13^C), or on an OXFORD AS500 spectrometer at 500 or 125 MHz (^13^C), or a Bruker spectrometer at 600 or 150 MHz (^13^C). The residual peak of the deuterated solvent was used as internal standard: CDCl_3_ (δ_H_ 7.26 ppm, δ_C_ 77.0 ppm); CD_3_CN (δ_H_ 1.94 ppm, δ_C_ 118.26 ppm); DMSO-*d*_6_ (δ_H_ 2.50 ppm, δ_C_ 39.5 ppm). High-resolution mass spectrometry (HRMS) for compounds were recorded in electrospray ionization (ESI) mode on an LCT Premier spectrometer connected to a Waters acquity UPLC I-class with acetonitrile–water used as mobile phase (1:1, with a flow rate of 0.25 mL/min). Liquid chromatography–mass spectrometry (LC-MS) spectra were recorded using an Agilent InfinityLab LC/MSD iQ Single Quadrupole system having a C18 Atlantis T3 column (5 μm, 3.0 mm × 50 mm), eluted with acetonitrile–water (95:5, isocratic conditions) and a flow rate of 0.60 mL/min. Electron spray ionization (ESI) was used and the results (chromatograms and spectra) were analyzed in an OpenLab CDS Software Platform. Compounds **1**–**12** are >95% pure as determined by reversed-phase HPLC.

#### *tert*-Butyl ((8*S*,11*S*)-3^2^-Acetamido-8-benzyl-4-,7,10-trioxo-2-oxa-6,9-diaza-1,3(1,4)-dibenzenacyclododecaphane-11-yl)picolinamide (**2**)

Compound **45** (0.25 g, 0.412 mmol) was dissolved in EtOAc (9 mL) and IBX (1.15 g, 4.12 mmol, 10 equiv) added. The reaction mixture was refluxed (85 °C) under inert atmosphere for 2 h when LC-MS analysis showed complete consumption of starting material. IBX was removed from the reaction mixture by centrifugation and filtration. The reaction mixture was concentrated and purified on a silica gel column using 50 to 100% EtOAc in *n*-hexane followed by preparative reversed-phase HPLC using a gradient from 20 to 80% of acetonitrile in water containing 0.1% formic acid to afford compound **2** as white powder (51 mg, 21%). HRMS: *m*/*z* calculated 601.2618, found 601.2660 [M + H]^+^. ^1^H NMR (400 MHz, CDCl_3_) δ 8.45 (s, 1H), 8.17 (s, 1H), 7.35–7.22 (m, 10H), 7.10 (m, 2H), 6.95 (d, *J* = 8.4 Hz, 1H), 6.60 (d, *J* = 8.9 Hz, 1H), 6.43 (dd, *J* = 37.1, 7.7 Hz, 2H), 6.32 (m, 1H), 5.53 (d, *J* = 9.9 Hz, 1H), 5.11 (m, 2H), 3.89 (m, 2H), 3.05 (m, 5H), 2.69 (t, *J* = 11.3 Hz, 2H), 2.28 (d, *J* = 2.7 Hz, 3H), 1.49 (s, 9H), 1.26 (s, 1H). ^13^C NMR (100 MHz, CDCl_3_) δ 199.56, 170.31, 169.11, 168.89, 160.29, 155.34, 154.12, 136.55, 134.40, 132.49, 131.45, 131.25, 130.32, 129.68, 129.06, 127.69, 126.33, 122.52, 122.28, 120.31, 80.63, 58.51, 56.33, 47.89, 40.05, 38.55, 30.13, 28.80, 25.19.

#### *tert*-Butyl ((8*S*,11*S*)-8-Benzyl-4,7,10-trioxo-3^2^-(picolinamido)-2-oxa-6,9-diaza-1,3(1,4)- dibenzenacyclododecaphane-11-yl)carbamate (**3**)

Compound **41** (0.30 g, 0.45 mmol, 1 equiv) was dissolved in EtOAc (10 mL) and IBX (1.27 g, 4.51 mmol, 10 equiv) was added to the solution. The reaction mixture was refluxed (85 °C) under inert atmosphere for 2 h when LC-MS analysis showed complete consumption of starting material. IBX was removed from the reaction mixture by centrifugation and filtration. The reaction mixture was concentrated under reduced pressure and purified by preparative reversed-phase HPLC using a gradient from 20 to 80% of acetonitrile in water containing 0.1% formic acid to afford compound **3** as a white powder (215 mg, 72%). HRMS: *m*/*z* calculated 664.2727, found 664.2716. ^1^H NMR (400 MHz, CDCl_3_) δ 10.89 (s, 1H), 8.71 (s, 1H), 8.63 (d, *J* = 4.7 Hz, 1H), 8.26 (d, *J* = 7.8 Hz, 1H), 7.92 (m, 1H), 7.48 (m, 1H), 7.35 (m, 2H), 7.24 (m, 5H), 7.08 (d, *J* = 6.9 Hz, 2H), 6.89 (d, *J* = 8.5 Hz, 1H), 6.71 (d, *J* = 8.7 Hz, 1H), 6.53 (d, *J* = 8.4 Hz, 1H), 6.28 (d, *J* = 6.8 Hz, 1H), 5.30 (d, *J* = 9.9 Hz, 1H), 5.07 (m, 2H), 3.96–3.83 (m, 2H), 3.18–3.08 (m, 2H), 2.98–2.88 (m, 2H), 2.67–2.60 (t, *J* = 11.4 Hz, 1H), 2.01 (d, *J* = 14.1, 2.7 Hz, 1H), 1.46 (s, 9H), 1.25 (s, 1H). ^13^C NMR (100 MHz, CDCl_3_) δ 199.28, 170.01, 168.55, 162.41, 160.22, 155.02, 149.58, 148.45, 136.31, 136.31, 133.89, 132.05, 131.45, 130.94, 129.88, 129.35, 128.79, 127.41, 126.83, 125.82, 122.67, 122.36, 122.24, 119.62, 80.33, 58.21, 56.11, 47.66, 39.75, 38.29, 29.83, 28.48.

#### *tert*-Butyl ((*S*)-3-(4-Methoxyphenyl)-1-oxo-1-(((*S*)-1-oxo-1-((2-oxo-2-(3-(picolinamido)phenyl)ethyl)amino)-3-phenylpropan-2-yl)amino)propan-2-yl)carbamate (**4**)

Compound **55** (20 mg, 0.013 mmol, 1 equiv) was dissolved in EtOAc-DMSO (0.5–0.1 mL) and IBX (35.8 mg, 0.13 mmol, 10 equiv) was added at 0 °C. The reaction mixture was refluxed (85 °C) under inert atmosphere for 3 h when LC-MS analysis showed complete consumption of starting material. IBX was removed from the reaction mixture by centrifugation and filtration. The reaction mixture was then concentrated under reduced pressure and purified by preparative reversed-phase HPLC using a gradient from 20 to 80% of acetonitrile in water containing 0.1% formic acid to afford **4** (5.5 mg, 55%) as a white solid. LC-MS: *m*/*z* calculated 680.3040, found 680.5 [M + H]^+^. HRMS: *m*/*z* calculated 680.3640, found 680.3628 [M + H]^+^. ^1^H NMR (500 MHz, CD_3_CN) δ 10.26 (s, 1H), 8.69 (dt, *J* = 4.8, 1.2 Hz, 1H), 8.42 (t, *J* = 1.9 Hz, 1H), 8.24 (dd, *J* = 8.0, 1.2 Hz, 1H), 8.12 (dd, *J* = 8.1, 2.2 Hz, 1H), 8.02 (td, *J* = 7.7, 1.8 Hz, 1H), 7.75 (dt, *J* = 7.8, 1.3 Hz, 1H), 7.61 (ddd, *J* = 7.6, 4.7, 1.3 Hz, 1H), 7.55 (t, *J* = 7.9 Hz, 1H), 7.31–7.27 (m, 1H), 7.25–7.21 (m, 3H), 7.06 (d, *J* = 8.4 Hz, 3H), 7.00 (d, *J* = 8.2 Hz, 1H), 6.81–6.79 (m, 2H), 5.47 (m, 1H), 4.63 (m, 3H), 4.16 (ddd, *J* = 8.9, 7.6, 5.4 Hz, 1H), 3.71 (s, 3H), 3.17 (dd, *J* = 14.0, 5.2 Hz, 1H), 2.98–2.90 (m, 2H), 2.68 (dd, *J* = 14.1, 8.9 Hz, 1H), 1.32 (s, 9H). ^13^C NMR (125 MHz, CD_3_CN) δ 195.20, 172.48, 171.97, 163.62, 159.43, 156.59, 150.50, 149.41, 139.72, 139.05, 138.39, 136.59, 131.28, 130.44, 130.37, 130.21, 130.16, 129.34, 128.00, 127.59, 125.93, 124.46, 123.21, 120.21, 114.65, 80.20, 57.21, 55.76, 55.02, 47.29, 38.35, 37.64, 28.48.

#### *tert*-Butyl ((8*S*,11*S*)-8-Benzyl-4,7,10-trioxo-3^2^-(picolinamido)-2-oxa-6,9-diaza-1,3(1,4)-dibenzenacyclotridecaphane-11-yl)carbamate (**5**)

Compound **44** (11 mg, 0.016 mmol, 1 equiv) was dissolved in EtOAc (0.2 mL) and IBX (45.3 mg, 0.16 mmol, 10 equiv) was added at 0 °C. The reaction mixture was refluxed (85 °C) under inert atmosphere for 3 h when LC-MS analysis showed complete consumption of starting material. IBX was removed from the reaction mixture by centrifugation and filtration. The reaction mixture was then concentrated under reduced pressure and purified by flash column chromatography using 10 to 20% MeOH in EtOAc. The fraction containing the product was further purified by preparative reversed-phase HPLC using a gradient from 20 to 80% of acetonitrile in water containing 0.1% formic acid to afford **5** (9.0 mg, 82%) as a white solid. HRMS: *m*/*z* calculated 678.2883, found 678.2915 [M + H]^+^. ^1^H NMR (600 MHz, CDCl_3_) δ 10.88 (s, 1H), 8.95 (s, 1H), 8.65 (s, 1H), 8.33 (d, *J* = 5.4 Hz, 1H), 7.93 (t, *J* = 7.9 Hz, 1H), 7.50 (s, 1H), 7.41 (d, *J* = 8.7 Hz, 2H), 7.24 (s, 1H), 7.12 (d, *J* = 8.4 Hz, 5H), 7.03 (s, 3H), 6.79 (d, *J* = 8.8 Hz, 1H), 5.38 (d, *J* = 10.3 Hz, 1H), 5.23–5.16 (m, 2H), 4.63 (d, *J* = 9.0 Hz, 1H), 4.03 (s, 2H), 3.49 (s, 2H), 3.34 (d, *J* = 16.0 Hz, 2H), 3.11 (d, *J* = 8.3 Hz, 2H), 3.03–2.94 (m, 3H), 2.76 (t, *J* = 10.7 Hz, 2H), 2.57 (t, *J* = 13.3 Hz, 2H), 2.37 (s, 2H), 1.25 (s, 9H), 0.87 (s, 2H). ^13^C NMR (150 MHz, CDCl_3_) δ 197.10, 171.61, 169.63, 162.48, 157.90, 156.46, 154.63, 149.85, 148.49, 137.98, 137.81, 136.50, 130.75, 130.49, 129.71, 129.25, 128.81, 127.26, 126.78, 126.24, 122.95, 122.74, 120.35, 120.16, 81.05, 56.88, 51.21, 47.50, 47.40, 39.55, 31.04, 30.98, 29.85, 28.49, 22.84, 14.27, 1.17.

#### *tert*-Butyl ((8*S*,11*S*)-8-Benzyl-7,10-dioxo-3^2^-(picolinamido)-2-oxa-6,9-diaza-1,3(1,4)-dibenzenacyclododecaphane-11-yl)carbamate (**6**)

2-Picolinic acid (12.7 mg, 0.10 mmol, 1 equiv) was dissolved in DCM (0.5 mL) and HATU (39.2 mg, 0.10 mmol, 1 equiv) was added in portions. DIPEA (26.8 μL, 0.16 mmol, 1.5 equiv) was then added dropwise to the reaction mixture. Compound **50** (54.8 mg, 0.10 mmol, 1 equiv) was dissolved in DCM (0.5 mL) and DIPEA (26.8 μL, 0.16 mmol, 1.5 equiv) was added. The two mixtures were combined and stirred at room temperature for 40 min, concentrated under reduced pressure and purified on a silica gel column and using 10% MeOH in EtOAc The fraction containing the product was further purified by preparative reversed-phase HPLC using a gradient from 20 to 80% of acetonitrile in water containing 0.1% formic acid to afford compound **6** (45 mg, 67%). HRMS: calculated 672.3395, found 672.3301 [M + Na]^+^. ^1^H NMR (500 MHz, CD_3_CN) δ 10.96 (s, 1H), 8.71 (d, *J* = 4.8 Hz, 1H), 8.37 (s, 1H), 8.27 (dt, *J* = 7.9, 1.1 Hz, 1H), 7.04 (td, *J* = 7.7, 1.7 Hz, 1H), 7.62 (ddd, *J* = 7.6, 4.7, 1.2 Hz, 1H), 7.14–6.95 (m, 11H), 6.78 (s, 1H), 6.15 (d, *J* = 10.4 Hz, 1H), 6.01 (d, *J* = 5.5 Hz, 1H), 5.62 (d, *J* = 8.5 Hz, 1H), 4.06 (s, 1H), 3.56 (m, 1H), 3.07–2.89 (m, 3H), 2.72–2.63 (q, *J* = 13.6 Hz, 1H), 2.56–2.50 (m, 1H), 1.93 (s, 9H), 1.28 (s, 1H). ^13^C NMR (125 MHz, CD_3_CN) δ 170.47, 168.58, 162.51, 160.74, 155.70, 150.63, 149.72, 149.42, 139.02, 137.06, 136.91, 134.54, 131.49, 130.68, 128.71, 127.79, 127.30, 122.92, 122.20, 79.72, 57.26, 53.72, 38.25, 37.55, 35.60, 28.40, 27.60.

#### *tert*-Butyl ((8*S*,11*S*)-8-Methyl-4,7,10-trioxo-3^2^-(picolinamido)-2-oxa-6,9-diaza-1,3(1,4)-dibenzenacyclododecaphane-11-yl)carbamate (**7**)

Compound **39** (10 mg, 0.017 mmol, 1 equiv) was dissolved in EtOAc-DMSO (0.2–0.05 mL) and Dess–Martin periodinane (DMP, 74.2 mg, 0.17 mmol, 10 equiv) was added at room temperature. The reaction mixture was stirred at room temperature for 3 h when LC-MS analysis showed complete consumption of starting material. DMP was removed from the reaction mixture by filtration and the reaction mixture was concentrated under reduced pressure and purified by preparative reversed-phase HPLC using a gradient from 20 to 80% of acetonitrile in water containing 0.1% formic acid to afford **7** (5.0 mg, 50%) as a white solid. HRMS: calculated 610.2714, found 610.2770 [M + Na]^+^. ^1^H NMR (500 MHz, CD_3_CN) δ 10.97 (s, 1H), 8.77–8.70 (m, 2H), 8.33–8.27 (m, 1H), 8.07 (tt, *J* = 7.7, 1.4 Hz, 1H), 7.65 (ddd, *J* = 7.5, 4.7, 1.3 Hz, 1H), 7.41–7.34 (m, 2H), 7.29 (d, *J* = 7.1 Hz, 1H), 6.92 (d, *J* = 8.4 Hz, 1H), 6.86 (d, *J* = 8.7 Hz, 1H), 6.79 (dd, *J* = 9.2, 3.1 Hz, 1H), 6.71 (s, 1H), 6.26 (d, *J* = 6.1 Hz, 1H), 5.63 (d, *J* = 8.2 Hz, 1H), 4.93 (s, 1H), 3.88 (q, *J* = 8.1 Hz, 1H), 3.60 (q, *J* = 9.3 Hz, 2H), 2.87 (d, *J* = 7.4 Hz, 2H), 2.15 (s, 2H), 2.00–1.95 (m, 3H), 1.43 (s, 9H), 1.19 (s, 1H), 1.11 (d, *J* = 6.6 Hz, 2H). ^13^C NMR (125 MHz, CD_3_CN) δ 200.86, 171.49, 171.45, 170.78, 163.11, 160.77, 155.94, 154.50, 150.41, 149.55, 139.18, 135.38, 132.61, 131.63, 131.33, 128.10, 126.44, 123.09, 122.72, 122.49, 119.29, 79.91, 58.48, 50.17, 50.07, 48.60, 37.95, 28.42, 19.45.

#### *tert*-Butyl ((8*R*,11*S*)-8-Benzyl-4,7,10-trioxo-3^2^-(picolinamido)-2-oxa-6,9-diaza-1,3(1,4)-dibenzenacyclododecaphane-11-yl)carbamate (**8**)

Compound **42** (20 mg, 0.03 mmol, 1 equiv) was dissolved in EtOAc-DMSO (0.5–0.1 mL) and IBX (84.1 mg, 0.30 mmol, 10 equiv) was added at 0 °C. The reaction mixture was refluxed (85 °C) under inert atmosphere for 3 h when LC-MS analysis showed complete consumption of starting material. IBX was removed from the reaction mixture by centrifugation and filtration. The reaction mixture was then concentrated under reduced pressure and purified by preparative reversed-phase HPLC using a gradient from 20 to 80% of acetonitrile in water containing 0.1% formic acid to afford **8** (10 mg, 50%) as a white solid. HRMS: calculated 664.3227, found 664.3295 [M + H]^+^. ^1^H NMR (500 MHz, CD_3_CN) δ 10.84 (s, 1H), 8.67 (q, *J* = 1.9 Hz, 2H), 8.24 (dq, *J* = 7.8, 1.1 Hz, 1H), 8.02 (tt, *J* = 7.8, 1.4 Hz, 1H), 7.61 (ddt, *J* = 7.5, 4.7, 1.3 Hz, 1H), 7.32–7.19 (m, 2H), 7.17 (q, *J* = 8.0 Hz, 4H), 6.99 (d, *J* = 7.0 Hz, 2H), 6.77 (d, *J* = 8.7 Hz, 2H), 6.54 (m, 2H), 6.49 (d, *J* = 6.3 Hz, 1H), 5.14 (s, 1H), 5.02–4.93 (m, 1H), 4.45–4.42 (m, 1H), 3.80 (q, *J* = 6.0 Hz, 1H), 3.38 (dd, *J* = 13.5, 4.2 Hz, 1H), 3.28 (d, *J* = 15.5 Hz, 1H), 2.83 (m, 2H), 2.68 (dd, *J* = 13.5, 3.9 Hz, 1H), 1.44 (s, 9H), 1.31 (s, 1H). ^13^C NMR (125 MHz, CD_3_CN) δ 200.68, 170.34, 169.23, 163.13, 161.25, 154.79, 150.33, 149.51, 139.17, 139.814, 137.19, 134.04, 133.19, 132.67, 132.60, 131.74, 130.44, 130.32, 130.23, 130.28, 129.39, 129.26, 128.08, 127.84, 127.48, 126.74, 123.17, 123.07, 122.34, 122.20, 119.38, 80.71, 56.73, 55.52, 48.20, 39.34, 37.06, 28.50.

#### *tert*-Butyl ((8*S*,11*S*)-4,7,10-Trioxo-8-phenyl-3^2^-(picolinamido)-2-oxa-6,9-diaza-1,3(1,4)-dibenzenacyclododecaphane-11-yl)carbamate (**9**)

Compound **43** (10 mg, 0.015 mmol, 1 equiv) was dissolved in EtOAc-DMSO (0.2–0.05 mL) and Dess–Martin periodinane (DMP, 67.1 mg, 0.15 mmol, 10 equiv) was added at 0 °C. The reaction mixture was stirred at room temperature for 3 h when LC-MS analysis showed complete consumption of starting material. DMP was removed from the reaction mixture by filtration and the reaction mixture was concentrated under reduced pressure and purified by preparative reversed-phase HPLC using a gradient from 20 to 80% of acetonitrile in water containing 0.1% formic acid to afford **9** (5.0 mg, 50%) as a white solid. HRMS: calculated 650.3170, found 650.3129 [M + H]^+^. ^1^H NMR (500 MHz, CD_3_CN) δ 10.96 (s, 1H), 8.76 (d, *J* = 4.7 Hz, 1H), 8.71 (d, *J* = 7.8 Hz, 1H), 8.30 (m,1H), 8.06 (t, *J* = 7.6 Hz, 1H), 7.63 (dd, *J* = 7.7, 5.0 Hz, 1H), 7.43 (m, 1H), 7.36 (m, 1H), 7.23 (m, 5H), 7.00 (d, *J* = 8.6 Hz, 1H), 6.90–6.85 (m, 3H), 6.67 (d, *J* = 6.4 Hz, 1H), 5.63 (m, 1H), 4.90 (m, 1H), 4.54 (dt, *J* = 9.3, 4.4 Hz, 1H), 3.95 (q, *J* = 6.0 Hz, 1H), 3.42 (dd, *J* = 13.7, 4.2 Hz, 1H), 2.81 (dd, *J* = 13.7, 4.0 Hz, 2H), 1.30 (s, 9H). ^13^C NMR (125 MHz, CD_3_CN) δ 200.75, 170.80, 169.08, 163.26, 160.79, 156.07, 154.92, 154.79, 150.47, 149.64, 139.96, 139.26, 135.56, 132.84, 132.42, 131.67, 131.41, 129.47, 128.72, 128.20, 127.23, 126.50, 123.40, 123.18, 122.82, 122.72, 37.55, 28.53.

#### *tert*-Butyl ((8*S*,11*S*)-8-Isobutyl-4,7,10-trioxo-3^2^-(picolinamido)-2-oxa-6,9-diaza-1,3(1,4)-dibenzenacyclododecaphane-11-yl)carbamate (**10**)

Compound **40** (10 mg, 0.016 mmol, 1 equiv) was dissolved in EtOAc-DMSO (0.5–0.1 mL) and IBX (74.2 mg, 0.17 mmol, 10 equiv) was added at room temperature. The reaction mixture was refluxed (85 °C) under inert atmosphere for 3 h when LC-MS analysis showed complete consumption of starting material. IBX was removed from the reaction mixture by centrifugation and filtration. The reaction mixture was then concentrated under reduced pressure and purified by preparative reversed-phase HPLC using a gradient from 20 to 80% of acetonitrile in water containing 0.1% formic acid to afford **10** (4.5 mg, 45%) as a white solid. LC-MS *m*/*z* calculated 630.2883, found 630.4 [M + H]^+^. HRMS: 630.3415 [M + H]^+^, 652.3241 [M + Na]^+^. ^1^H NMR (500 MHz, CD_3_CN) δ 10.94 (s, 1H), 8.75 (s, 1H), 8.70 (m, 1H), 8.28 (dd, *J* = 7.7, 2.3 Hz, 1H), 8.05 (td, *J* = 7.9, 2.0 Hz, 1H), 7.64–7.61 (m, 1H), 7.38 (d, *J* = 8.8 Hz, 2H), 7.35–7.30 (m, 1H), 6.92 (m, 1H), 6.81–6.78 (m, 2H), 6.64 (s, 1H), 6.20 (d, *J* = 7.8 Hz, 1H), 5.68 (d, *J* = 8.4 Hz, 1H), 5.04–4.99 (m, 1H), 3.84–3.76 (m, 2H), 3.50 (dd, *J* = 15.9, 3.0 Hz, 1H), 2.92 (t, *J* = 12.2 Hz, 1H), 2.79 (d, *J* = 11.9 Hz, 1H), 1.47–1.27 (m, 11H), 0.80 (t, *J* = 6.0 Hz, 6H). ^13^C NMR (125 MHz, CD_3_CN) δ 200.39, 171.05, 170.40, 163.19, 160.80, 156.17, 154.86, 150.50, 149.63, 149.61, 139.23, 135.80, 133.30, 132.20, 131.65, 131.23, 128.15, 126.73, 123.15, 122.81, 122.49, 119.40, 80.05, 58.66, 52.84, 48.43, 44.27, 37.24, 28.57, 25.14, 23.25, 22.83.

#### *tert*-Butyl ((8*S*,11*S*)-11-Acetamido-8-benzyl-4,7,10-trioxo-2-oxa-6,9-diaza-1,3(1,4)-dibenzenacyclododecaphane-3^2^-yl)picolinamide (**11**)

Compound **3** (0.75 g, 1.31 mmol) was taken up in DCM (10 mL) and TFA (2.0 mL, 26.1 mmol, 19.9 equiv) was added dropwise at 0 °C. The reaction temperature was raised to 25 °C and maintained until completion, confirmed by TLC (3.5 h). The reaction mixture was then concentrated under reduced pressure and purified by preparative reversed-phase HPLC using a gradient from 20 to 80% of acetonitrile in water (containing 0.1% formic acid) to afford Boc deprotected compound **3** (200 mg, 0.355 mmol). This material was dissolved in DCM (5 mL) and TEA (0.197 mL, 1.42 mmol, 1.1 equiv) was added at 0 °C followed by dropwise addition of AcCl (0.05 mL, 0.7 mmol 0.5 equiv). The reaction temperature was raised to 25 °C and maintained until completion, confirmed by TLC (∼6 h). The reaction mixture was then concentrated under reduced pressure and purified by preparative reversed-phase HPLC using a gradient from 20 to 80% of acetonitrile in water containing 0.1% formic acid to afford **11** (400 mg, 58%). HRMS: calculated 606.2823, found 606.2889 [M + H]^+^. ^1^H NMR (400 MHz, CDCl_3_) δ 10.88 (s, 1H), 8.72 (d, *J* = 2.2 Hz, 1H), 8.65 (d, *J* = 3.9 Hz, 1H), 8.29 (d, *J* = 7.8 Hz, 1H), 7.97–7.89 (m, 1H), 7.53–7.46 (m, 1H), 7.35 (dd, *J* = 8.6, 2.2 Hz, 1H), 7.26–7.19 (m, 3H), 7.08 (d, *J* = 6.1 Hz, 2H), 6.87 (d, *J* = 7.9 Hz, 1H), 6.76 (d, *J* = 8.6 Hz, 1H), 6.51 (d, *J* = 7.7 Hz, 1H), 6.40 (d, *J* = 7.1 Hz, 1H), 6.08 (d, *J* = 8.9 Hz, 1H), 5.23 (dd, *J* = 10.0, 3.0 Hz, 1H), 5.05 (dd, *J* = 15.5, 10.1 Hz, 1H), 4.34–4.24 (m, 1H), 3.89–3.79 (m, 1H), 3.48 (s, 2H), 3.20–3.08 (m, 2H), 3.01–2.86 (m, 2H), 2.62 (dd, *J* = 13.1, 9.6 Hz, 1H), 1.68 (s, 3H), 1.07 (s, 1H). ^13^C NMR (100 MHz, CDCl_3_) δ 199.17, 169.82, 169.45, 168.49, 162.55, 160.31, 154.34, 149.58, 148.53, 137.87, 136.29, 133.57, 131.88, 131.53, 130.94, 130.00, 129.34, 128.84, 127.48, 126.91, 125.88, 122.70, 122.41, 122.23, 119.78, 56.71, 56.16, 51.01, 47.69, 39.85, 38.24, 23.39.

#### *tert*-Butyl ((8*S*,11*S*)-8-Benzyl-4,7,10-trioxo-11-pivalamido-2-oxa-6,9-diaza-1,3(1,4)-dibenzenacyclododecaphane-3^2^-yl)picolinamide (**12**)

Compound **3** (0.75 mg, 1.31 mmol, 1 equiv) was dissolved up in DCM (10 mL) and TFA (2 mL, 26.1 mmol, 19.9 equiv) was added dropwise at 0 °C. The reaction temperature was raised to 25 °C and maintained until completion, confirmed by TLC (3 h). The reaction mixture was then concentrated under reduced pressure and purified by preparative reversed-phase HPLC using a gradient from 20 to 80% of acetonitrile in water (containing 0.1% formic acid), affording Boc-deprotected material. The amine intermediate (200 mg, 0.355 mmol) was dissolved in DCM (5 mL) and TEA (0.197 mL, 1.42 mmol, 1.1 equiv) was added to it at 0 °C followed by dropwise addition of pivaloyl chloride (0.086 mL, 0.7 mmol, 0.5 equiv). The reaction temperature was then raised to 25 °C and maintained until completion of the reaction, confirmed by TLC (∼5 h). The reaction mixture was then concentrated under reduced pressure and purified by preparative reversed-phase HPLC using a gradient from 20 to 80% of acetonitrile in water containing 0.1% formic acid to afford **12** (455 mg, 62%). HRMS: calculated 648.3328, found 648.3362 [M + H]^+^. ^1^H NMR (400 MHz, CD_3_CN) δ 10.93 (s, 1H), 8.70 (q, *J* = 3.4 Hz, 2H), 8.27 (dt, *J* = 7.9, 1.4 Hz, 1H), 8.05 (tdd, *J* = 7.7, 2.8, 1.6 Hz, 1H), 7.63 (ddt, *J* = 7.5, 4.8, 1.3 Hz, 1H), 7.37 (s, 1H), 7.30 (dt, *J* = 8.7, 2.5 Hz, 2H), 7.25–7.16 (m, 4H), 6.98 (dt, *J* = 7.1, 2.6 Hz, 3H), 6.80 (dd, *J* = 8.7, 2.3 Hz, 1H), 6.67 (s, 1H), 6.59 (d, *J* = 9.2 Hz, 1H), 6.52 (d, *J* = 8.0 Hz, 1H), 6.33 (d, *J* = 6.4 Hz, 1H), 4.88 (dd, *J* = 16.3, 9.6 Hz, 1H), 4.22 (ddd, *J* = 11.8, 8.1, 3.8 Hz, 1H), 3.80 (td, *J* = 6.7, 4.5 Hz, 1H), 3.33 (dt, *J* = 16.0, 2.8 Hz, 1H), 3.02 (td, *J* = 12.0, 2.4 Hz, 2H), 2.84 (dd, *J* = 5.8, 3.1 Hz, 3H), 1.18 (d, *J* = 2.5 Hz, 9H). ^13^C NMR (125 MHz, CD_3_CN) δ 200.19, 178.16, 170.32, 168.96, 162.73, 160.35, 154.18, 150.04, 149.19, 138.81, 136.95, 135.29, 132.06, 131.18, 130.03, 128.66, 127.73, 127.17, 126.16, 122.78, 122.73, 122.31, 118.98, 117.92, 56.95, 55.42, 47.99, 38.98, 38.79, 37.35, 27.29.

#### (*S*)-2-((*tert*-Butoxycarbonyl)amino)-4-(4-((*tert*-butyldimethylsilyl)oxy)phenyl)butanoic Acid (**15**)

Imidazole (0.69 g, 7.45 mmol, 3 equiv) and TBSCl (1.12 g, 7.45 mmol, 2.2 equiv) were added to a solution of (*S)*-Boc-homotyrosine (1.00 g, 3.39 mmol, 1 equiv) in DCM (36 mL) at 0 °C and the mixture was stirred for 18 h at room temperature. The white precipitate was filtered off and the filtrate was concentrated under reduced pressure to give a light-yellow oil. The oil was taken up in THF (12 mL) and water (24 mL) followed by addition of potassium carbonate (246 mg, 1.78 mmol, 0.5 equiv). The mixture was then stirred at room temperature for 1.5 h. Saturated aqueous ammonium chloride (150 mL) and EtOAc (200 mL) were added to the reaction mixture; the phases were separated, and the aqueous phase was extracted with EtOAc (2 × 200 mL). The combined organic phases were washed with brine (100 mL), dried over anhydrous sodium sulfate and concentrated *in vacuo* to give a colorless oil (850 mg, 60%). A part the oil was further purified by preparative reversed-phase HPLC using a gradient from 20 to 80% of acetonitrile in water containing 0.1% formic acid for analytical characterization. LC-MS: *m*/*z* calculated 432.3, found 432.3 [M + Na]^+^. ^1^H NMR (400 MHz, CD_3_CN) δ 7.59 (d, *J* = 8.5 Hz, 2H), 6.29 (d, *J* = 8.6 Hz, 2H), 6.20 (d, *J* = 8.0 Hz, 1H), 4.58 (m, 1H), 3.12 (p, *J* = 8.6 Hz, 2H), 2.56–2.34 (m, 2H), 1.94 (s, 9H), 1.49 (s, 9H), 0.69 (s, 6H). ^13^C NMR (100 MHz, CD_3_CN) δ 174.63, 156.79, 154.82, 135.96, 130.42, 120.88, 79.98, 53.91, 34.23, 31.64, 28.56, 25.99, 18.80.

#### *tert*-Butyl ((*S*)-1-((2-(4-Fluoro-3-nitrophenyl)-2-hydroxyethyl)amino)-1-oxopropan-2-yl)carbamate (**16**)

Boc-l-Ala-OH (1.20 g, 6.34 mmol, 1 equiv) was dissolved in DCM (30 mL) and HATU (2.41 g, 6.34 mmol, 1 equiv) and DIPEA (1.67 mL, 9.51 mmol, 1.5 equiv) were added. 2-(4-Fluoro-3-nitrophenyl)-2-hydroxyethan-1-aminium (1.50 g, 6.34 mmol, 1 equiv) was dissolved in DCM and DIPEA (1.67 mL, 9.51 mmol, 1.5 equiv) was added, the two mixtures combined and stirred at room temperature for 40 min. The reaction mixture was diluted with DCM (20 mL) and washed with aqueous 1 M HCl (2 × 10 mL), saturated aqueous sodium bicarbonate solution (2 × 10 mL) and brine (2 × 10 mL), dried over anhydrous sodium sulfate, filtered, and concentrated under reduced pressure. The residue was purified on a silica gel column using 50% EtOAc in *n*-hexane to afford **16** (1.80 g, 77%) as a light-yellow powder. A small portion of this material was further purified on preparative reversed-phase HPLC using a gradient from 20 to 80% of acetonitrile in water containing 0.1% formic acid for analytical characterization. LC-MS: *m*/*z* calculated 394.14, found 394.0 [M + Na]^+^. ^1^H NMR (400 MHz, CD_3_CN) δ 8.05 (d, *J* = 7.3 Hz, 1H), 7.71–7.68 (m, 1H), 7.36 (dd, *J* = 11.2, 8.7 Hz, 1H), 6.80 (s, 1H), 5.51 (s, 1H), 4.84 (d, *J* = 5.9 Hz, 1H), 4.34 (s, 1H), 3.93 (p, *J* = 7.2 Hz, 1H), 3.42 (m, 3H), 1.39 (s, 9H), 1.18 (dd, *J* = 7.2, 3.9 Hz, 3H). ^13^C NMR (100 MHz, CD_3_CN) δ 155.67, 153.09, 140.17, 133.73–133.64 (d, *J* = 8.9 Hz), 123.65 (d, *J* = 2.5 Hz), 118.11–117.89 (d, *J* = 21.0 Hz), 71.00, 53.20, 46.24, 40.89, 27.52, 24.47, 22.22, 20.76.

#### *tert*-Butyl ((*S*)-1-((2-(4-Fluoro-3-nitrophenyl)-2-hydroxyethyl)amino)-4-methyl-1-oxopentan-2-yl)carbamate (**17**)

Boc-l-Leu-OH (1.20 g, 5.19 mmol, 1 equiv) was dissolved in DCM (15 mL) and HATU (2.41 g, 6.34 mmol, 1 equiv) and DIPEA (1.66 mL, 9.51 mmol, 1.5 equiv) were added. 2-(4-Fluoro-3-nitrophenyl)-2-hydroxyethan-1-aminium (1.55 g, 6.34 mmol, 1 equiv) was dissolved in DCM and DIPEA (1.66 mL, 9.51 mmol, 1.5 equiv) was added, the two mixtures combined and stirred at room temperature for 40 min. The reaction mixture was diluted with DCM (15 mL) and washed with aqueous 1 M HCl solution (2 × 10 mL), saturated aqueous sodium bicarbonate solution (2 × 10 mL) and brine (2 × 10 mL), dried over anhydrous sodium sulfate, filtered, and concentrated under reduced pressure. The residue was purified on a silica gel column using 50% EtOAc in *n*-hexane to afford **17** (1.80 g, 69%) as a light-yellow powder. A small portion of this material was further purified on reversed-phase HPLC using a gradient from 20 to 80% acetonitrile in water containing 0.1% TFA for analytical characterization. LC-MS: *m*/*z* calculated 437.20, found 437.2 [M + Na]^+^. ^1^H NMR (400 MHz, CD_3_CN) δ 8.05 (dd, *J* = 7.4, 2.2 Hz, 1H), 7.71–7.68 (m, 1H), 7.36 (dd, *J* = 11.2, 8.6 Hz, 1H), 6.85 (s, 1H), 5.46 (s, 1H), 4.85 (p, *J* = 4.8 Hz, 1H), 4.40 (s, 1H), 3.91 (m, 2H), 3.48–3.35 (m, 2H), 2.19–2.09 (m, 2H), 1.56 (t, *J* = 8.0 Hz, 1H), 1.39 (s, 9H), 0.87 (t, *J* = 7.3 Hz, 6H). ^13^C NMR (100 MHz, CD_3_CN) δ 156.63, 154.05, 141.13, 134.65 (d, *J* = 9.0 Hz), 124.59, 119.06, 79.88, 71.96, 54.16, 47.20, 41.85, 28.48, 25.43, 23.18, 21.71.

#### *tert*-Butyl ((*R*)-1-((2-(4-Fluoro-3-nitrophenyl)-2-hydroxyethyl)amino)-1-oxo-3-phenylpropan-2-yl)carbamate (**19**)

Boc-d-Phe-OH (1.35 g, 5.07 mmol, 1 equiv) was dissolved in DCM (28 mL) and HATU (1.93 g, 5.07 mmol, 1 equiv) and DIPEA (1.33 mL, 7.6 mmol, 1.5 equiv) were added. 2-(4-Fluoro-3-nitrophenyl)-2-hydroxyethan-1-aminium (1.20 g, 5.07 mmol, 1 equiv) was dissolved in DCM and DIPEA (1.33 mL, 7.6 mmol, 1.5 equiv) was added, the two mixtures combined and stirred at room temperature for 40 min. The reaction mixture was diluted with DCM (20 mL) and washed with aqueous 1 M HCl solution (2 × 10 mL), saturated aqueous sodium bicarbonate solution (2 × 10 mL) and brine (2 × 10 mL), dried over anhydrous sodium sulfate, filtered, and concentrated under reduced pressure. The residue was purified on a silica gel column using 50% EtOAc in *n*-hexane to afford compound **19** (1.70 g, 75%) as a light-yellow powder. A small portion of this material was further purified on preparative reversed-phase HPLC using a gradient from 20 to 80% of acetonitrile in water containing 0.1% formic acid for analytical characterization. LC-MS: *m*/*z* calculated 470.17, found 470.1 [M + Na]^+^. ^1^H NMR (500 MHz, DMSO-*d*_6_) δ 8.15 (ddd, *J* = 13.5, 7.3, 2.2 Hz, 1H), 8.07–7.99 (m, 1H), 7.80 (dddd, *J* = 27.9, 8.9, 4.3, 2.3 Hz, 1H), 7.59 (ddd, *J* = 11.3, 8.6, 5.7 Hz, 1H), 7.34–7.20 (m, 4H), 6.93 (dd, *J* = 20.0, 8.7 Hz, 1H), 5.92 (dd, *J* = 7.1, 4.5 Hz, 1H), 4.81 (q, *J* = 5.6 Hz, 1H), 4.19–4.08 (m, 1H), 3.34–3.27 (m, 2H), 2.87 (dt, *J* = 13.8, 4.5 Hz, 1H), 2.66 (dt, *J* = 13.7, 10.9 Hz, 1H), 1.33 (d, *J* = 6.1 Hz, 9H), 1.23 (s, 1H). ^13^C NMR (125 MHz, DMSO-*d*_6_) δ 172.42 (d, *J* = 11.9 Hz), 156.93–151.84 (m), 141.48 (dd, *J* = 7.8, 3.7 Hz), 139.95–135.70 (m), 134.60 (d, *J* = 8.7 Hz), 129.73, 129.58, 128.42, 126.59, 124.03 (d, *J* = 10.4 Hz), 118.45 (d, *J* = 21.0 Hz), 78.38 (d, *J* = 7.6 Hz), 70.20 (d, *J* = 4.9 Hz), 56.27, 56.06, 46.42, 46.30, 38.01, 37.84, 28.56, 28.21.

#### *tert*-Butyl ((*S*)-2-((2-(4-Fluoro-3-nitrophenyl)-2-hydroxyethyl)amino)-2-oxo-1-phenylethyl)carbamate (**20**)

(*S*)-2-((*tert*-Butoxycarbonyl)amino)-2-phenylacetic acid (531 mg, 2.11 mmol, 1 equiv) was dissolved in DCM (10 mL) and HATU (1.04 g, 2.75 mmol, 1 equiv) and DIPEA (0.813 mL, 4.67 mmol, 1.5 equiv) were added. 2-(4-Fluoro-3-nitrophenyl)-2-hydroxyethan-1-aminium (0.50 g, 2.11 mmol, 1 equiv) was dissolved in DCM (5 mL) and DIPEA (0.813 mL, 4.67 mmol, 1.5 equiv) was added, the two mixtures combined and stirred at room temperature for 40 min. The reaction mixture was diluted with DCM (20 mL) and washed with aqueous 1 M HCl solution (2 × 10 mL), saturated aqueous sodium bicarbonate solution (2 × 10 mL) and brine (2 × 10 mL), dried over anhydrous sodium sulfate, filtered, and concentrated under reduced pressure. The residue was purified on a silica gel column (50% EtOAc in hexanes) to afford **20** (595 mg, 50%) as a light-yellow powder. A small portion of this material was further purified on reversed-phase HPLC using a gradient from 20 to 80% acetonitrile in water containing 0.1% formic acid for analytical characterization. LC-MS: *m*/*z* calculated 457.16, found 457.2 [M + Na]^+^.

#### *tert*-Butyl ((*S*)-3-(4-((*tert*-Butyldimethylsilyl)oxy)phenyl)-1-(((*S*)-1-((2-(4-fluoro-3-nitrophenyl)-2-hydroxyethyl)amino)-1-oxopropan-2-yl)amino)-1-oxopropan-2-yl)carbamate (**21**)

(*S*)-2-((*tert*-Butoxycarbonyl)amino)-3-(4-((*tert*-butyldimethylsilyl)oxy)phenyl)propanoic acid (1.07 g, 2.69 mmol, 1 equiv) was dissolved in DCM (20 mL) and HATU (2.13 g, 2.69 mmol, 1 equiv) and DIPEA (0.75 mL, 4.11 mmol, 1.5 equiv) were added. Then compound **16** (1.00 g, 2.69 mmol, 1 equiv) was dissolved in 4 M HCl in dioxane (4 mL). After stirring for 30 min the solvent was evaporated, DIPEA (0.75 mL, 4.11 mmol, 1.5 equiv) was added and the two solutions were combined at room temperature. After 40 min, when LC-MS showed complete conversion, the reaction mixture was diluted with DCM (20 mL) and washed with aqueous 1 M HCl solution (2 × 10 mL), saturated aqueous sodium bicarbonate solution (2 × 10 mL) and brine (2 × 10 mL), dried over anhydrous sodium sulfate, filtered, and concentrated under reduced pressure. The residue was purified on a silica gel column using 0 to 10% MeOH in EtOAc to afford **21** (1.05 g, 60%) as a light orange powder. A small portion of this material was further purified on reversed-phase HPLC using a gradient from 20 to 80% of acetonitrile in water containing 0.1% formic acid for analytical characterization. LC-MS: *m*/*z* calculated 649.30, found 649.2 [M + H]^+^.

#### *tert*-Butyl ((*S*)-3-(4-((*tert*-Butyldimethylsilyl)oxy)phenyl)-1-(((*S*)-1-((2-(4-fluoro-3-nitrophenyl)-2-hydroxyethyl)amino)-4-methyl-1-oxopentan-2-yl)amino)-1-oxopropan-2-yl)carbamate (**22**)

(*S*)-2-((*tert*-Butoxycarbonyl)amino)-3-(4-((*tert*-butyldimethylsilyl)oxy)phenyl)propanoic acid (1.15 g, 2.91 mmol, 1 equiv) was dissolved in DCM (20 mL) and HATU (1.22 g, 2.91 mmol, 1 equiv) and DIPEA (0.81 mL, 4.66 mmol, 1.5 equiv) were added. Then compound **17** (1.20 g, 2.91 mmol, 1 equiv) was dissolved in 4 M HCl in dioxane (4 mL). After stirring for 30 min the solvent was evaporated, DIPEA (0.75 mL, 4.11 mmol, 1.5 equiv) was added and the two solutions were combined at room temperature. After 40 min, when LC-MS showed complete conversion, the reaction mixture was diluted with DCM (20 mL) and washed with aqueous 1 M HCl solution (2 × 10 mL), saturated aqueous sodium bicarbonate solution (2 × 10 mL) and brine (2 × 10 mL), dried over anhydrous sodium sulfate, filtered, and concentrated under reduced pressure. The residue was purified on a silica gel column using 0 to 10% MeOH in EtOAc to afford **22** (1.05 g, 52%) as a light orange powder. A small portion of this material was further purified on reversed-phase HPLC using a gradient from 20 to 80% acetonitrile in water containing 0.1% formic acid to provide **22** for analytical characterization. LC-MS: *m*/*z* calculated 690.89, found 691.2 [M + H]^+^.

#### *tert*-Butyl ((*S*)-3-(4-((*tert*-Butyldimethylsilyl)oxy)phenyl)-1-(((*R*)-1-((2-(4-fluoro-3-nitrophenyl)-2-hydroxyethyl)amino)-1-oxo-3-phenylpropan-2-yl)amino)-1-oxopropan-2-yl)carbamate (**24**)

(*R*)-2-((*tert*-Butoxycarbonyl)amino)-3-(4-((*tert*-butyldimethylsilyl)oxy)phenyl)propanoic acid (1.50 g, 3.35 mmol, 1 equiv) was dissolved in DCM (20 mL) and HATU (1.28 g, 3.35 mmol, 1 equiv) and DIPEA (0.88 mL, 5.03 mmol, 1.5 equiv) were added. Then compound **19** (1.33 g, 3.35 mmol, 1 equiv) was dissolved in 4 M HCl in dioxane (4 mL). After stirring for 30 min, the solvent was evaporated, DIPEA (0.75 mL, 4.11 mmol, 1.5 equiv) was added and the two solutions were combined at room temperature. After 40 min, when LC-MS showed complete conversion the reaction mixture was diluted with DCM (20 mL) and washed with aqueous 1 M HCl solution (2 × 10 mL), saturated aqueous sodium bicarbonate solution (2 × 10 mL) and brine (2 × 10 mL), dried over anhydrous sodium sulfate, filtered, and concentrated under reduced pressure and purified on a silica gel column using 0 to 10% MeOH in EtOAc to afford **24** (1.95 g, 80%) as a yellow powder. A small portion of this material was further purified on reversed-phase HPLC using a gradient from 20 to 80% acetonitrile in water containing 0.1% formic acid for analytical characterization. LC-MS: *m*/*z* calculated 725.33, found 725.6 [M + H]^+^. ^1^H NMR (500 MHz, DMSO-*d*_6_) δ 8.25–8.17 (m, 2H), 8.11–8.08 (ddd, *J* = 9.7, 4.2, 2.2 Hz, 1H), 7.69 (m, 1H), 7.54–7.50 (m, 1H), 7.23–7.14 (m, 5H), 6.97–6.94 (m, 2H), 6.70–6.63 (m, 3H), 5.87 (dd, *J* = 14.8, 4.5 Hz, 1H), 4.74 (qd, *J* = 5.9, 2.9 Hz, 1H), 4.50 (m, 1H), 4.08 (m, 1H), 3.35 (m, 2H), 2.89–2.86 (m, 1H), 2.64 (m, 1H), 2.35 (ddd, *J* = 17.9, 13.7, 10.1 Hz, 1H), 1.27 (d, *J* = 3.7 Hz, 9H), 0.93 (s, 9H), 0.15 (s, 6H). ^13^C NMR (125 MHz, DMSO-*d*_6_) δ 171.43–171.27 (m), 155.19–154.71 (m), 153.45–153.37 (m), 152.66–152.64 (m), 141.10 (dd, *J* = 5.9, 3.7 Hz), 137.81 (d, *J* = 2.2 Hz), 136.50, 136.44 (dd, *J* = 7.8, 1.9 Hz), 134.27 (dd, *J* = 13.1, 8.7 Hz), 134.16, 130.40 (d, *J* = 4.3 Hz), 129.26, 127.99 (d, *J* = 2.7 Hz), 126.29, 123.46 (t, *J* = 3.4 Hz), 119.19 (d, *J* = 2.9 Hz), 118.05 (dd, *J* = 20.7, 3.6 Hz), 77.92, 69.78, 69.69, 55.63, 55.53, 53.77 (d, *J* = 9.6 Hz), 46.03 (d, *J* = 6.0 Hz), 38.18, 38.11, 37.82, 36.82, 36.77, 28.57, 28.15, 27.78, 25.60, 17.94.

#### *tert*-Butyl ((*S*)-3-(4-((*tert*-Butyldimethylsilyl)oxy)phenyl)-1-(((*S*)-2-((2-(4-fluoro-3-nitrophenyl)-2-hydroxyethyl)amino)-2-oxo-1-phenylethyl)amino)-1-oxopropan-2-yl)carbamate (**25**)

(*S*)-2-((*tert*-Butoxycarbonyl)amino)-3-(4-((*tert*-butyldimethylsilyl)oxy)phenyl)propanoic acid (0.46 g, 1.15 mmol, 1 equiv) was dissolved in DCM (10 mL) and HATU (0.52 g, 1.36 mmol, 1 equiv) and DIPEA (0.476 mL, 2.73 mmol, 1.5 equiv) were added. Then, compound **20** (0.50 g, 1.15 mmol, 1 equiv) was dissolved in 4 M HCl in dioxane (4 mL). After stirring for 30 min, the mixture was concentrated, DIPEA (0.75 mL, 4.11 mmol, 1.5 equiv) was added and the two mixtures combined at room temperature. After 40 min, when LC-MS showed complete conversion the reaction mixture was diluted with DCM (20 mL) and washed with an aqueous 1 M HCl solution (2 × 10 mL), saturated aqueous sodium bicarbonate solution (2 × 10 mL) and brine (2 × 10 mL), dried over anhydrous sodium sulfate, filtered, and concentrated under reduced pressure. The resulting mixture was purified on a silica gel column using 0 to 10% MeOH in EtOAc to afford **25** (415 mg, 51%) as a white powder. A small portion of this material was further purified on reversed-phase HPLC using a gradient from 40 to 95% acetonitrile in water containing 0.1% formic acid for analytical characterization. LC-MS: *m*/*z* calculated 711.38, found 711.5 [M + H]^+^.

#### *tert*-Butyl ((*S*)-4-(4-((*tert*-Butyldimethylsilyl)oxy)phenyl)-1-(((*S*)-1-((2-(4-fluoro-3-nitrophenyl)-2-hydroxyethyl)amino)-1-oxo-3-phenylpropan-2-yl)amino)-1-oxobutan-2-yl)carbamate (**26**)

(*S*)-2-((*tert*-Butoxycarbonyl)amino)-4-(4-((*tert*-butyldimethylsilyl)oxy)phenyl)butanoic acid (1.15 g, 2.81 mmol, 1 equiv) was dissolved in DCM (20 mL) and HATU (1.07 g, 2.81 mmol, 1 equiv) and DIPEA (0.73 mL, 4.21 mmol, 1.5 equiv) were added. Then, compound **15** (1.26 g, 2.81 mmol, 1 equiv) was dissolved in 4 M HCl in dioxane (12 mL). After stirring for 30 min, the mixture was concentrated, DIPEA (0.75 mL, 4.11 mmol, 1.5 equiv) was added and the two mixtures were combined at room temperature. After 40 min, when LC-MS showed complete conversion the reaction mixture was diluted with DCM (20 mL) and washed with aqueous 1 M HCl solution (2 × 10 mL), saturated aqueous sodium bicarbonate solution (2 × 10 mL) and brine (2 × 10 mL), dried over anhydrous sodium sulfate, filtered, and concentrated under reduced pressure. The residue was purified on a silica gel column using 0 to 10% MeOH in EtOAc to afford **27** (957 mg, 46%) as a white powder. A small portion of this material was further purified on reversed-phase HPLC using a gradient from 20 to 80% of acetonitrile in water (0.1% formic acid) for analytical characterization. LC-MS: *m*/*z* calculated 739.5, found 739.3 [M + H]^+^. ^1^H NMR (400 MHz, DMSO-*d*_6_) δ 8.10–8.03 (m, 2H), 7.78–7.50 (m, 2H), 7.49–7.45 (m, 1H), 7.21–7.13 (m, 5H), 7.17–7.08 (m, 2H), 7.00–6.94 (m, 3H), 6.74–6.72 (m, 2H), 5.83 (dd, *J* = 11.3, 4.5 Hz, 1H), 4.71–4.68 (m, 1H), 4.48 (ddd, *J* = 17.6, 13.4, 9.0 Hz, 1H), 3.80 (dt, *J* = 13.5, 6.4 Hz, 1H), 3.40 (dt, *J* = 12.7, 6.2 Hz, 1H), 3.31–3.13 (m, 1H), 2.83–2.90 (m, 1H), 2.76–2.63 (m, 1H), 2.36 (t, *J* = 5.2 Hz, 2H), 1.70–1.58 (m, 2H), 1.35 (s, 9H), 1.22 (d, *J* = 6.8 Hz, 1H), 0.83 (s, 9H), 0.18 (s, 6H). ^13^C NMR (100 MHz, DMSO-*d*_6_) δ 171.18, 155.30, 153.05, 152.32, 140.95, 137.61, 134.28, 129.29 (d, *J* = 13.7 Hz), 127.92, 126.18, 123.40, 119.55, 118.09, 117.88 (d, *J* = 20.6 Hz), 78.23, 69.74, 54.30, 53.40, 46.02, 34.07, 30.59, 28.17, 25.58, 17.92.

#### *tert*-Butyl ((8*S*,11*S*)-4-Hydroxy-8-methyl-3^2^-nitro-7,10-dioxo-2-oxa-6,9-diaza-1,3(1,4)-dibenzenacyclododecaphane-11-yl)carbamate (**27**)

Compound **21** (0.50 g, 0.77 mmol, 1 equiv) was dissolved in DMF (100 mL), CsF (2.34 g, 15.41 mmol, 20 equiv) was added in portions and the reaction mixture was stirred at 50 °C. After 3 h the DMF was evaporated under reduced pressure and the residue was purified on a silica gel column using 10 to 20% MeOH in EtOAc to afford **27** (325 mg, 82%) as a pale-orange powder. A part of this was further purified on reversed-phase HPLC using a gradient from 20 to 80% acetonitrile in water containing 0.1% formic acid for analytical characterization. LC-MS: *m*/*z* calculated 515.21, found 515.0 [M + H]^+^. ^1^H NMR (500 MHz, DMSO-*d*_6_) δ 8.43 (q, *J* = 5.9 Hz, 1H), 8.00 (d, *J* = 6.2 Hz, 1H), 7.63 (q, *J* = 3.5 Hz, 1H), 7.32–7.29 (m, 2H), 7.164–7.05 (m, 2H), 6.98–6.95 (m, 2H), 5.89 (s, 1H), 4.79 (d, *J* = 5.4 Hz, 1H), 4.70 (m, 1H), 4.08 (td, *J* = 9.5, 4.4 Hz, 2H), 3.75 (q, *J* = 7.0 Hz, 1H), 3.55 (td, *J* = 14.9, 8.3 Hz, 2H), 3.27–3.19 (m, 3H), 3.02 (dd, *J* = 13.8, 4.4 Hz, 2H), 2.82 (dd, *J* = 13.9, 10.4 Hz, 1H), 1.33 (s, 9H), 1.26 (dd, *J* = 13.8, 6.9 Hz, 2H), 1.21–1.12 (m, 2H). ^13^C NMR (125 MHz, DMSO-*d*_6_) δ 173.55, 169.86, 155.49, 154.34, 154.25, 148.53, 148.39, 140.66, 140.62 139.53, 139.46, 134.38, 134.32, 132.61, 130.95, 123.08, 123.03, 120.54, 118.29, 118.17, 114.94, 78.08, 69.83, 69.56, 55.22, 48.27, 48.18, 45.96, 45.79, 35.77, 28.18, 17.50.

#### *tert*-Butyl ((8*S*,11*S*)-4-Hydroxy-8-isobutyl-3^2^-nitro-7,10-dioxo-2-oxa-6,9-diaza-1,3(1,4)-dibenzenacyclododecaphane-11-yl)carbamate (**28**)

Compound **22** (0.50 g, 0.72 mmol, 1 equiv) was dissolved in DMF (100 mL), CsF (2.20 g, 14.5 mmol, 20 equiv) was added in portions and the reaction mixture was stirred at 50 °C. After 3 h the DMF was evaporated under reduced pressure and the residue was purified on a silica gel column using 10 to 20% MeOH in EtOAc t afford **28** (325 mg, 81%) as a pale-orange powder. A part of this was further purified on reversed-phase HPLC using a gradient from 20 to 80% acetonitrile in water containing 0.1% formic acid for analytical characterization. LC-MS: *m*/*z* calculated 578.24, found 578.1 [M + Na]^+^. ^1^H NMR (500 MHz, DMSO-*d*_6_) δ 8.31 (dt, *J* = 20.7, 5.9 Hz, 1H), 7.98 (d, *J* = 2.1 Hz, 1H), 7.62 (dd, *J* = 8.6, 2.2 Hz, 1H), 7.28–7.26 (m, 2H), 7.04 (d, *J* = 8.6 Hz, 1H), 6.94–6.90 (dd, *J* = 8.3, 6.0 Hz, 3H), 5.84 (s, 1H), 4.78 (t, *J* = 5.3 Hz, 1H), 4.64 (t, *J* = 6.0 Hz, 1H), 4.01 (td, *J* = 8.8, 4.2 Hz, 1H), 3.55–3.50 (m, 2H), 3.22 (dd, *J* = 11.7, 6.6 Hz, 1H), 2.95 (dd, *J* = 13.7, 4.5 Hz, 1H), 2.80 (dd, *J* = 13.7, 9.7 Hz, 1H), 1.48–1.45 (m, 1H), 1.35 (s, 9H), 1.32–1.21 (m, 4H), 0.85–0.77 (m, 6H). ^13^C NMR (125 MHz, DMSO-*d*_6_) δ 173.35, 155.33, 154.23, 154.13, 148.55, 148.47, 140.54, 140.51, 139.60, 139.52, 134.56, 134.53, 132.67, 130.99, 123.13, 123.10, 120.46, 120.41, 118.21, 118.12, 77.93, 69.92, 69.62, 55.35, 51.71, 45.77, 45.72, 42.27, 42.08, 35.92, 29.06, 28.18, 27.90, 23.79, 23.75, 22.85, 22.74, 21.82, 21.79.

#### *tert*-Butyl ((8*R*,11*S*)-8-Benzyl-4-hydroxy-3^2^-nitro-7,10-dioxo-2-oxa-6,9-diaza-1,3(1,4)-dibenzenacyclododecaphane-11-yl)carbamate (**30**)

Compound **24** (0.53 g, 0.72 mmol, 1 equiv) was dissolved in DMF (100 mL), CsF (2.18 g, 14.34 mmol, 20 equiv) was added in portions and the reaction mixture was stirred at 50 °C. After 3 h the DMF was evaporated under reduced pressure and the residue was purified on a silica gel column using 10 to 20% MeOH in EtOAc to afford **30** (380 mg, 88%) as a pale-orange powder in. A part of this was further purified on reversed-phase HPLC using a gradient from 20 to 80% acetonitrile in water containing 0.1% formic acid for analytical characterization. LC-MS: *m*/*z* calculated 591.24, found 591.4 [M + H]^+^. ^1^H NMR (500 MHz, CD_3_CN) δ 7.98 (s, 1H), 7.30–6.08 (m, 11H), 5.13 (s, 1H), 4.96 (s, 1H), 4.28 (s, 1H), 3.86 (m, 1H), 3.71 (m, 1H), 3.31 (dd, *J* = 13.9, 4.8 Hz, 1H), 3.00 (m, 1H), 2.82–2.70 (m, 2H), 2.69 (dd, *J* = 13.7, 4.2 Hz, 1H), 1.40 (s, 8H), 1.33 (dd, *J* = 12.8, 6.4 Hz, 2H). ^13^C NMR (125 MHz, CD_3_CN) δ 169.80, 169.32, 160.33, 155.82, 152.93, 143.69, 139.80, 137.03, 133.23, 131.45, 130.22, 128.64, 126.99, 125.49, 122.86, 121.82, 117.90, 115.64, 80.18, 70.19, 56.56, 53.58, 45.41, 37.78, 36.94, 28.12.

#### *tert*-Butyl ((8*S*,11*S*)-4-Hydroxy-3^2^-nitro-7,10-dioxo-8-phenyl-2-oxa-6,9-diaza-1,3(1,4)-dibenzenacyclododecaphane-11-yl)carbamate (**31**)

Compound **25** (0.53 g, 0.75 mmol, 1 equiv) was dissolved in DMF (100 mL), CsF (2.26 g, 14.91 mmol, 20 equiv) was added in portions and the reaction mixture was stirred at 50 °C. After 3 h the DMF was evaporated under reduced pressure and the residue was purified on a silica gel column using 10 to 20% MeOH in EtOAc to afford **31** (325 mg, 76%) as a pale-orange powder in. A part of this was further purified on reversed-phase HPLC using a gradient from 20 to 80% acetonitrile in water containing 0.1% formic acid for analytical characterization. LC-MS: *m*/*z* calculated 577.26, found 577.3 [M + H]^+^.

#### *tert*-Butyl ((8*S*,11*S*)-8-Benzyl-4-hydroxy-3^2^-nitro-7,10-dioxo-2-oxa-6,9-diaza-1,3(1,4)-dibenzenacyclotridecaphane-11-yl)carbamate (**32**)

Compound **27** (1.00 g, 1.35 mmol, 1 equiv) was dissolved in DMF (100 mL) and CsF (4.11 g, 27.07 mmol, 20 equiv) was added in portions at 50 °C. After 3 h the DMF was evaporated under reduced pressure and the residue purified on a silica gel column using 10 to 40% MeOH in EtOAc to afford **32** (700 mg, 86%). A part of this was further purified on reversed-phase HPLC using a gradient from 20 to 80% of acetonitrile in water containing 0.1% formic acid for analytical characterization. LC-MS: *m*/*z* calculated 605.26, found 605.2 [M + H]^+^. ^1^H NMR (500 MHz, CD_3_CN) δ 7.79 (s, 1H), 7.34 (dd, *J* = 8.7, 2.3 Hz, 1H), 7.25–7.16 (m, 3H), 7.12 (d, *J* = 6.3 Hz, 2H), 7.10–7.04 (m, 2H), 6.97 (dd, *J* = 8.6, 2.1 Hz, 3H), 6.42–6.37 (m, 2H), 5.51 (d, *J* = 8.7 Hz, 1H), 4.85 (s, 1H), 4.27–4.19 (m, 1H), 3.79–3.70 (s, 1H), 3.26–3.18 (m, 1H), 3.01–2.93 (m, 2H), 2.84 (dt, *J* = 13.4, 4.2 Hz, 1H), 2.74 (dd, *J* = 13.9, 6.7 Hz, 1H), 2.52–2.41 (m, 2H), 2.07 (m, 1H), 1.70–1.62 (m, 2H), 1.45 (s, 9H), 1.44–1.41 (m, 2H), 1.38 (s, 1H). ^13^C NMR (125 MHz, CD_3_CN) δ 171.59, 171.03, 158.59, 157.25, 153.07, 139.75, 139.48, 137.95, 133.12, 131.35, 130.40, 129.04, 127.47, 124.52, 122.72, 122.63, 80.30, 71.10, 54.11, 52.89, 46.40, 39.00, 32.69, 32.09, 28.54.

#### *tert*-Butyl ((8*S*,11*S*)-3^2^-Amino-4-hydroxy-8-methyl-7,10-dioxo-2-oxa-6,9-diaza-1,3(1,4)-dibenzenacyclododecaphane-11-yl)carbamate (**33**)

Compound **27** (0.30 g, 0.58 mmol, 1 equiv) was added to Pd–C (50%, 0.15 g) and MeOH (10 mL) was added under argon. The reaction mixture was then stirred under 1 atm H_2_ in a parr hydrogenator. When LC-MS showed completion of the reaction after 1.5 h, the reaction mixture was filtered through a Celite pad and the solvent was removed under reduced pressure to afford **33** (0.85 g, 60%) as a light-yellow powder. A part of this was further purified on reversed-phase HPLC using a gradient from 20 to 80% acetonitrile in water containing 0.1% formic acid for analytical characterization. LC-MS: *m*/*z* calculated 485.24, found 485.20 [M + H]^+^.

#### *tert*-Butyl ((8*S*,11*S*)-3^2^-Amino-4-hydroxy-8-isobutyl-7,10-dioxo-2-oxa-6,9-diaza-1,3(1,4)-dibenzenacyclododecaphane-11-yl)carbamate (**34**)

Compound **28** (0.32 g, 0.58 mmol, 1 equiv) was added to Pd–C (50%, 0.16 g) and MeOH (10 mL) was added under argon. The reaction mixture was then stirred under 1 atm H_2_ in a parr hydrogenator. When LC-MS showed completion of the reaction after 1.5 h, the reaction mixture was filtered through a Celite pad and the solvent was removed under reduced pressure to afford **34** (156 mg, 52%) as a white powder. A part of this was further purified on reversed-phase HPLC using a gradient from 20 to 80% acetonitrile in water containing 0.1% formic acid for analytical characterization. LC-MS: *m*/*z* calculated 527.28, found 527.1 [M + H]^+^. ^1^H NMR (500 MHz, DMSO-*d*_6_) δ 8.20 (m, 1H), 7.16 (d, *J* = 8.1 Hz, 1H), 6.85 (m, 1H), 6.78 (m, 4H), 6.69 (d, *J* = 8.0 Hz, 2H), 6.49 (dd, *J* = 8.2, 2.4 Hz, 2H), 5.44 (s, 1H), 4.87 (s, 1H), 4.47 (dt, *J* = 14.1, 5.9 Hz, 2H), 3.96 (dt, *J* = 8.7, 4.5 Hz, 2H), 2.96 (dd, *J* = 13.8, 4.6 Hz, 3H), 2.79 (dd, *J* = 13.7, 9.3 Hz, 2H), 2.06 (m, 1H), 1.64 (dq, *J* = 14.1, 6.9 Hz, 3H), 1.46–1.39 (m, 3H), 1.33 (s, 9H), 1.31 (m, 3H), 0.86 (m, 6H). ^13^C NMR (125 MHz, DMSO-*d*_6_) δ 173.30, 156.11, 155.30, 140.84, 140.08, 132.10, 130.39, 119.70, 116.31, 116.26, 114.08, 113.43, 77.87, 71.16, 55.56, 52.14, 46.60, 42.68, 35.82, 28.20, 27.92, 23.91, 23.07, 23.02, 21.84.

#### *tert*-Butyl ((8*S*,11*S*)-3^2^-Amino-8-benzyl-4-hydroxy-7,10-dioxo-2-oxa-6,9-diaza-1,3(1,4)-dibenzenacyclododecaphane-11-yl)carbamate (**35**)

Compound **29** (1.48 g, 2.51 mmol) was added to Pd–C (10%, 1.01 g) and MeOH (13 mL) was added under argon. The reaction mixture was then stirred under 1 atm H_2_ in a parr hydrogenator. When LC-MS showed completion of the reaction after 2 h, the reaction mixture was filtered through a Celite pad and the solvent was removed under reduced pressure to afford **35** (0.85 g, 60%) as a white powder which was used in the next step without further purification. A part of this was further purified on reversed-phase HPLC using a gradient from 20 to 80% acetonitrile in water containing 0.1% formic acid for analytical characterization. LC-MS: *m*/*z* calculated 561.27, found 561.3 [M + H]^+^. ^1^H NMR (500 MHz, DMSO-*d*_6_) δ 7.58 (s, 1H), 7.23–6.91 (m, 10H), 6.72–6.62 (m, 2H), 6.16 (m, 2H), 5.39–5.18 (m, 3H), 5.18 (m, 2H), 4.30–4.17 (m, 2H), 3.50 (m, 1H), 2.96 (dd, *J* = 14.0, 3.9 Hz, 1H), 2.82 (m, 4H), 1.42 (s, 9H). ^13^C NMR (125 MHz, DMSO-*d*_6_) δ 169.61, 166.71, 160.33, 154.80, 147.29, 140.06, 139.23, 136.23, 132.65, 130.11, 129.80, 127.61, 126.14, 121.40, 120.45, 116.18, 115.00, 112.13, 78.06, 71.29, 55.64, 52.33, 45.53, 36.73, 28.17.

#### *tert*-Butyl ((8*R*,11*S*)-3^2^-Amino-8-benzyl-4-hydroxy-7,10-dioxo-2-oxa-6,9-diaza-1,3(1,4)-dibenzenacyclododecaphane-11-yl)carbamate (**36**)

Compound **30** (0.25 mg, 0.42 mmol, 1 equiv) was dissolved in EtOH-H_2_O (6:1), then iron powder (0.47 mg, 8.47 mmol, 20 equiv) and ammonium chloride (22.66 mg, 0.42 mmol, 1 equiv) were added under vigorous stirring. The reaction mixture was refluxed for 2 h and filtered through a Celite pad. The filtrate was concentrated under reduced pressure and the residue was purified by preparative reversed-phase HPLC using a gradient from 20 to 80% acetonitrile in water containing 0.1% formic acid, to afford **36** (155 mg, 65%) as a light-yellow solid. LC-MS: *m*/*z* calculated 561.27, found 561.3 [M + H]^+^.

#### *tert*-Butyl ((8*S*,11*S*)-3^2^-Amino-4-hydroxy-7,10-dioxo-8-phenyl-2-oxa-6,9-diaza-1,3(1,4)-dibenzenacyclododecaphane-11-yl)carbamate (**37**)

Compound **31** (0.30 g, 0.52 mmol, 1 equiv) was added to Pd–C (37%, 111 mg) and MeOH (3 mL) was added under argon. The reaction mixture was stirred under 1 atm H_2_ in a parr hydrogenator. When LC-MS showed completion of the reaction after 1.5 h, the reaction mixture was filtered through a Celite pad. The solvent was removed under reduced pressure and the residue was purified by preparative reversed-phase HPLC using a gradient from 20 to 80% acetonitrile in water containing 0.1% formic acid, to afford **37** (150 mg, 53%) as white powder. LC-MS: *m*/*z* calculated 547.25, found 547.4 [M + H]^+^.

#### *tert*-Butyl ((8*S*,11*S*)-3^2^-Amino-8-benzyl-4-hydroxy-7,10-dioxo-2-oxa-6,9-diaza-1,3(1,4)-dibenzenacyclotridecaphane-11-yl)carbamate (**38**)

Compound **32** (1.00 g, 1.65 mmol, 1 equiv) was dissolved in EtOH-H_2_O (6:1), iron powder (1.73 g, 31.37 mmol, 19 equiv) and ammonium chloride (88.5 mg, 1.65 mmol, 1 equiv) were added under vigorous stirring. The reaction mixture was refluxed for 3 h and filtered through a Celite pad. The filtrate was concentrated under reduced pressure and the residue was purified on a silica gel column using 10 to 20% MeOH in EtOAc to afford **38** (605 mg, 64%). A part of this was further purified on reversed-phase HPLC using a gradient from 20 to 80% acetonitrile in water containing 0.1% formic acid for analytical characterization. LC-MS: *m*/*z* calculated 575.28, found 575.2 [M + H]^+^.

#### *tert*-Butyl ((8*S*,11*S*)-4-Hydroxy-8-methyl-7,10-dioxo-3^2^-(picolinamido)-2-oxa-6,9-diaza-1,3(1,4)-dibenzenacyclododecaphane-11-yl)carbamate (**39**)

2-Picolinic acid (19.1 mg, 0.16, 1 equiv) was dissolved in DCM (0.5 mL) and HATU (39.2 mg, 0.10 mmol, 1 equiv) was added in portions. DIPEA (26.8 μL, 0.16 mmol, 1.5 equiv) was added dropwise to the reaction mixture, followed by the addition of **33** (50 mg, 0.10 mmol, 1 equiv) dissolved in DCM (0.5 mL) and DIPEA (26.8 μL, 0.16 mmol, 1.5 equiv) and stirred at room temperature for 40 min. The solvents were removed under reduced pressure and the residue was purified on a silica gel column using 10% MeOH in EtOAc to afford **39** (45 mg, 74%). Part of the compound was further purified by preparative reversed-phase HPLC using a gradient from 20 to 80% acetonitrile in water containing 0.1% formic acid for analytical characterization. LC-MS: *m*/*z* calculated 590.26, found 590.2 [M + H]^+^.

#### *tert*-Butyl ((8*S*,11*S*)-4-Hydroxy-8-isobutyl-7,10-dioxo-3^2^-(picolinamido)-2-oxa-6,9-diaza-1,3(1,4)-dibenzenacyclododecaphane-11-yl)carbamate (**40**)

2-Picolinic acid (12.7 mg, 0.1 mmol, 1 equiv) was dissolved in DCM (0.5 mL) and HATU (39.2 mg, 0.10 mmol, 1 equiv) was added in portions. DIPEA (26.8 μL, 0.16 mmol, 1.5 equiv) was then added dropwise to the reaction mixture followed by the addition of **34** (54.34 mg, 0.10 mmol, 1 equiv) dissolved in DCM (0.5 mL) and DIPEA (26.8 μL, 0.16 mmol, 1.5 equiv) and stirred at room temperature for 40 min. The solvents were removed under reduced pressure and the residue was purified on a silica gel column using 10% MeOH in EtOAc to afford **40** (45 mg, 74%, as a mixture of two diastereomers). Part of the compound was further purified by preparative reversed-phase HPLC using a gradient from 20 to 80% acetonitrile in water containing 0.1% formic acid for analytical characterization. LC-MS: *m*/*z* calculated 664.31, found 664.4 [M + H]^+^.

#### *tert*-Butyl ((8*S*,11*S*)-8-Benzyl-4-hydroxy-7,10-dioxo-3^2^-(picolinamido)-2-oxa-6,9-diaza-1,3(1,4)-dibenzenacyclododecaphane-11-yl)carbamate (**41**)

Compound **35** (0.40 g, 0.713 mmol, 1 equiv) was dissolved in DMF (5 mL), picolinoyl chloride (0.20 mg, 1.07 mmol, 1.5 equiv) was added followed by DIPEA (248 μL, 1.43 mmol, 2 equiv) and DMAP (15 mg, 0.12 mmol, 0.17 equiv), after which the resulting solution was stirred at room temperature. The reaction was monitored by LC-MS and completed in 30 min. The solvents were removed under reduced pressure and the residue purified on a silica gel column using 60 to 100% EtOAc in hexanes to afford compound **41** (0.39 g, 82%) as a white powder. Part of the compound was further purified by preparative reversed-phase HPLC using a gradient from 20 to 80% acetonitrile in water containing 0.1% formic acid for analytical characterization. HRMS: *m*/*z* calculated 665.29, found 665.29. ^1^H NMR (400 MHz, DMSO-*d*_6_) δ 10.95–10.922 (m, 1H), 8.77 (m, 1H), 8.23 (m, 1H), 8.14 (m, 1H), 7.73 (m, 2H), 7.09 (m, 8H), 6.91 (m, 2H), 6.12 (dd, *J* = 24.7, 5.9 Hz, 1H), 5.58 (s, 1H), 4.89 (s, 1H), 4.46 (dd, *J* = 10.7, 5.2 Hz, 1H), 4.06 (s, 1H), 3.68 (s, 1H), 3.52 (d, *J* = 6.9 Hz, 1H), 2.89 (m, 4H), 2.69 (m, 2H), 1.41 (s, 9H). ^13^C NMR (100 MHz, DMSO-*d*_6_) δ 169.50, 169.26, 161.08, 159.36, 159.25, 154.71, 149.17, 149.13, 148.84, 138.82, 138.61, 136.41, 136.09, 133.75, 133.72, 130.69, 130.67, 130.65, 130.64, 129.68, 129.05, 127.57, 127.49, 127.33, 126.13, 126.01, 122.09, 122.07, 121.15, 78.04, 56.01, 52.17, 45.59, 36.75, 29.22, 28.20.

#### *tert*-Butyl ((8*R*,11*S*)-8-Benzyl-4-hydroxy-7,10-dioxo-3^2^-(picolinamido)-2-oxa-6,9-diaza-1,3(1,4)-dibenzenacyclododecaphane-11-yl)carbamate (**42**)

2-Picolinic acid (33.3 mg, 0.27 mmol, 1 equiv) was dissolved in DCM (0.5 mL) and HATU (102. Eight mg, 0.27 mmol, 1 equiv) was added in portions. DIPEA (70.2 μL, 0.41 mmol, 1.5 equiv) was then added dropwise to the reaction mixture followed by the addition of **36** (151.6 mg, 0.27 mmol, 1 equiv) dissolved in DCM (1.5 mL) and DIPEA (70.2 μL, 0.41 mmol, 1.5 equiv) and stirred at room temperature for 40 min. The solvents were removed under reduced pressure and the residue was purified on a silica gel column using 10% MeOH in EtOAc to afford **42** (125 mg, 70%) as a white powder. Part of the compound was further purified by preparative reversed-phase HPLC using a gradient from 20 to 80% acetonitrile in water containing 0.1% formic acid for analytical characterization. LC-MS: *m*/*z* calculated 666.29, found 666.6 [M + H]^+^.

#### *tert*-Butyl ((8*S*,11*S*)-4-Hydroxy-7,10-dioxo-8-phenyl-3^2^-(picolinamido)-2-oxa-6,9-diaza-1,3(1,4)-dibenzenacyclododecaphane-11-yl)carbamate (**43**)

2-Picolinic acid (6.76 mg, 0.055 mmol, 1 equiv) was dissolved in DCM (0.5 mL) and HATU (20.9 mg, 0.055 mmol, 1 equiv) was added in portions. DIPEA (14.3 μL, 0.082 mmol, 1.5 equiv) was then added dropwise to the reaction mixture followed by the addition of **37** (30 mg, 0.055 mmol, 1 equiv) dissolved in DCM (0.5 mL) and DIPEA (14.3 μL, 0.082 mmol, 1.5 equiv) and stirred at room temperature for 40 min. The solvents were removed under reduced pressure and the residue was purified on a silica gel column using 10% MeOH in EtOAc to afford **43** (26 mg, 73%, as a mixture of two diastereomers). Part of the compound was further purified by preparative reversed-phase HPLC using a gradient from 20 to 80% acetonitrile in water containing 0.1% formic acid for analytical characterization. LC-MS: *m*/*z* calculated 652.27, found 652.3 [M + H]^+^.

#### *tert*-Butyl ((8*S*,11*S*)-8-Benzyl-4-hydroxy-7,10-dioxo-3^2^-(picolinamido)-2-oxa-6,9-diaza-1,3(1,4)-dibenzenacyclotridecaphane-11-yl)carbamate (**44**)

2-Picolinic acid (3.57 mg, 0.03 mmol, 0.83 equiv) was dissolved in DMC (2 mL) and HATU (13.3 mg, 0.03 mmol, 1 equiv) and DIPEA (9 μL, 0.05 mmol, 1.5 equiv) were added to the solution followed by the addition of **38** (20 mg, 0.035 mmol, 1 equiv) dissolved in DCM (2 mL) and DIPEA (9 μL, 0.05 mmol, 1.5 equiv) and stirred at room temperature for 30 min. The solvents were removed under reduced pressure and the residue was purified on a silica gel column using 10% MeOH in EtOAc to afford **44** (16 mg, 67%). Part of the compound was further purified by preparative reversed-phase HPLC using a gradient from 20 to 80% acetonitrile in water containing 0.1% formic acid for analytical characterization. LC-MS: *m*/*z* 680.31, found 680.4 [M + H]^+^. ^1^H NMR (500 MHz, DMSO-*d*_6_) δ 10.83 (s, 1H), 8.75 (d, *J* = 5.3 Hz, 1H), 8.44 (s, 1H), 8.23 (d, *J* = 7.9 Hz, 1H), 8.12 (t, *J* = 7.6 Hz, 1H), 7.73 (d, *J* = 8.4 Hz, 1H), 7.20–7.00 (m, 9H), 6.92 (d, *J* = 8.9 Hz, 1H), 6.81 (d, *J* = 8.2 Hz, 1H), 6.58 (d, *J* = 8.4 Hz, 1H), 5.56 (d, *J* = 4.1 Hz, 1H), 4.82 (s, 1H), 4.12 (d, *J* = 7.5 Hz, 1H), 3.73 (t, *J* = 11.4 Hz, 1H), 2.87 (m, 1H), 2.76 (m, 2H), 2.70 (m, 2H), 1.83 (m, 2H), 1.68 (m, 2H), 1.41 (s, 6H), 1.25 (m, 3H). ^13^C NMR (125 MHz, DMSO-*d*_6_) δ 170.18, 169.20, 161.32, 157.77, 155.96, 149.18, 148.76, 148.46, 138.52, 137.78, 137.01, 130.23, 129.31, 129.11, 127.79, 127.27, 126.10, 122.63, 122.07, 121.18, 120.20, 117.13, 78.45, 70.34, 52.91, 51.39, 45.43, 40.05, 38.09, 31.49, 31.28, 28.20.

#### *tert*-Butyl ((8*S*,11*S*)-3^2^-Acetamido-8-benzyl-4-hydroxy-7,10-dioxo-2-oxa-6,9-diaza-1,3(1,4)-dibenzenacyclododecaphane-11-yl)picolinamide (**45**)

Compound **35** (0.41 g, 0.713 mmol, 1 equiv) was dissolved in THF (12 mL) and acetic anhydride (67.5 μL, 0.713 mmol, 1 equiv) was added followed by the addition of DIPEA (202 μL, 1.07 mmol, 1.5 equiv) and DMAP (14.81 mg, 0.12 mmol, 0.17 equiv) and stirred at room temperature. The reaction was monitored by LC-MS and found to be completed in 2 h. The solvents were removed under reduced pressure and the residue purified on a silica gel column using 60 to 100% EtOAc in hexanes. To avoid contamination of hydroxyl acetylated product, NaOMe in MeOH (0.5 M, 10 mL) was added to it for *O*-deacetylation. The reaction was completed in 10 min as monitored by LC-MS, the solvent was removed under reduced pressure and the reaction mixture was washed with aqueous HCl (1 M, 1 × 100 mL), saturated aqueous sodium hydrogen carbonate solution (1 × 100 mL), dried, filtered and concentrated under reduced pressure. Purification by preparative reversed-phase HPLC using a gradient from 20 to 80% acetonitrile in water containing 0.1% formic acid afforded **45** (312 mg, 73%). LC-MS: *m*/*z* calculated 603.28, found 603.3 [M + H]^+^.

#### *tert*-Butyl (*S*)-(1-((4-Fluoro-3-nitrophenethyl)amino)-1-oxo-3-phenylpropan-2-yl)carbamate (**47**)

Boc-l-Phe-OH (1.00 g, 5.43 mmol, 1 equiv) was dissolved in DCM (15 mL) and HATU (2.07 g, 5.43 mmol, 1 equiv) and DIPEA (1.42 mL, 8.14 mmol, 1.5 equiv) were added followed by the addition of **46** (1.00 g, 5.43 mmol, 1 equiv) dissolved in DCM and DIPEA (1.42 mL, 8.14 mmol, 1.5 equiv) and stirred at room temperature for 40 min. The reaction mixture was diluted with DCM (20 mL) and washed with an aqueous 1 M HCl solution (2 × 10 mL), saturated aqueous sodium bicarbonate solution (2 × 10 mL), and brine (2 × 10 mL), dried over anhydrous sodium sulfate, and filtered. The solvents were removed under reduced pressure and the residue was purified on a silica gel column using 50% EtOAc in hexanes to afford **47** (1.55 g, 66%) as a light-yellow powder. A small portion of this material was purified by preparative reversed-phase HPLC using a gradient from 20 to 80% acetonitrile in water containing 0.1% formic acid for analytical characterization. LC-MS: *m*/*z* calculated 432.19, found 432.2 [M + H]^+^.

#### *tert*-Butyl ((*S*)-3-(4-((*tert*-Butyldimethylsilyl)oxy)phenyl)-1-(((*S*)-1-((4-fluoro-3-nitrophenethyl)amino)-1-oxo-3-phenylpropan-2-yl)amino)-1-oxopropan-2-yl)carbamate (**48**)

Compound **14** (0.92 g, 2.32 mmol, 1 equiv) was dissolved in DCM (10 mL) and HATU (0.97 g, 2.32 mmol, 1 equiv), and DIPEA (0.64 mL, 3.72 mmol, 1.5 equiv) were added. Compound **47** (1.00 g, 2.32 mmol, 1 equiv) was dissolved in 4 M HCl in dioxane (4 mL) at room temperature and was stirred for 30 min followed by removal of solvents under reduced pressure. The resulting oil was dissolved in DCM (15 mL) and DIPEA (0.64 mL, 3.72 mmol, 1.5 equiv), after which this solution was added to the solution containing compound **14** and stirred for 40 min when LC-MS showed complete conversion. The solvents were removed under reduced pressure and the residue was purified on a silica gel column using 0 to 10% MeOH in EtOAc to afford **48** (905 mg, 55%) as a white powder. A small portion of this material was then concentrated under reduced pressure and purified by preparative reversed-phase HPLC using a gradient from 20 to 80% acetonitrile in water containing 0.1% formic acid for analytical characterization. LC-MS: *m*/*z* calculated 709.34, found 709.4 [M + H]^+^.

#### *tert*-Butyl ((8*S*,11*S*)-8-Benzyl-3^2^-nitro-7,10-dioxo-2-oxa-6,9-diaza-1,3(1,4)-dibenzenacyclododecaphane-11-yl)carbamate (**49**)

Compound **48** (0.50 g, 0.71 mmol, 1 equiv) was dissolved in DMF (100 mL), CsF (2.20 g, 14.5 mmol, 20 equiv) was added in portions and the reaction mixture was stirred at 50 °C. After 3 h the solvent was evaporated under reduced pressure and the residue was purified on a silica gel column using 10 to 20% MeOH in EtOAc to afford **49** (335 mg, 85%) as a pale-orange powder. A part of this was further purified on reversed-phase HPLC using a gradient from 20 to 80% of acetonitrile in water containing 0.1% formic acid for analytical characterization. LC-MS: *m*/*z* calculated 575.25, found 575.6 [M + H]^+^.

#### *tert*-Butyl ((8*S*,11*S*)-3^2^-Amino-8-benzyl-7,10-dioxo-2-oxa-6,9-diaza-1,3(1,4)-dibenzenacyclododecaphane-11-yl)carbamate (**50**)

Compound **49** (0.25 g, 0.45 mmol, 1 equiv) was dissolved in EtOH-H_2_O (6:1), then iron powder (0.50 g, 8.92 mmol, 20 equiv) and ammonium chloride (23.8 mg, 0.45 mmol, 1 equiv) were added under vigorous stirring. The reaction mixture was refluxed for 3 h and filtered through a Celite pad and concentrated under reduced pressure to afford **50** (156 mg, 66%) as a light-yellow solid. A part was then dried under reduced pressure and purified by preparative reversed-phase HPLC using a gradient from 20 to 80% of acetonitrile in water containing 0.1% formic acid for analytical characterization. LC-MS: *m*/*z* calculated 545.27, found 545.3 [M + H]^+^.

#### *tert*-Butyl ((*S*)-1-((2-Hydroxy-2-(3-nitrophenyl)ethyl)amino)-1-oxo-3-phenylpropan-2-yl)carbamate (**52**)

Boc-l-Phe-OH (1.00 g, 4.57 mmol, 1 equiv) was dissolved in DCM (15 mL), HATU (2.28 g, 5.99 mmol, 1 equiv) and DIPEA (1.76 mL, 7.6 mmol, 1.5 equiv) were added followed by the addition of **51** (1.20 g, 10.11 mmol, 1 equiv) dissolved in DCM (10 mL) and DIPEA (1.76 mL, 7.6 mmol, 1.5 equiv) and stirred at room temperature for 40 min. The reaction mixture was diluted with DCM (20 mL) and washed with an aqueous 1 M HCl solution (2 × 10 mL), saturated aqueous sodium bicarbonate solution (2 × 10 mL) and brine (2 × 10 mL), dried over anhydrous sodium sulfate, and filtered. The solvents were removed under reduced pressure and the residue purified on a silica gel column using 50% EtOAc in *n*-hexane to afford **52** (1.45 g, 74%) as a light-yellow powder. A small portion of this material was purified by preparative reversed-phase HPLC using a gradient from 20 to 80% of acetonitrile in water containing 0.1% formic acid for analytical characterization. LCMS: *m*/*z* calculated 452.18, found 452.2 [M + Na]^+^. ^1^H NMR (400 MHz, DMSO-*d*_6_) δ 8.22 (d, *J* = 6.9 Hz, 1H), 8.12 (dd, *J* = 8.2, 2.5 Hz, 1H), 8.00 (q, *J* = 6.1 Hz, 1H), 7.80 (d, *J* = 7.6 Hz, 1H), 7.76 (d, *J* = 7.4 Hz, 1H), 7.63–7.58 (m, 1H), 7.19 (dq, *J* = 16.3, 7.9 Hz, 5H), 6.83 (dd, *J* = 17.8, 8.7 Hz, 1H), 5.85 (s, 1H), 4.80 (q, *J* = 6.1 Hz, 1H), 4.10 (qd, *J* = 9.5, 4.1 Hz, 1H), 3.46–3.26 (m, 4H), 2.82 (dt, *J* = 13.7, 4.3 Hz, 1H), 2.64–2.56 (m, 1H), 1.28 (d, *J* = 5.1 Hz, 9H). ^13^C NMR (100 MHz, DMSO-*d*_6_) δ 171.97, 171.91, 155.22, 155.15, 147.64, 147.60, 145.89, 145.83, 138.26, 133.08, 133.01, 129.48, 129.45, 129.11, 127.96, 127.94, 126.12, 126.10, 122.01, 121.96, 120.84, 120.81, 77.95, 77.89, 70.33, 55.78, 55.59, 46.18, 46.08, 37.62, 37.46, 28.11.

#### *tert*-Butyl ((*S*)-1-(((*S*)-1-((2-Hydroxy-2-(3-nitrophenyl)ethyl)amino)-1-oxo-3-phenylpropan-2-yl)amino)-3-(4-methoxyphenyl)-1-oxopropan-2-yl)carbamate (**53**)

(*S*)-2-((*tert*-Butoxycarbonyl)amino)-3-(4-methoxyphenyl)propanoic acid (1.74 g, 2.79 mmol, 1 equiv) was dissolved in DCM (20 mL) and HATU (1.06 g, 2.79 mmol, 1 equiv) and DIPEA (0.73 mL, 4.19 mmol, 1.5 equiv) were added. Compound **52** (1.2 g, 2.79 mmol, 1 equiv) was dissolved in 4 N HCl in dioxane (4 mL) and stirred for 30 min followed by removal of the solvent under reduced pressure. The residue was dissolved in DCM (15 mL) and DIPEA (0.73 mL, 4.19 mmol, 1.5 equiv), and then added to the solution containing (*S*)-2-((*tert*-butoxycarbonyl)amino)-3-(4-methoxyphenyl)propanoic acid and stirred for 30 min. The solvents were removed under reduced pressure and the residue was purified on a silica gel column using 0 to 10% MeOH in EtOAc to afford **53** (980 mg, 60%) as a white powder. A small portion of this material was further purified on reversed-phase HPLC using a gradient from 20 to 80% of acetonitrile in water containing 0.1% formic acid for analytical characterization. LC-MS: *m*/*z* calculated 607.27, found 607.3 [M + H]^+^. ^1^H NMR (400 MHz, CD_3_CN) δ 8.20 (s, 1H), 8.10 (dd, *J* = 8.2, 2.5 Hz, 1H), 7.71 (m, 1.13), 7.56 (m, 1H), 7.30–7.21 (m, 6H), 7.17 (t, *J* = 8.2 Hz, 2H), 7.05 (d, *J* = 8.5 Hz, 3H), 6.92 (t, *J* = 6.6 Hz, 2H), 6.82 (d, *J* = 8.7 Hz, 3H), 5.48 (d, *J* = 7.0 Hz, 1H), 4.84–4.81 (m, 1H), 4.47–4.44 (m, 1H), 4.31 (s, 1H), 4.09–4.06 (m, 1H), 3.74 (s, 3H), 3.45–3.237 (m, 3H), 3.04 (dd, *J* = 13.2, 6.2 Hz, 1H), 2.92–2.83 (m, 3H), 2.67–2.61 (m, 2H), 1.31 (s, 9H). ^13^C NMR (100 MHz, CD_3_CN) δ 172.59, 159.50, 149.34, 145.89, 138.28, 133.53, 131.27, 130.44, 130.30, 129.40, 129.37, 127.68, 127.65, 123.26, 123.23, 121.89, 121.86, 114.72, 80.38, 72.48, 72.25, 57.33, 55.83, 55.24, 47.73, 38.27, 37.48, 28.49.

#### *tert*-Butyl ((*S*)-1-(((*S*)-1-((-2-(3-Aminophenyl)-2-hydroxyethyl)amino)-1-oxo-3-phenylpropan-2-yl)amino)-3-(4-methoxyphenyl)-1-oxopropan-2-yl)carbamate (**54**)

Compound **53** (0.50 g, 0.71 mmol, 1 equiv) was dissolved in EtOH-H_2_O (6:1), followed by addition of iron powder (0.79 g, 14.1 mmol, 20 equiv) and ammonium chloride (37.8 mg, 0.71 mmol, 1 equiv) under vigorous stirring. The reaction mixture was refluxed for 2 h until LC-MS showed completion of the reaction, filtered through a Celite pad and concentrated under reduced pressure to afford **54** (330 mg, 69%) as a light-yellow solid. A part was further purified on reversed-phase HPLC using a gradient from 20 to 80% of acetonitrile in water containing 0.1% formic acid for analytical characterization. LC-MS: *m*/*z* calculated 577.30, found 577.3 [M + H]^+^. ^1^H NMR (500 MHz, CD_3_CN) δ 7.30–7.22 (m, 2H), 7.20–7.16 (m, 4H), 7.06–7.03 (m, 3H), 6.98 (d, *J* = 7.9 Hz, 1H), 6.83 (dd, *J* = 17.5, 8.7 Hz, 3H), 6.63–6.59 (m, 3H), 6.55–6.53 (m, 2H), 5.51 (d, *J* = 7.8 Hz, 1H), 4.52–4.49 (m, 2H), 4.14–4.13 (m, 1H), 3.73 (s, 3H), 3.38–3.30 (m, 1H), 3.24–3.13 (m, 1H), 3.07 (dd, *J* = 14.0, 5.6 Hz, 1H), 2.94–2.86 (m, 2H), 2.69–2.64 (m, 1H), 1.33 (s, 9H). ^13^C NMR (125 MHz, CD_3_CN) δ 172.55, 172.50, 172.26, 159.37, 156.60, 148.55, 144.58, 144.54, 138.24, 131.19, 130.29, 130.07, 129.90, 129.89, 129.29, 129.27, 127.55, 115.87, 115.85, 114.59, 114.56, 113.06, 113.03, 80.23, 73.45, 73.17, 57.20, 55.71, 55.13, 48.06, 38.37, 38.31, 37.46, 28.39.

#### *tert*-Butyl ((*S*)-1-(((*S*)-1-((-2-Hydroxy-2-(3-(picolinamido)phenyl)ethyl)amino)-1-oxo-3-phenylpropan-2-yl)amino)-3-(4-methoxyphenyl)-1-oxopropan-2-yl)carbamate (**55**)

2-Picolinic acid (9.09 mg, 0.07 mmol, 1 equiv) was dissolved in DCM (0.5 mL) and HATU (28.1 mg, 0.07 mmol, 1 equiv) was added in portions. DIPEA (12.9 μL, 0.07 mmol, 1.5 equiv) was then added dropwise to the reaction mixture followed by **54** (50 mg, 0.07 mmol, 1 equiv) dissolved in DCM (1.5 mL) and DIPEA (12.9 μL, 0.07 mmol, 1.5 equiv) and stirred at room temperature for 40 min. The solvents were removed under reduced pressure and the residue purified on a silica gel column using 10% MeOH in EtOAc to afford **55** (30 mg, 52%, as a mixture of two diastereomers). A part was further purified on reversed-phase HPLC using a gradient from 20 to 80% of acetonitrile in water containing 0.1% formic acid for analytical characterization. LC-MS: *m*/*z* calculated 682.32, found 682.4 [M + H]^+^. ^1^H NMR (400 MHz, CD_3_CN) δ 8.41 (s, 1H), 8.26 (s, 1H), 7.51 (m, 2H), 7.26–7.20 (m, 6H), 7.17–6.89 (m, 4H), 6.79 (d, *J* = 8.8 Hz, 3H), 5.34 (s, 1H), 4.68–4.63 (m, 1H), 4.51 (d, *J* = 5.6 Hz, 0H), 4.13–4.09 (m, 1H), 3.73 (s, 3H), 3.34 (m, 2H), 3.07–2.88 (m, 4H), 2.68 (t, *J* = 10.4 Hz, 2H), 1.31 (s, 9H). ^13^C NMR (100 MHz, CD_3_CN) δ 172.64, 172.48, 163.03, 160.36, 160.33, 159.41, 156.66, 144.34, 144.28, 138.63, 138.56, 138.07, 137.98, 131.19, 130.26, 130.23, 129.78, 129.73, 129.37, 129.34, 127.66, 127.63, 122.87, 122.60, 122.53, 119.68, 119.64, 114.71, 79.04, 78.85, 78.59, 73.06, 57.30, 55.90, 55.13, 48.19, 38.26, 38.19, 37.52, 28.71.

### Inhibition of Parasite Growth

The growth of the *P. falciparum* blood stage 3D7 strain,^53^ the two *T. brucei* species in axenic cultures,^54^*T. cruzi* in infected human fibroblasts (MRC-5 cell line),^55^ and of *L. infantum* in infected primary mouse macrophages (PMMs)^56^ was determined as previously reported in the presence and absence of inhibitors **1**–**12**.

### Aqueous Solubility, Log *D*, Cell Permeability and Metabolic Stability

The kinetic solubility in phosphate buffered saline at pH 7.4, chromatographic Log *D* at pH 7.4, cell permeability across a MDCK-MDR1 cell monolayer at pH 7.4, and the clearance in mouse liver microsomes of **1**–**12** was determined at TCG Lifesciences.

### Conformational Analysis

Conformational sampling and selection of conformations for QM calculation were done as previously described, with the following modifications.^21^ The geometry of the selected conformations from conformational sampling was optimized using the DFT method (B3LYP with Def2-SVP basis set) using the conductor-like polarizable continuum model (CPCM) implicit solvation model.^57^ Subsequently, energies (entropy, enthalpy, and Gibbs free energy contributions) and trajectories were extracted and analyzed. All QM calculations were performed using the ORCA tool (version 5.0).^21,46^

### Crystal Structure Prediction

Crystal structure prediction was conducted using the program GRACE 2.8 with a point charge force field parametrization scheme as already described.^27,58^ The final energy ranking of the crystal structures was performed using periodic DFT-D, using the Perdew–Burke–Ernzerhof (PBE) functional with the Neumann-Perrin (NP) dispersion correction, a plane wave basis set (520 eV, 2π × 0.07 Å^–1^*k*-point grid), and default projected-augmented wave (PAW) pseudopotentials, as implemented in VASP 5.4.1.^59−63^

### Free Energy Perturbation Based Solubility Prediction

A Free Energy Perturbation (FEP) based protocol was used to compute aqueous crystalline and amorphous solubility, as previously described.^22,24^ Here, the predictions rely on turning off the intermolecular interactions of the investigated molecule in an amorphous aggregate, or crystalline supercell, in water to compute an average sublimation free energy. The hydration-free energy is computed through a process of inserting the molecule of interest into a box of water through turning on its intermolecular interactions with water. The production runs of the simulation were conducted for 20 ns and 24 lambda windows using the Desmond Molecular Dynamics package, as distributed by Schrodinger, with the OPLS-4 force field.^64,65^

## Supporting Information Available

Potencies of **1**–**12** as inhibitors of the four kinetoplastid parasites, low energy conformations of **3** and **5** obtained by Monte Carlo–QM calculations, metabolic liabilities of **3**, additional structure–property correlations for **1**–**12**, NMR spectra for **1**–**12** and intermediates in their synthesis, LCMS characterization of **1**–**12** (PDF)
Structures (SMILES codes), inhibitory potencies and *in vitro* ADME data for **1**–**12** (CSV)

## Abbreviations Used

Boc: *tert*-butyloxycarbonyl
CsF: cesium fluoride
CSP: crystal structure prediction
DIPEA: *N*,*N*-diisopropylethylamine
ER: efflux ratio
FEP: free energy perturbation
HATU: hexafluorophosphate azabenzotriazole tetramethyl uronium
IBX: 2-iodoxybenzoic acid
MEC: minimum energy conformation
MDCK-MDR1: Madin-Darby canine kidney cells transfected with the human MDR1 gene
*P*_app_ A→B: permeability across a MDCK MDR1 monolayer in the apical-to-basolateral direction
PMM: CC_50_, primary mouse macrophages 50% cellular cytotoxicity
Ro5: Lipinski’s rule of five
