# Supplementary material for: Discovery of a Series of Macrocycles as Potent Inhibitors of Leishmania Infantum
Source: J Med Chem. 2024 Oct 8;67(20):18170–93. doi: 10.1021/acs.jmedchem.4c01370 (PMC11513892; doi:10.1021/acs.jmedchem.4c01370)

SUPPORTING INFORMATION

Discovery of a Series of Macrocycles as Potent Inhibitors of Leishmania Infantum

Federico Riu,[a] Larissa Alena Ruppitsch,[a] Duc Duy Vo,[a,b] Richard S. Hong,[c] Mohit Tyagi,[a] An Matheeussen,[d] Sarah Hendrickx,[d] Vasanthanathan Poongavanam,[a] Guy Caljon,[d] Ahmad Y. Sheikh,[e] Peter Sjö,[f,*] Jan Kihlberg[a,*]

[a]*Department of Chemistry – BMC, Uppsala University, 751 23 Uppsala, Sweden*

[b]*Science for Life Laboratory, Department of Cell and Molecular Biology, Uppsala University, 751 24 Uppsala, Sweden*

[c]*Molecular Profiling and Drug Delivery, Research & Development, AbbVie Inc., Worcester, MA 01605, USA*

[d]*Laboratory of Microbiology, Parasitology and Hygiene, University of Antwerp, Universiteitsplein 1, 2610 Wilrijk, Belgium*

[e]*Molecular Profiling and Drug Delivery, Research & Development, AbbVie Inc, North Chicago, IL 60064, USA*

[f]*Drugs for Neglected Diseases initiative (DNDi), 15 Chemin Camille-Vidart, 1202 Geneva, Switzerland*

Corresponding authors

Peter Sjö, psjo@dndi.org, ORCID: 0000-0002-6768-0536

Jan Kihlberg, jan.kihlberg@kemi.uu.se, ORCID: 0000-0002-4205-6040

CONTENTS

- Page S3
  In vitro potencies for inhibition of the growth of four kinetoplastid parasites by compounds **1-12** (Table S1).

- Page S3
  Endo and *exo* conformations for compound **3**, obtained using the Monte Carlo-QM protocol for conformational sampling (Figure S1).

- Page S4
  Endo and *exo* conformations for compound **5**, obtained using the Monte Carlo-QM protocol for conformational sampling (Figure S2).

- Page S4
  Metabolically labile sites (indicated by red circles) observed for compound **3** after incubation with rat liver microsomes. (Figure S3).

- Page S5
  Correlations between **A**) the permeability across MDCK-MDR1 cell monolayers and $LogD_{7.4}$ and **B**) the potency for inhibition of the growth of *L. infantum* and the cell permeability for compounds **1-12** (Figure S4).

- Pages S6-S51
  NMR spectra.

- Pages S52-S57
  HPLC chromatograms and mass spectra.

Table S1. *In vitro* potencies for inhibition of the growth of four kinetoplastid parasites by compounds **1-12**.[a]

COMPOUNDS		1	2	3	4	5	6	7	8	9	10	11	12
EC_{50}	*T. b. brucei*	6.1 (2.0)	37 (24)	15 (10)	33 (0.2)	7.4 (3.0)	12 (11)	49 (15)	8.1 (0.1)	7.7 (0)	8.5 (0.8)	36 (19)	30 (1.4)
	T. b. rhodesiense	6.0 (1.4)	29 (26)	9.1 (4.6)	6.1 (0.9)	5.6 (2.4)	6.3 (2.4)	9.7 (1.4)	6.4 (0.1)	1.9 (0.1)	2.1 (0.4)	24 (10)	21 (13)
	T. cruzi	15 (4.7)	41 (26)	20 (18)	64 (0)	18 (14)	19 (11)	36 (5.6)	6.1 (2.1)	19 (11)	50 (15)	33 (7.9)	64 (0)
	L. infantum	33 (0.4)	31 (19)	1.8 (1.9)	38 (26)	17 (14)	12 (5.9)	16 (4.7)	8.2 (0.1)	6.6 (1.5)	2.1 (0.6)	38 (7.8)	7.1 (1.0)

[a] Mean values, with standard deviation in parenthesis, from 2 to 14 measurements.

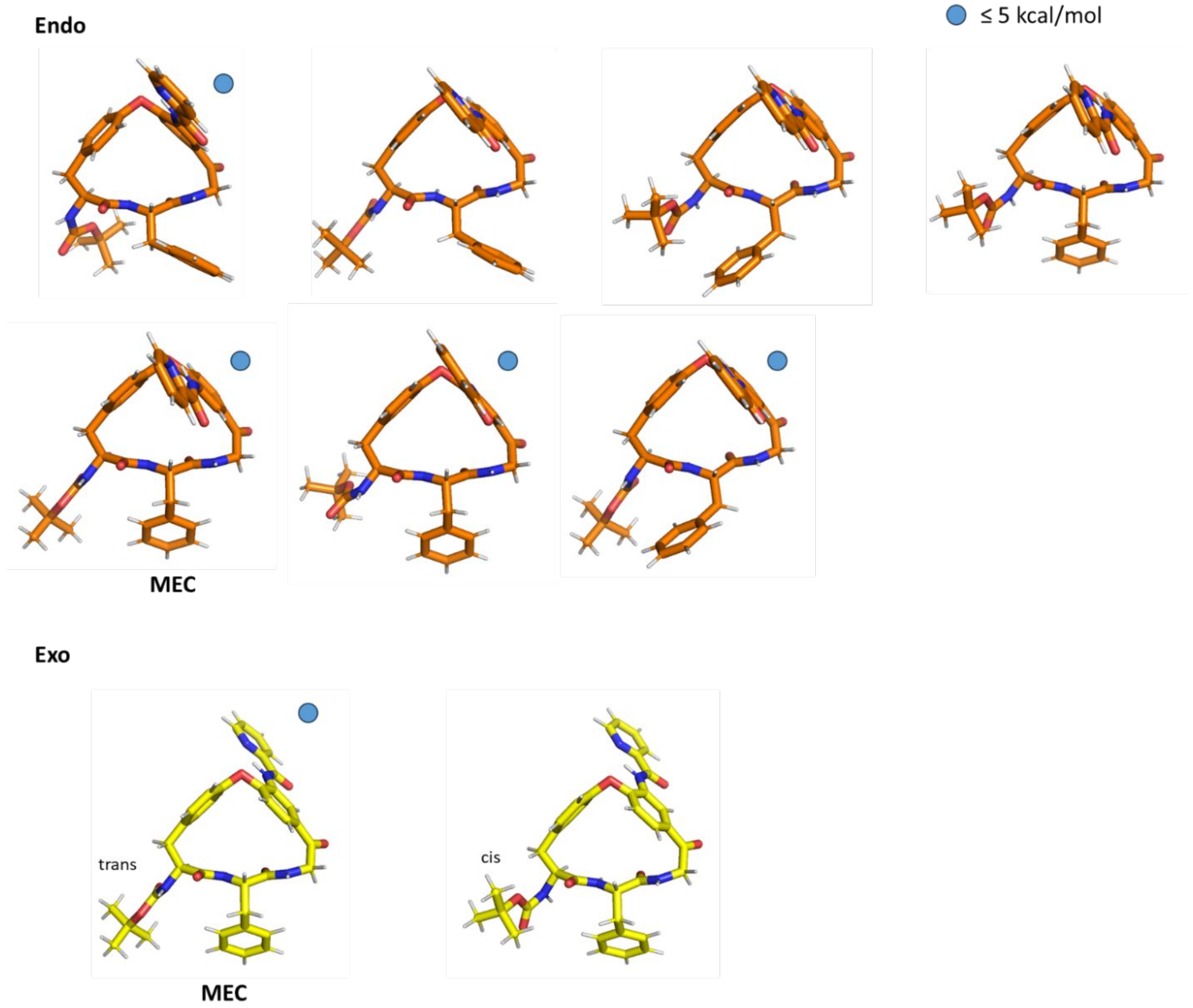

Figure S1. *Endo* and *exo* conformations for compound **3**, obtained using the Monte Carlo-QM protocol for conformational sampling. Minimum energy conformations (MECs) and all conformations within 5 kcal/mol of the MECs are shown.

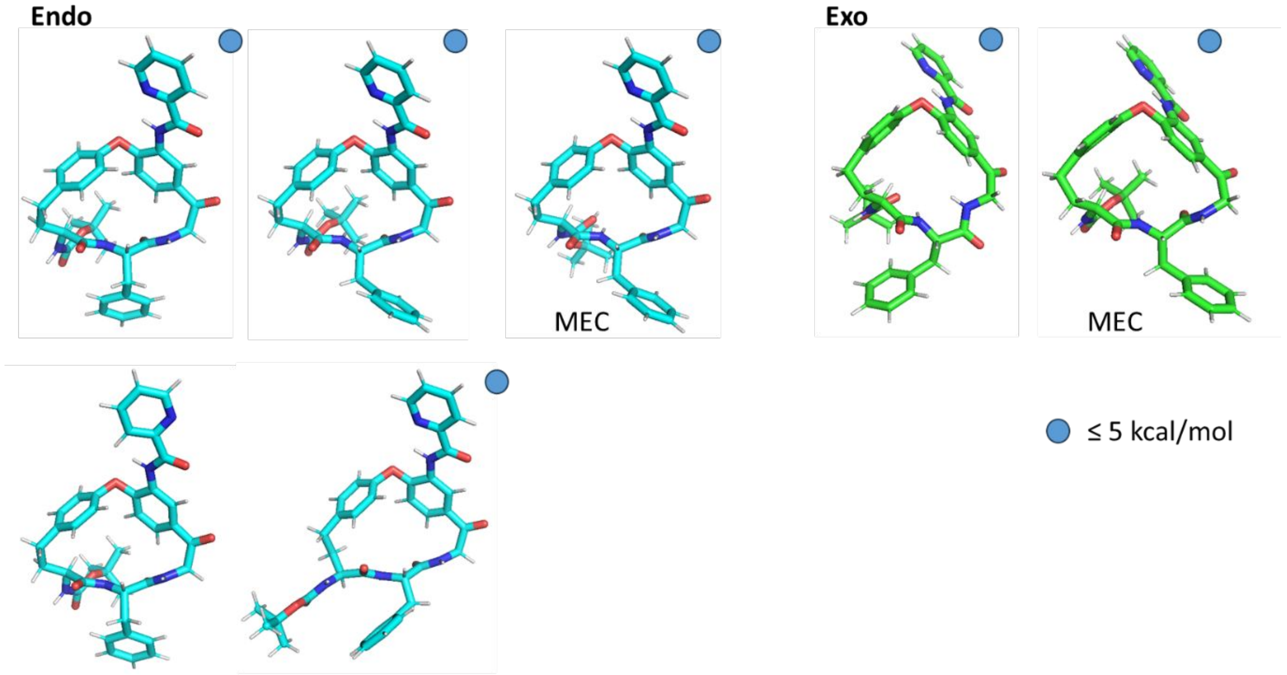

Figure S2. *Endo* and *exo* conformations for compound **5**, obtained using the Monte Carlo-QM protocol for conformational sampling. Minimum energy conformations (MECs) and all conformations within 5 kcal/mol of the MECs are shown.

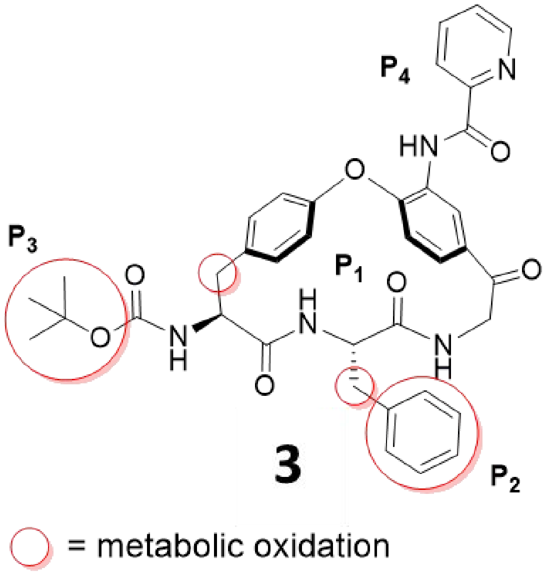

Figure S3. Metabolically labile sites (indicated by red circles) observed for compound **3** after incubation with rat liver microsomes. Samples were collected at 5, 10, 20, 30 and 60 minutes.

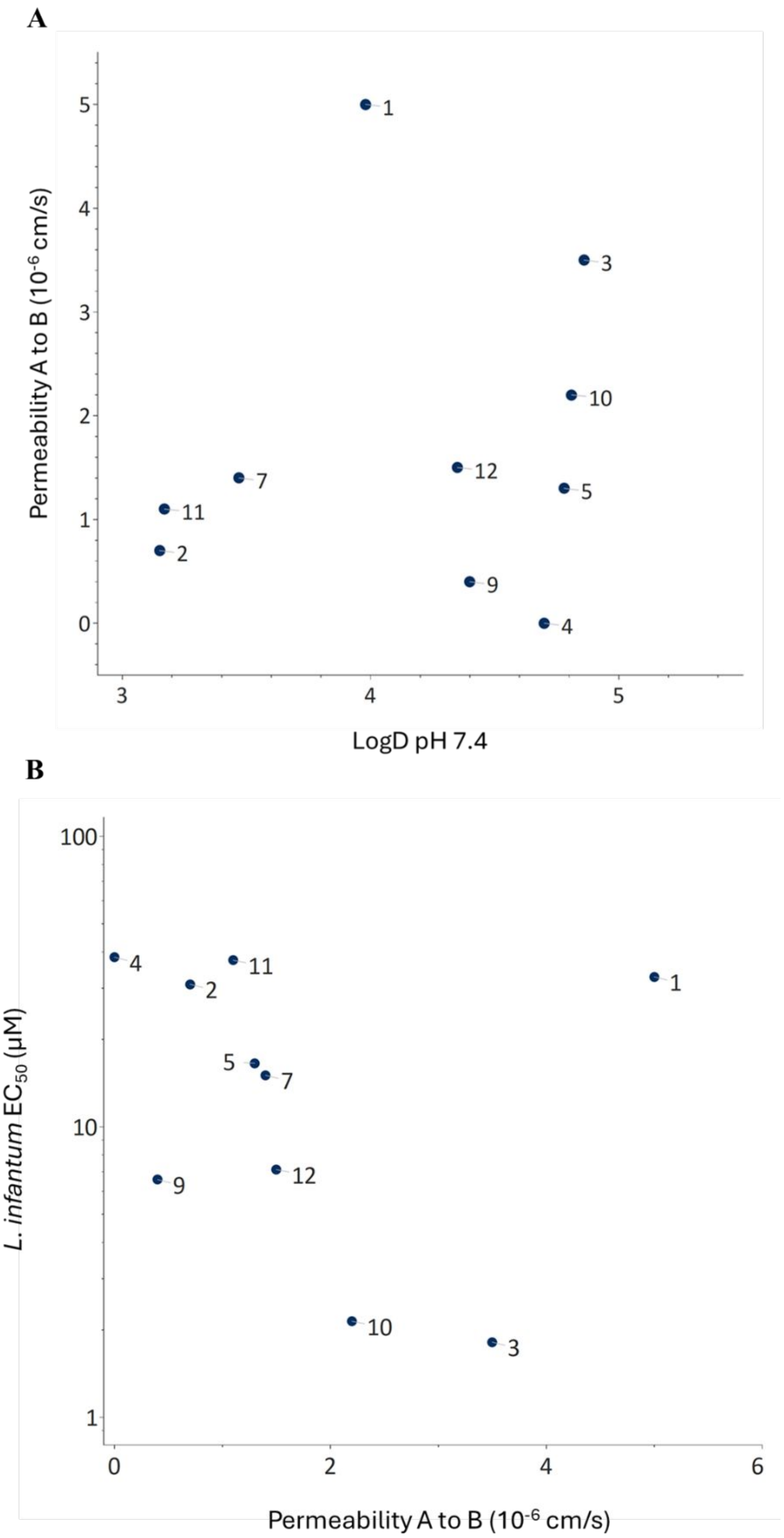

Figure S4. Correlations between **A**) the permeability across MDCK-MDR1 cell monolayers and $LogD_{7.4}$ and **B**) the potency for inhibition of the growth of *L. infantum* and the cell permeability for compounds **1-12**.

NMR SPECTRA

Compound 2: ^{1}H NMR ($CDCl_3$, 400 MHz)

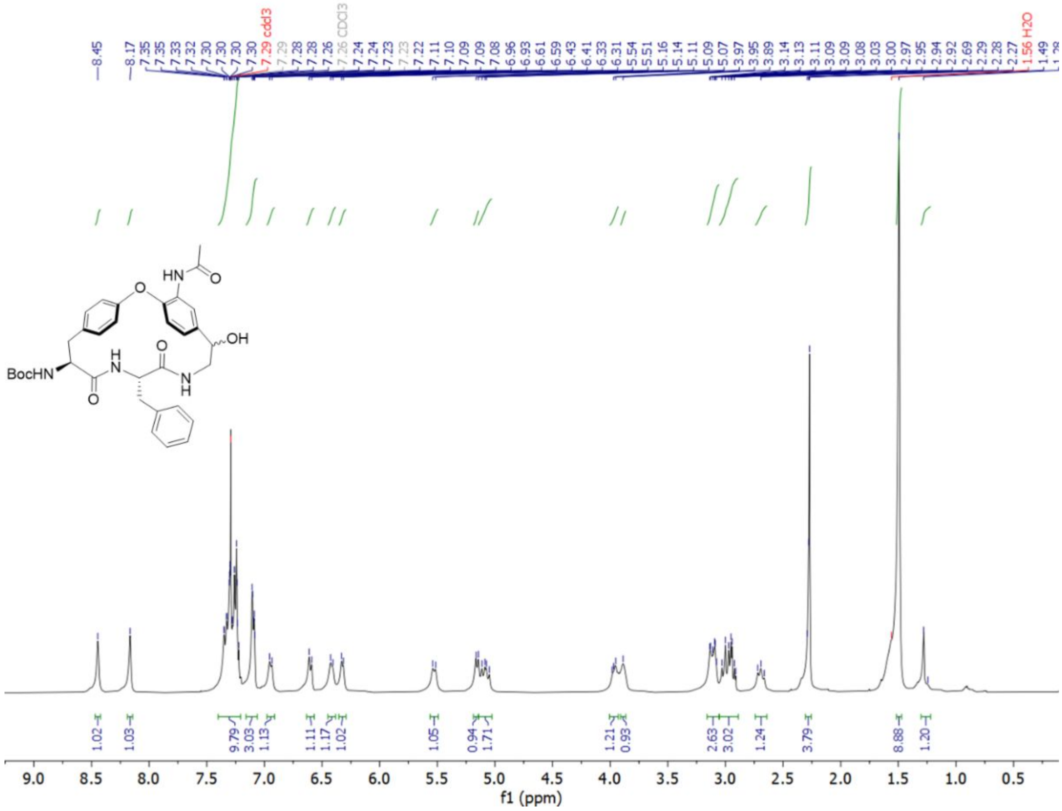

Compound 2: ^{13}C NMR ($CDCl_3$, 100 MHz)

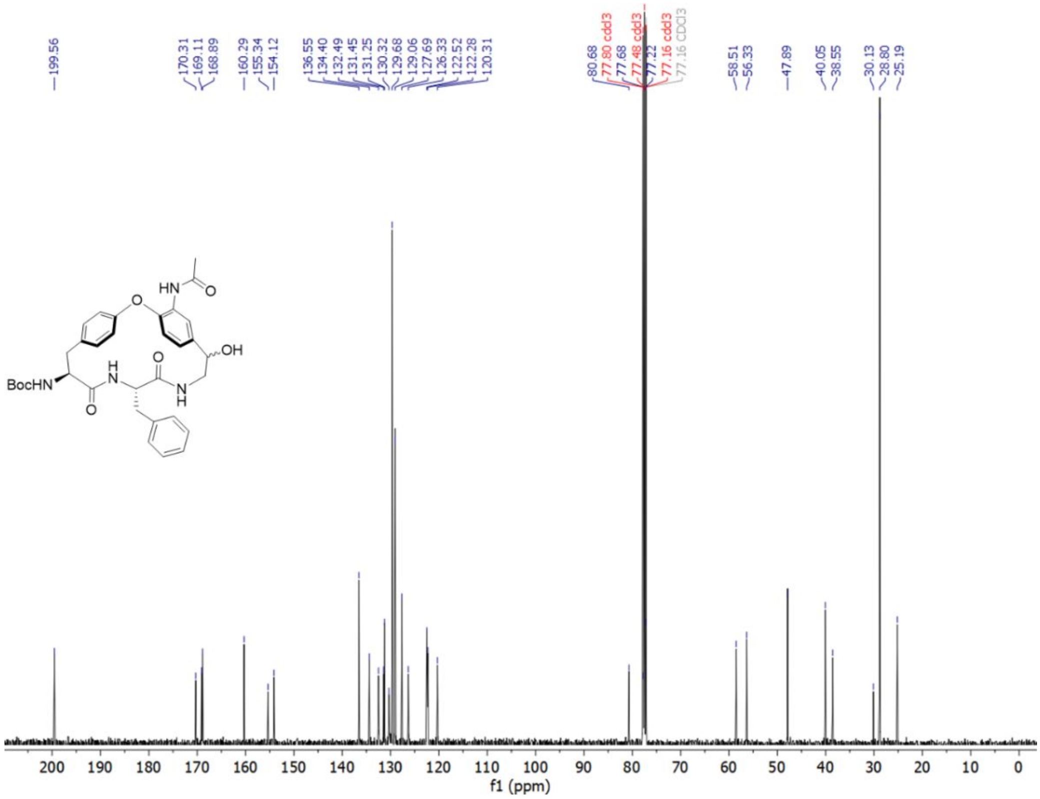

Compound 2: COSY NMR ($CDCl_3$)

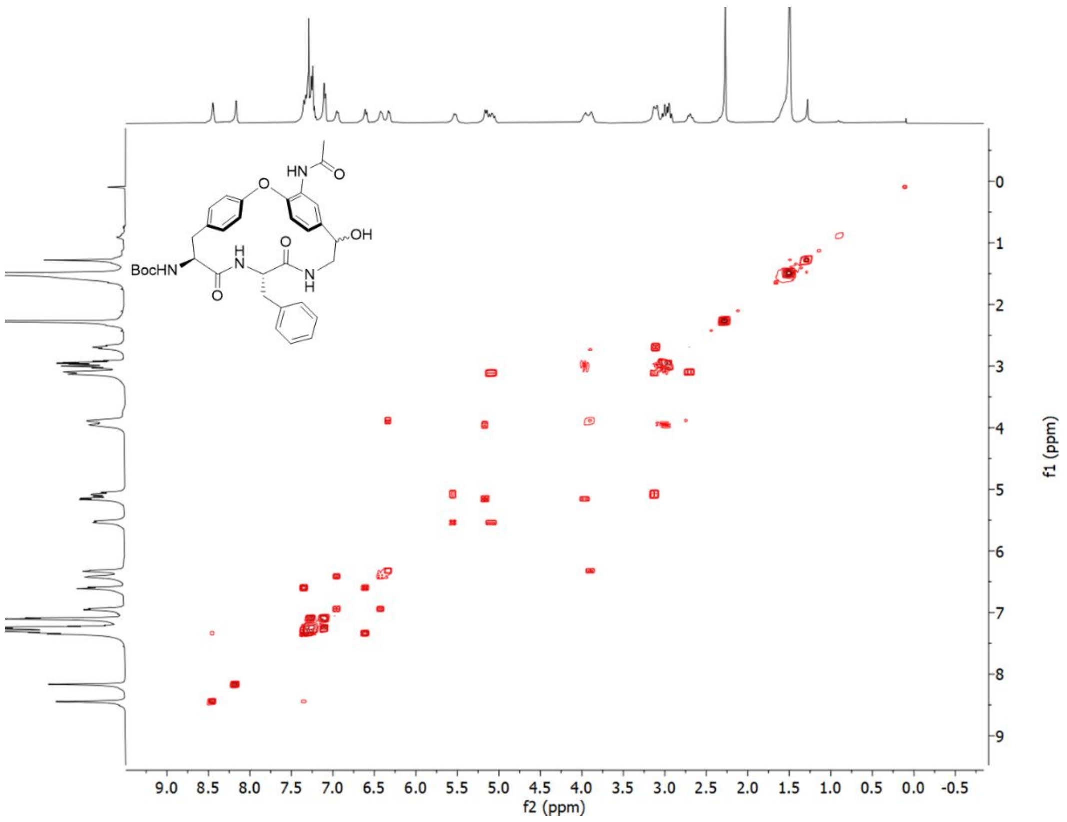

Compound 2: HSQC NMR ($CDCl_3$)

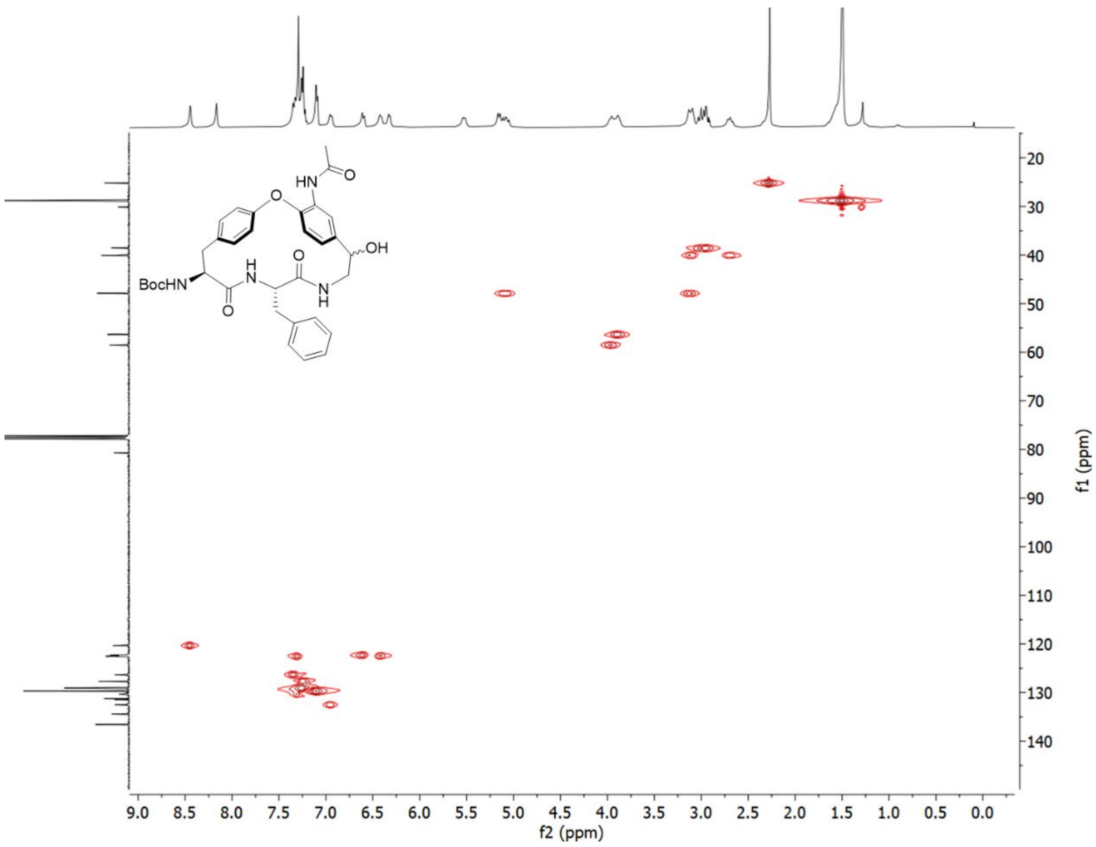

Compound 2: HMBC NMR ($CDCl_3$)

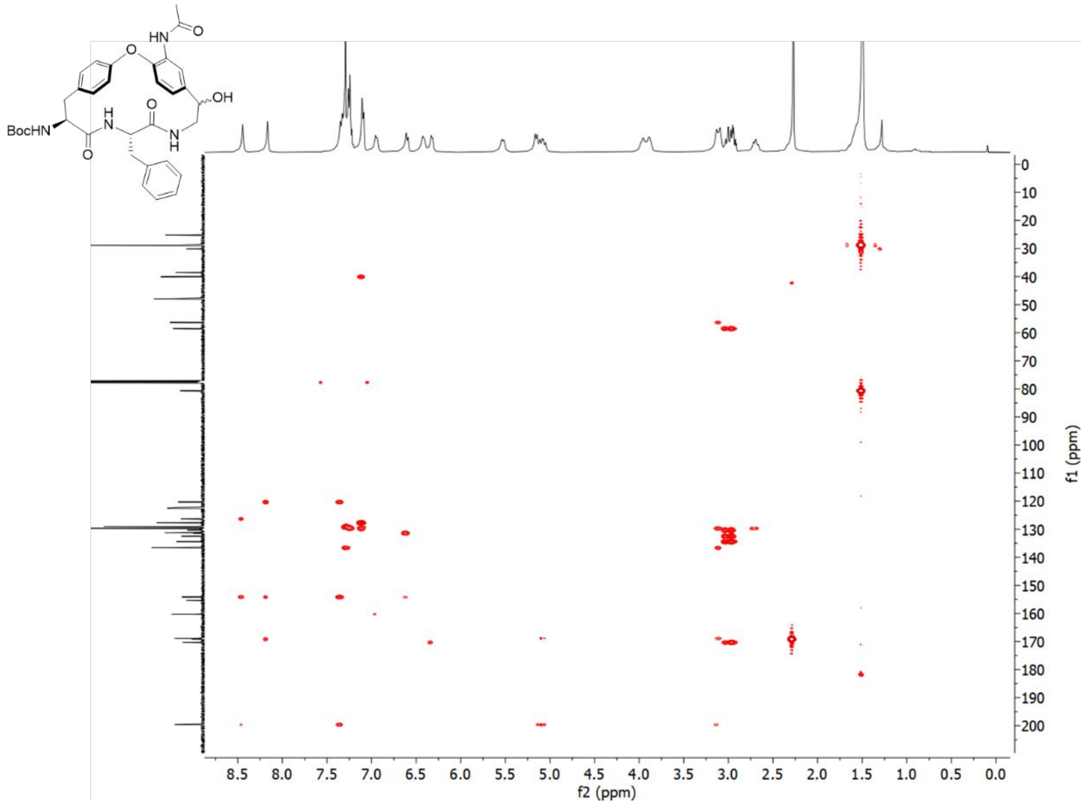

Compound 3: ^{1}H NMR ($CDCl_3$, 600 MHz)

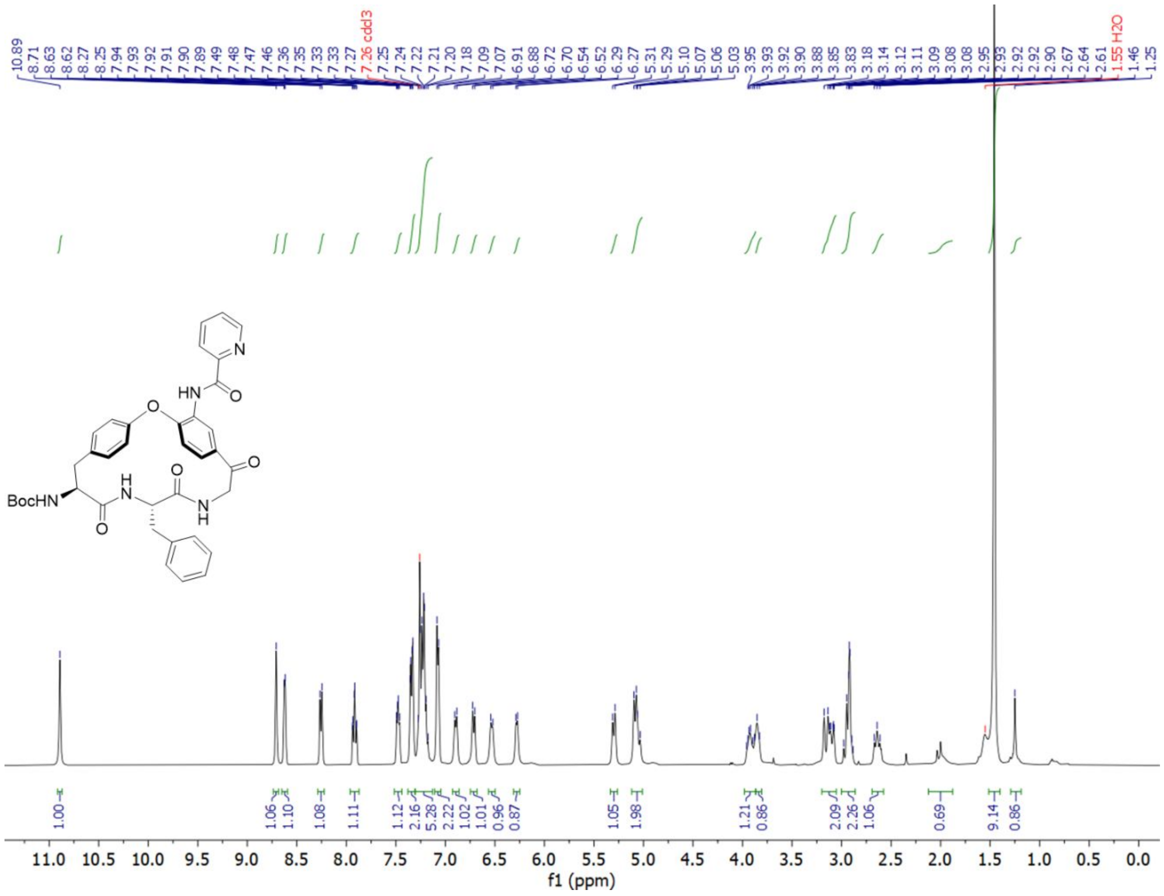

Compound 3: ^{13}C NMR ($CDCl_3$, 150 MHz)

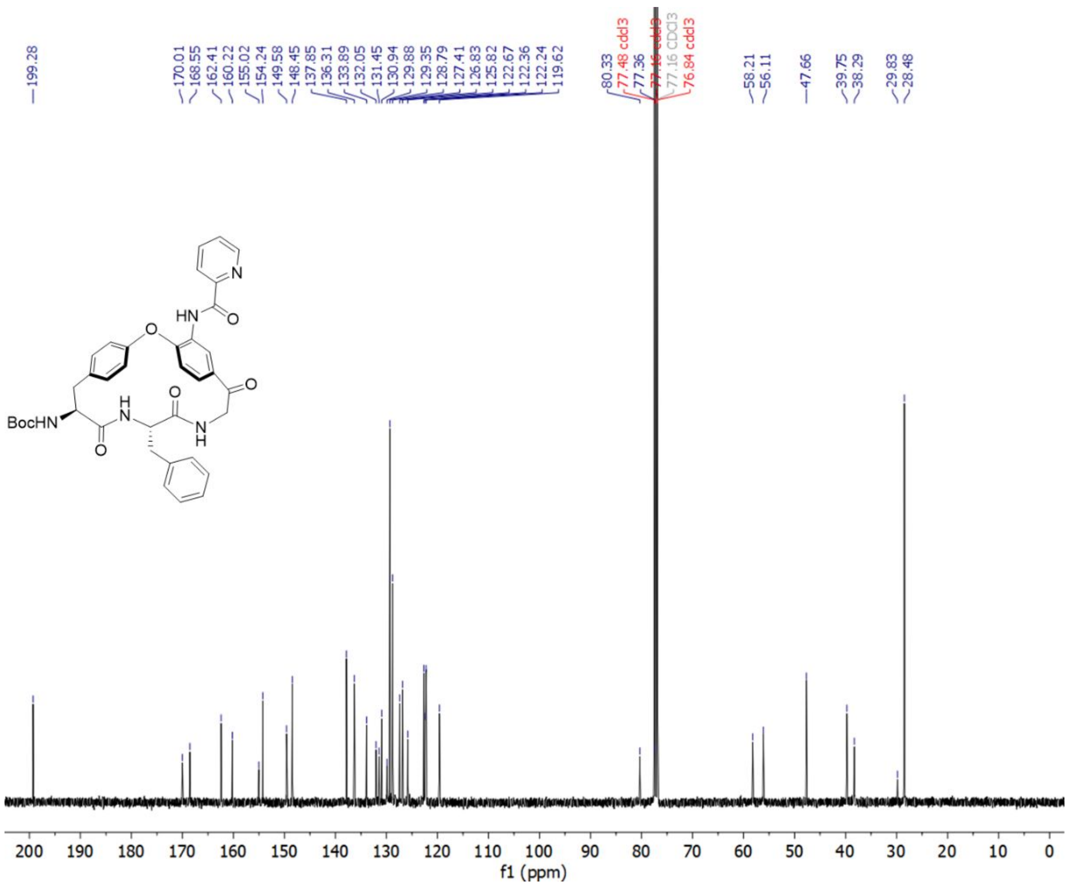

Compound 3: COSY NMR ($CDCl_3$)

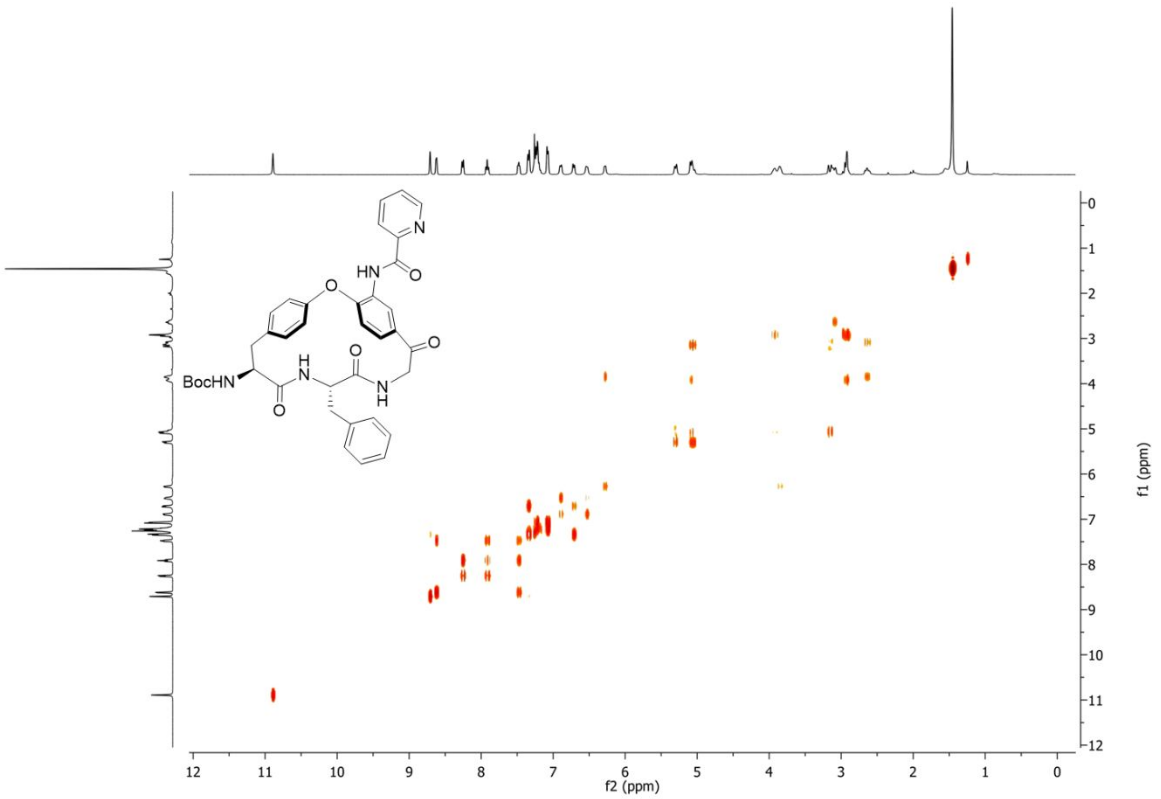

Compound 3: HSQC NMR ($CDCl_3$)

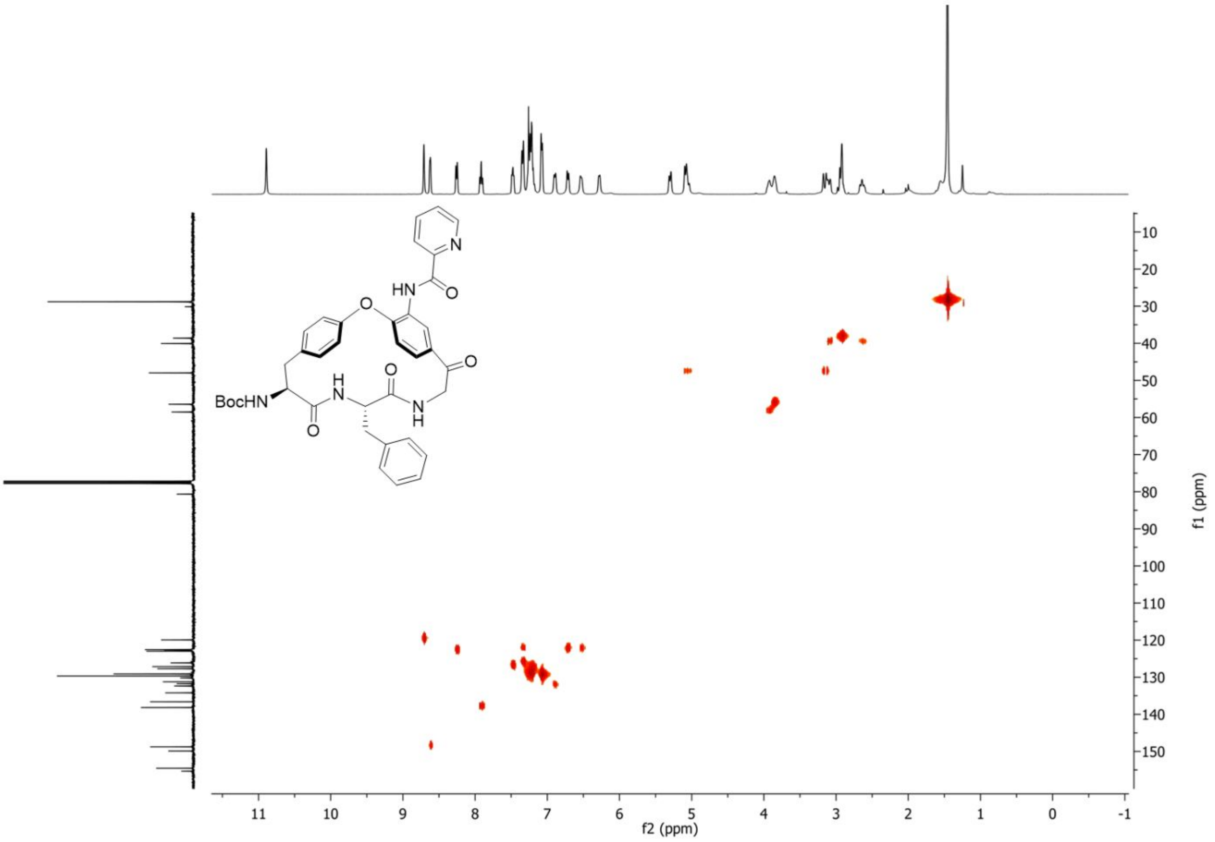

Compound 3: HMBC NMR ($CDCl_3$)

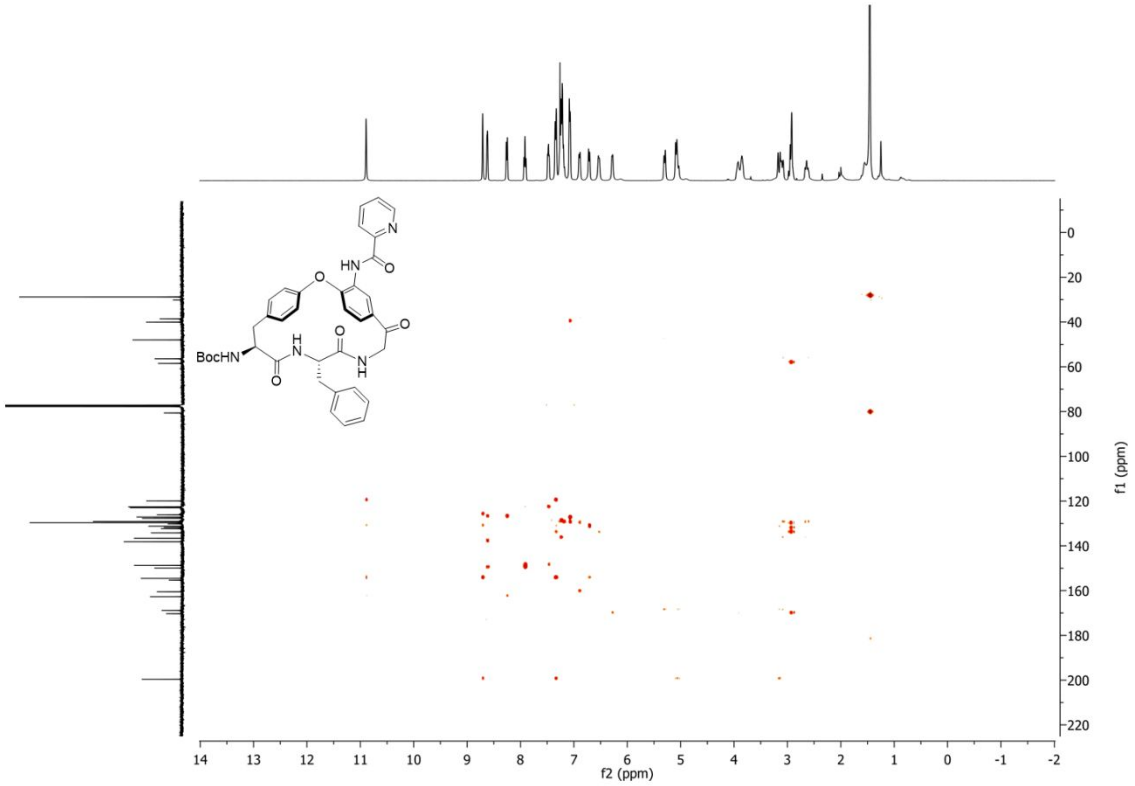

Compound 4: ^{1}H NMR (CD_3CN, 500 MHz)

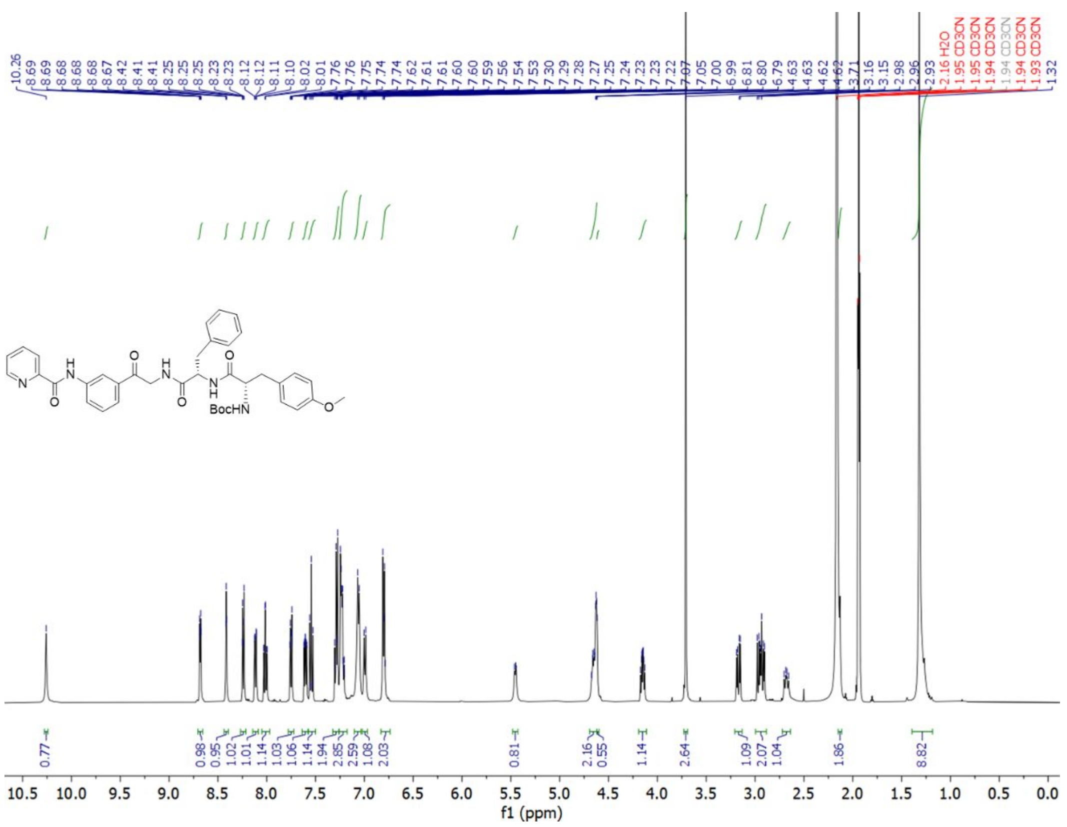

Compound 4: ^{13}C NMR (CD_3CN, 125 MHz)

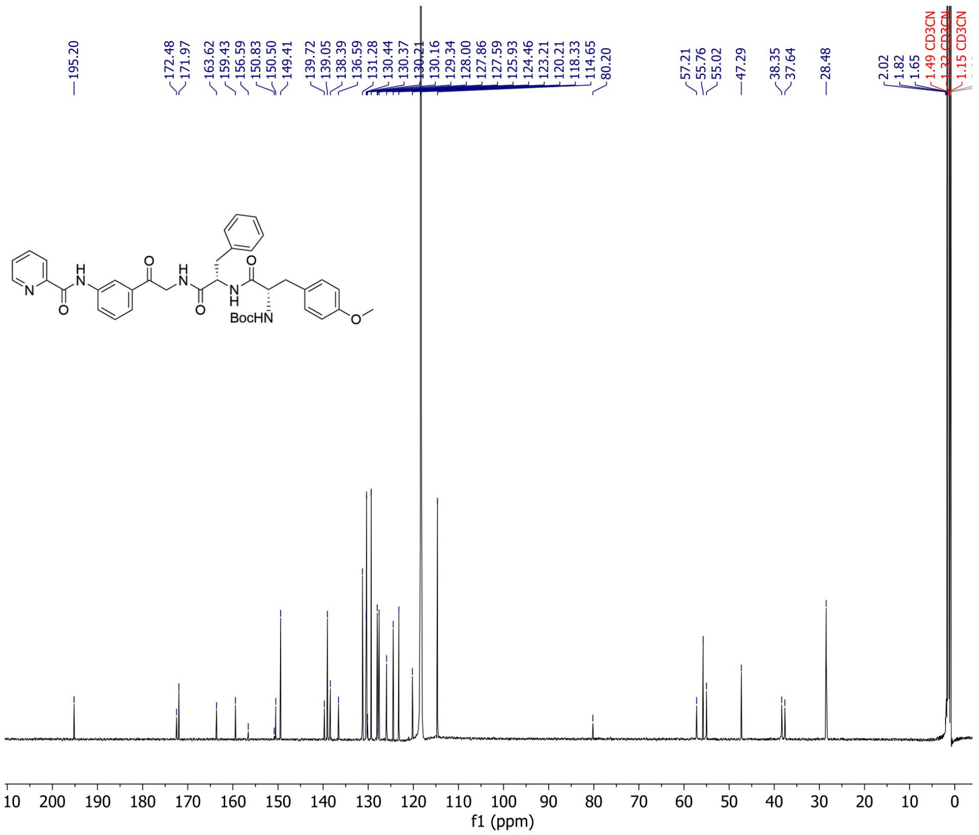

Compound 4: COSY NMR (CD_3CN)

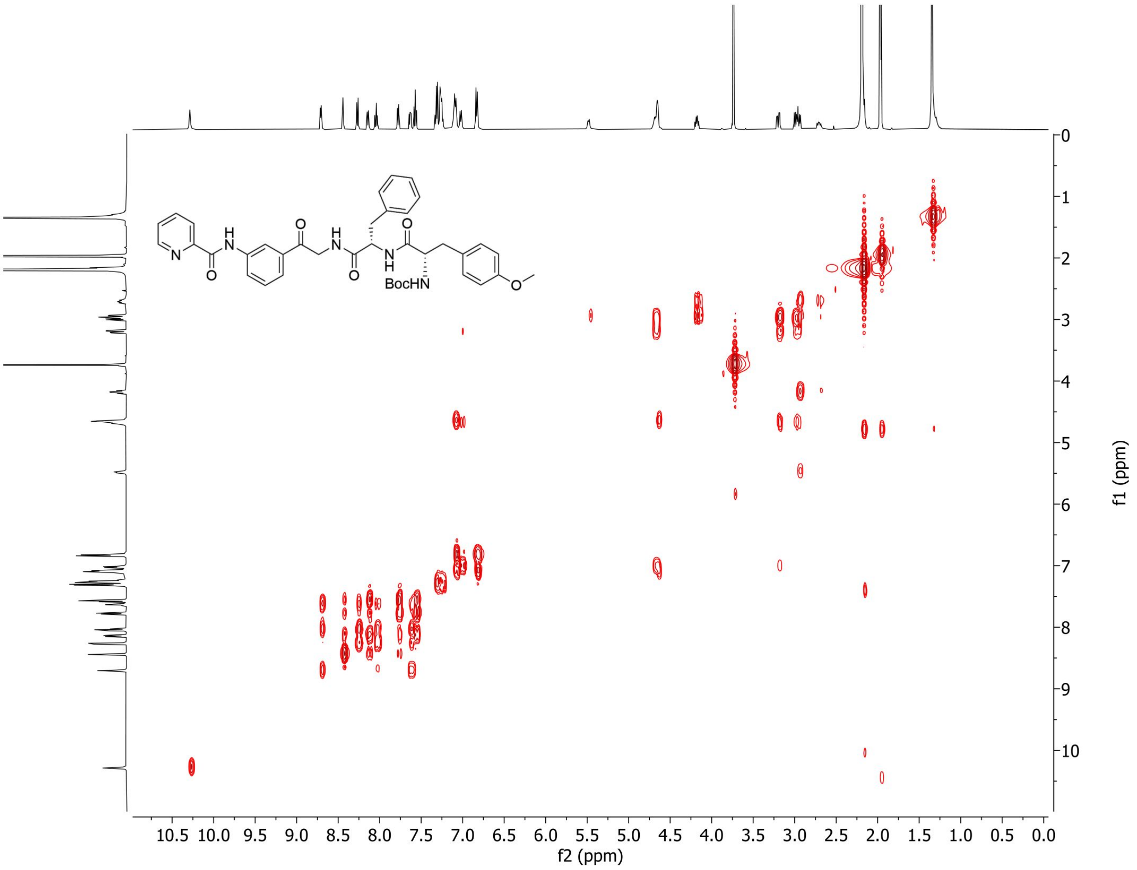

Compound 4: HSQC NMR (CD_3CN)

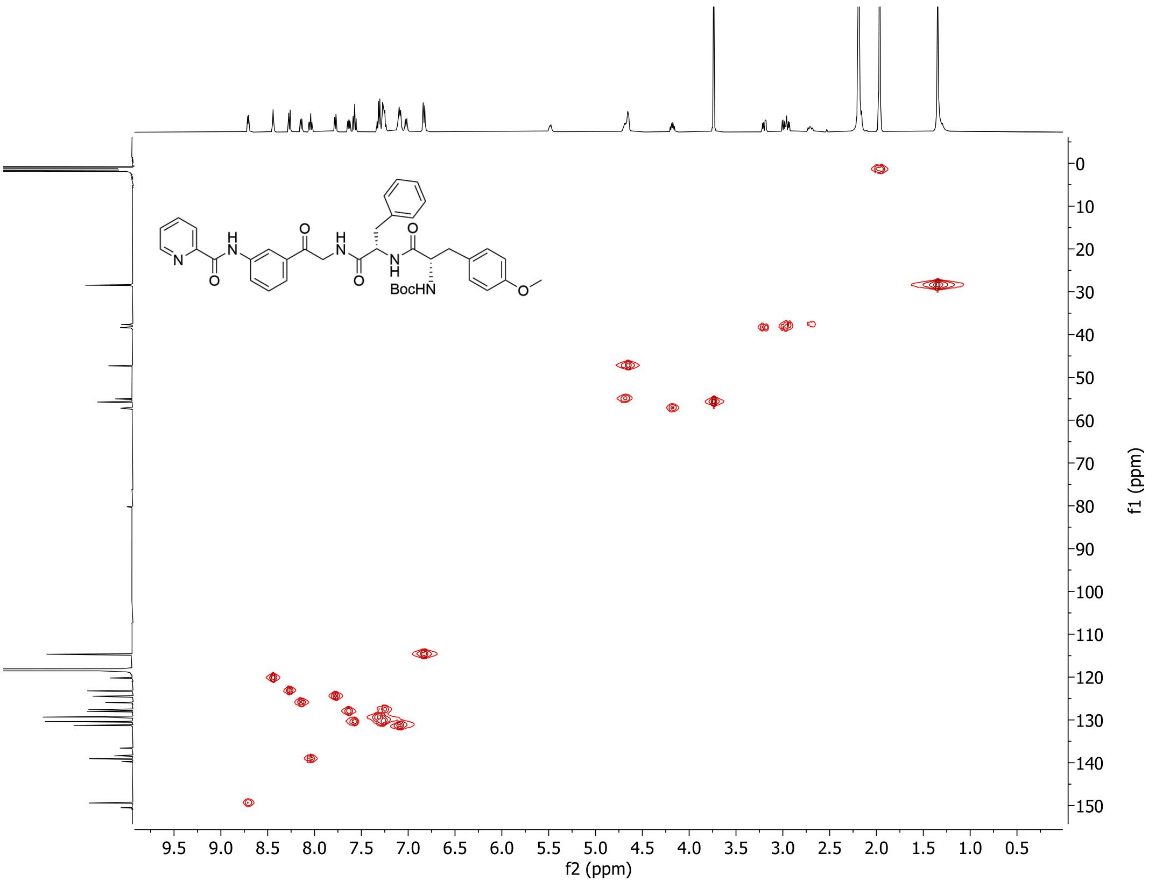

Compound 4: HMBC NMR (CD_3CN)

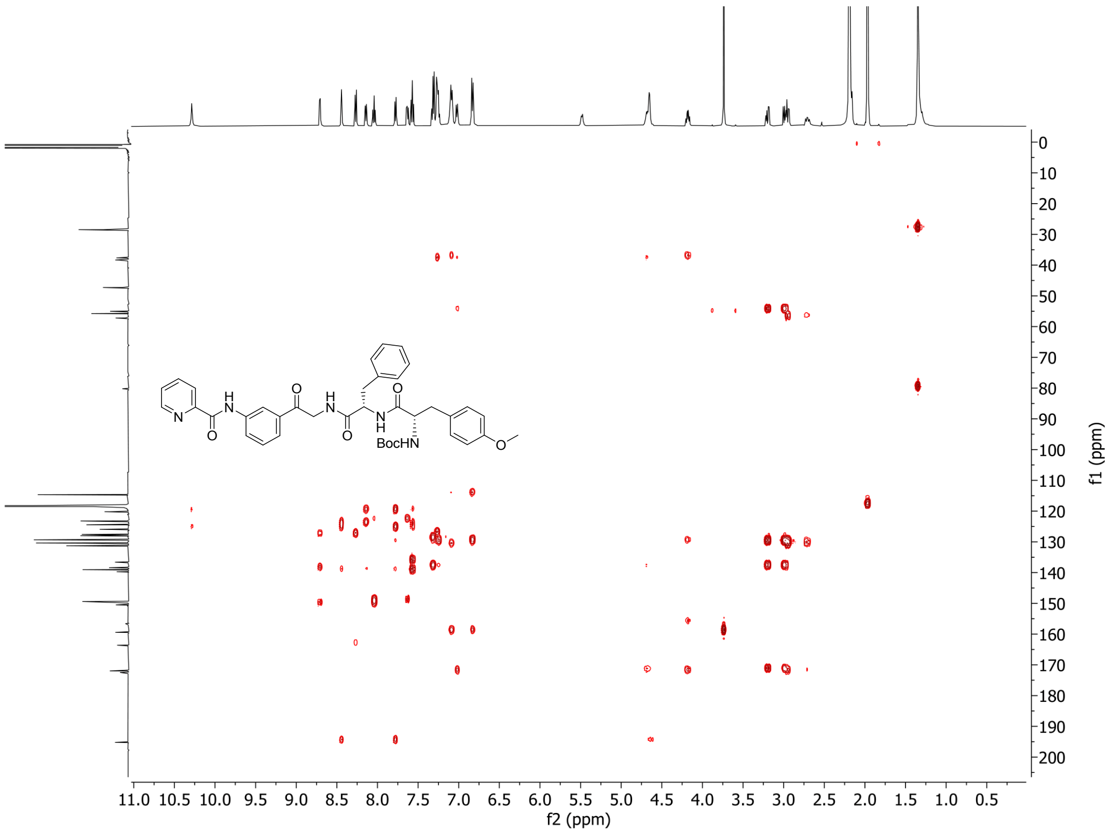

Compound 5: ^{1}H NMR ($CDCl_3$, 600 MHz)

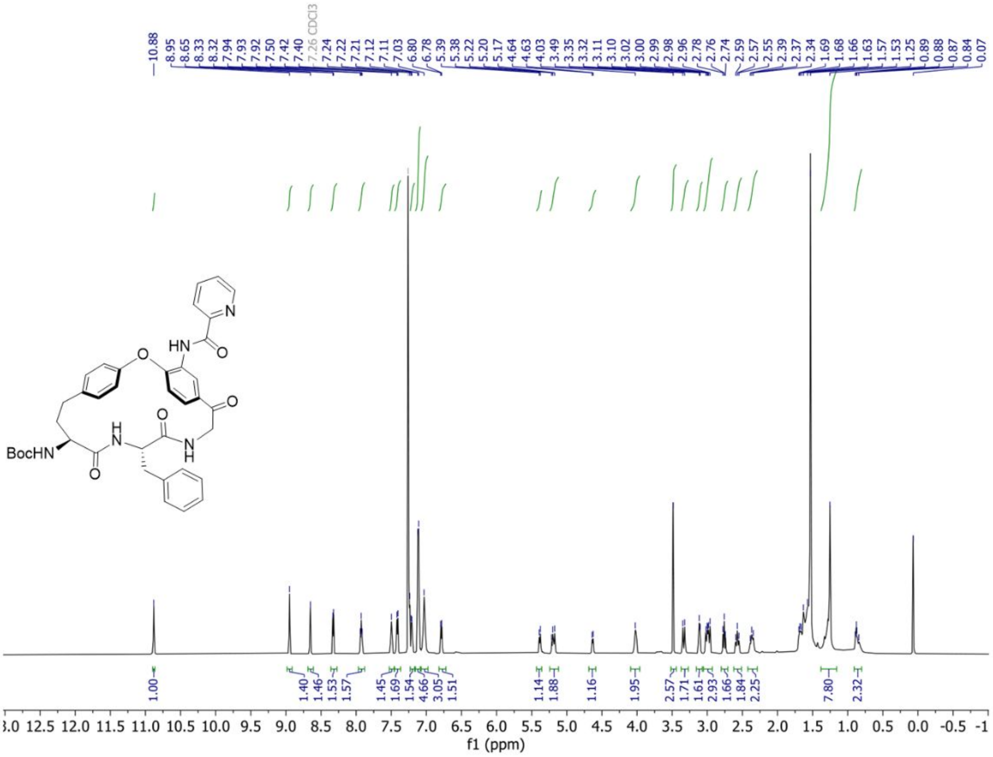

Compound 5: ^{13}C NMR ($CDCl_3$, 150 MHz)

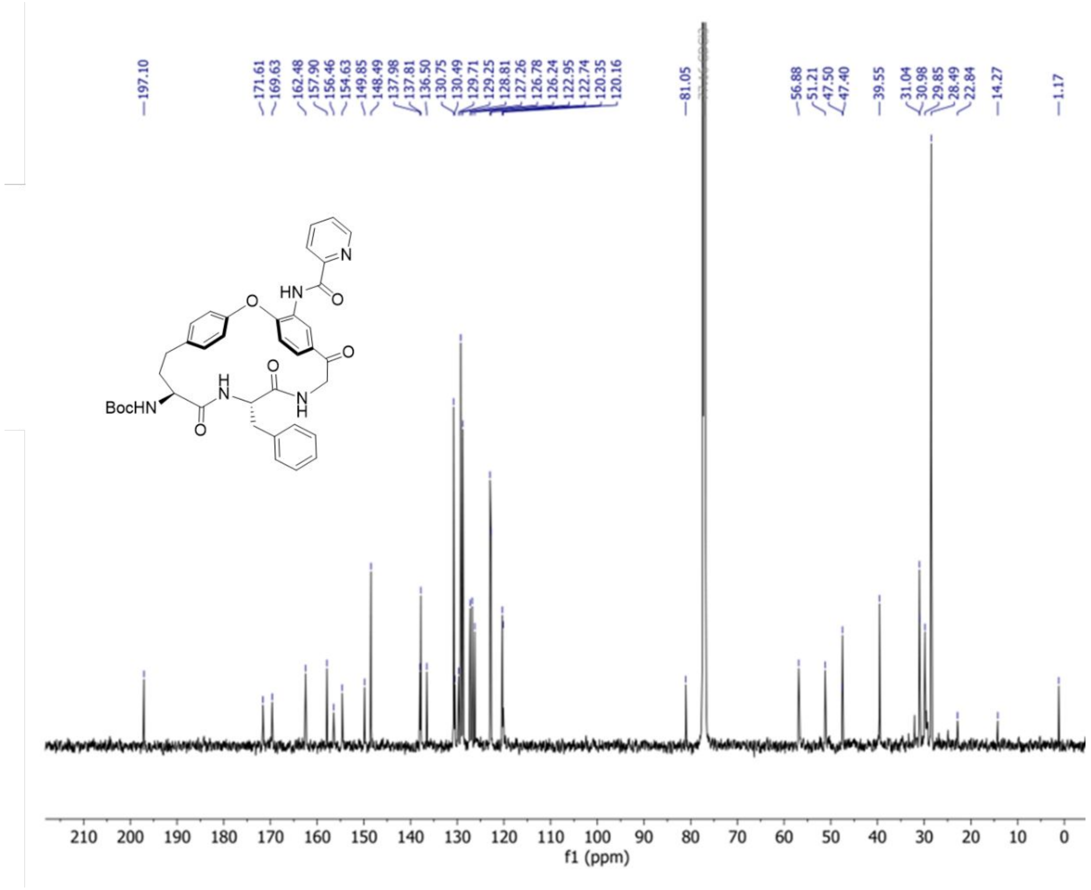

Compound 5: COSY NMR ($CDCl_3$)

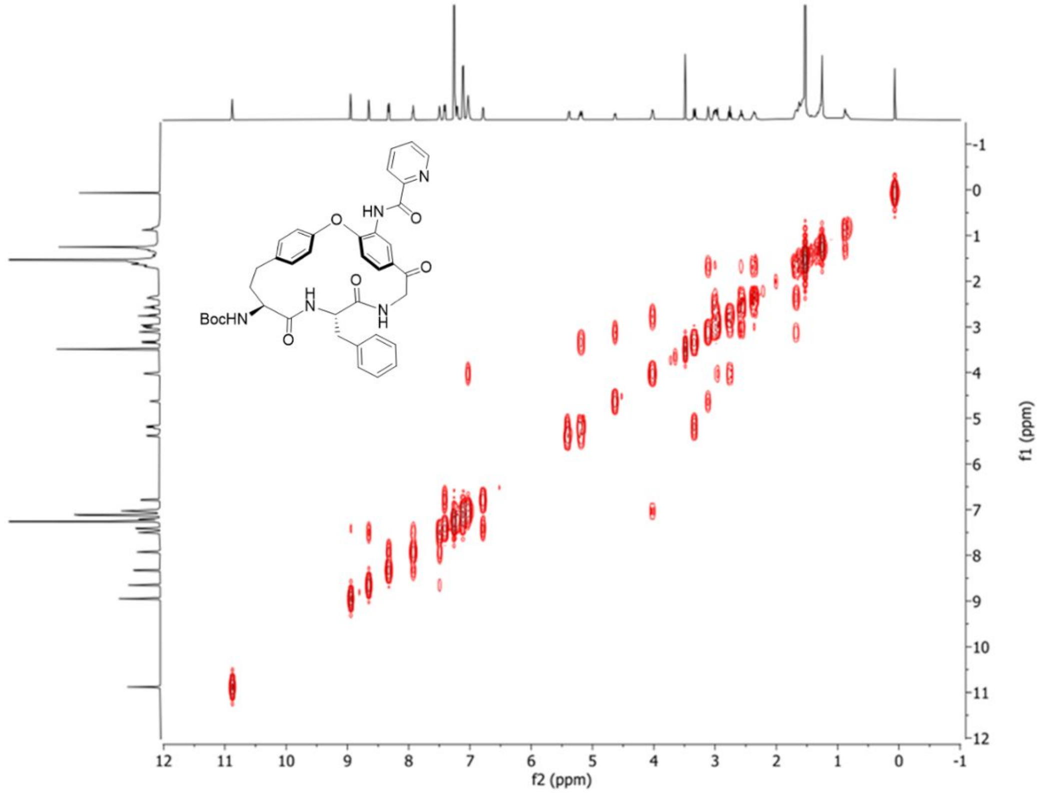

Compound 5: HSQC NMR ($CDCl_3$)

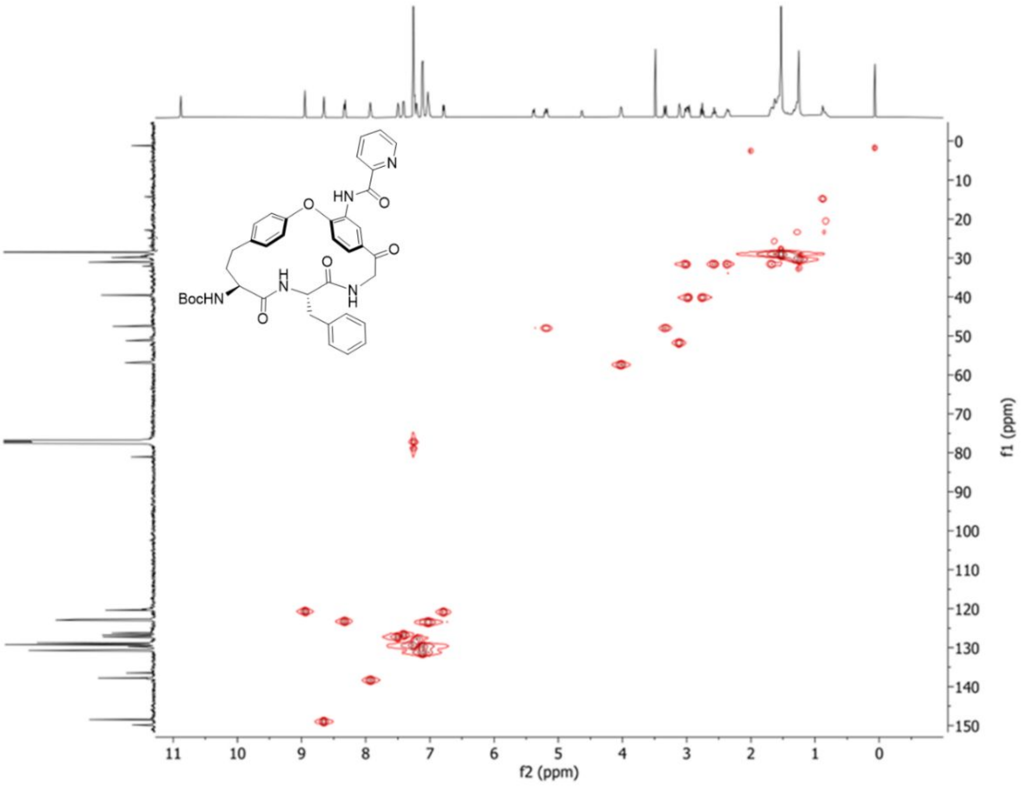

Compound 5: HMBC NMR ($CDCl_3$)

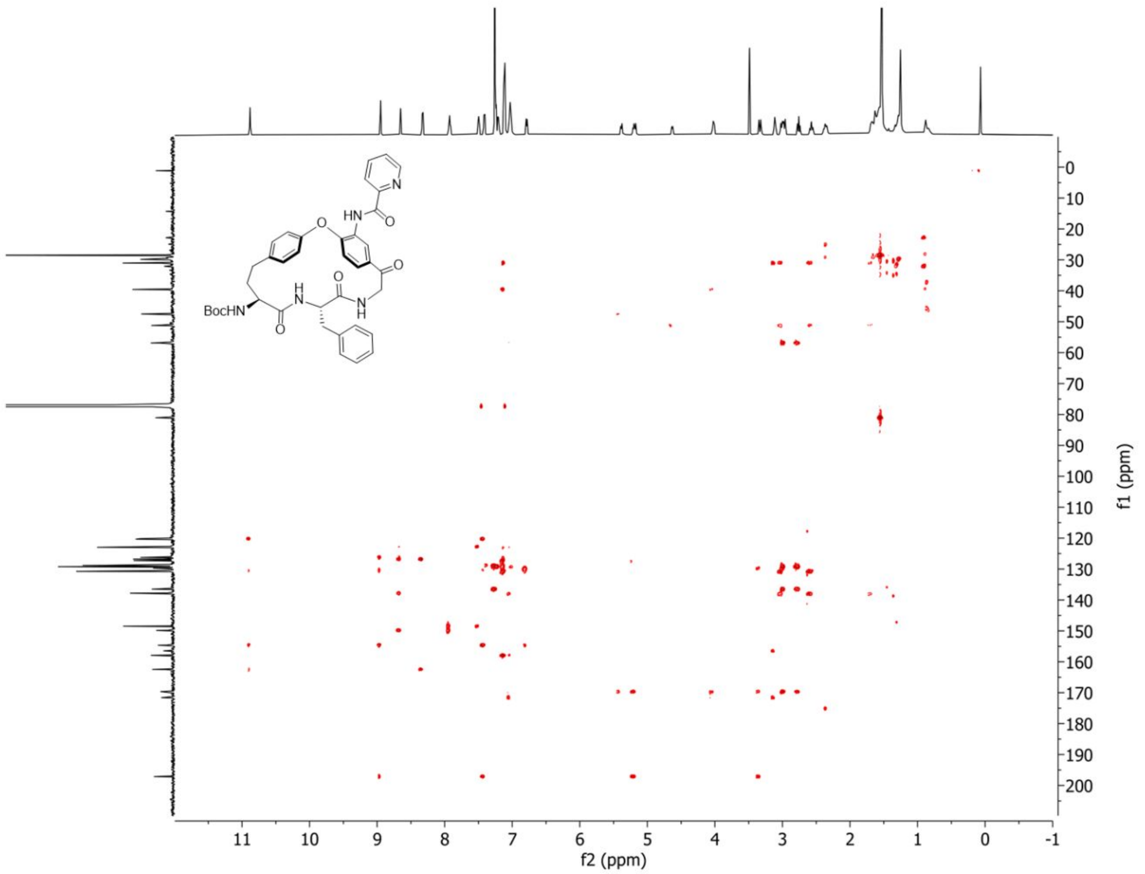

Compound 6: ^{1}H NMR (CD_3CN, 400 MHz)

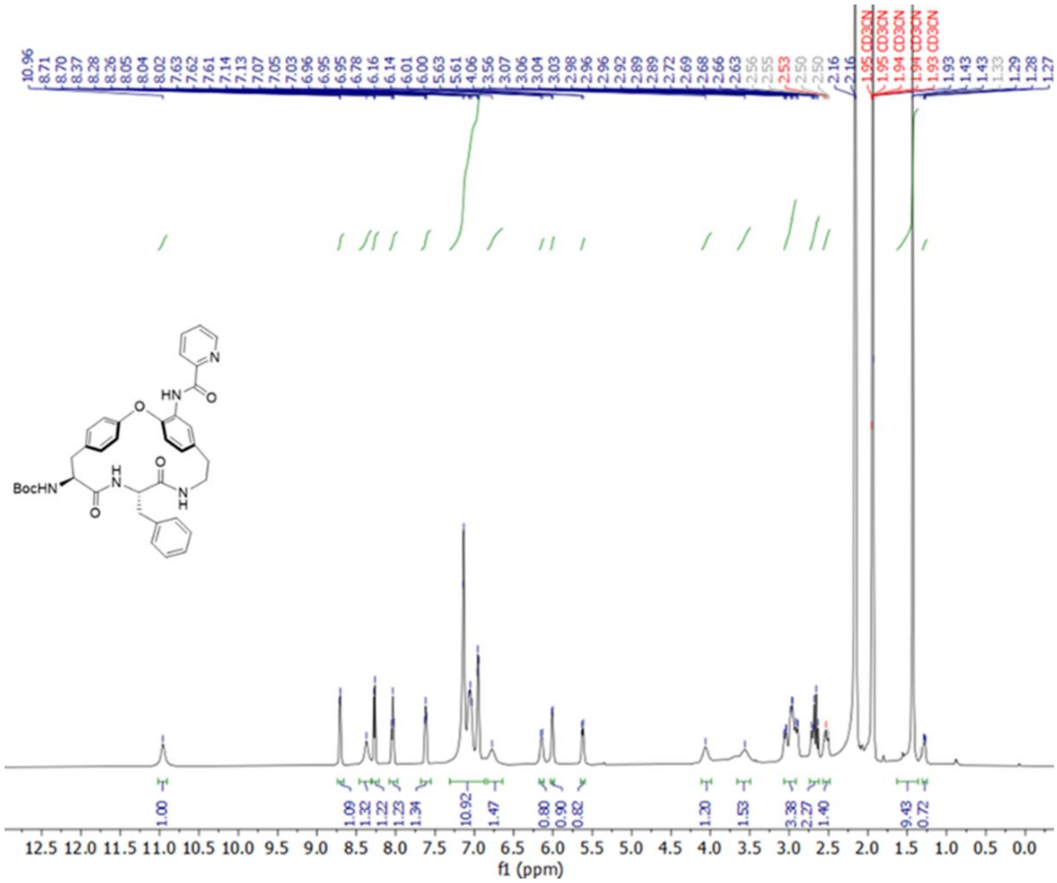

Compound 6: ^{13}C NMR (CD_3CN, 125 MHz)

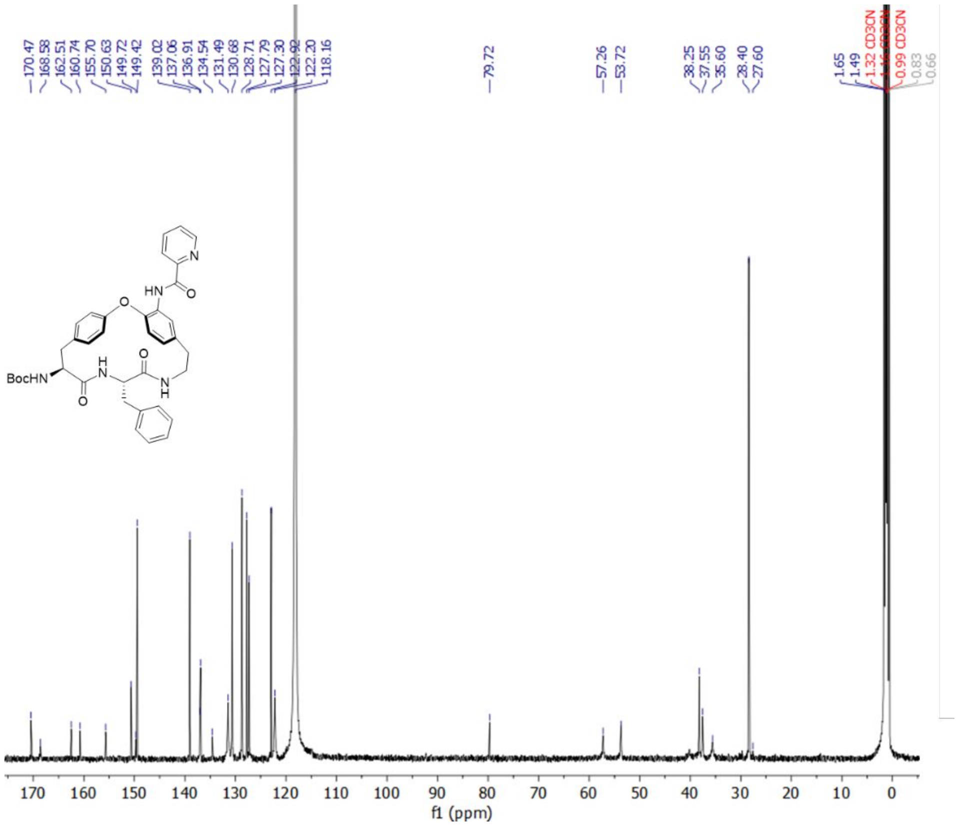

Compound 6: COSY NMR (CD_3CN)

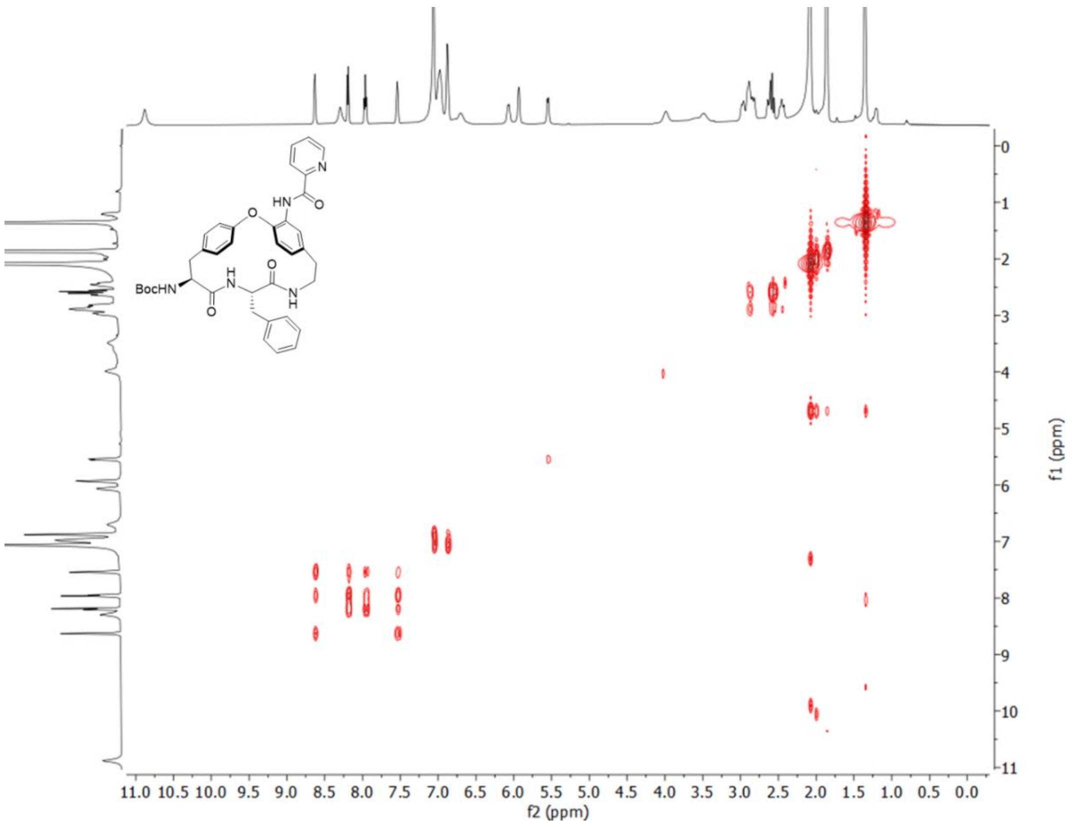

Compound 6: HSQC NMR (CD_3CN)

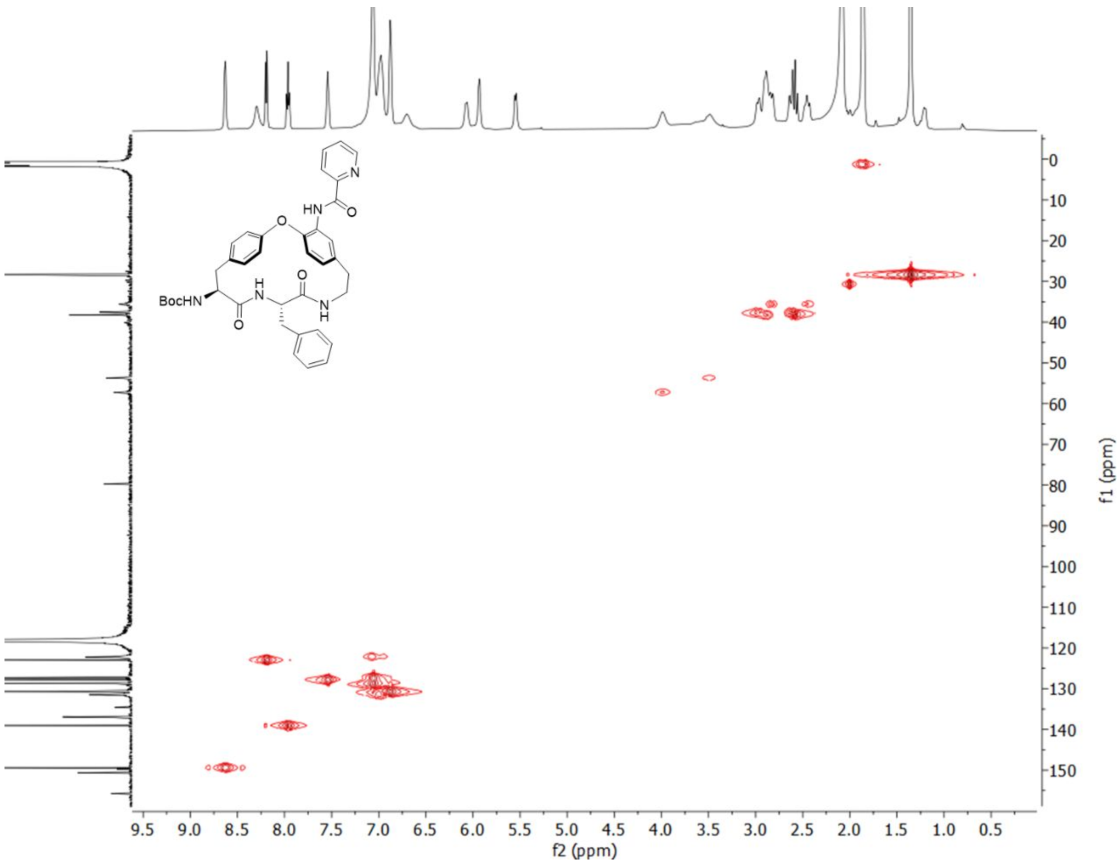

Compound 6: HMBC NMR (CD_3CN)

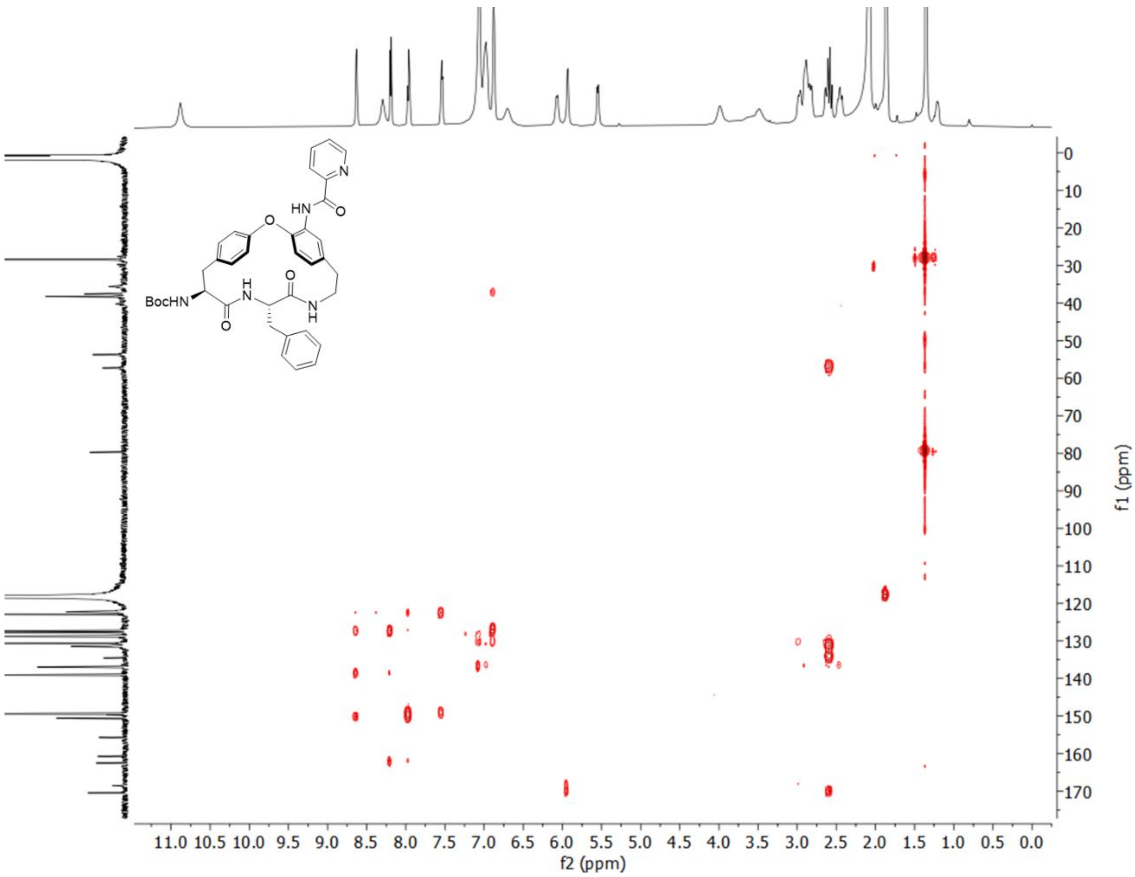

Compound 7: ^{1}H NMR (CD_3CN, 500 MHz)

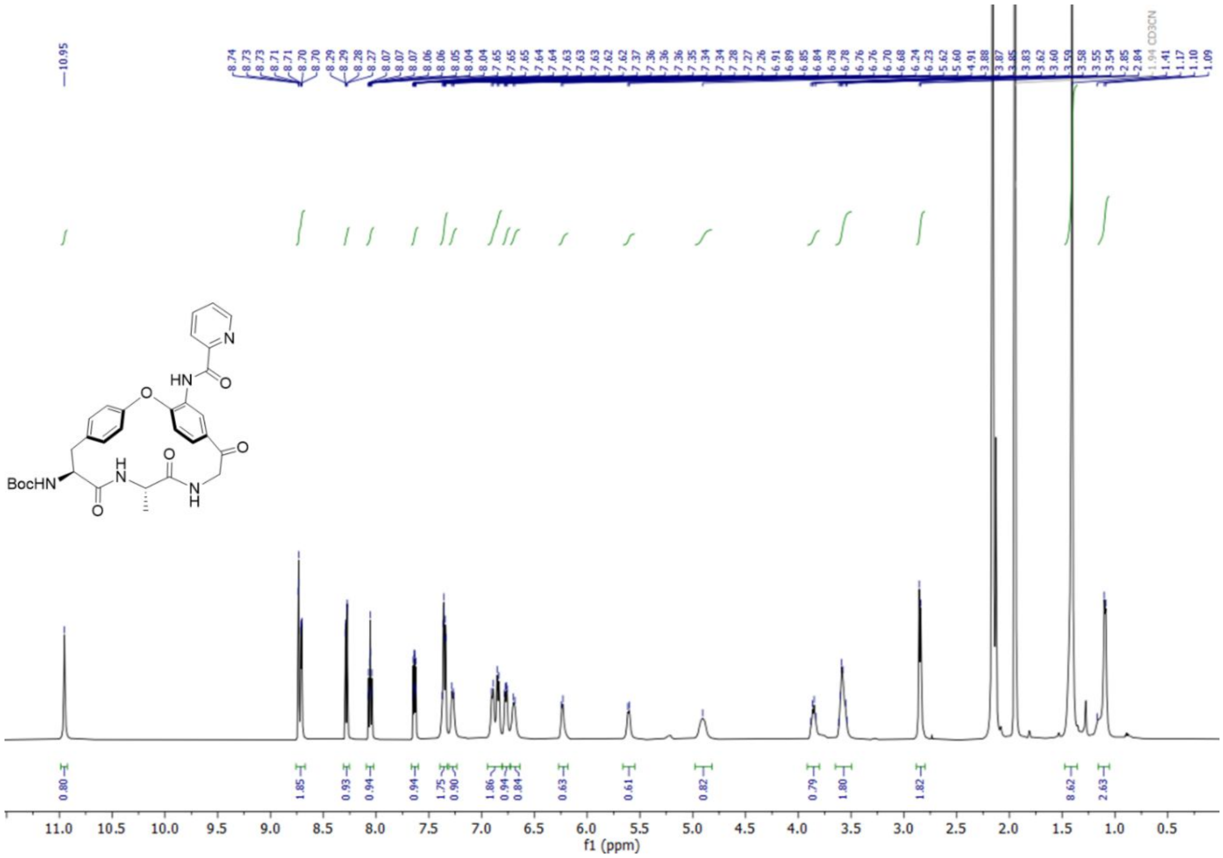

Compound 7: ^{13}C NMR (CD_3CN, 125 MHz)

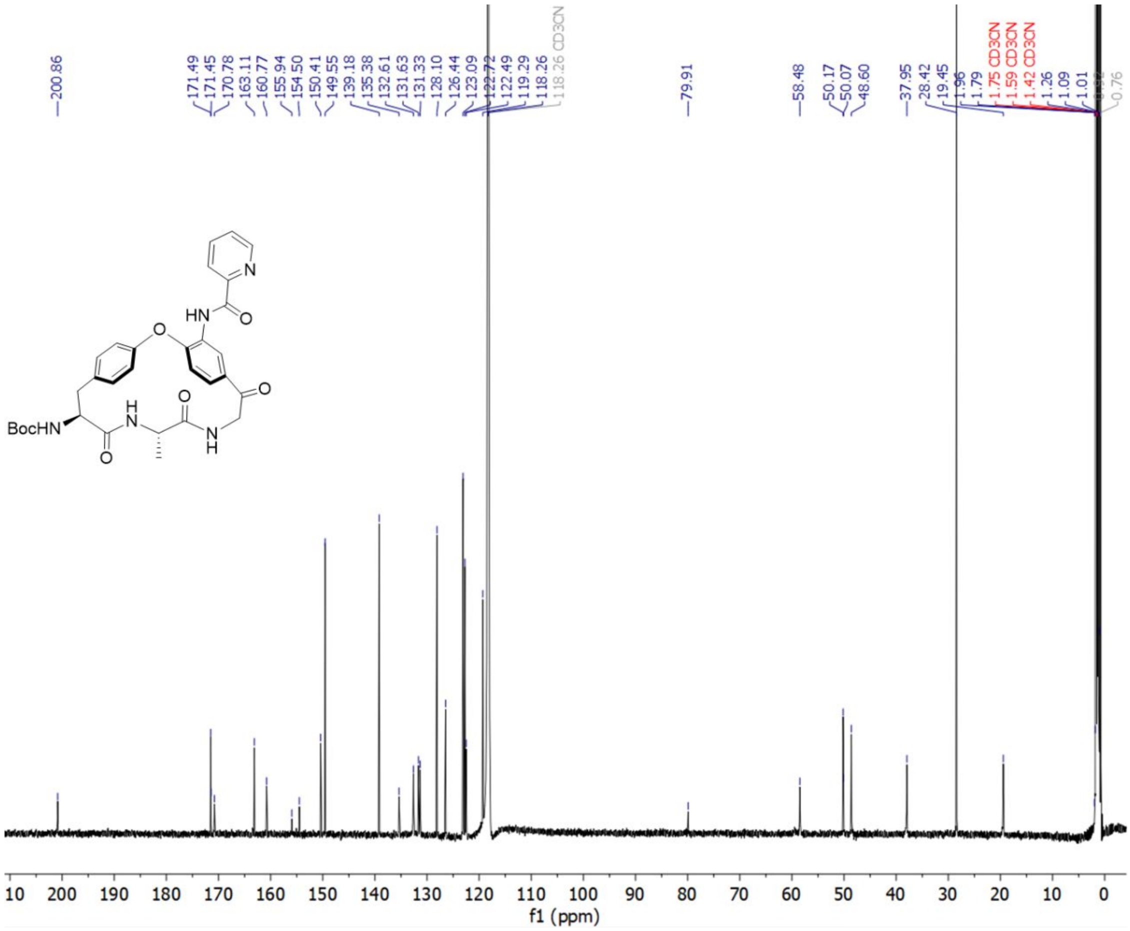

Compound 7: COSY NMR (CD_3CN)

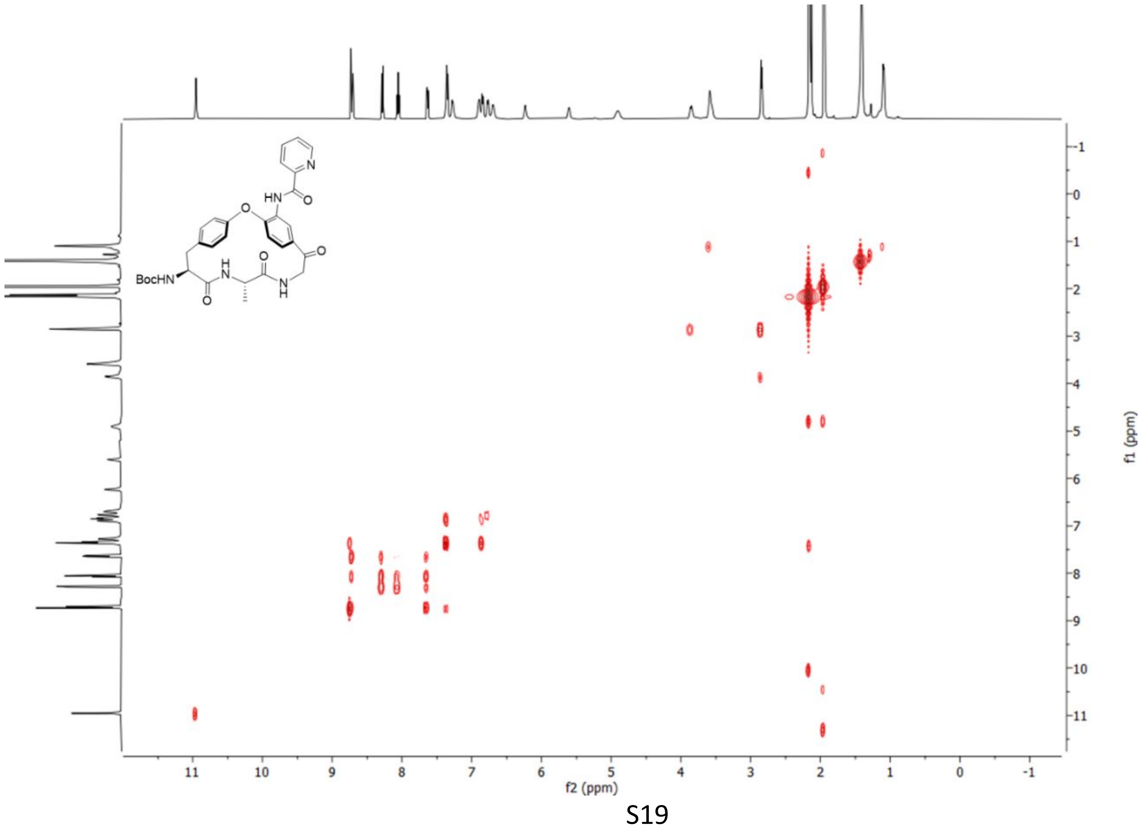

Compound 7: HSQC NMR (CD_3CN)

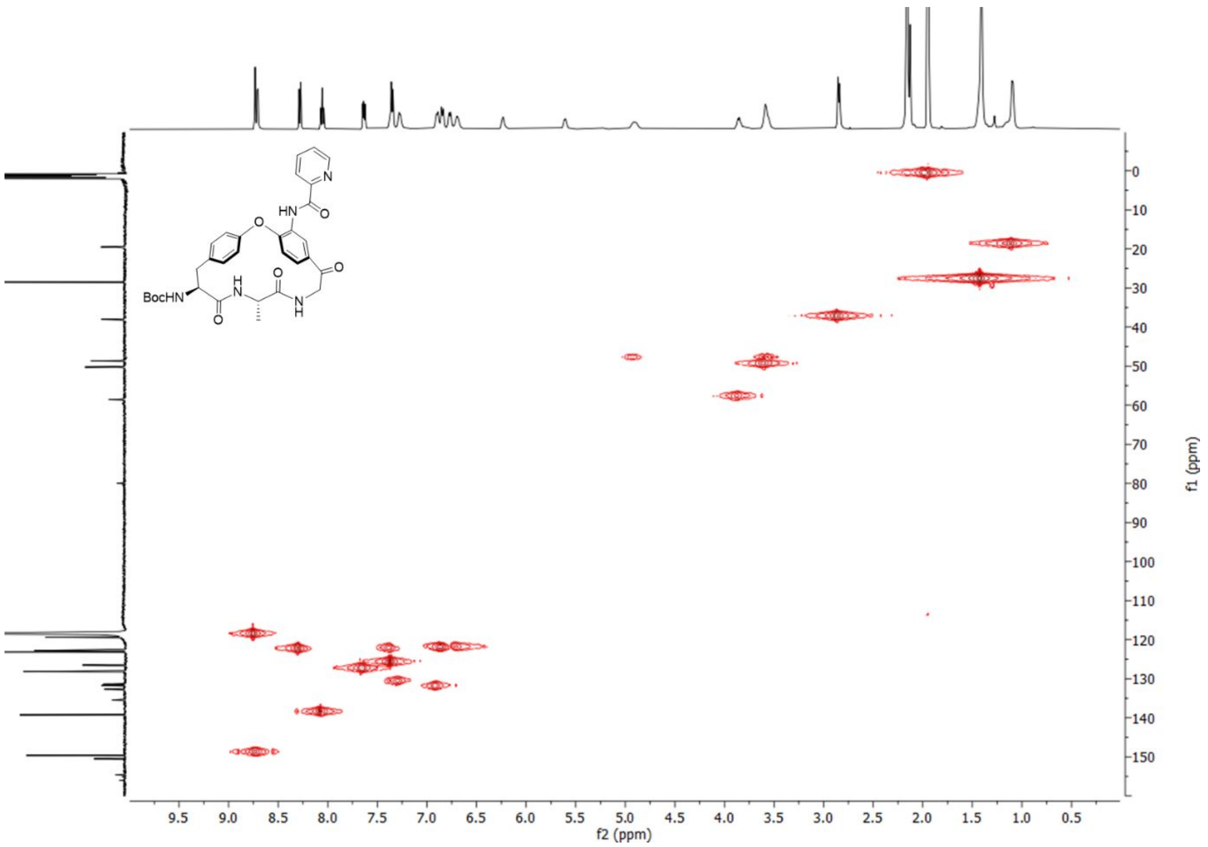

Compound 7: HMBC NMR (CD_3CN)

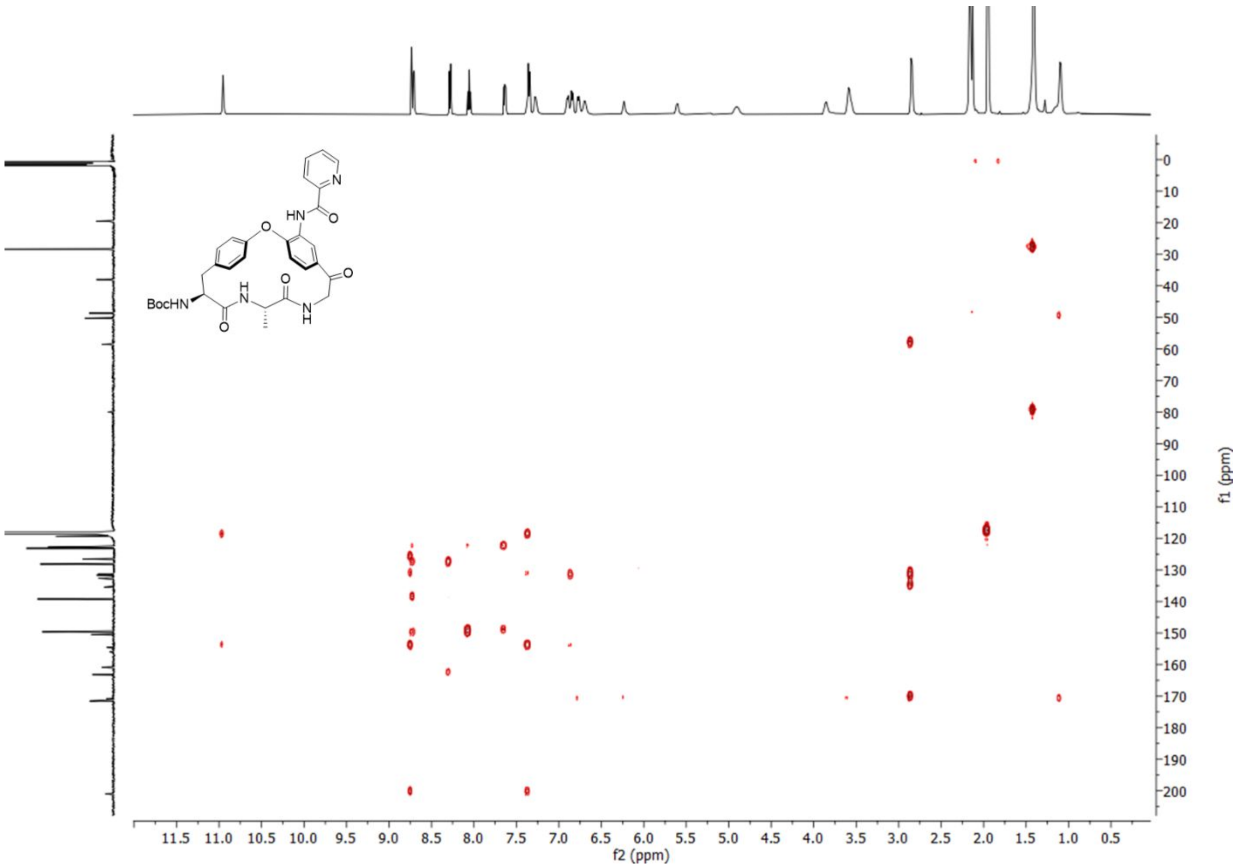

Compound 8: ^{1}H NMR (CD_3CN, 500 MHz)

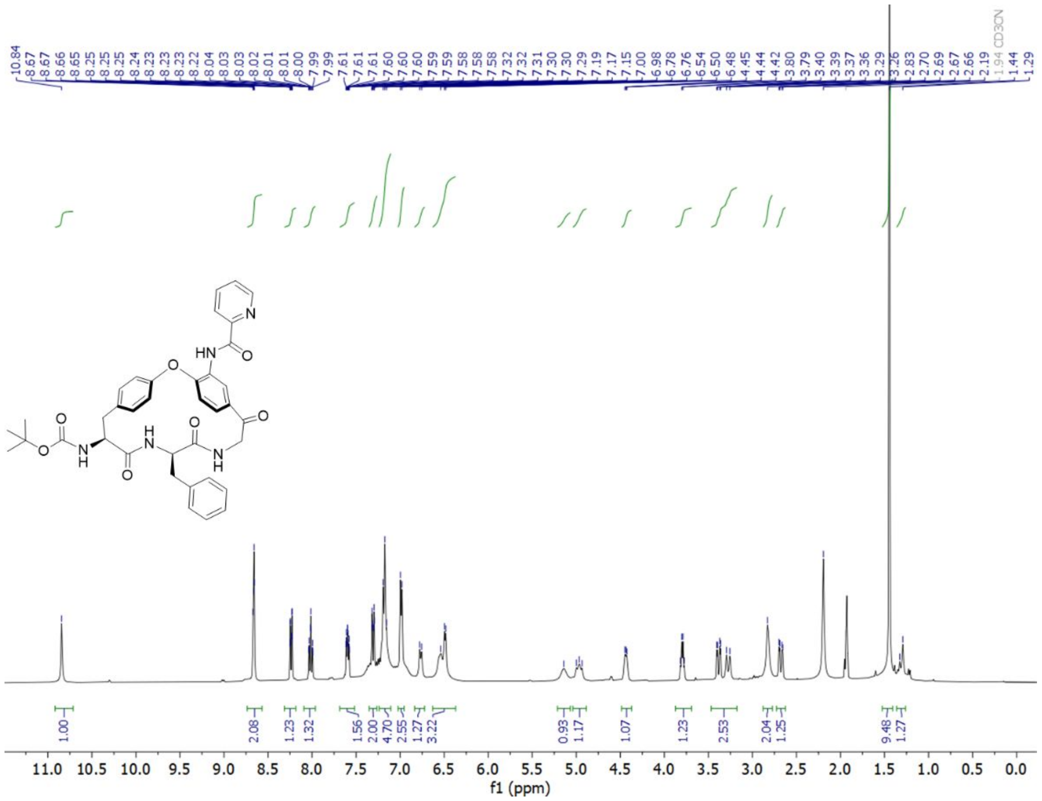

Compound 8: ^{13}C NMR (CD_3CN, 125 MHz)

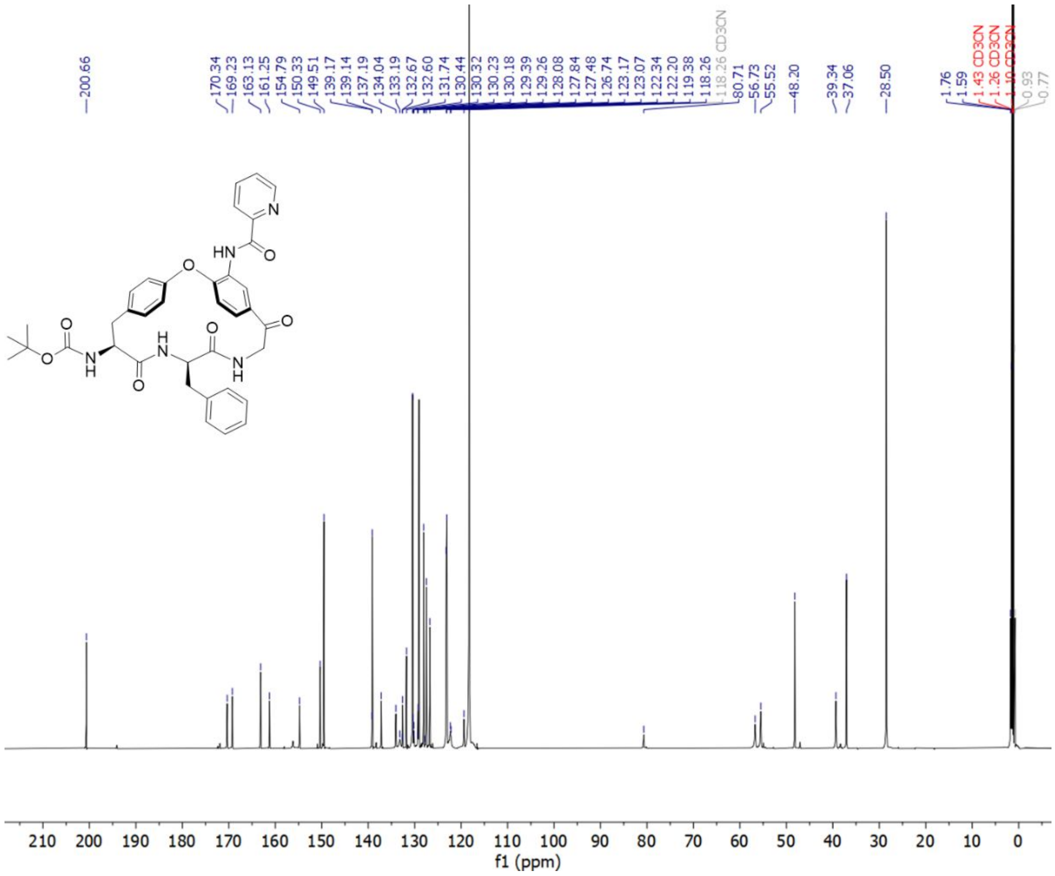

Compound 8: COSY NMR (CD_3CN)

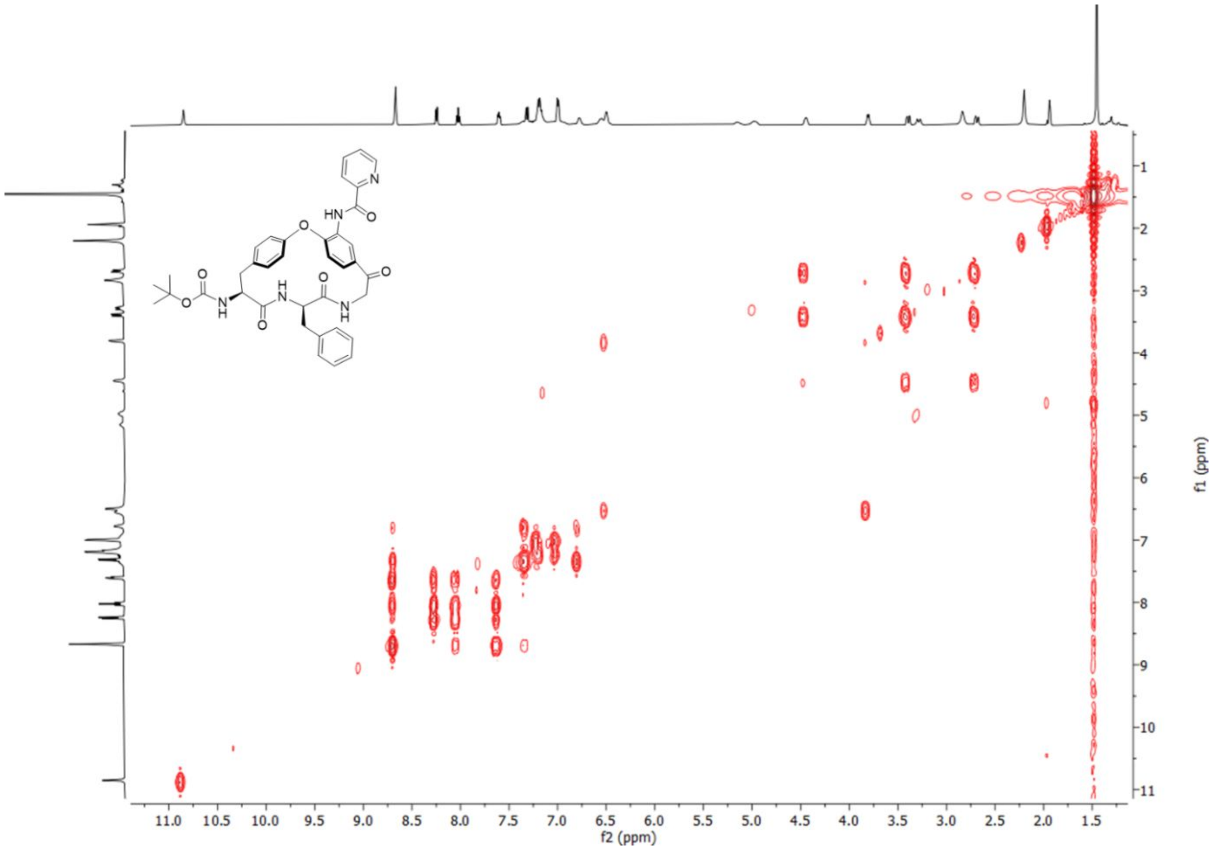

Compound 8: HSQC NMR (CD_3CN)

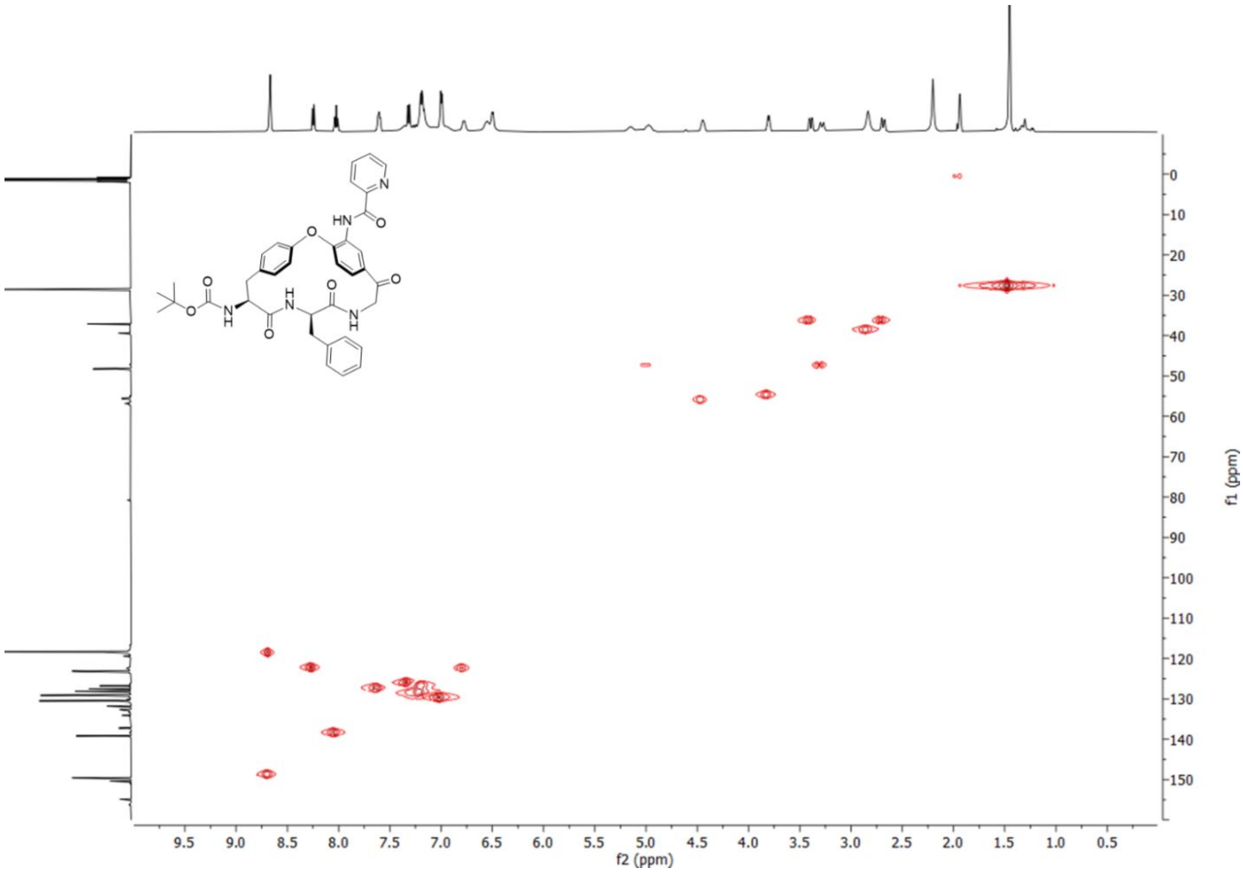

Compound 8: HMBC NMR (CD_3CN)

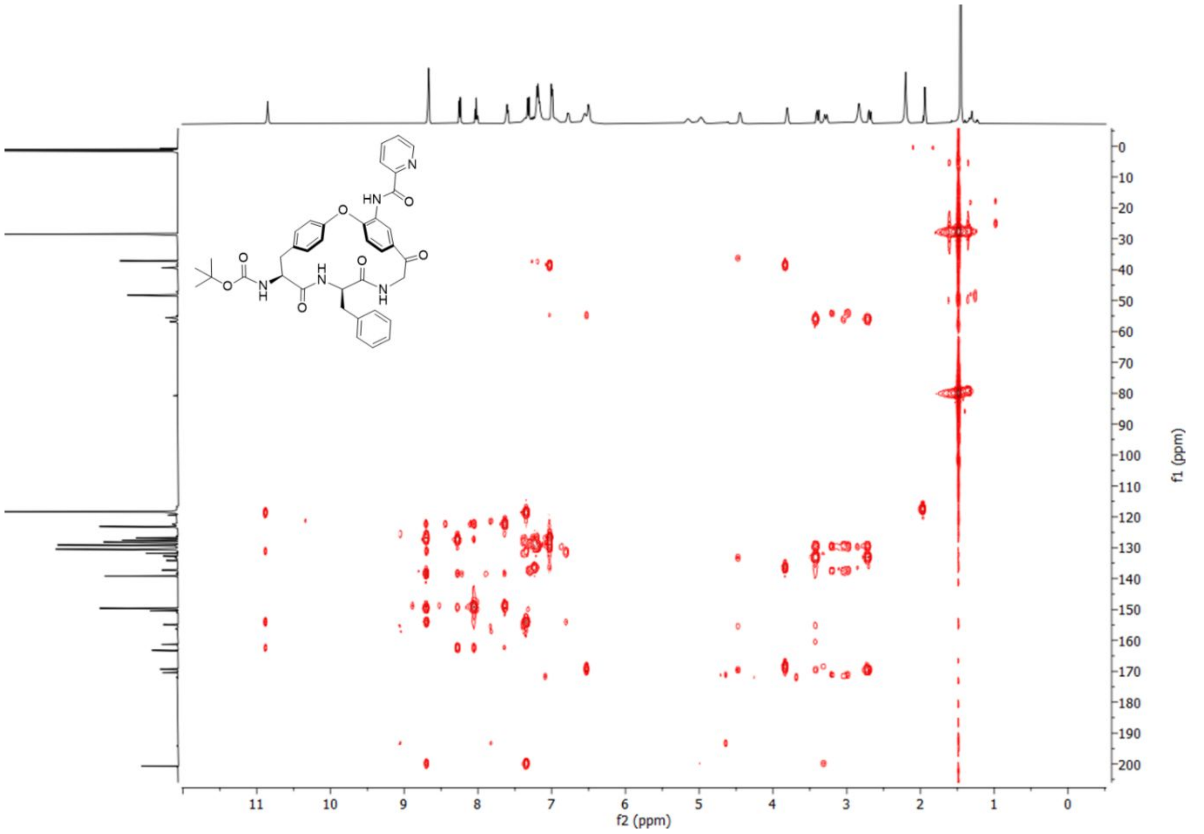

Compound 9: ^1H NMR (CD_3CN, 500 MHz)

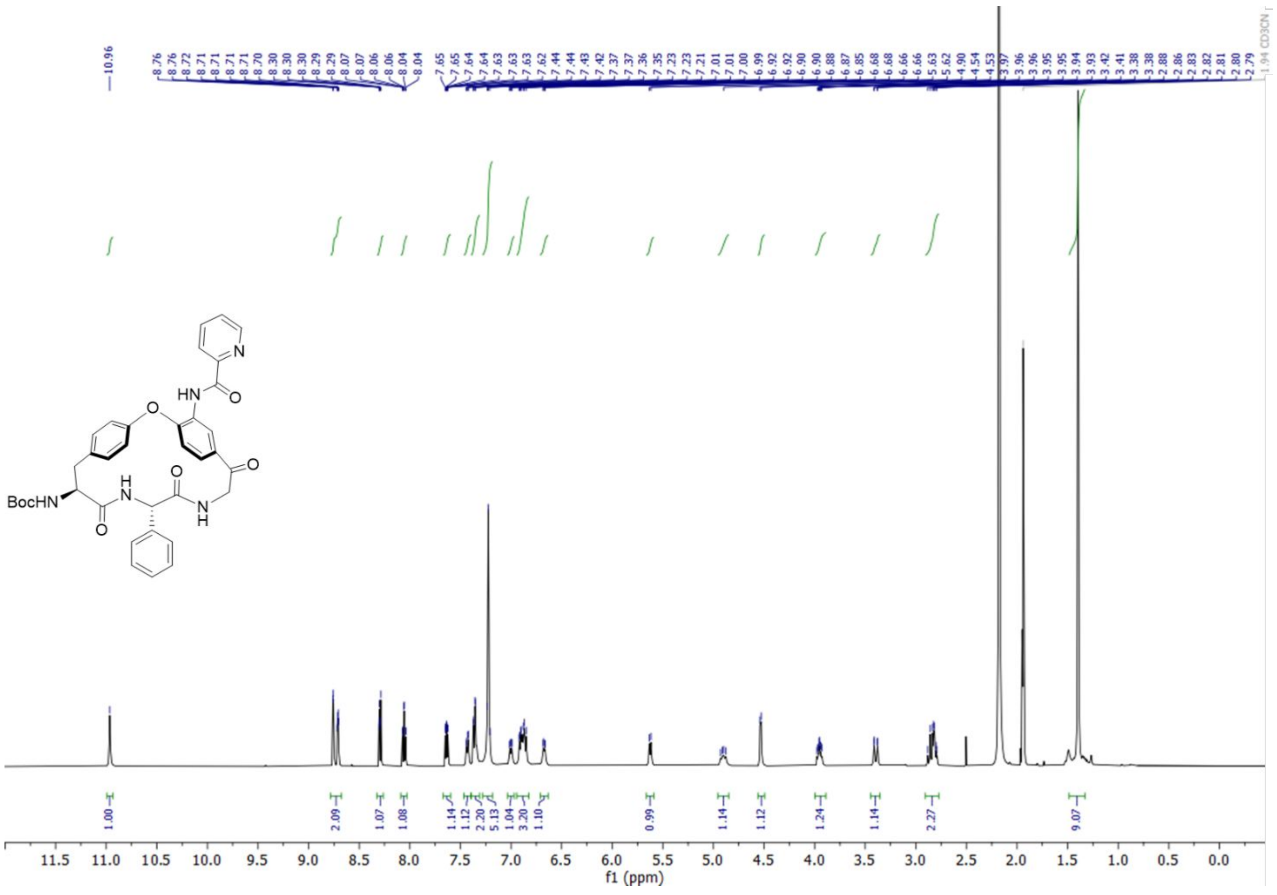

Compound 9: ^{13}C NMR (CD_3CN, 125 MHz)

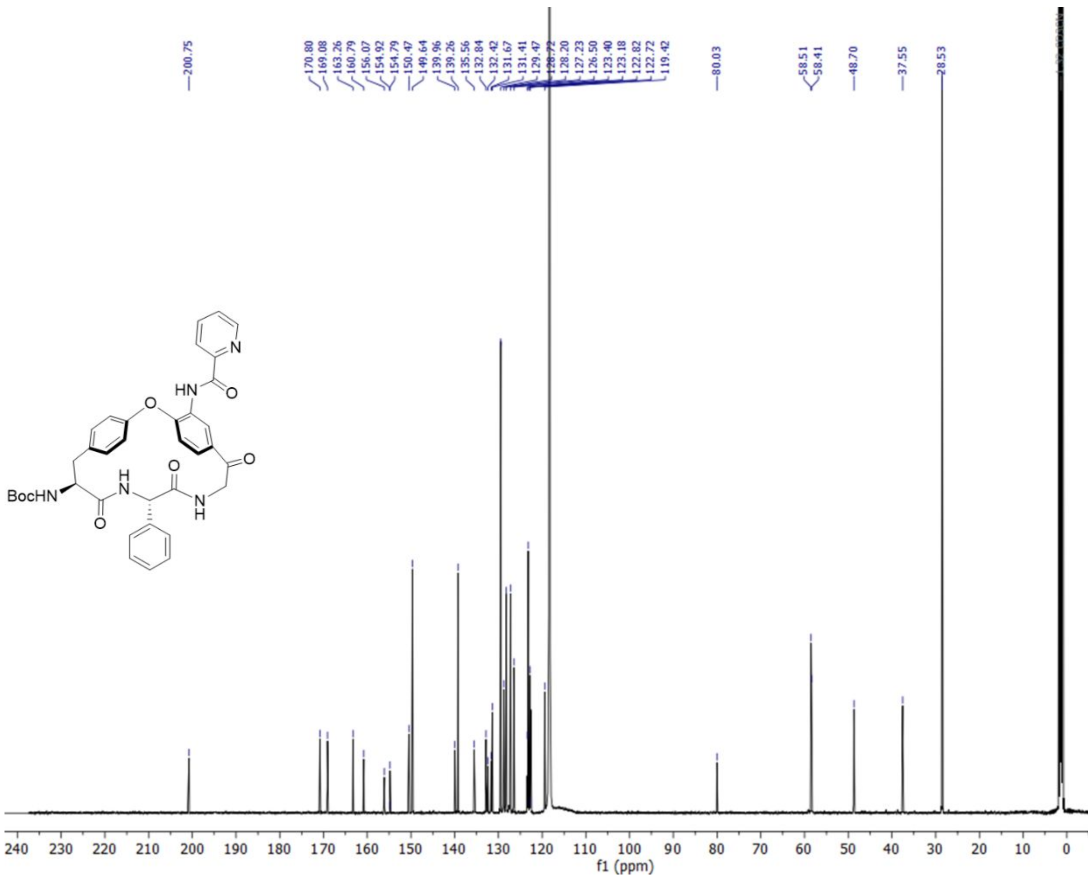

Compound 9: COSY NMR (CD_3CN)

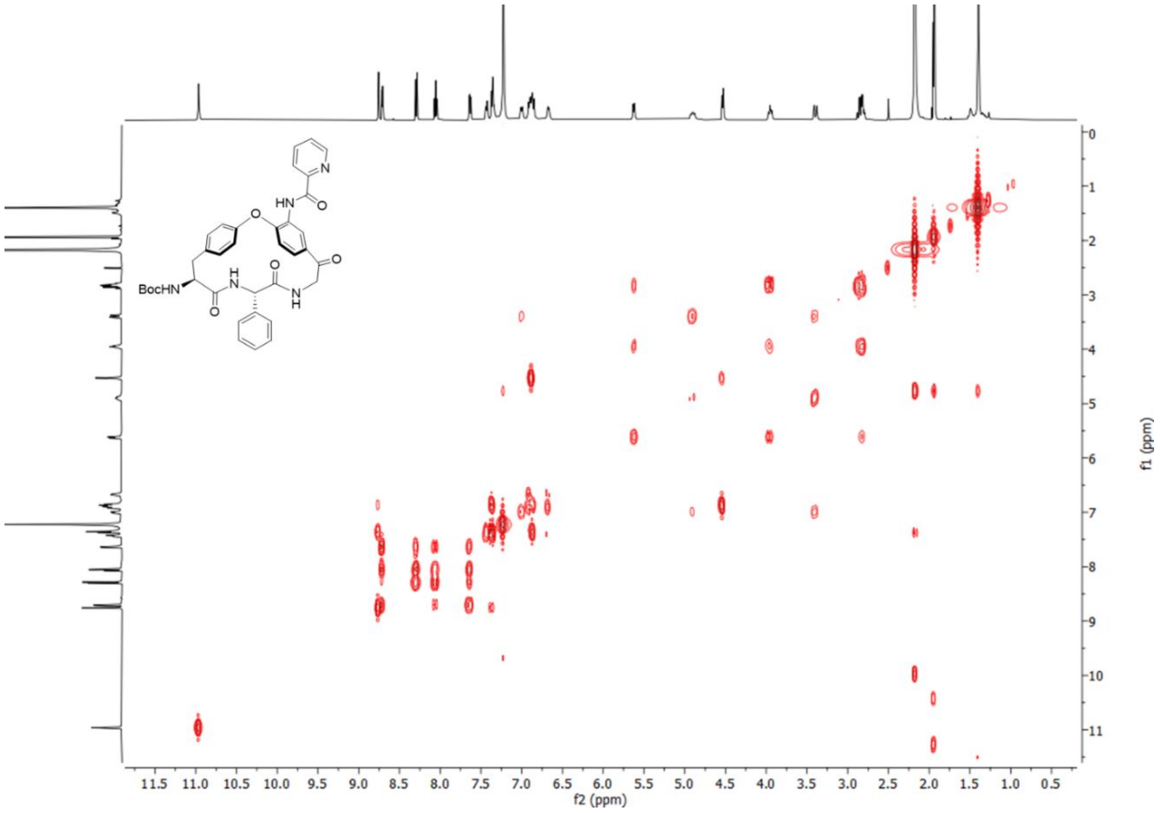

Compound 9: HSQC NMR (CD_3CN)

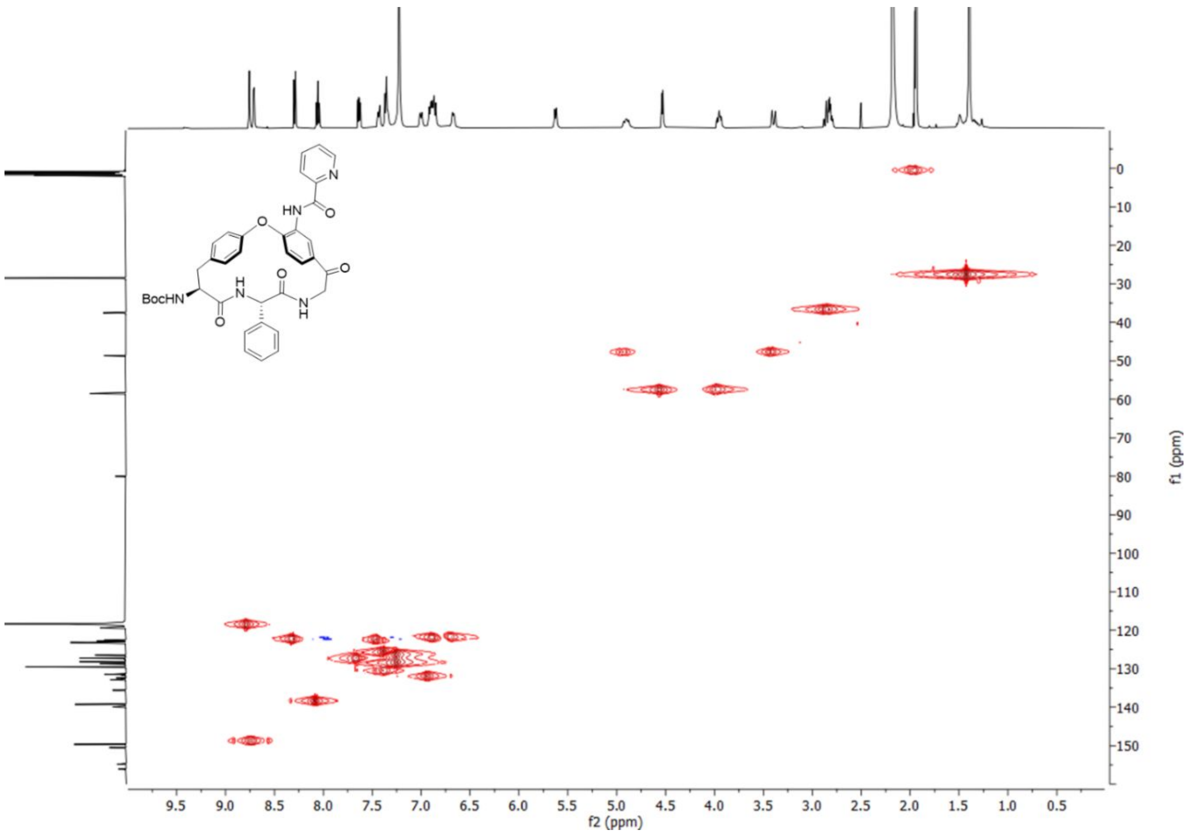

Compound 9: HMBC NMR (CD_3CN)

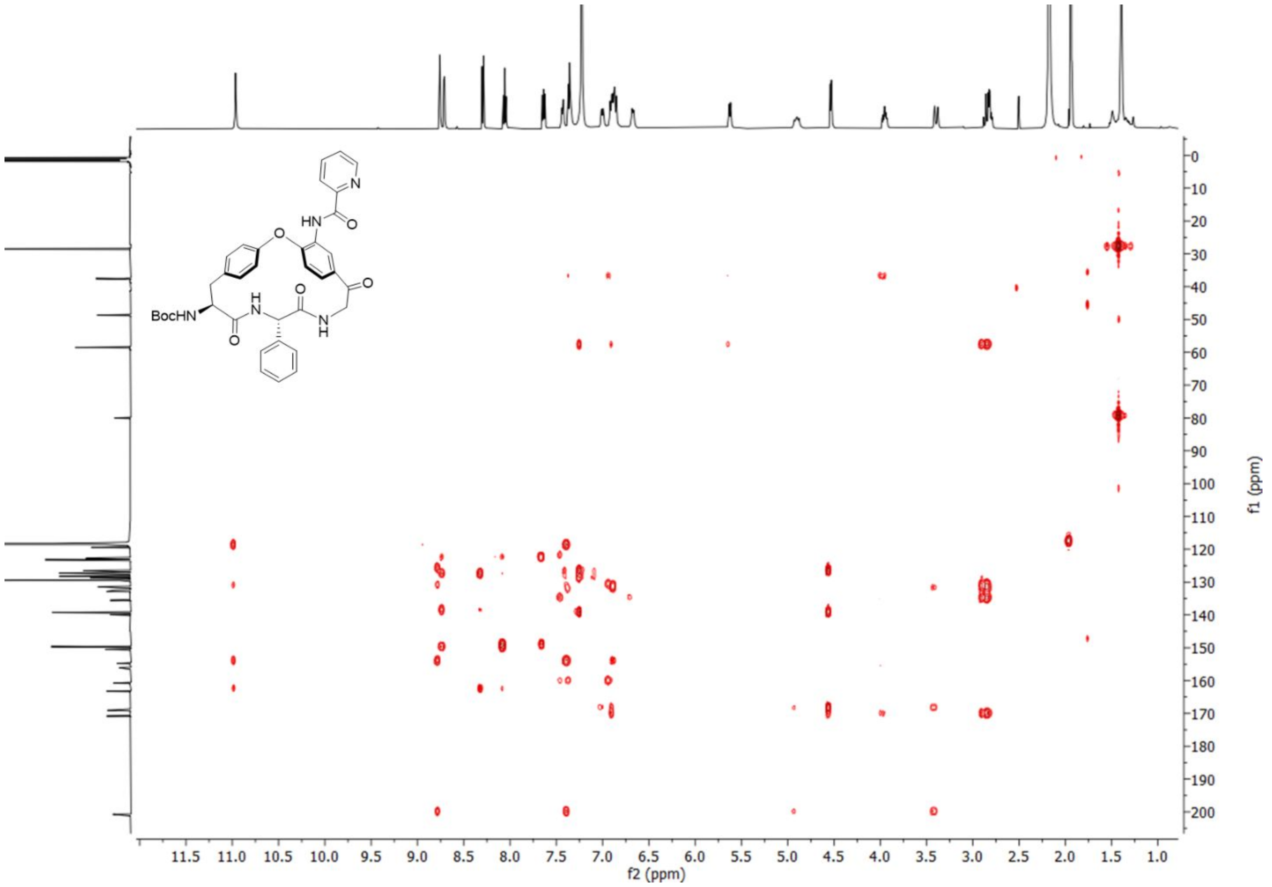

Compound 10: ^{1}H NMR (CD_3CN, 500 MHz)

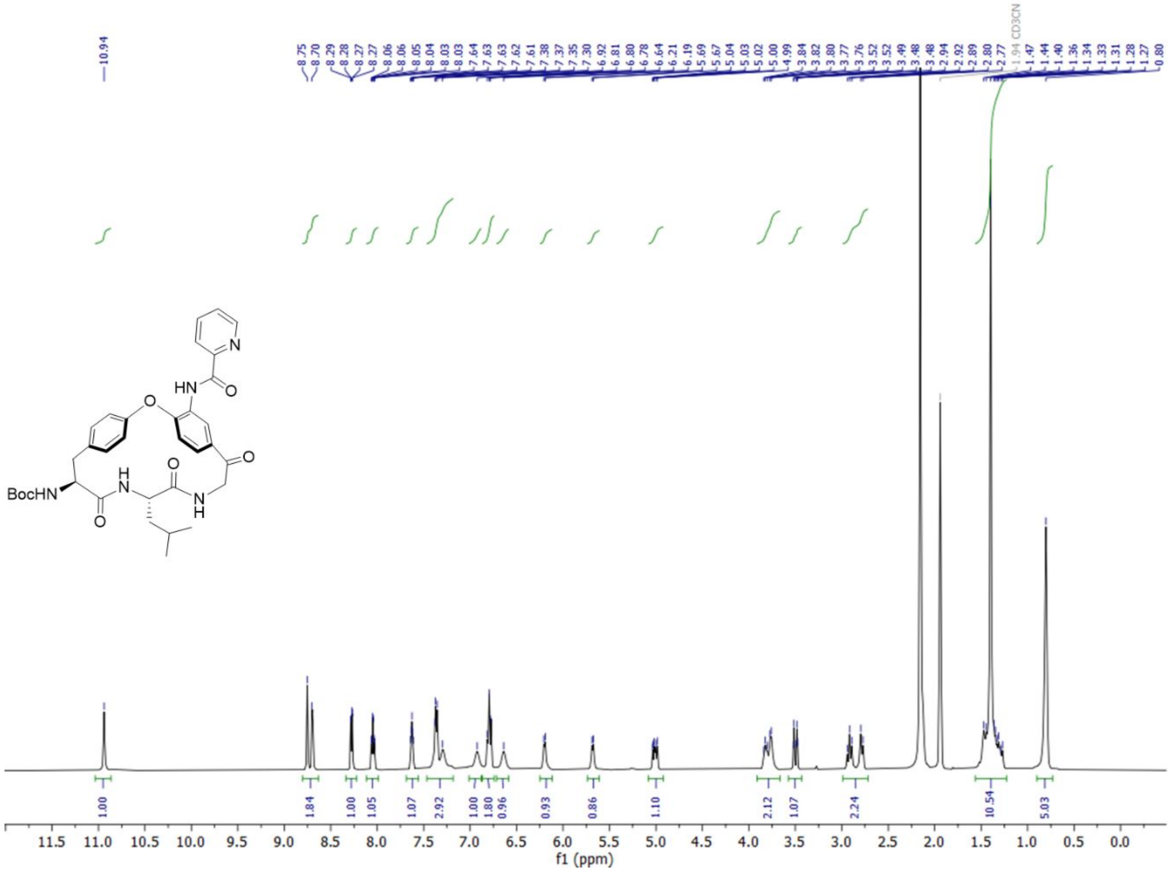

Compound 10: ^{13}C NMR (CD_3CN, 125 MHz)

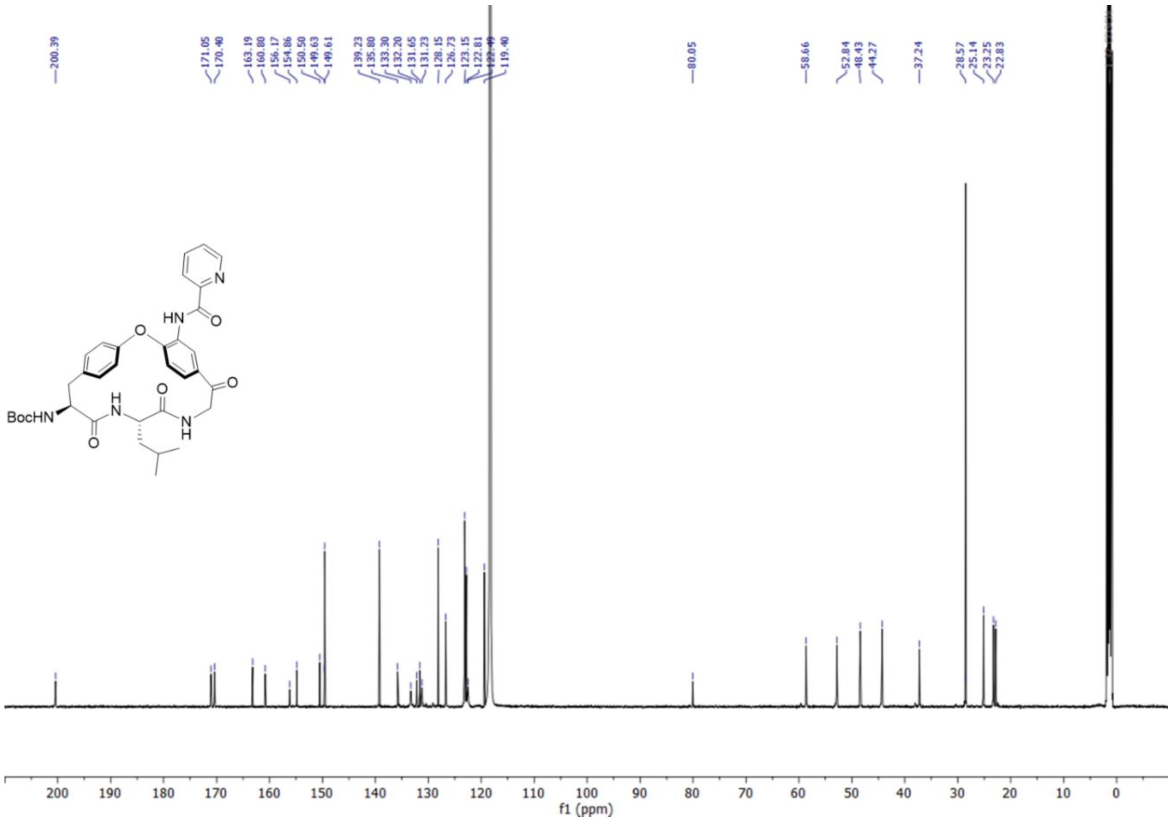

Compound 10: COSY NMR (CD_3CN)

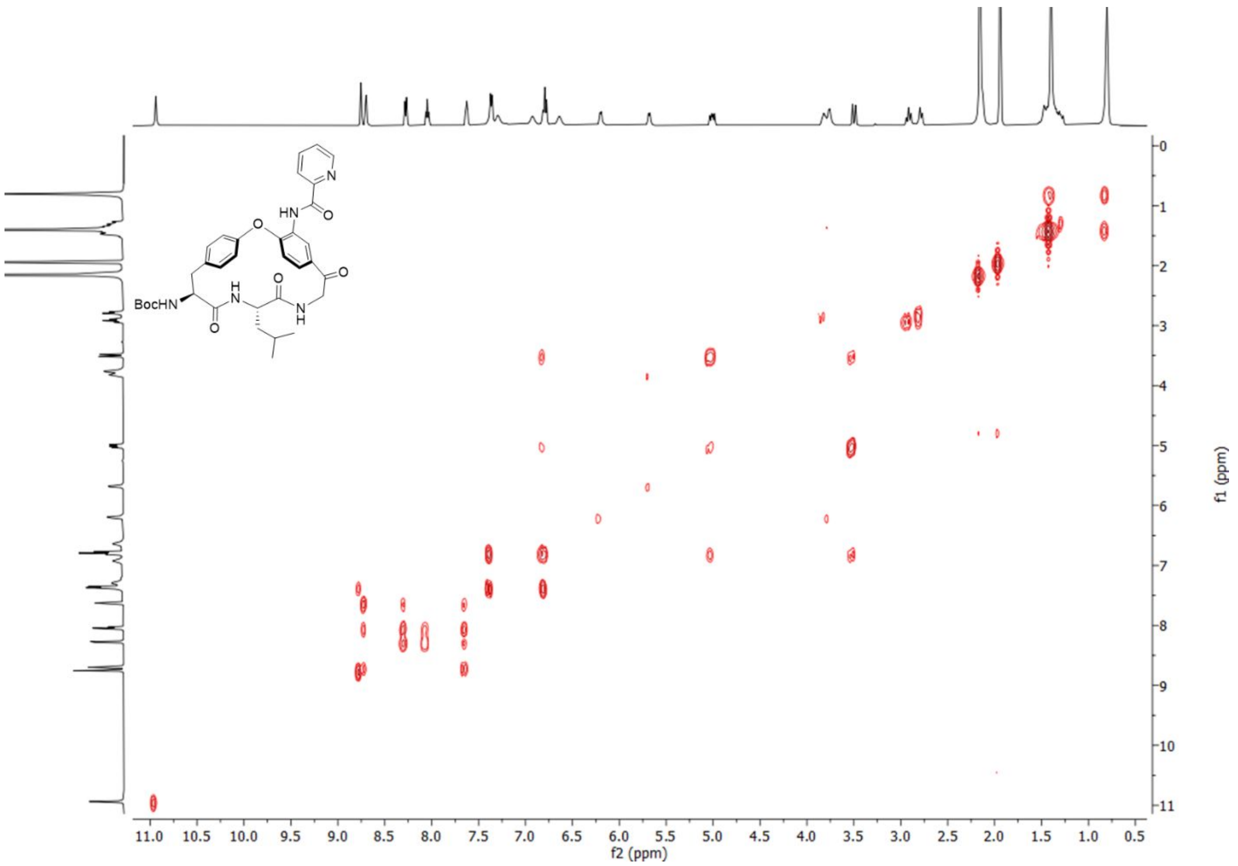

Compound 10: HSQC NMR (CD_3CN)

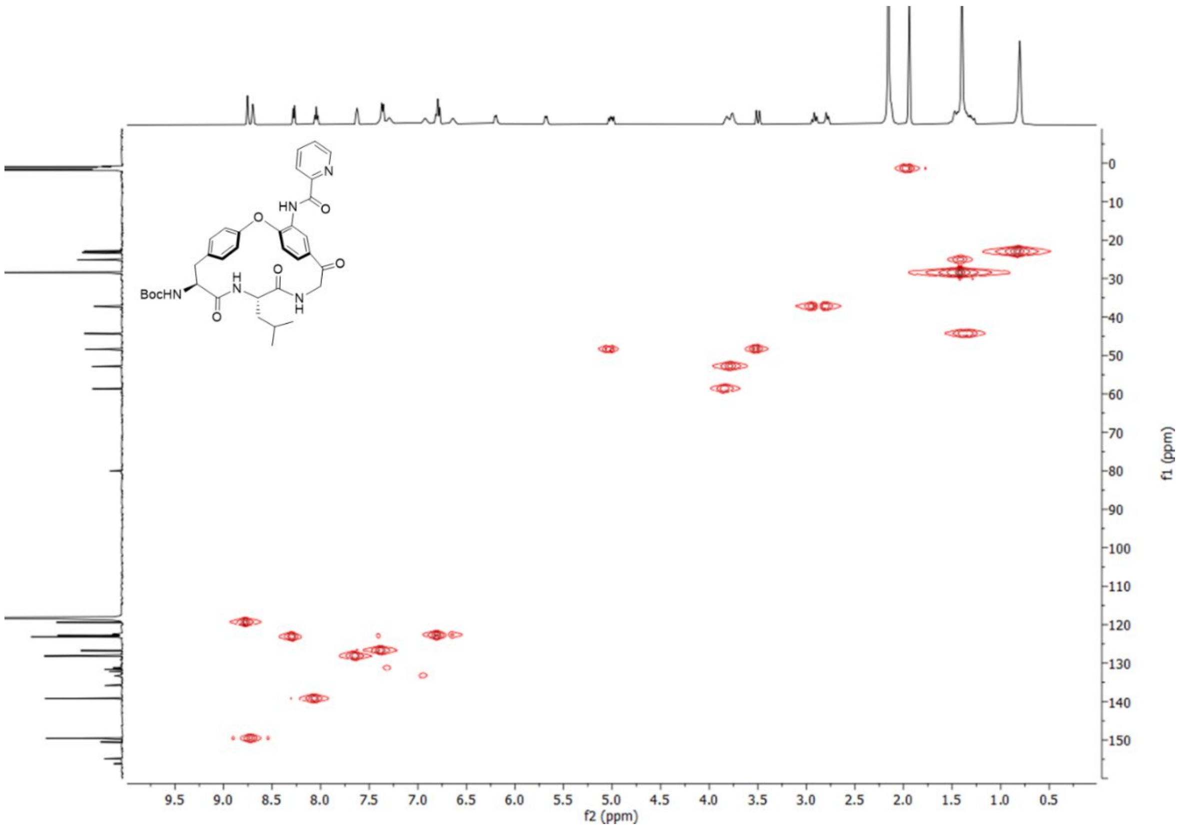

Compound 10: HMBC NMR (CD_3CN)

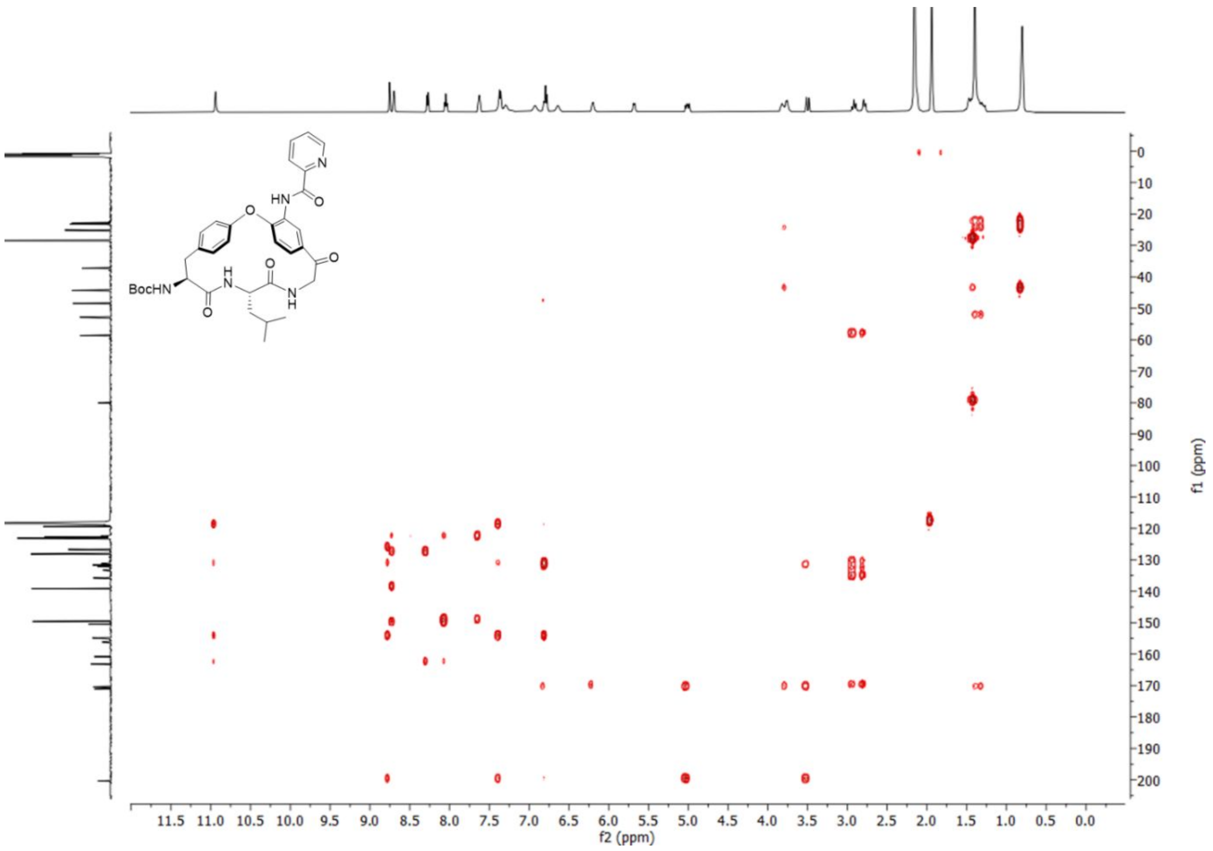

Compound 11: ^1H NMR ($CDCl_3$, 400 MHz)

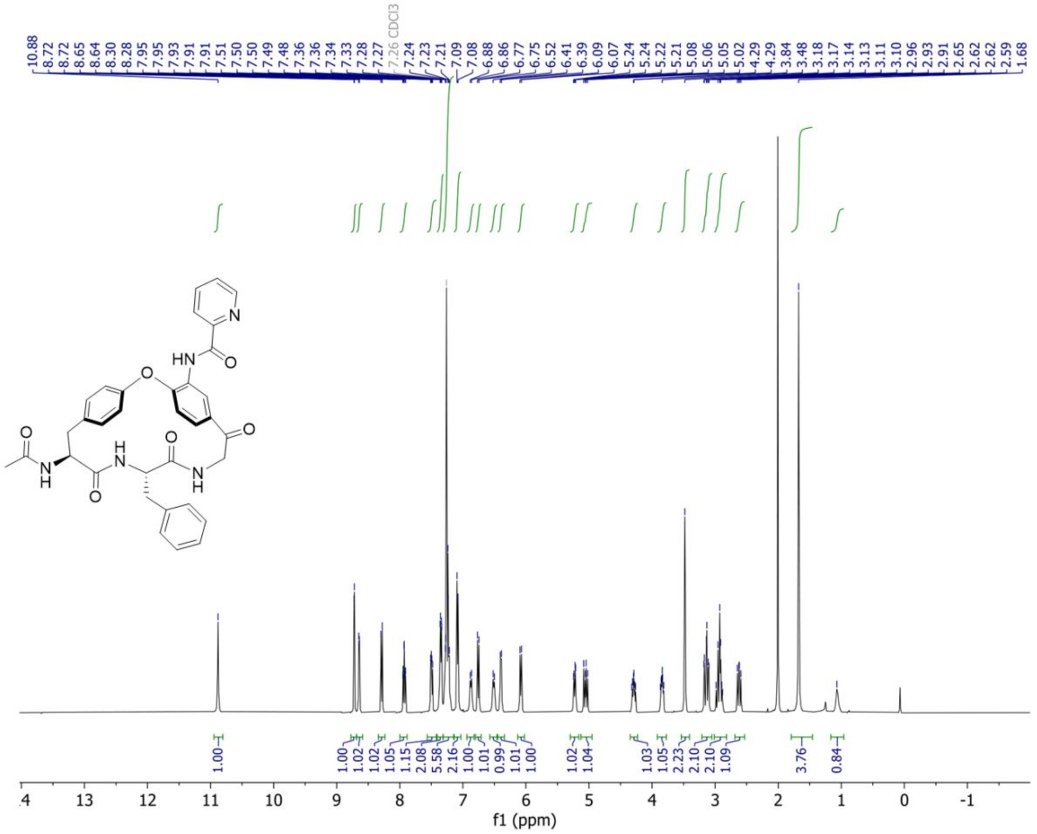

Compound 11: ^{13}C NMR (CDCl$_3$, 100 MHz)

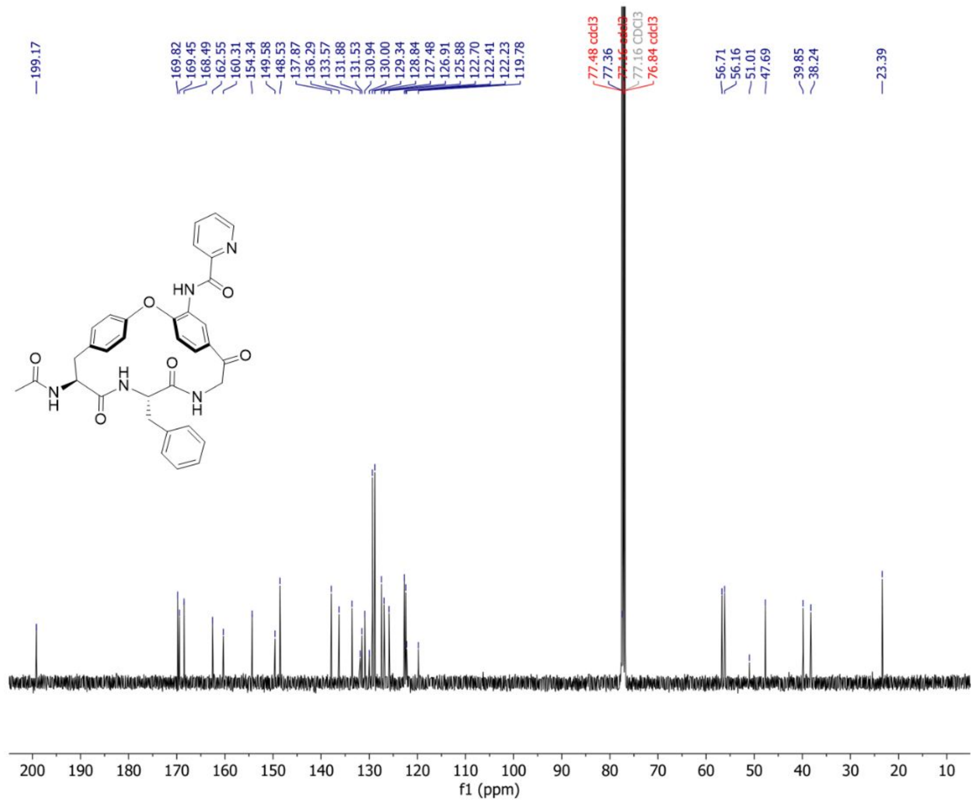

Compound 11: COSY NMR (CDCl$_3$)

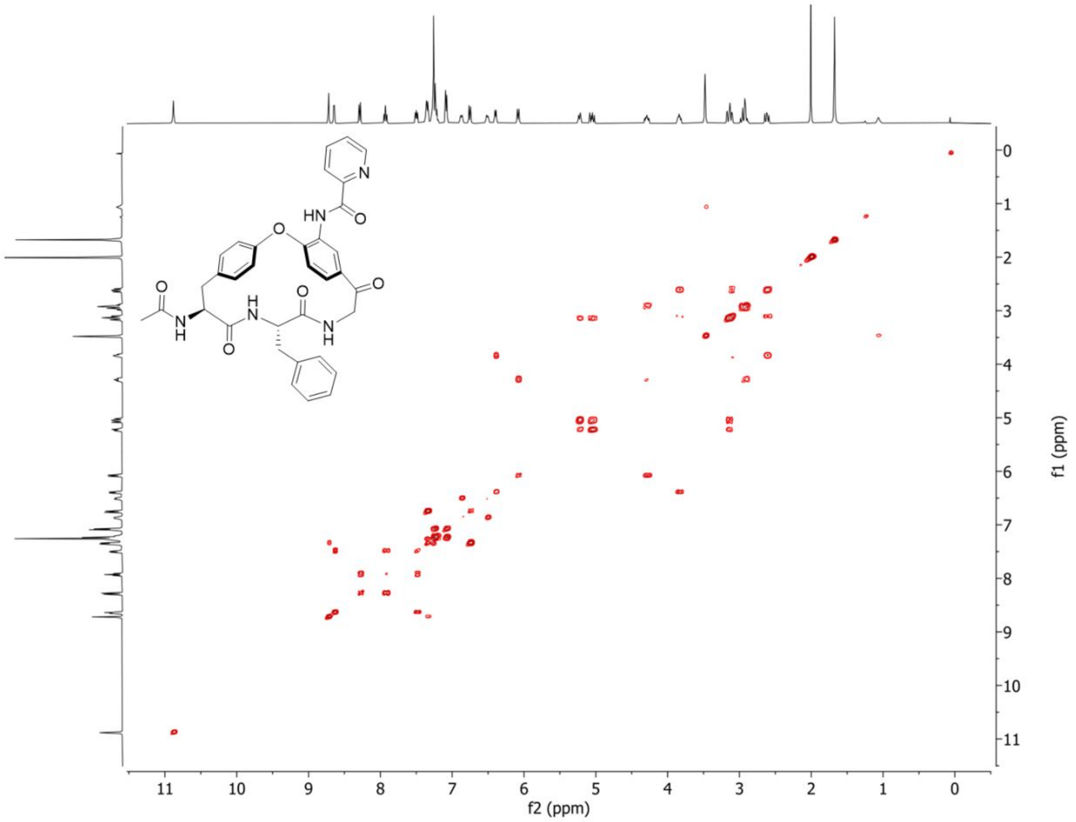

Compound 11: HSQC ($CDCl_3$)

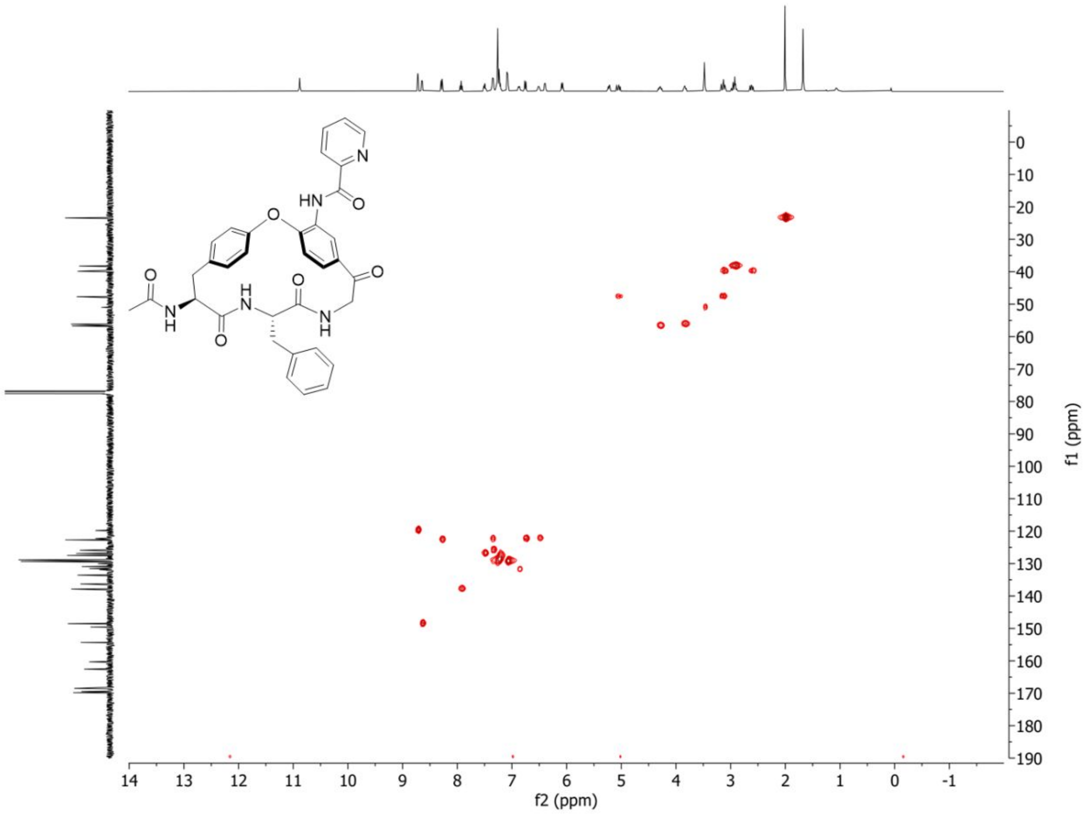

Compound 11: HMBC NMR ($CDCl_3$)

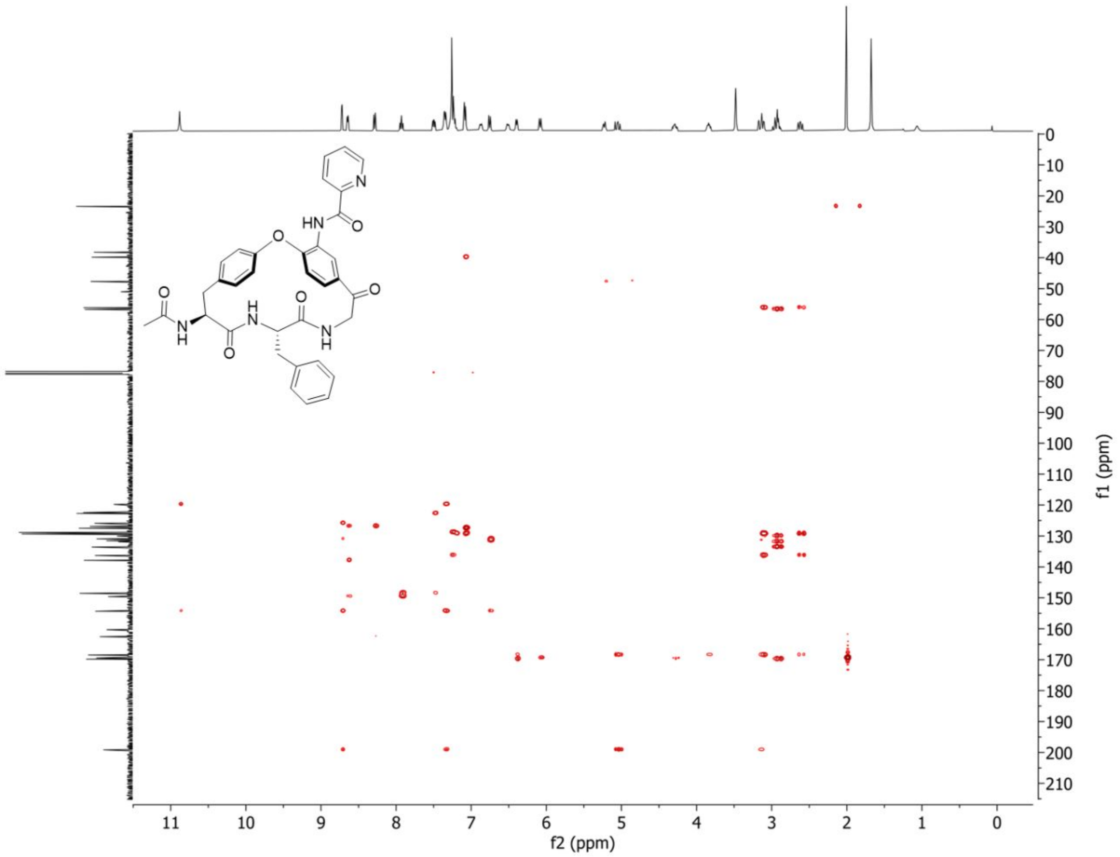

Compound 12: ^{1}H NMR (CD_3CN, 400 MHz)

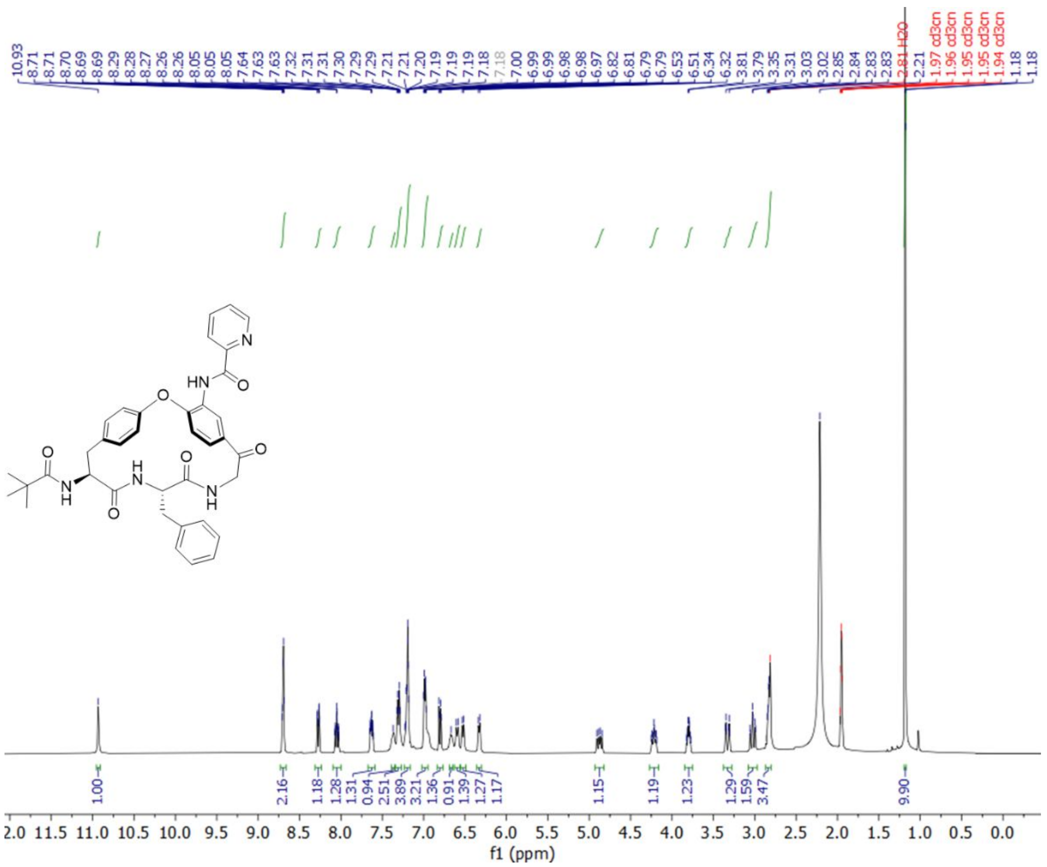

Compound 12: ^{13}C NMR (CD_3CN, 500 MHz)

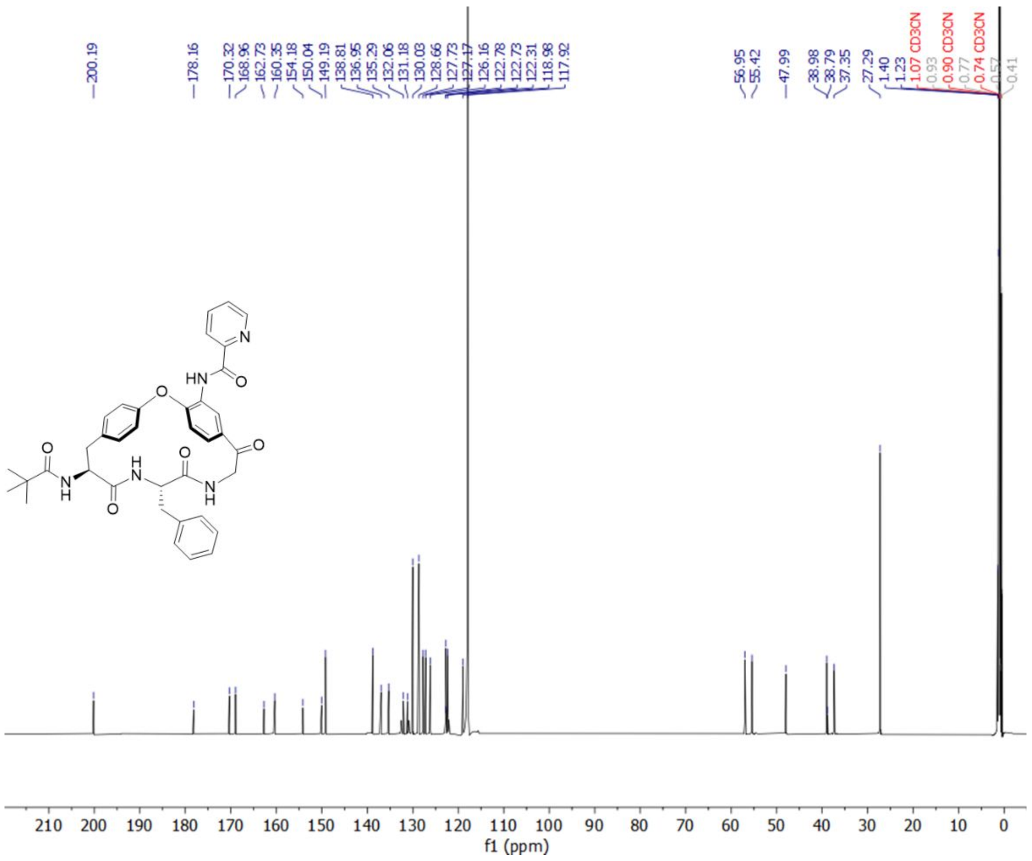

Compound 12: COSY (CD_3CN)

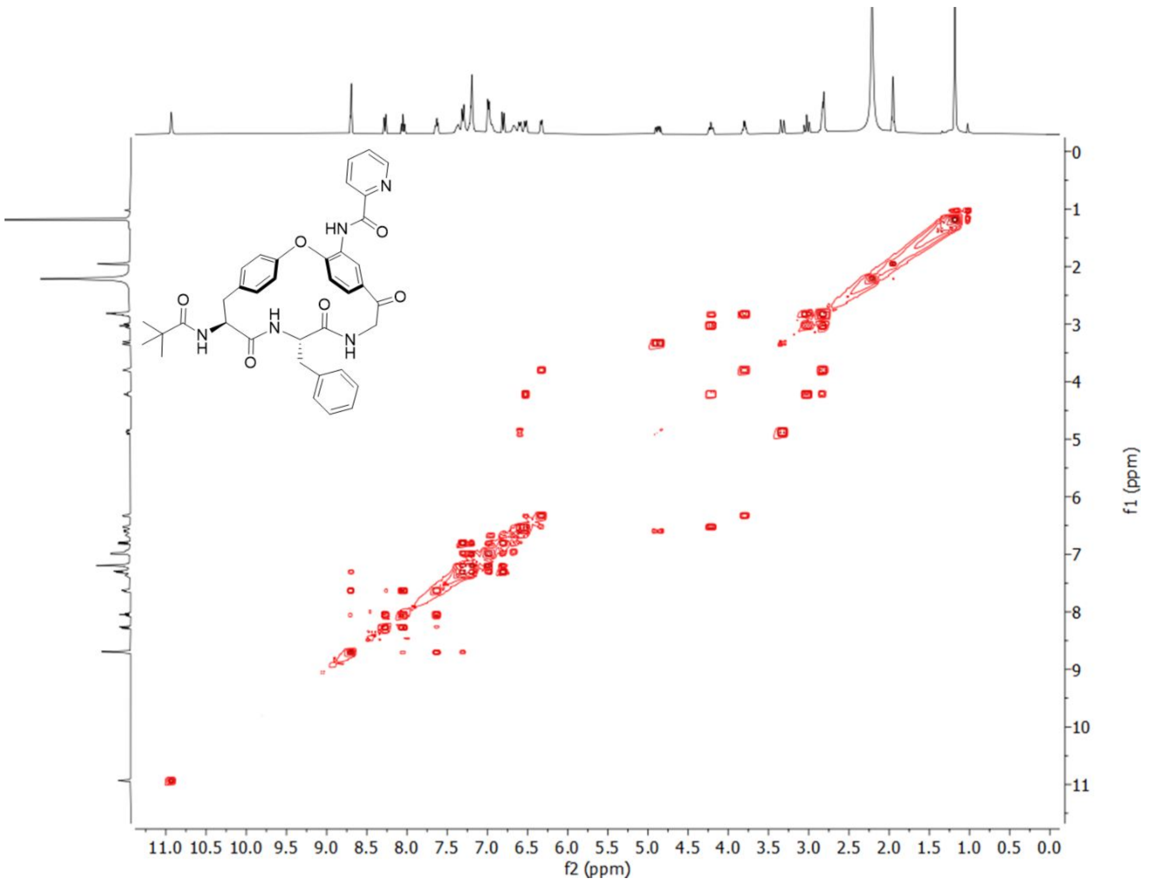

Compound 12: HSQC (CD_3CN)

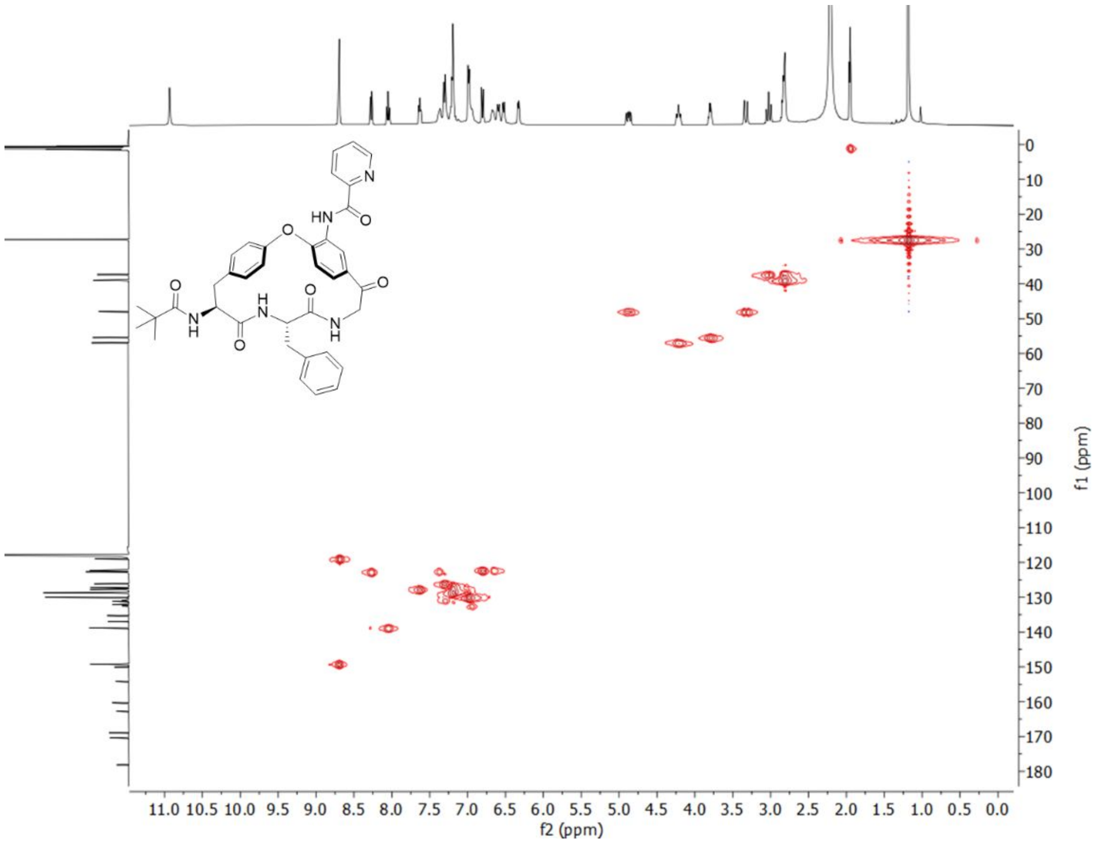

Compound 12: HMBC (CD_3CN)

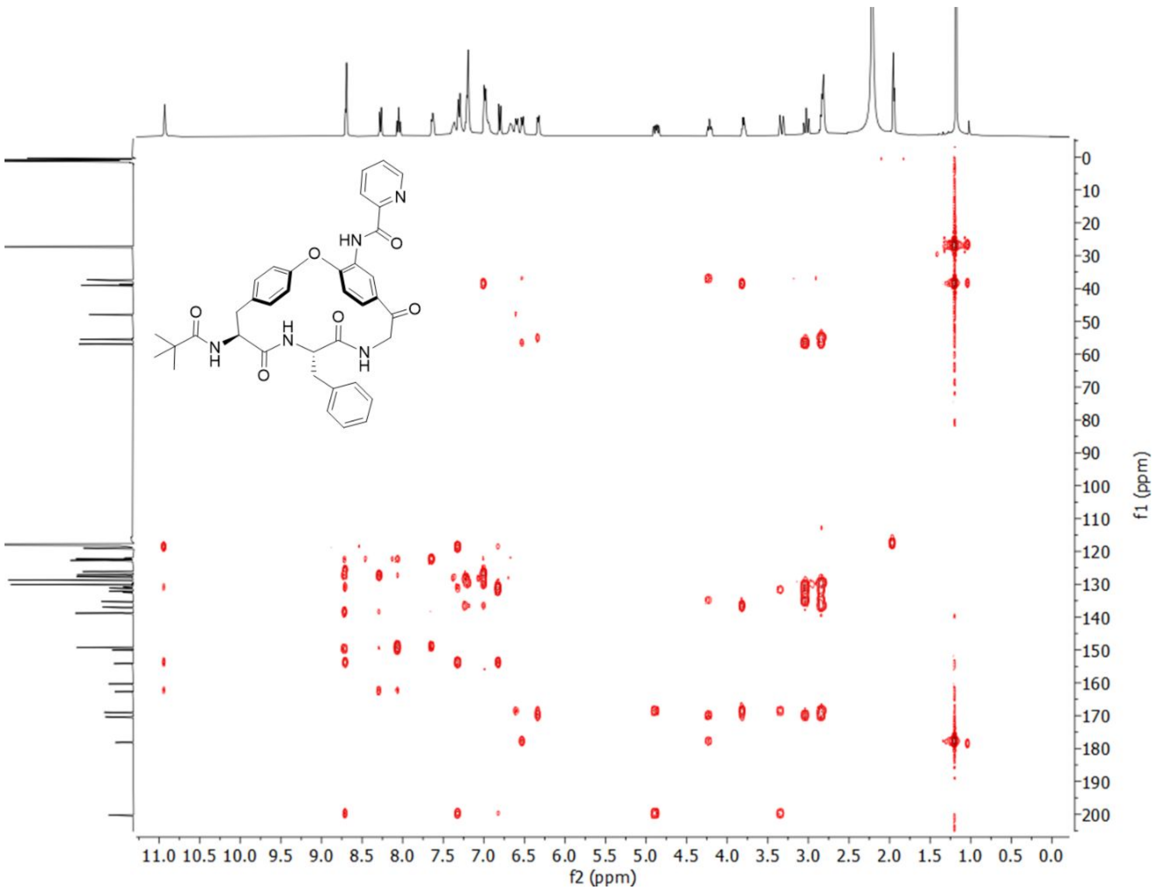

Compound 15: ^1H NMR (CD_3CN, 400 MHz)

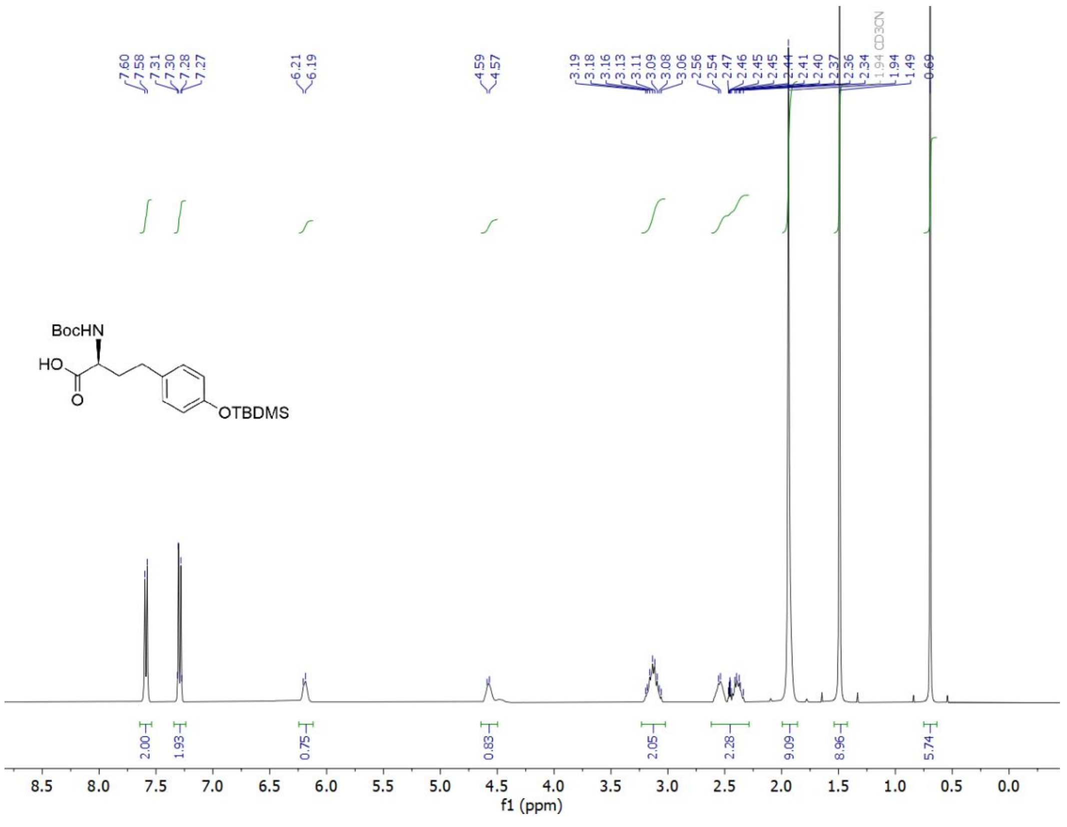

Compound 15: ^{13}C NMR (CD_3CN, 100 MHz)

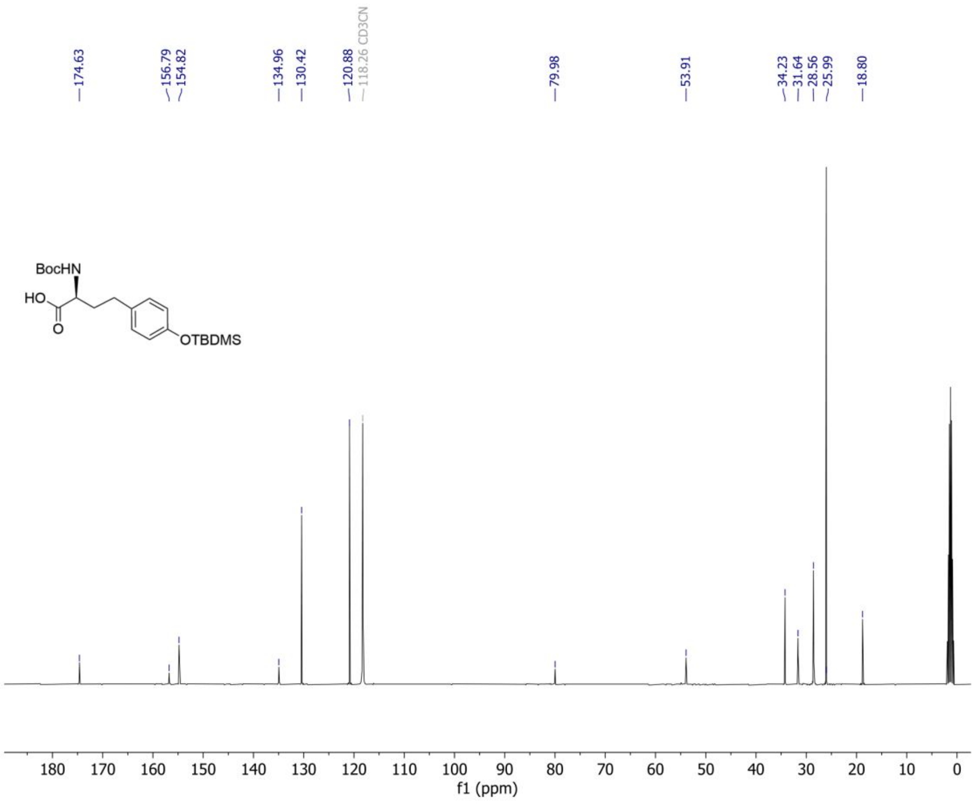

Compound 16: ^{1}H NMR (CD_3CN, 400 MHz)

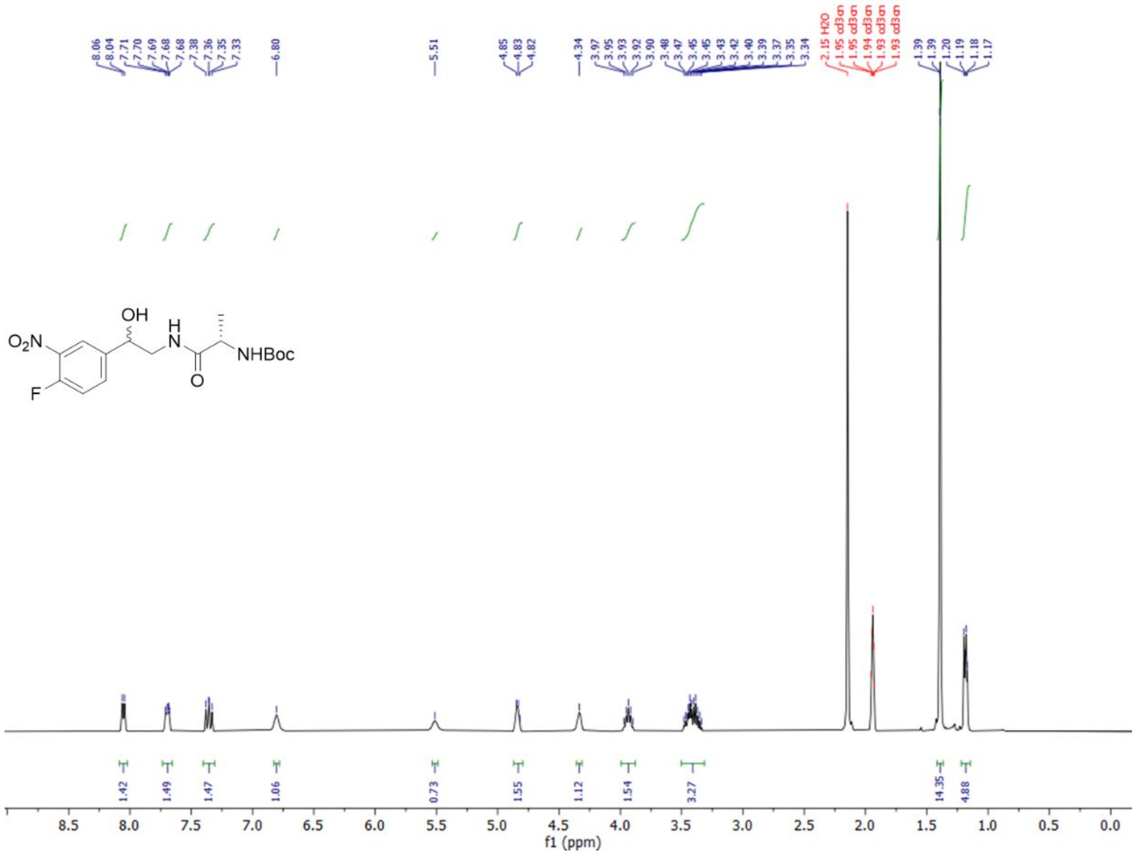

Compound 16: ^{13}C NMR (CD_3CN, 100 MHz)

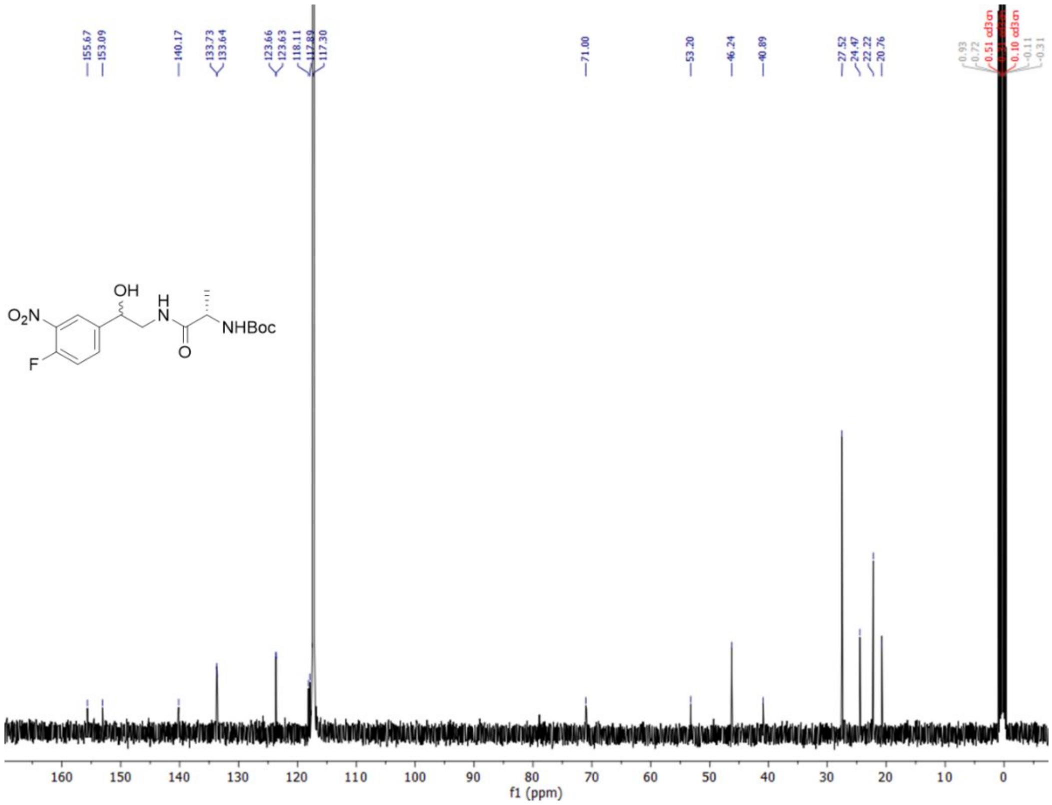

Compound 17: ^{1}H NMR (CD_3CN, 400 MHz)

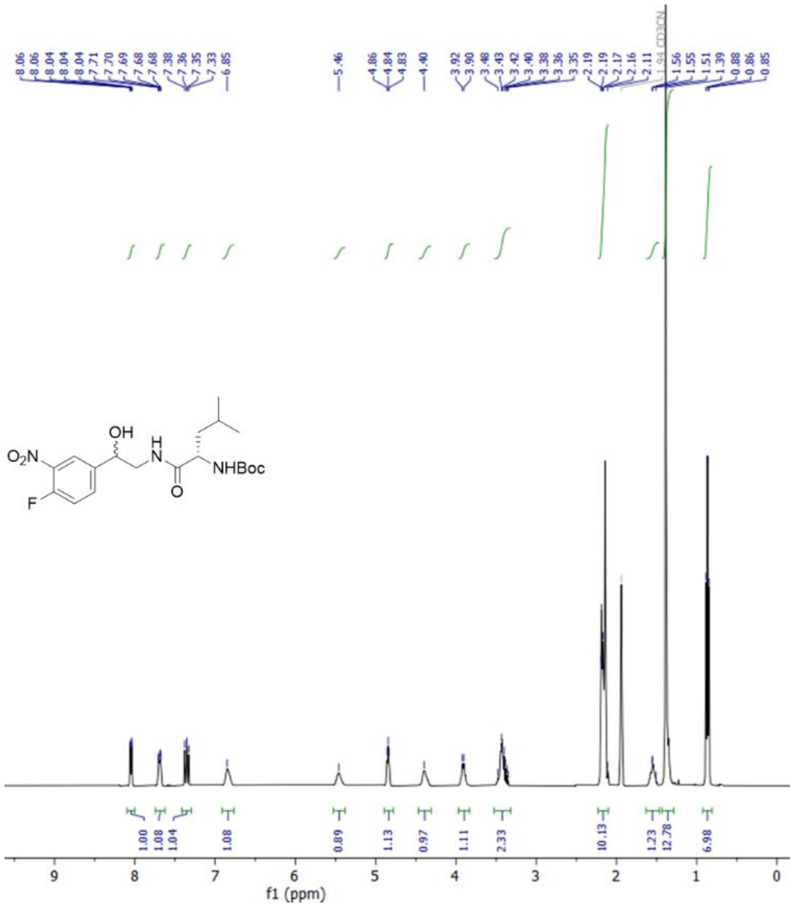

Compound 17: ^{13}C NMR (CD_3CN, 100 MHz)

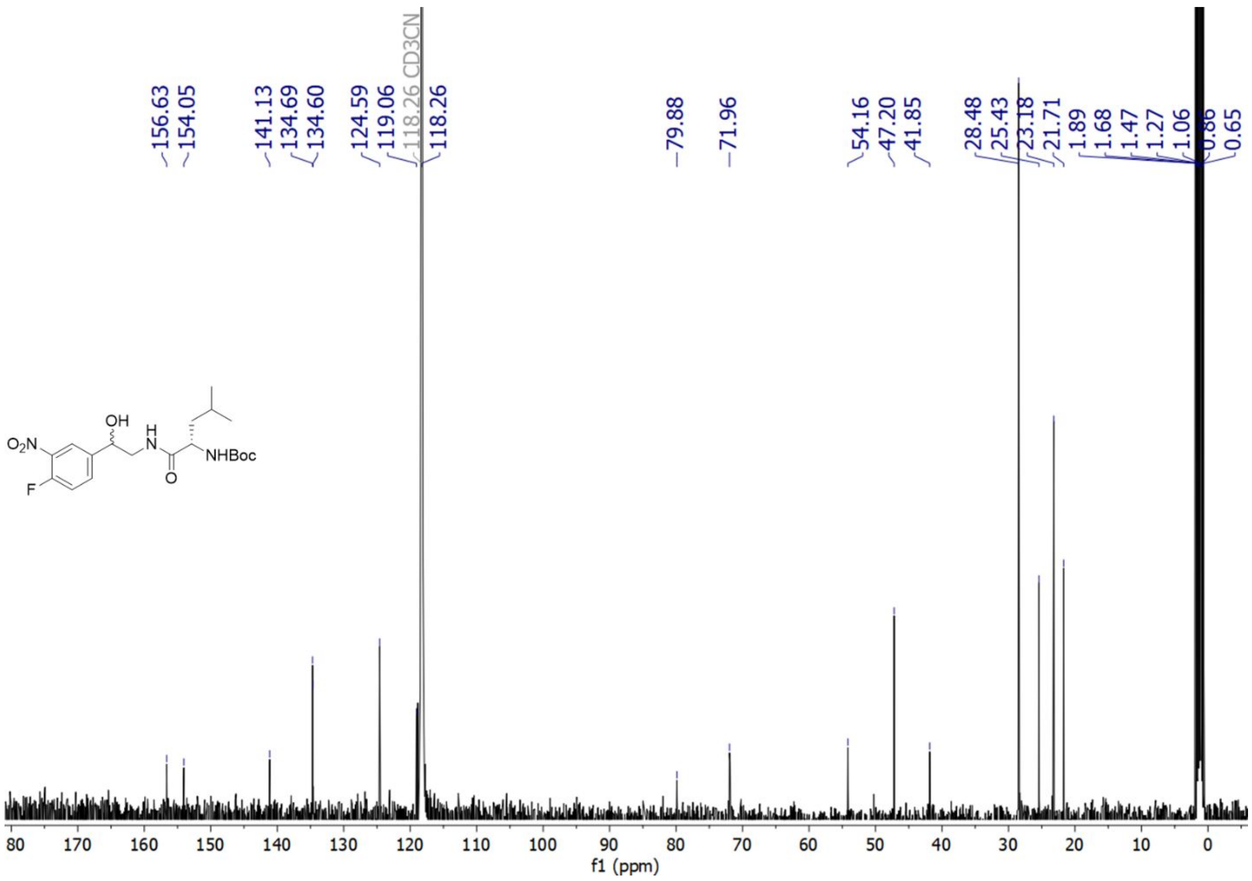

Compound 19: ^{1}H NMR (DMSO-d_6, 500 MHz)

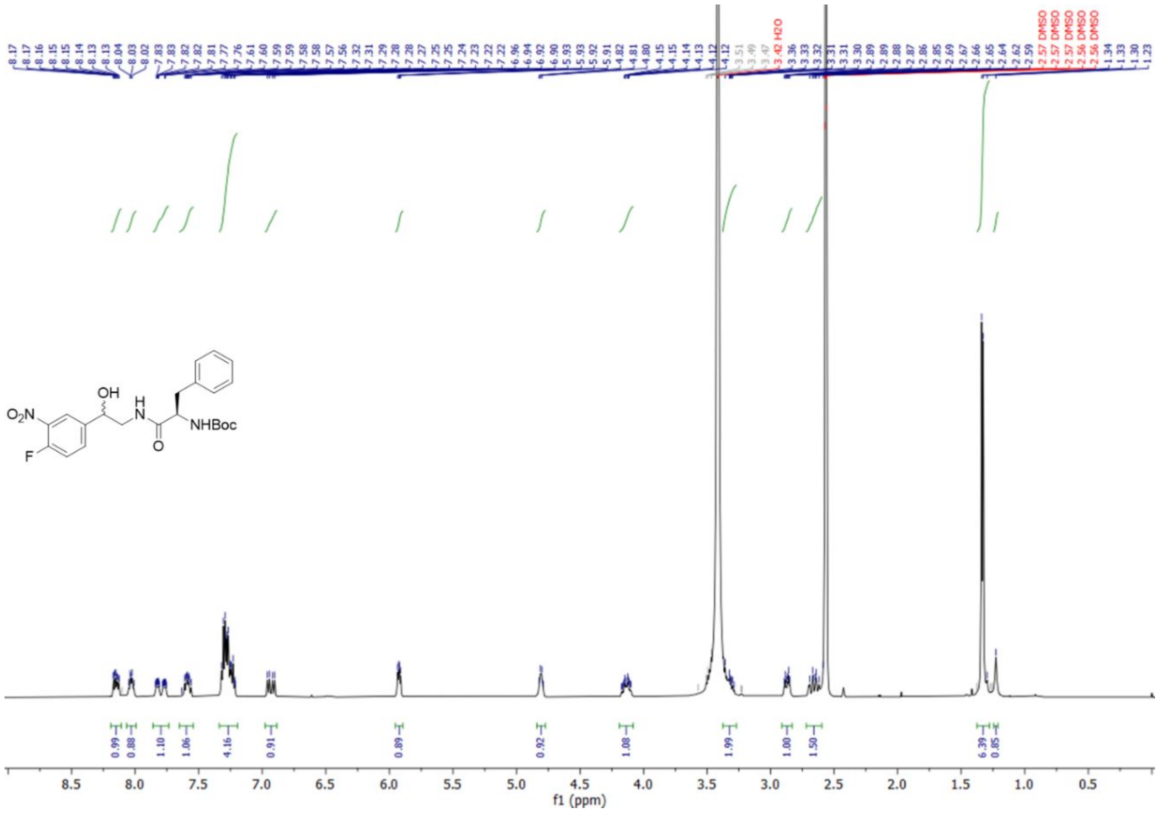

Compound 19: ^{13}C NMR (DMSO-d_6, 125 MHz)

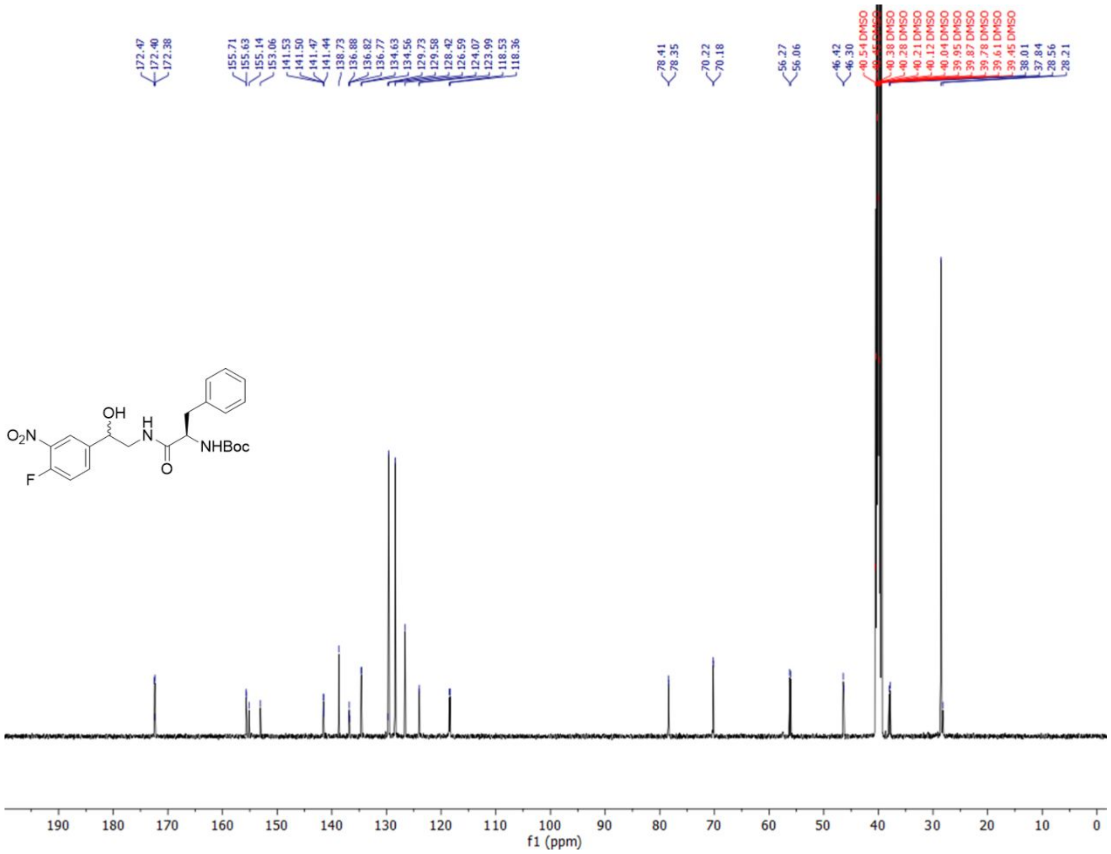

Compound 24: ^{1}H NMR (DMSO-d_6, 500 MHz)

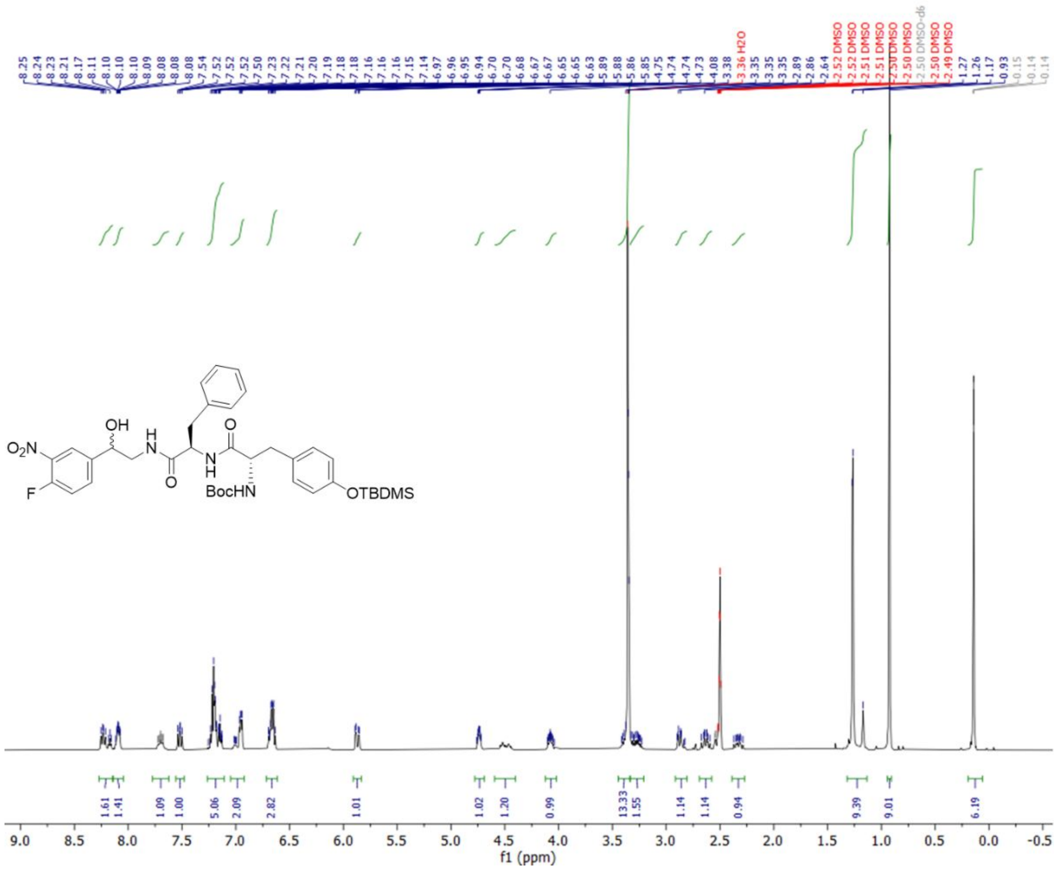

Compound 24: ^{13}C NMR (DMSO-d_6, 125 MHz)

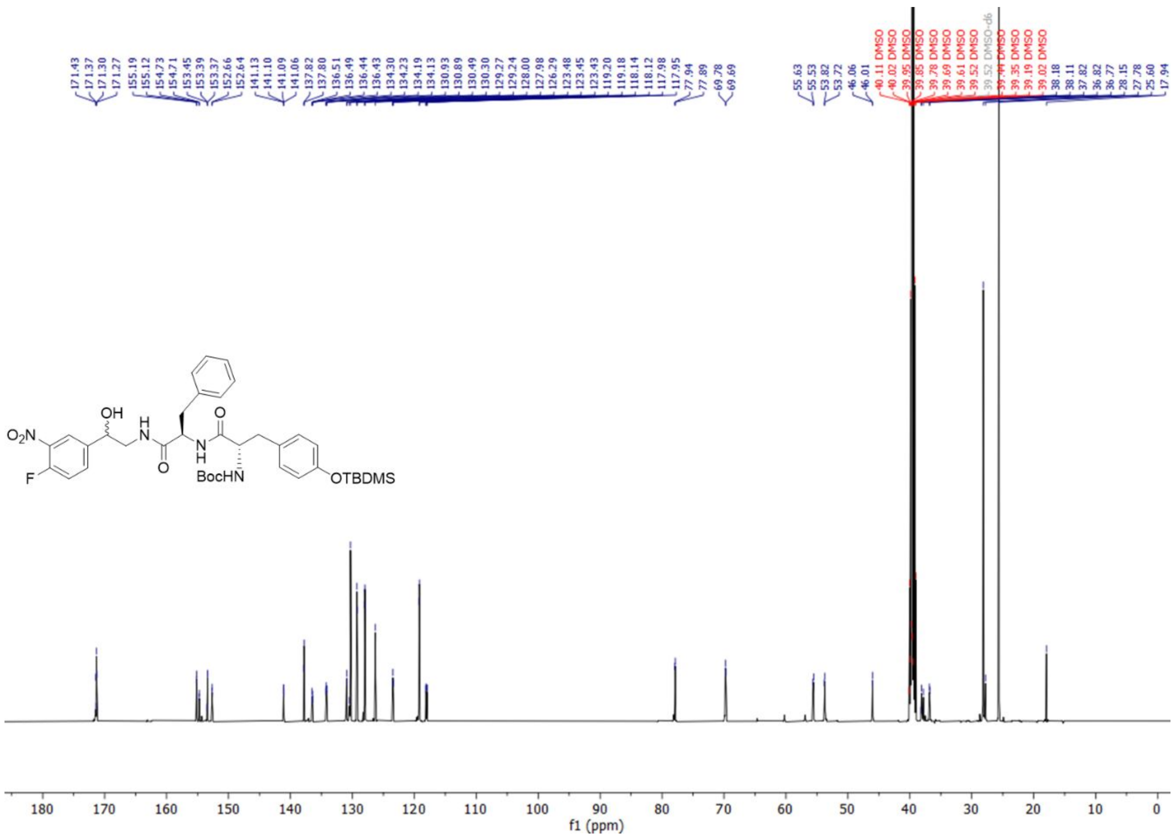

Compound 26: ^{1}H NMR (DMSO-d_6, 400 Hz)

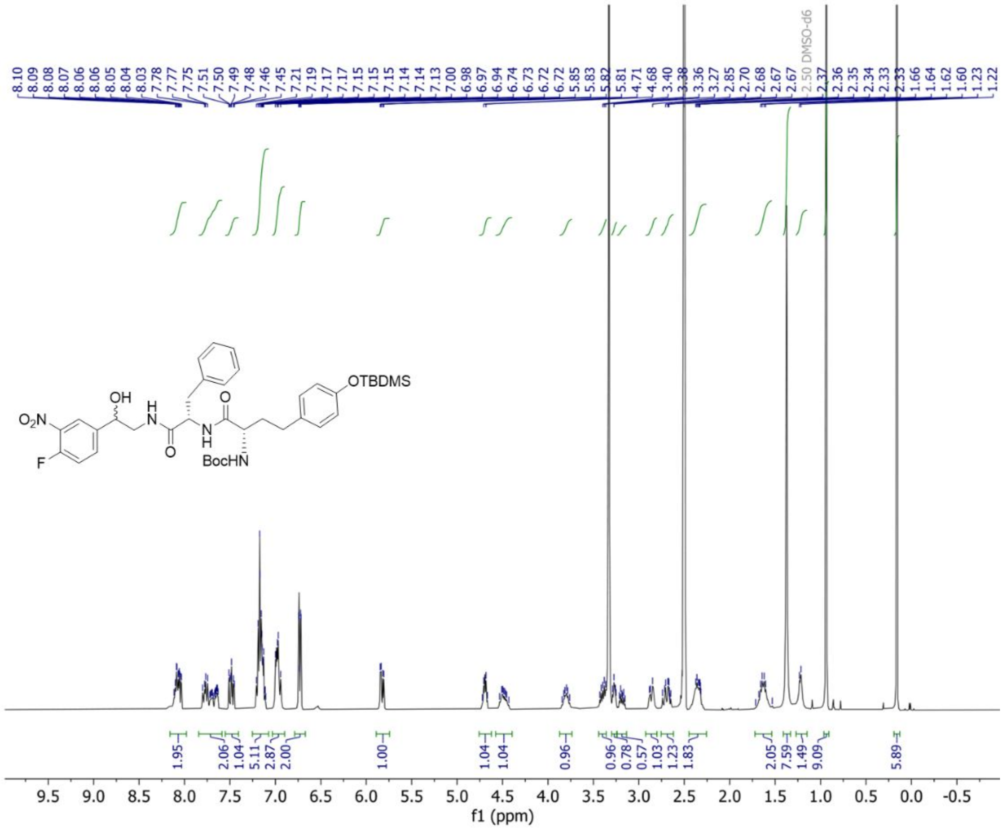

Compound 26: ^{13}C NMR (DMSO-d$_6$, 100 MHz)

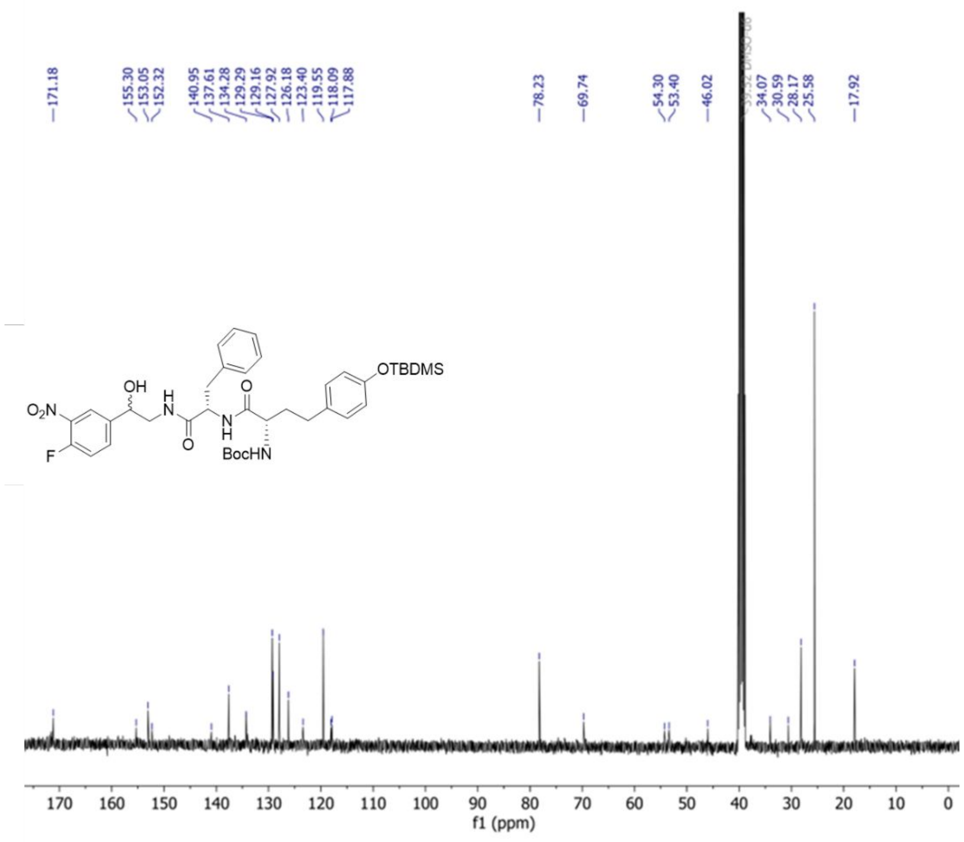

Compound 27: ^{1}H NMR (DMSO-d$_6$, 400 MHz)

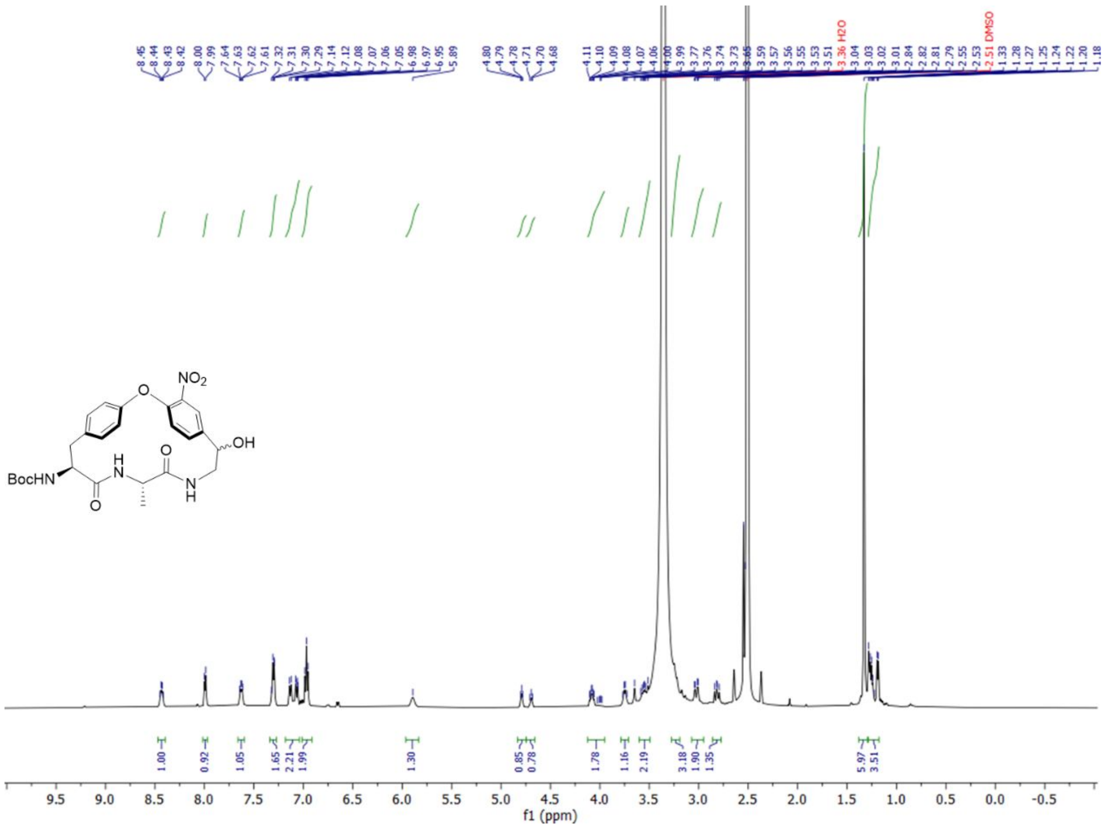

Compound 27: ^{13}C NMR (DMSO-d$_6$, 100 MHz)

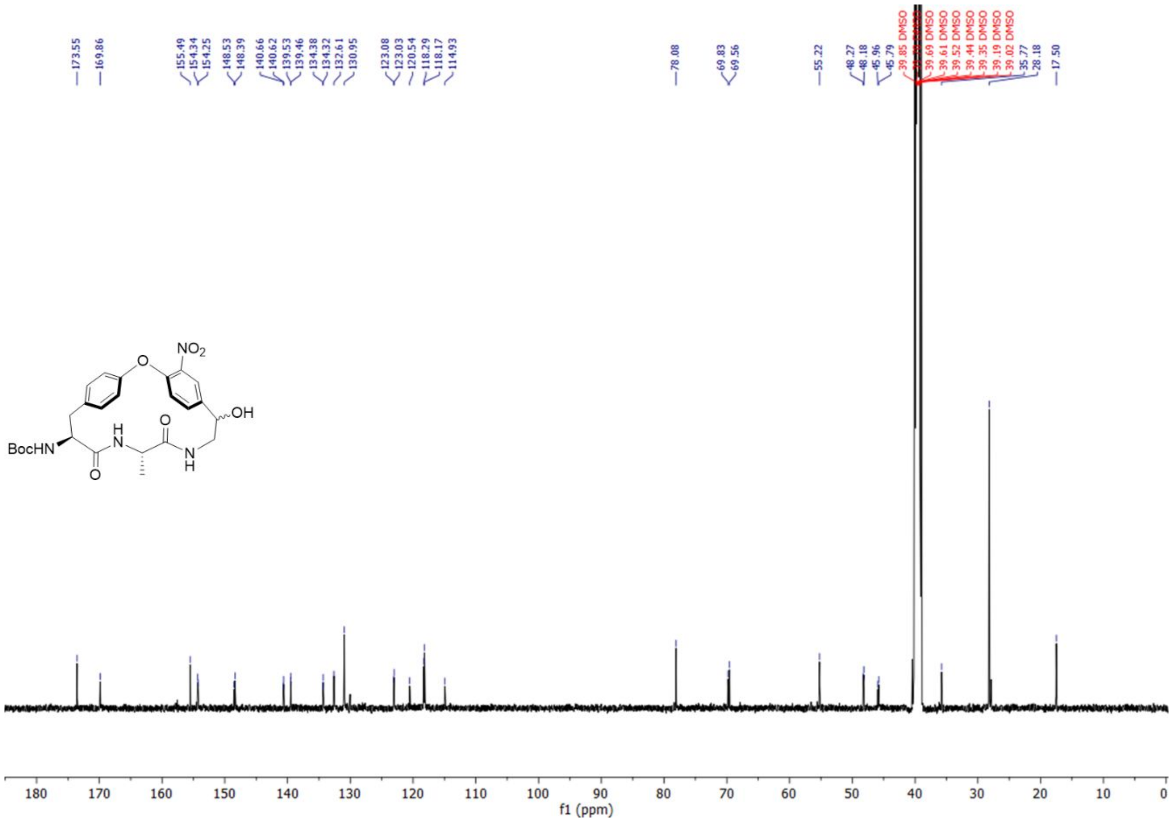

Compound 28: ^{1}H NMR (DMSO-d$_6$, 500 MHz)

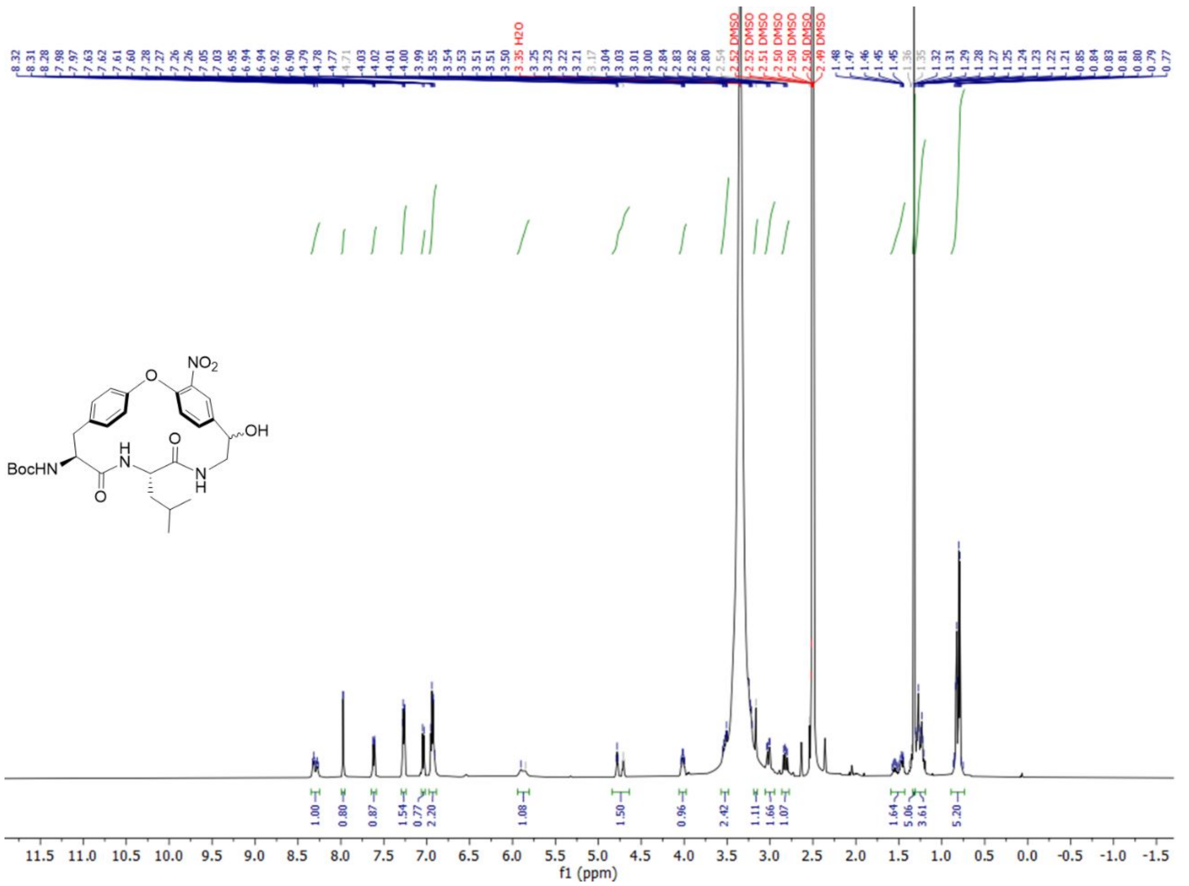

Compound 28: ^{13}C NMR (DMSO-d_6, 125 MHz)

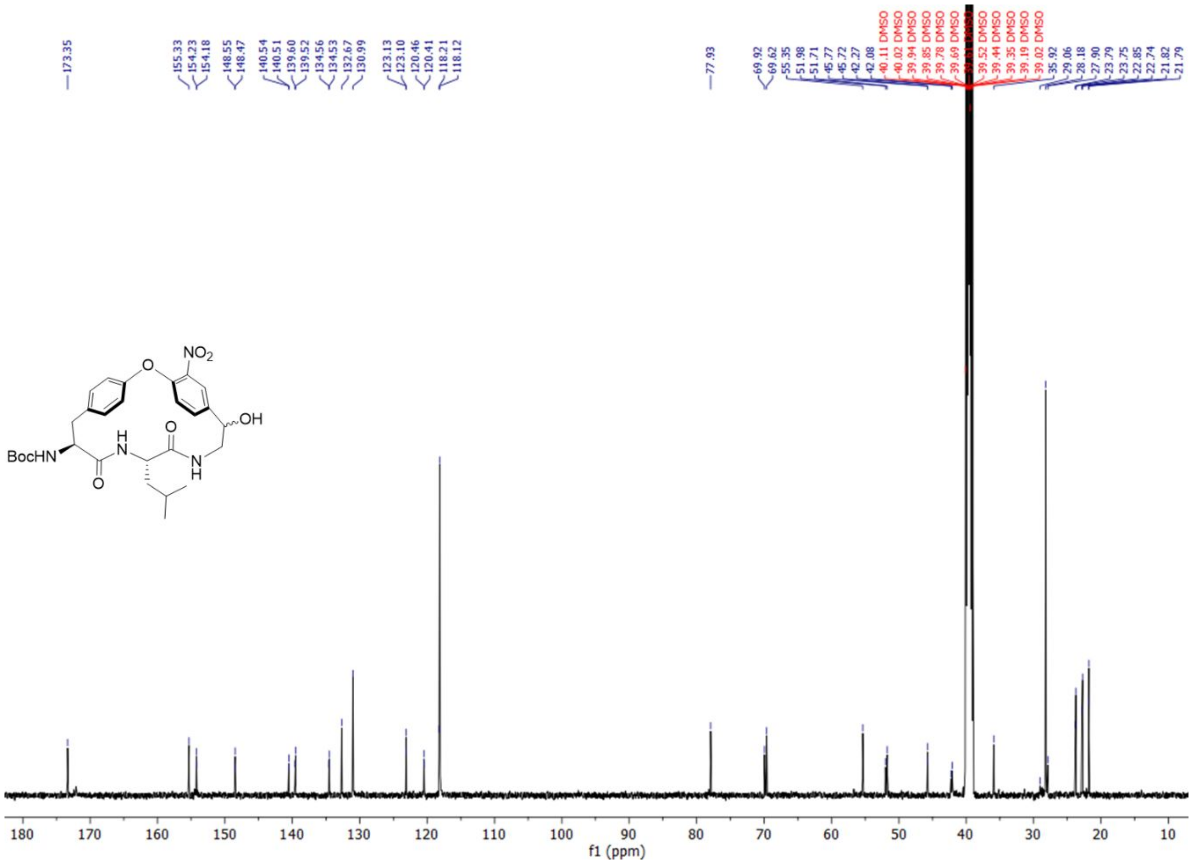

Compound 30: ^{1}H NMR (DMSO-d_6, 500 MHz)

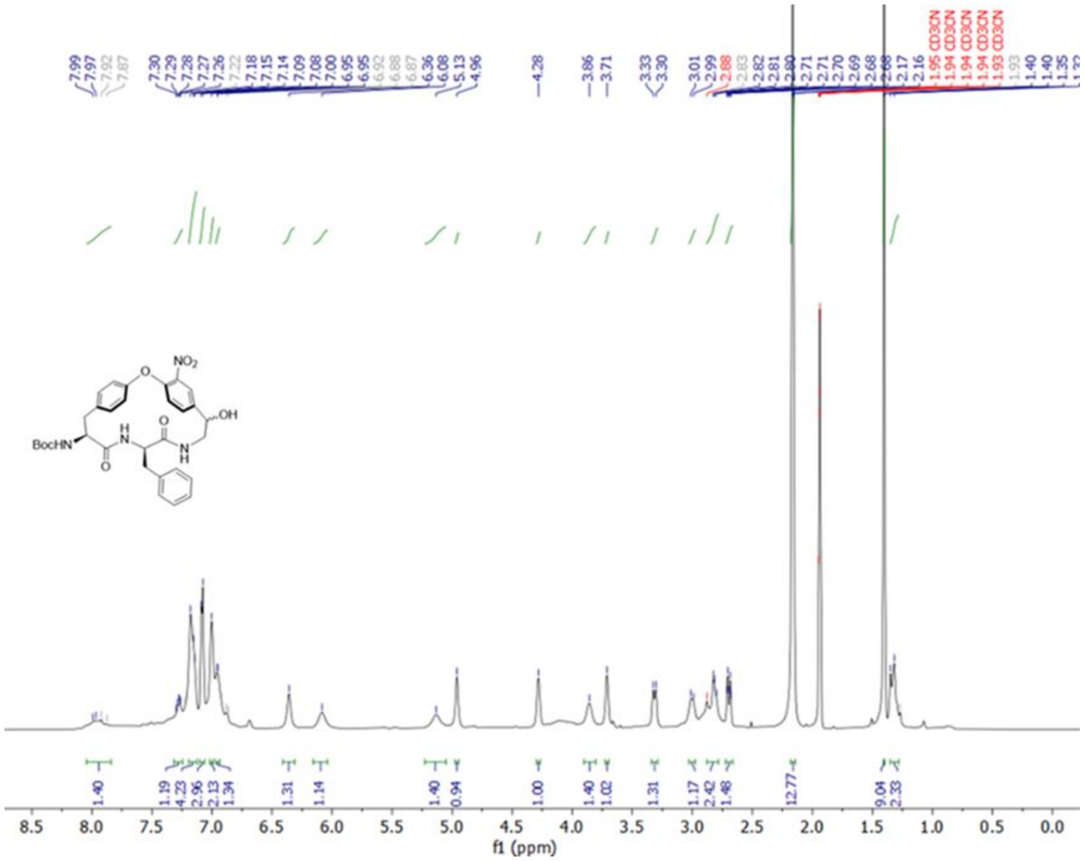

Compound 30: ^{13}C NMR (DMSO-d$_6$, 125 MHz)

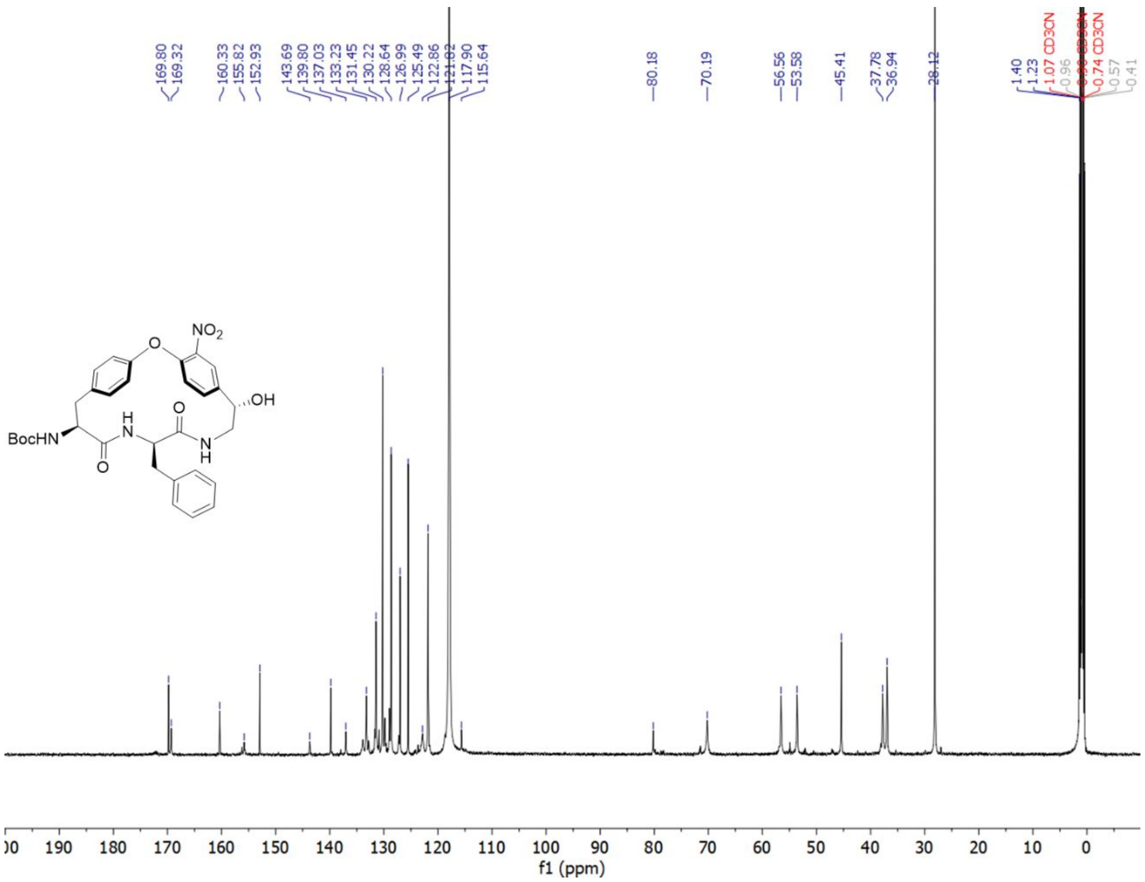

Compound 32: ^{1}H NMR (CD$_3$CN, 500 MHz)

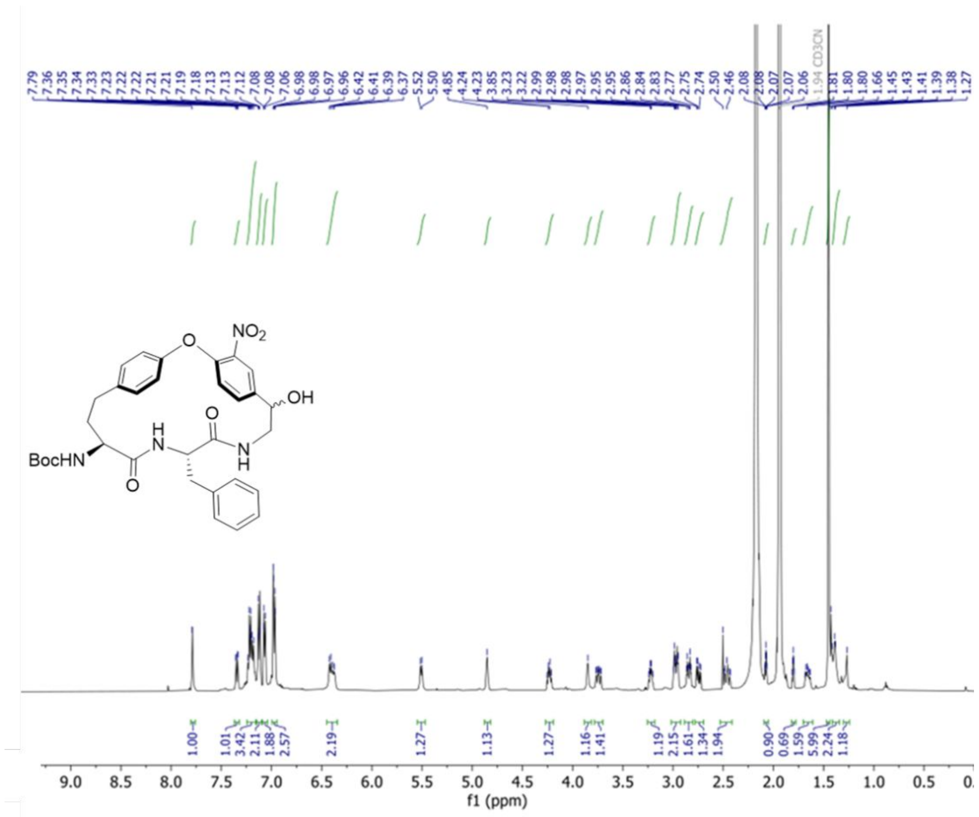

Compound 32: ^{13}C NMR (CD_3CN, 125 MHz)

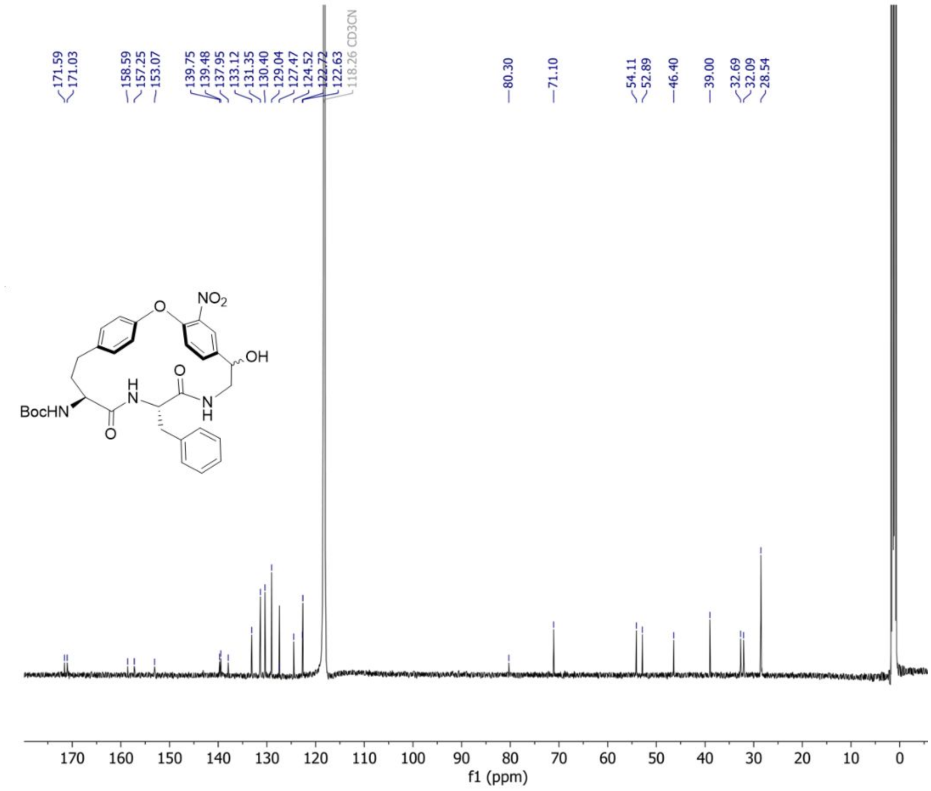

Compound 34: ^{1}H NMR (DMSO-d_6, 500 MHz)

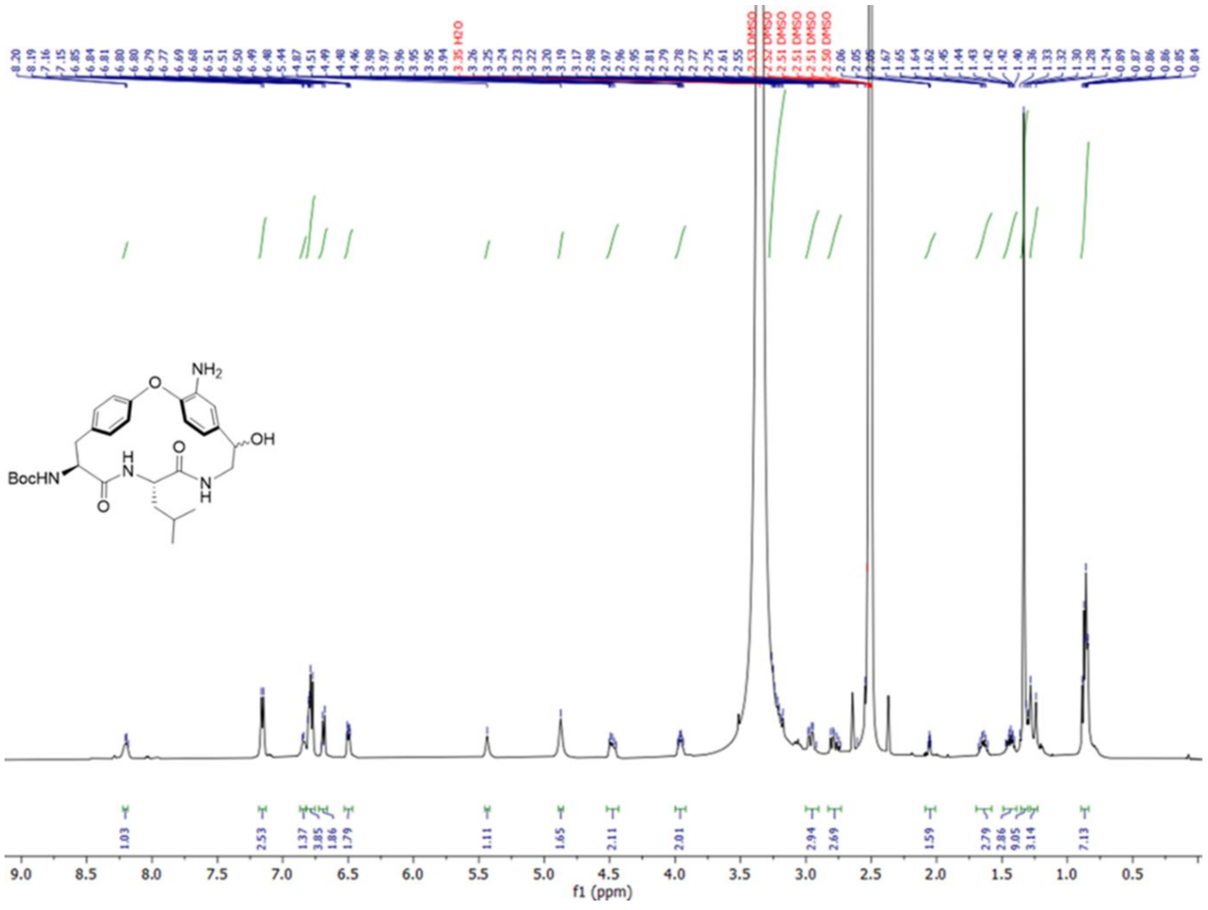

Compound 34: ^{13}C NMR (DMSO-d_6, 125 MHz)

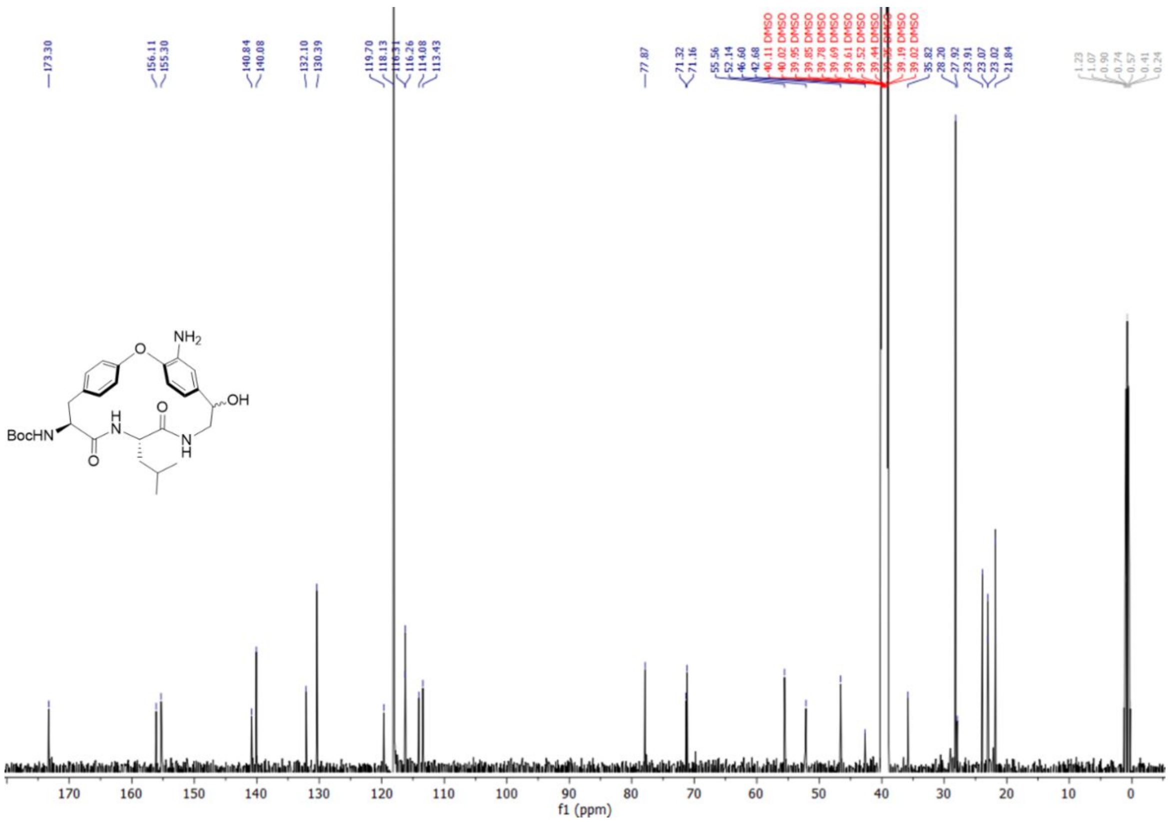

Compound 35: ^{1}H NMR (DMSO-d_6, 500 MHz)

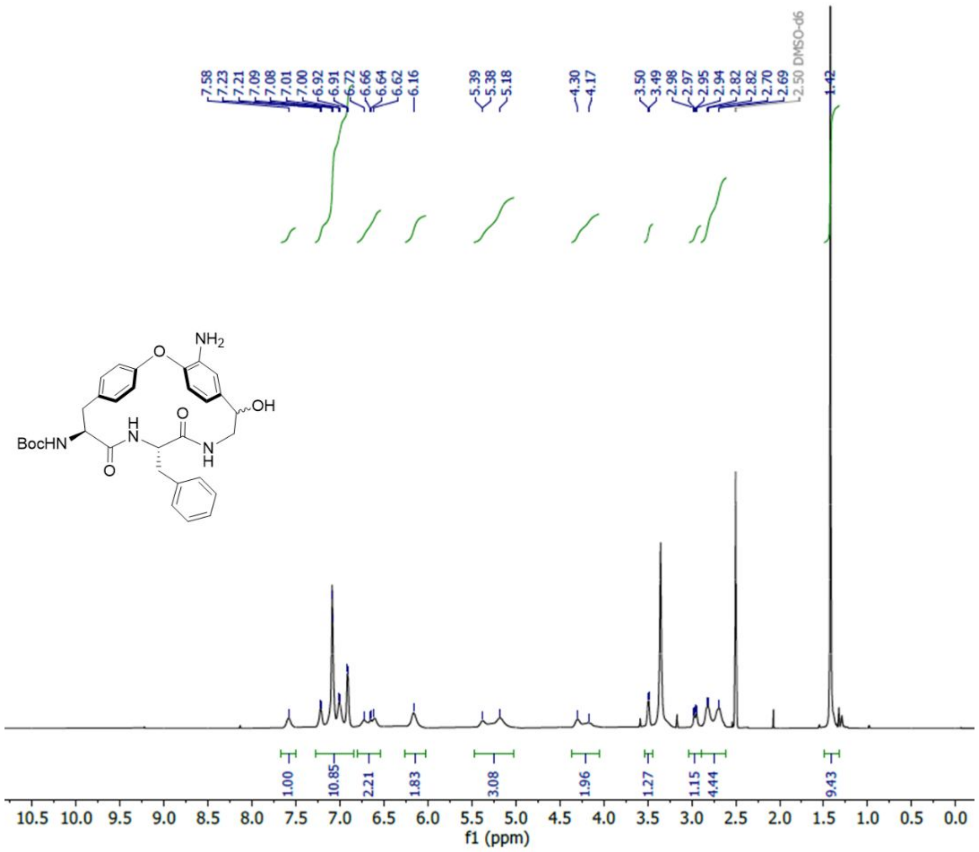

Compound 35: ^{13}C NMR (DMSO-d_6, 500 MHz)

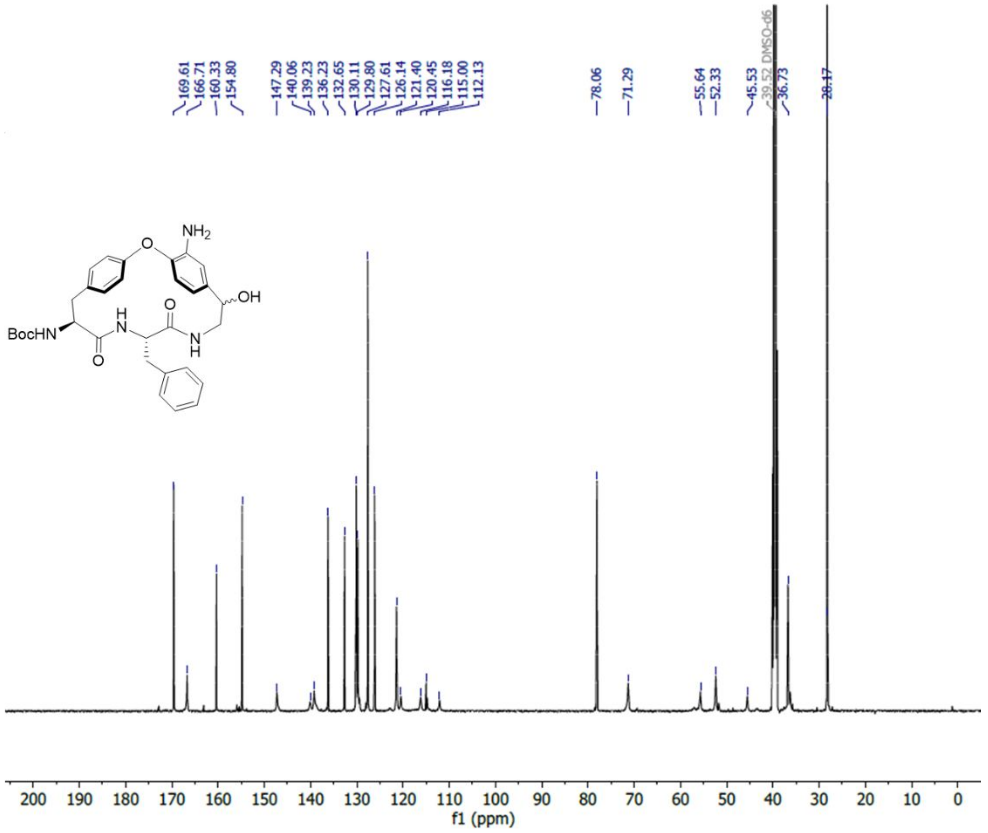

Compound 41: ^{1}H NMR ($CDCl_3$, 400 MHz)

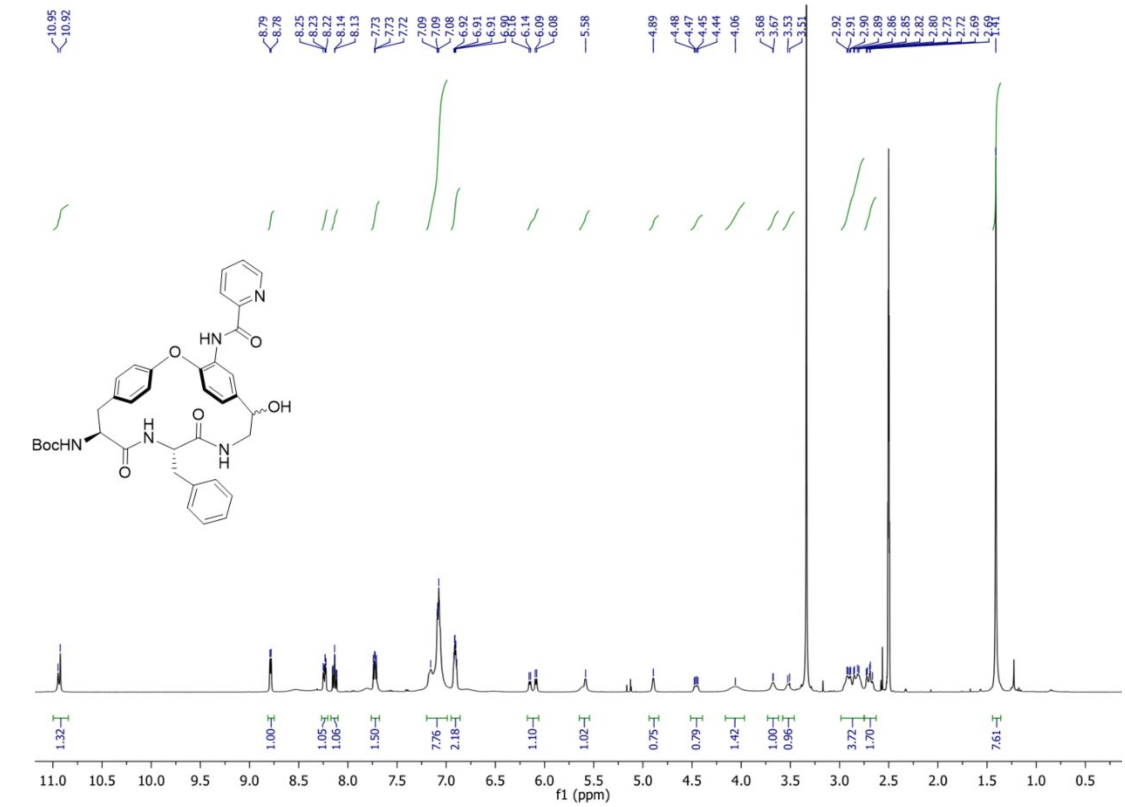

Compound 41: ^{13}C NMR ($CDCl_3$, 100 MHz)

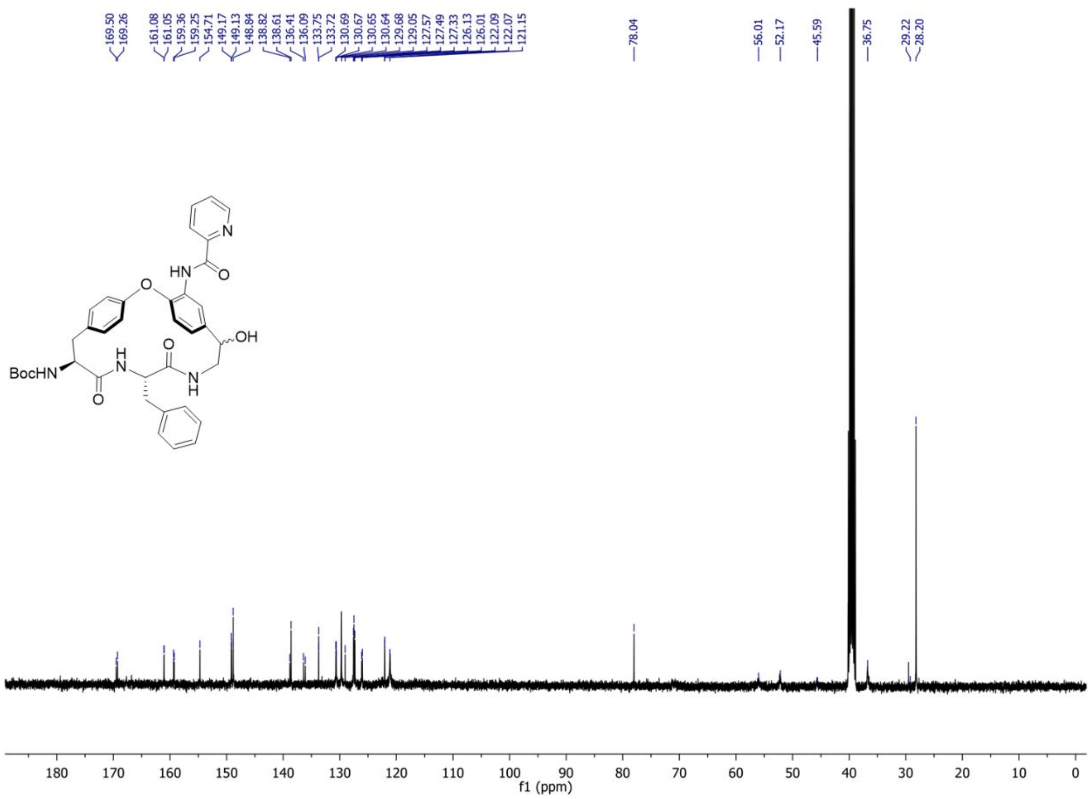

Compound 44: ^{1}H NMR (DMSO-d_6, 500 MHz)

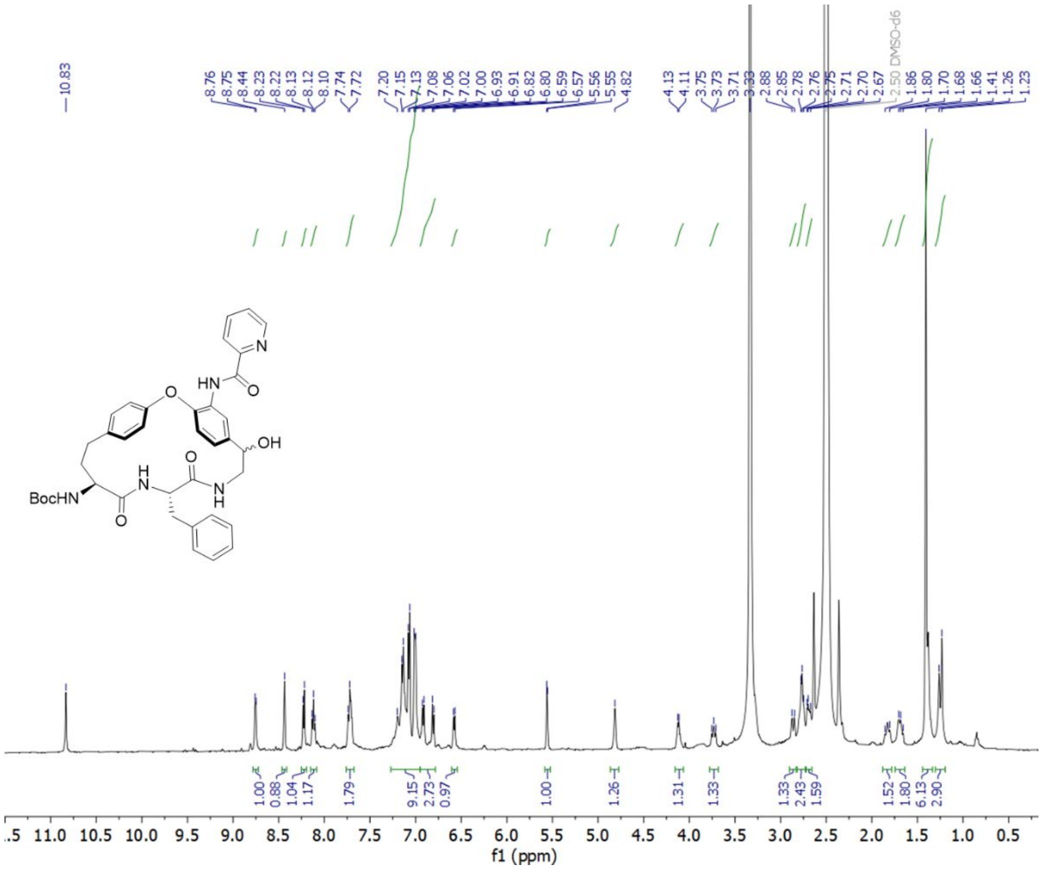

Compound 44: ^{13}C NMR (DMSO-d_6, 125 MHz)

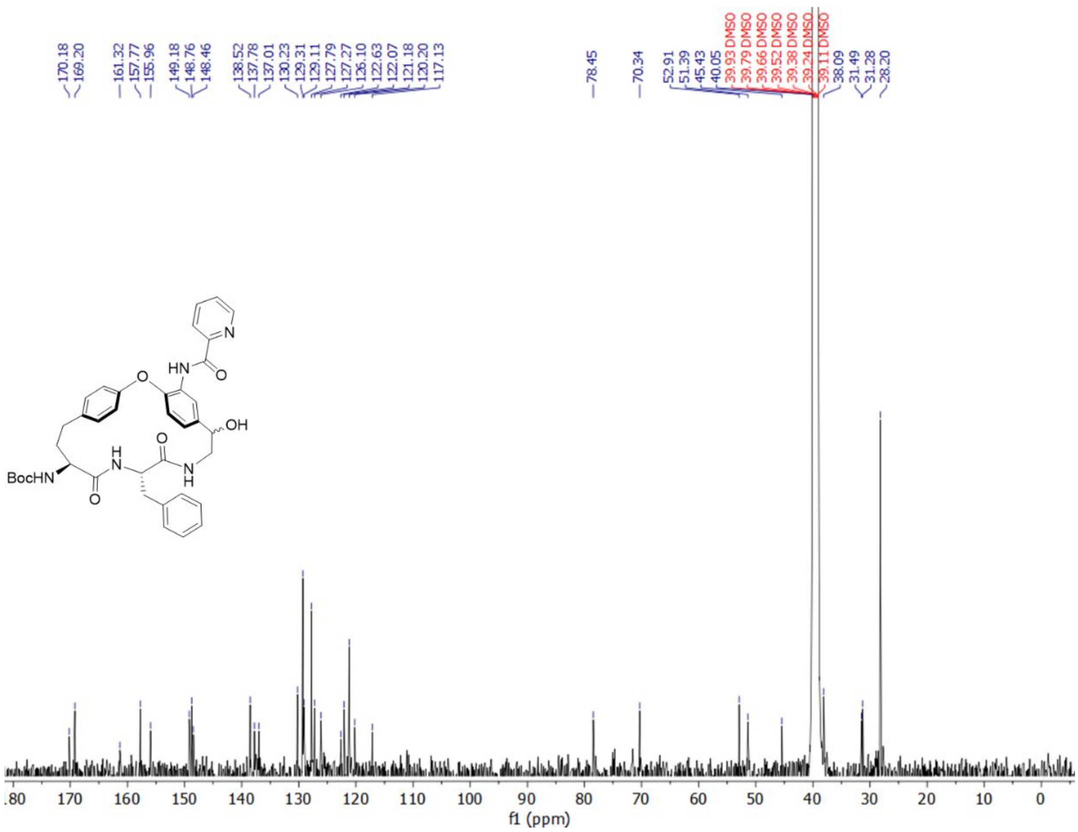

Compound 52: ^{1}H NMR (DMSO-d_6, 400 MHz)

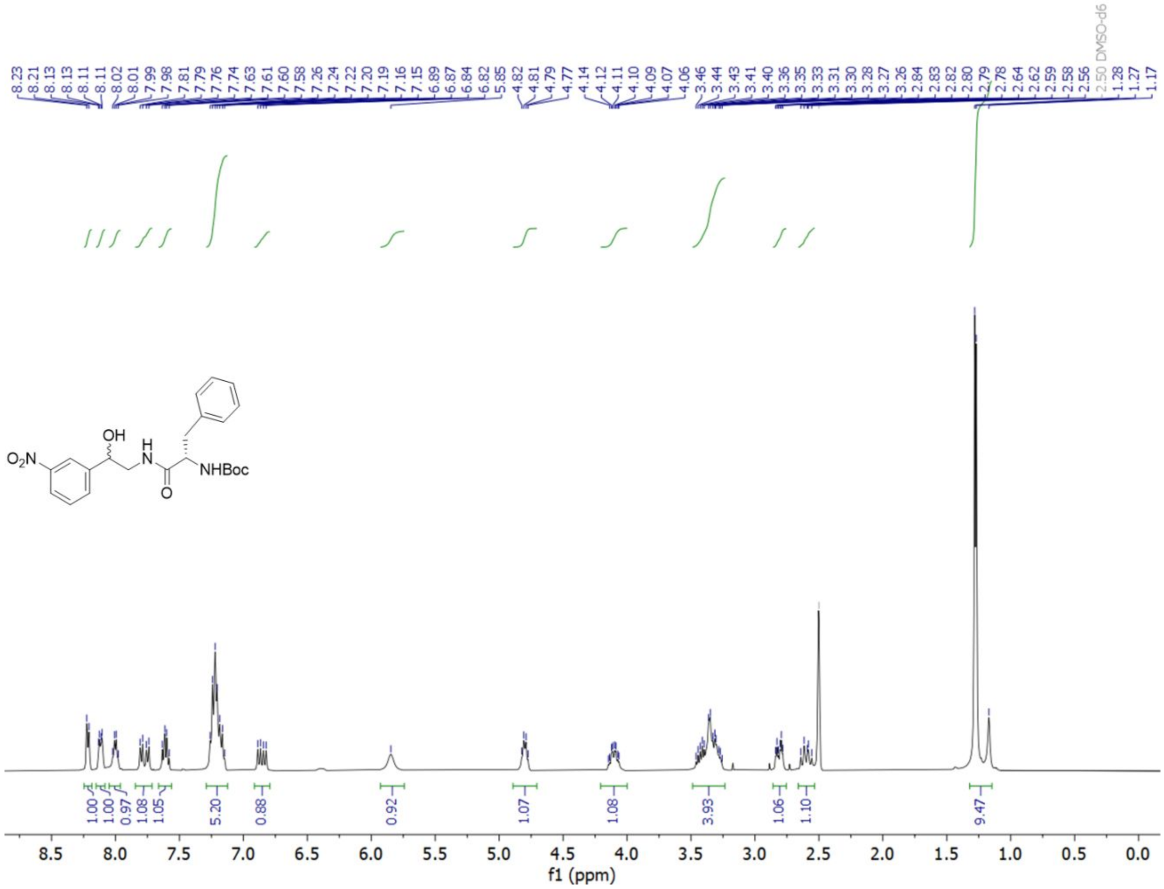

Compound 52: ^{13}C NMR (DMSO-d_6, 100 MHz)

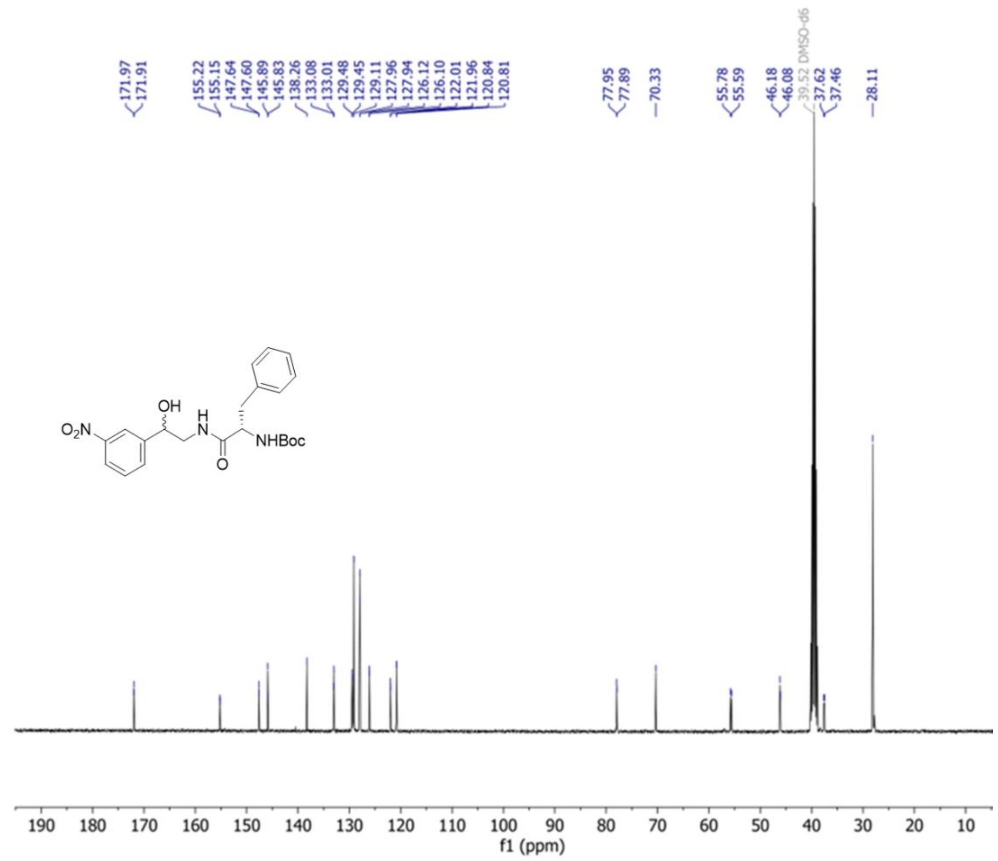

Compound 53: ^{1}H NMR (CD_3CN, 400 MHz)

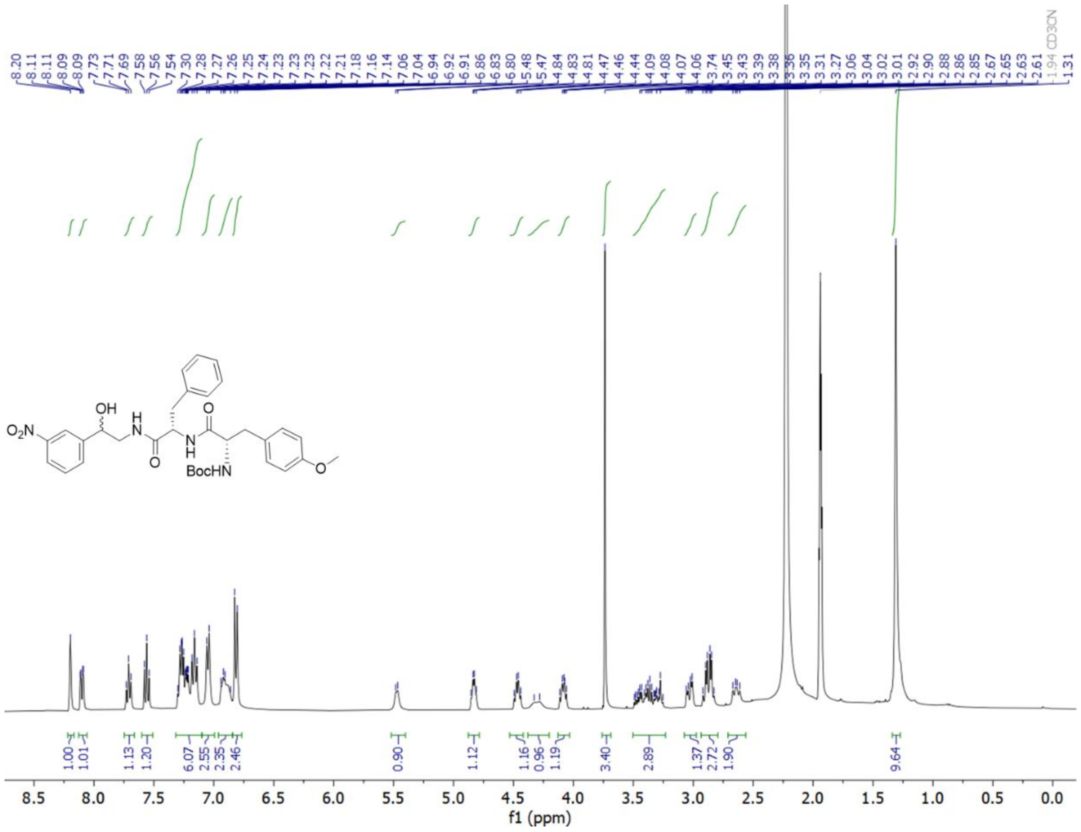

Compound 53: ^{13}C NMR (CD_3CN, 100 MHz)

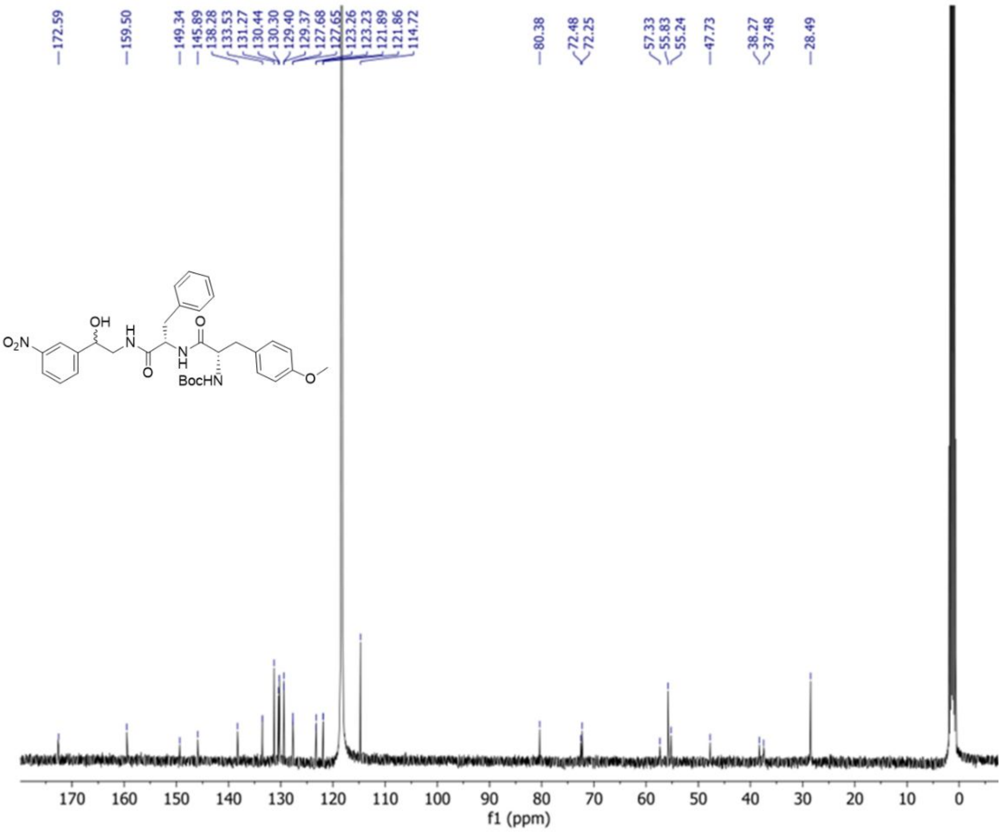

Compound 54: ^{1}H NMR (CD_3CN, 500 MHz)

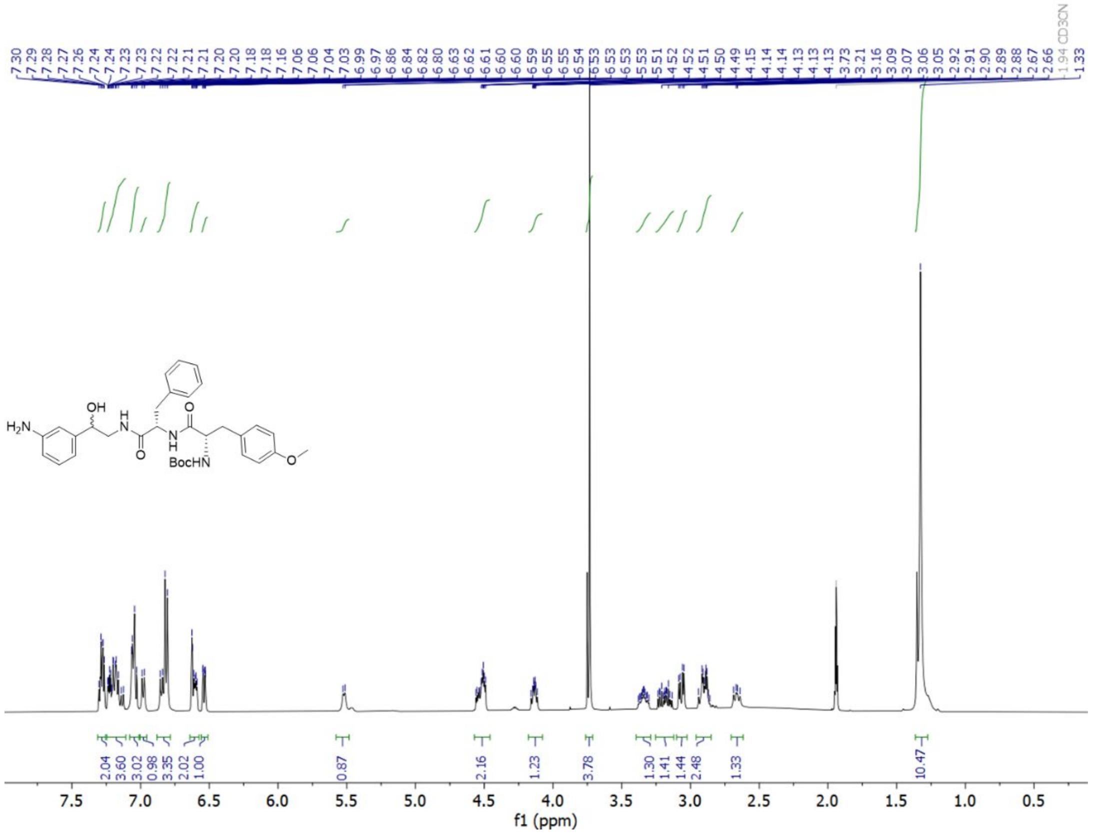

Compound 54: ^{13}C NMR (CD_3CN, 125 MHz)

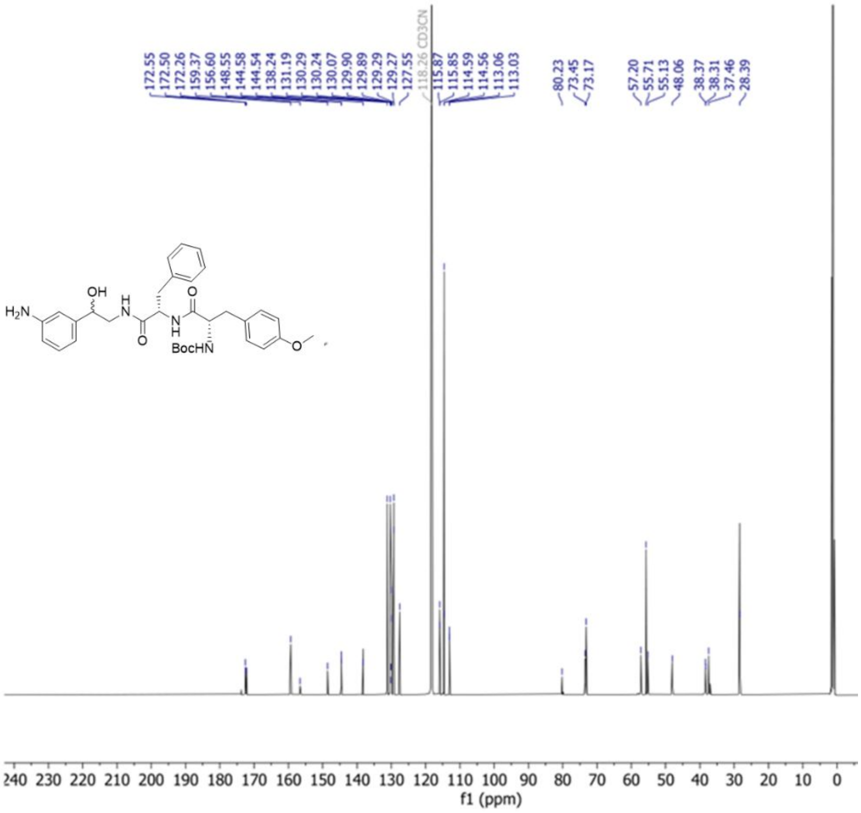

Compound 55: ^1H NMR (CD_3CN, 400 MHz)

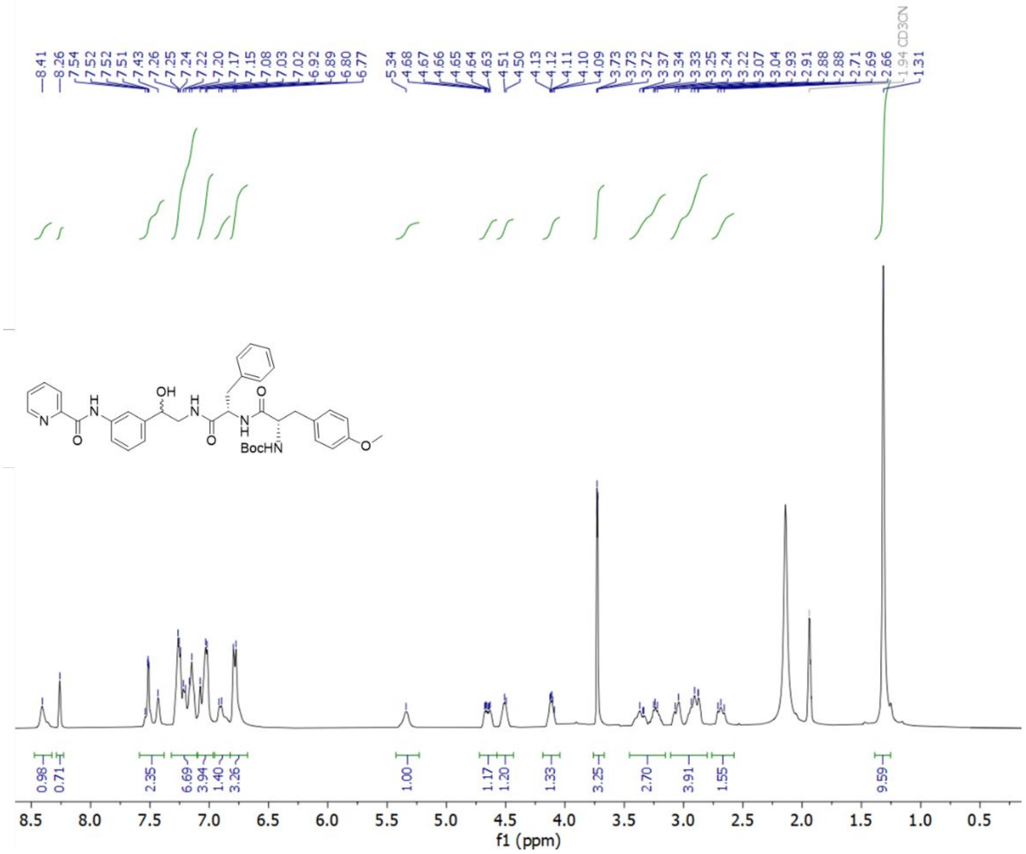

Compound 55: ^{13}C NMR (CD_3CN, 100 MHz)

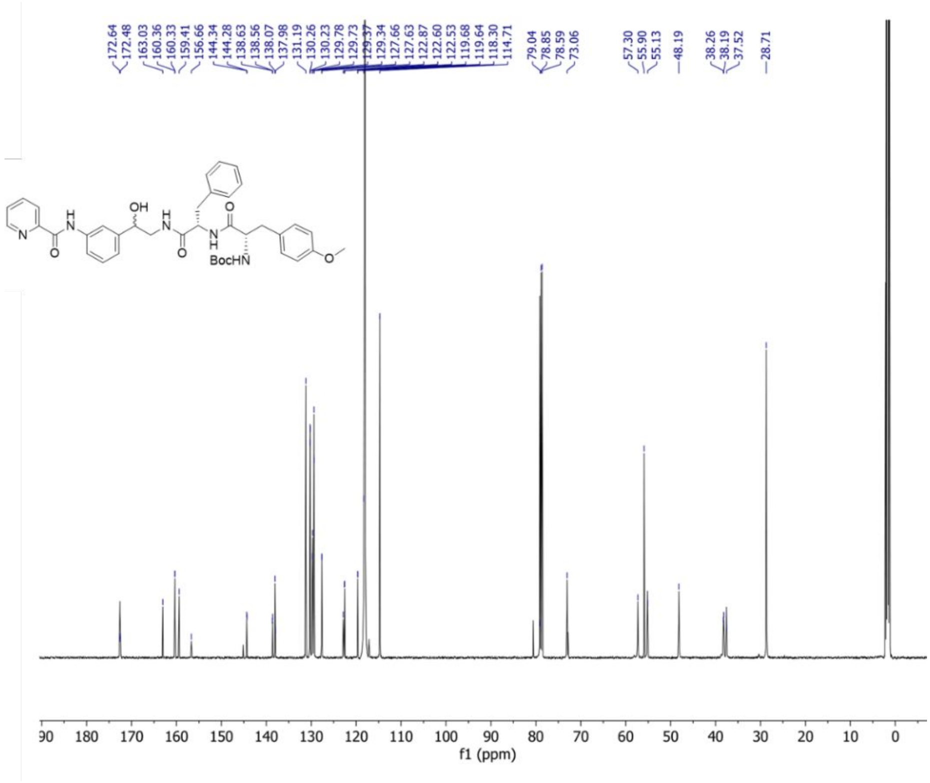

HPLC CHROMATOGRAMS AND MASS SPECTRA

LC-MS after purification by HPLC: Compound 1

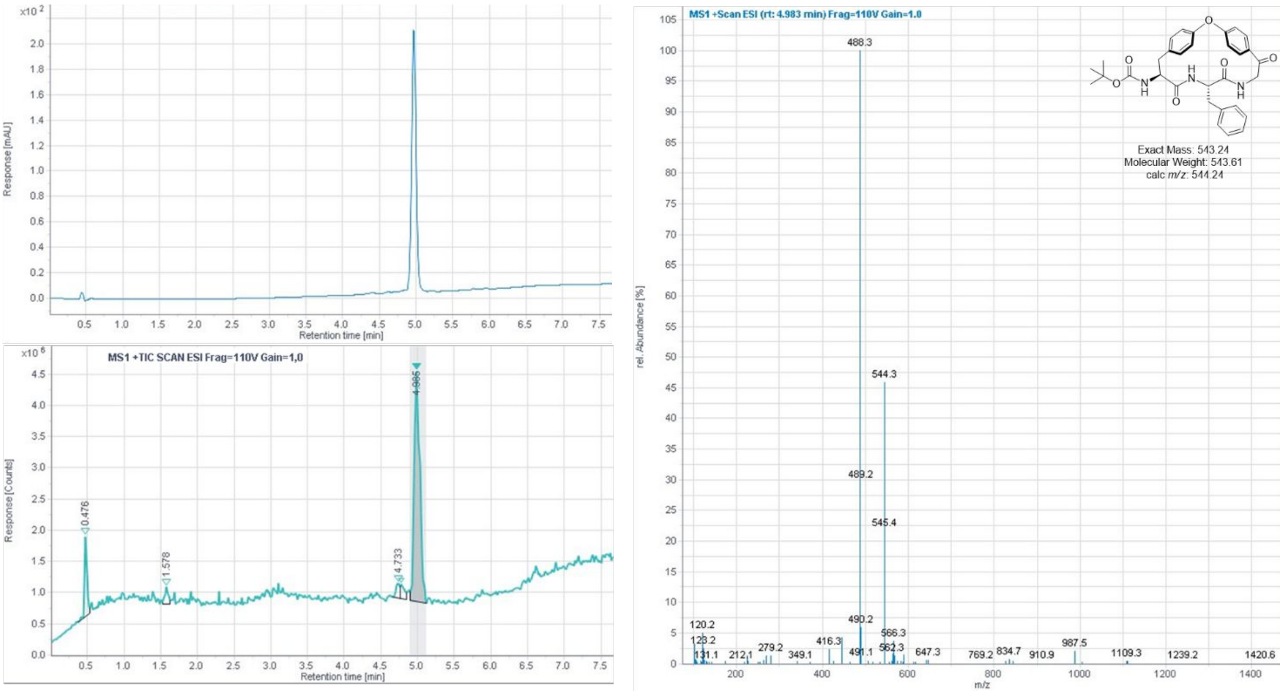

LC-MS after purification by HPLC: Compound 2

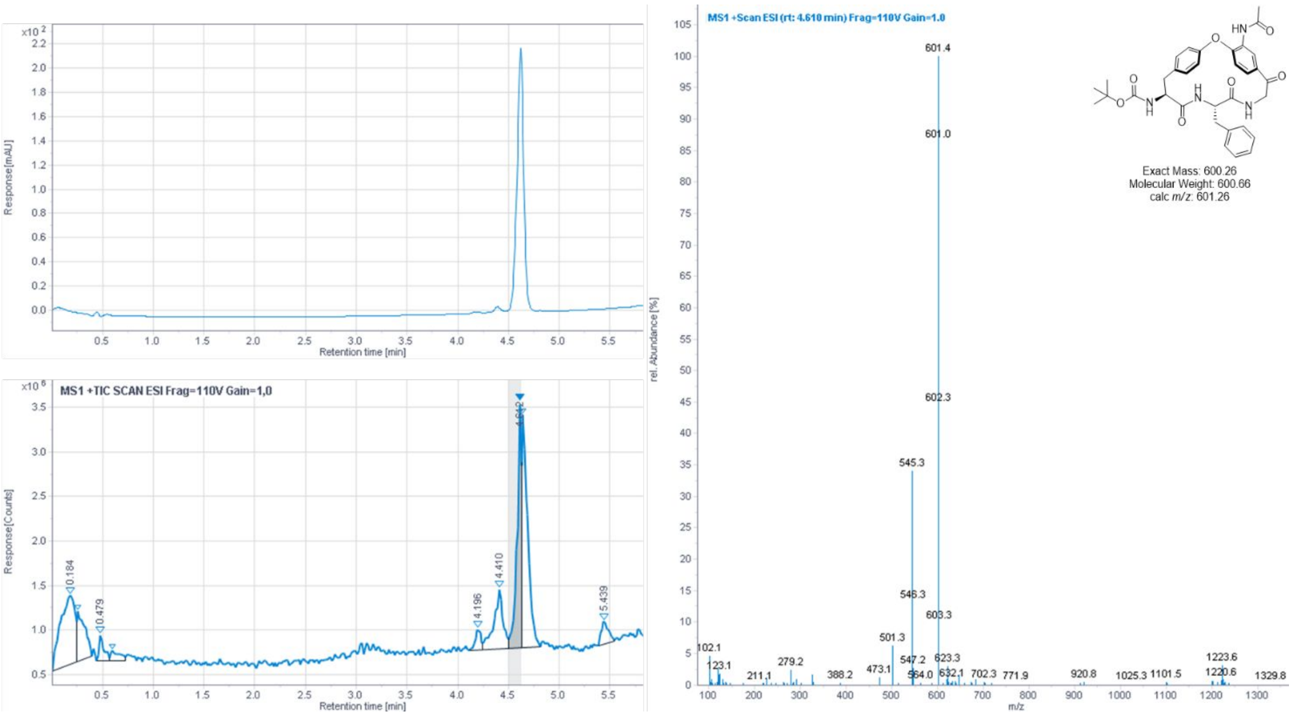

LC-MS after purification by HPLC purification: Compound 3

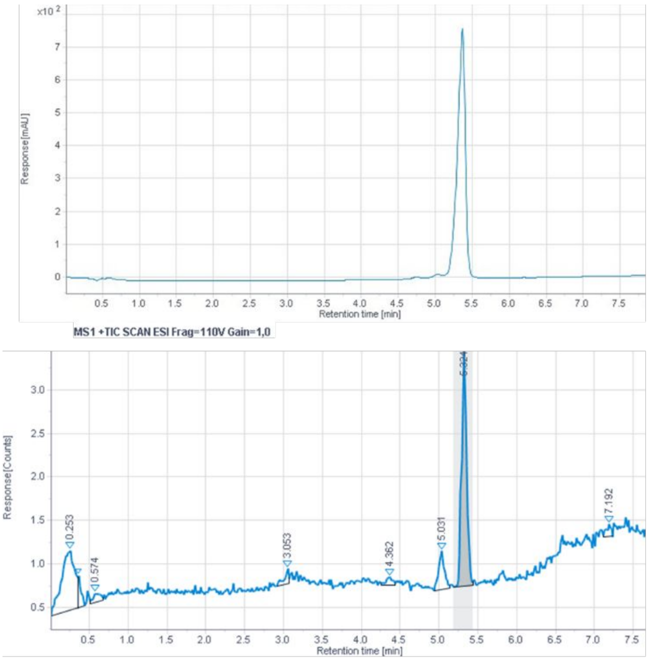

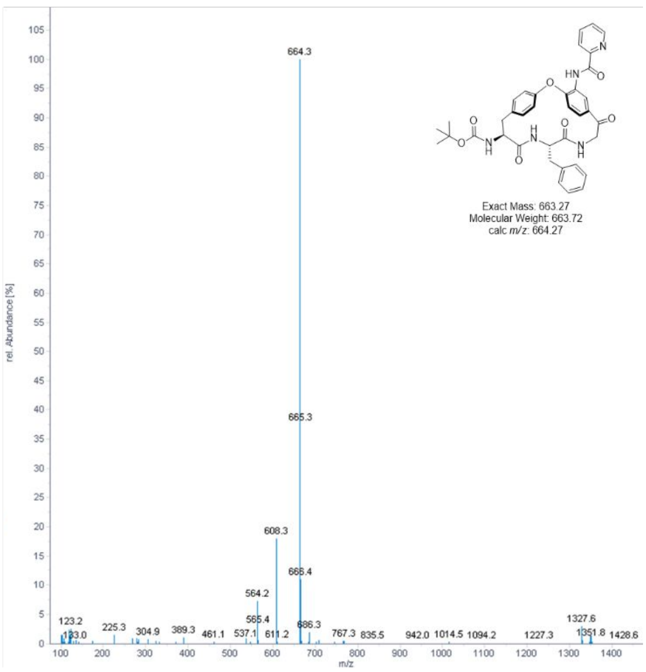

LC-MS after purification by HPLC: Compound 4

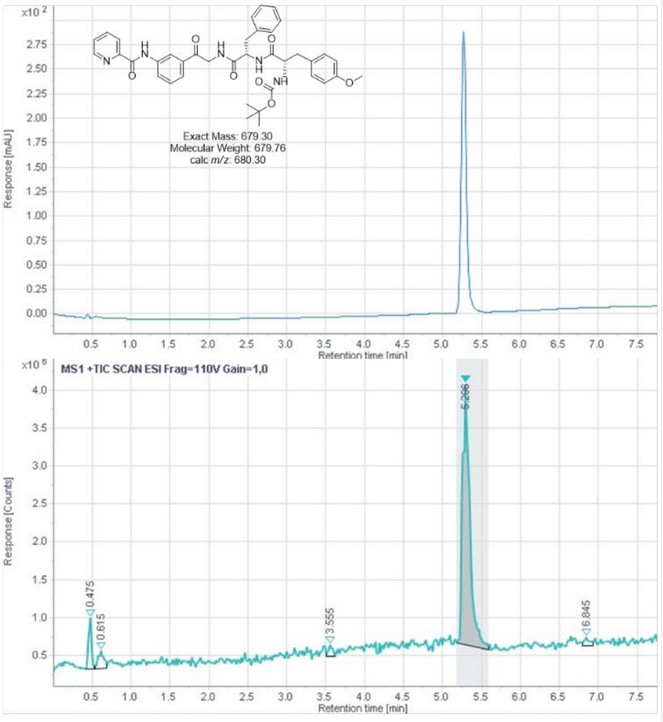

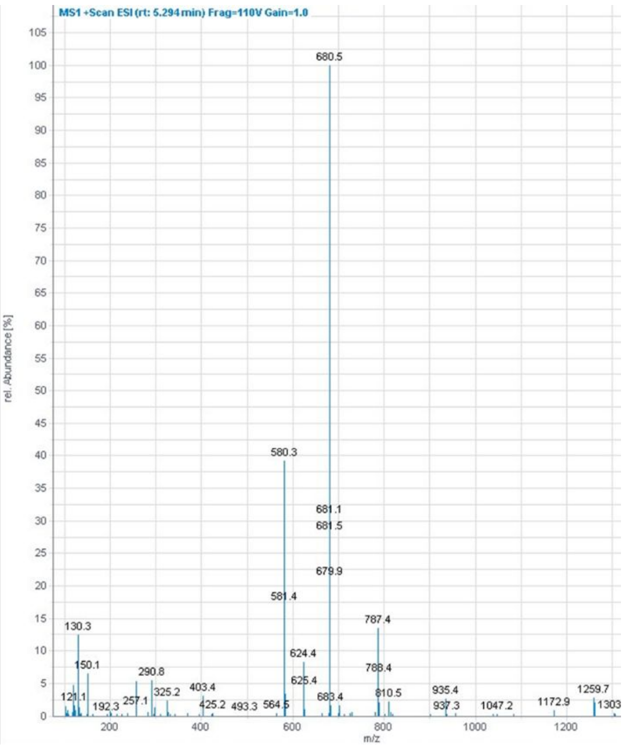

LC-MS after purification by HPLC: Compound 5

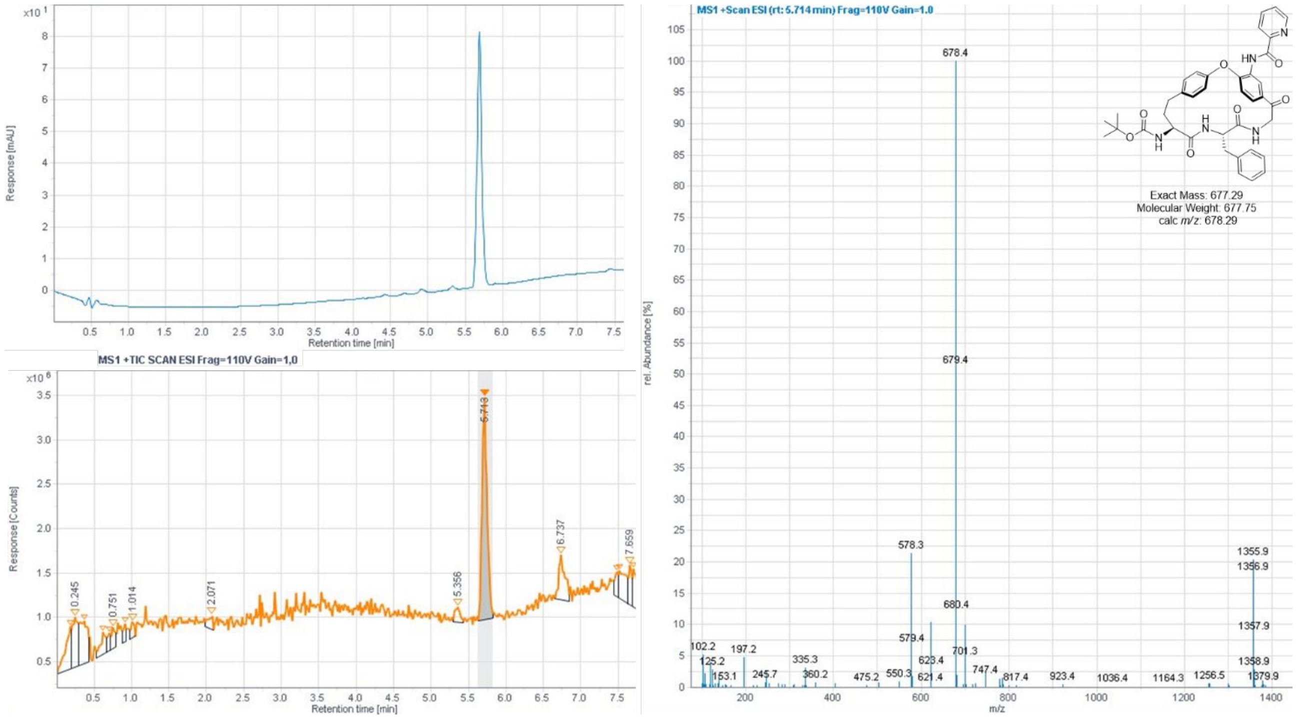

LC-MS after purification by HPLC: Compound 6

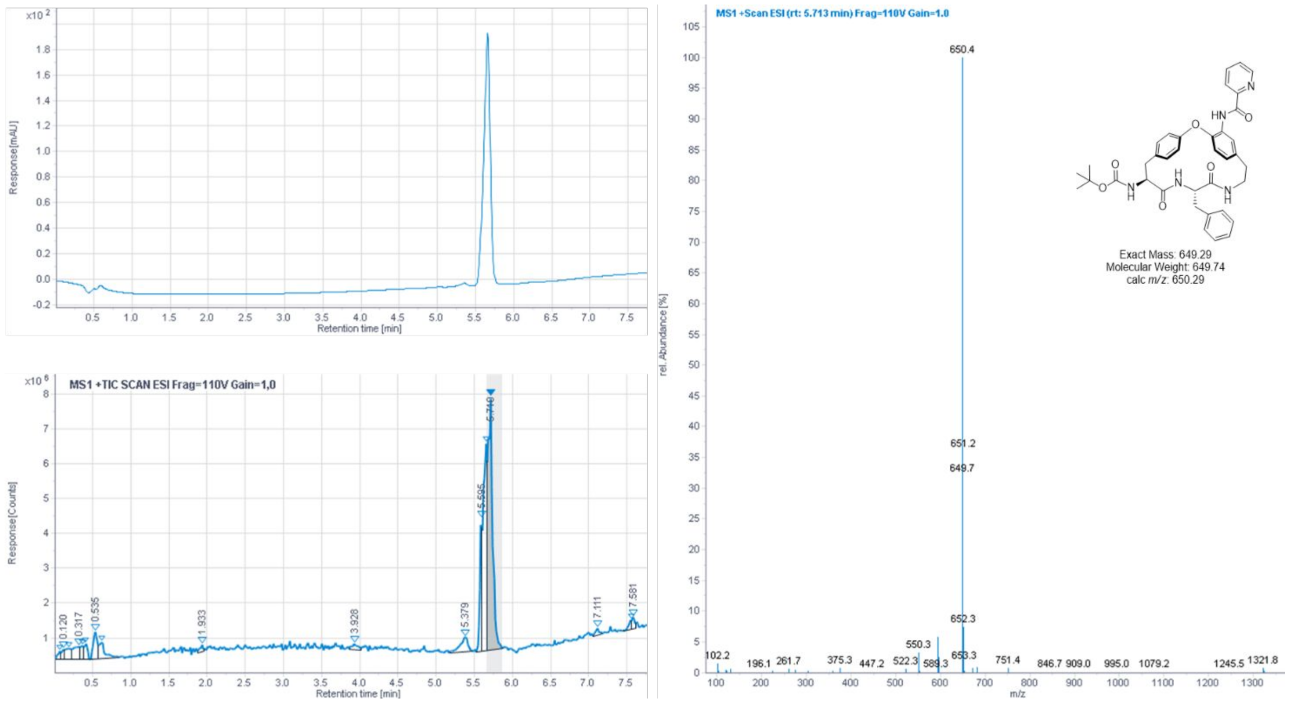

LC-MS after purification by HPLC: Compound 7

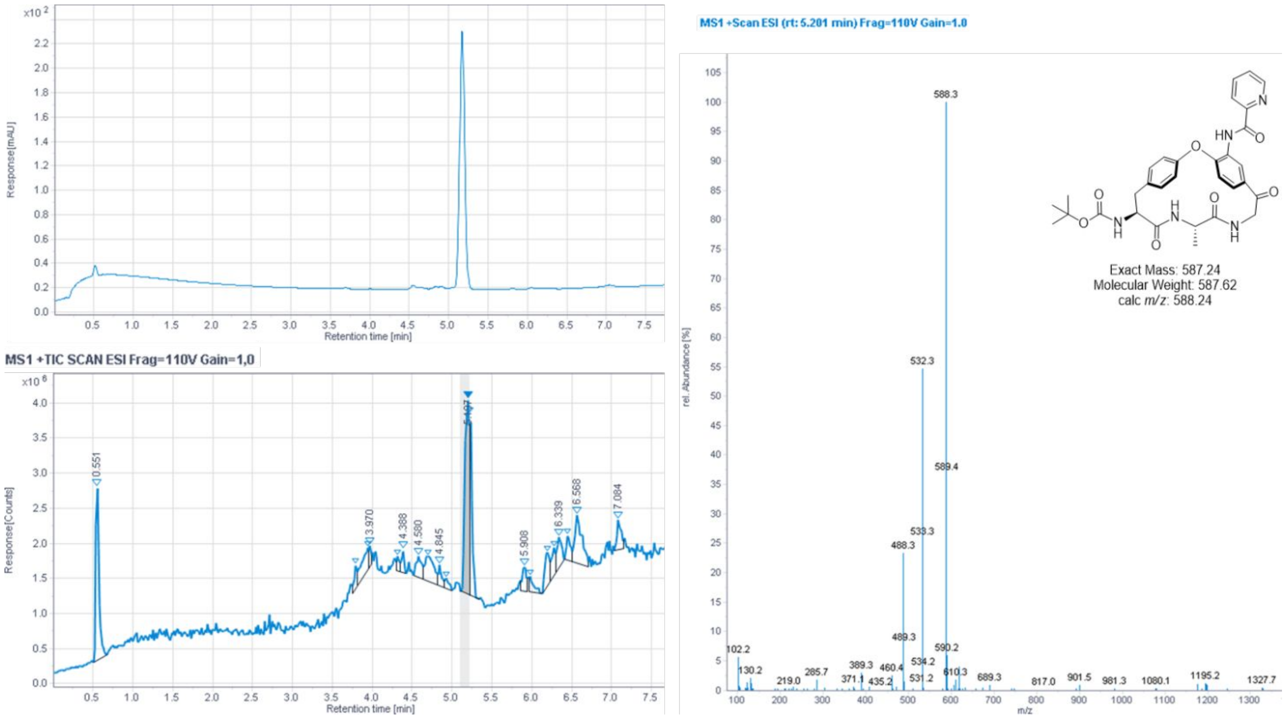

LC-MS after purification by HPLC: Compound 8

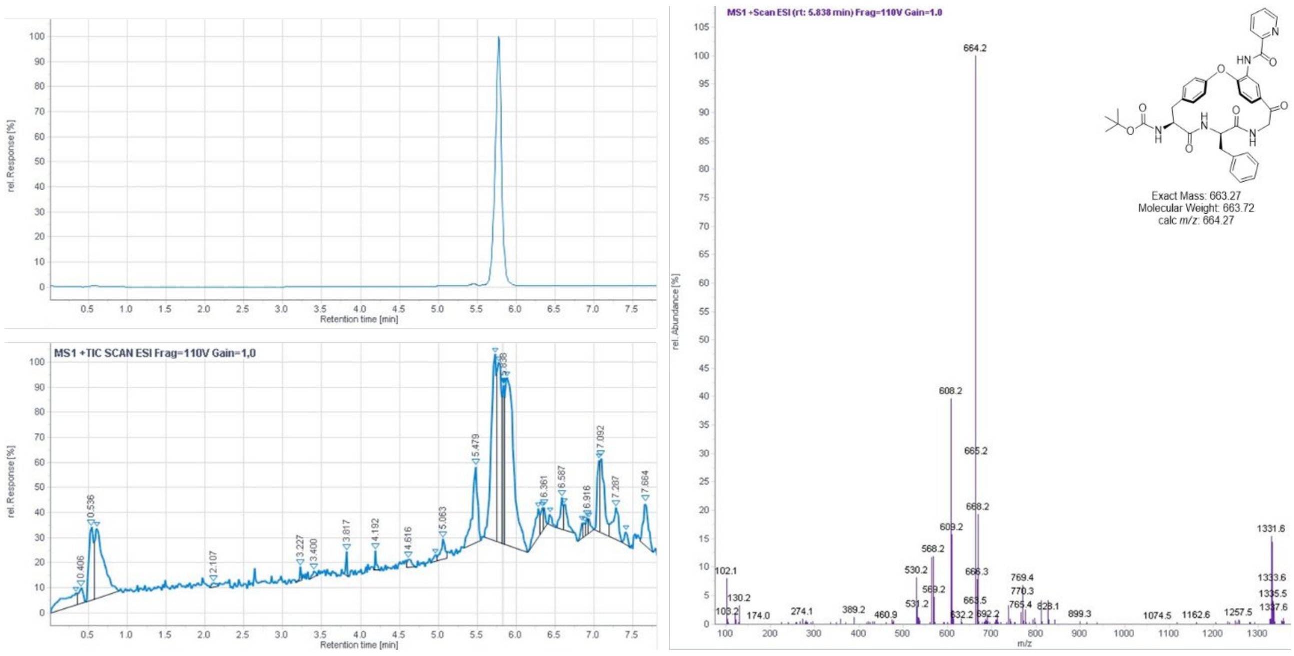

LC-MS after purification by HPLC: Compound 9

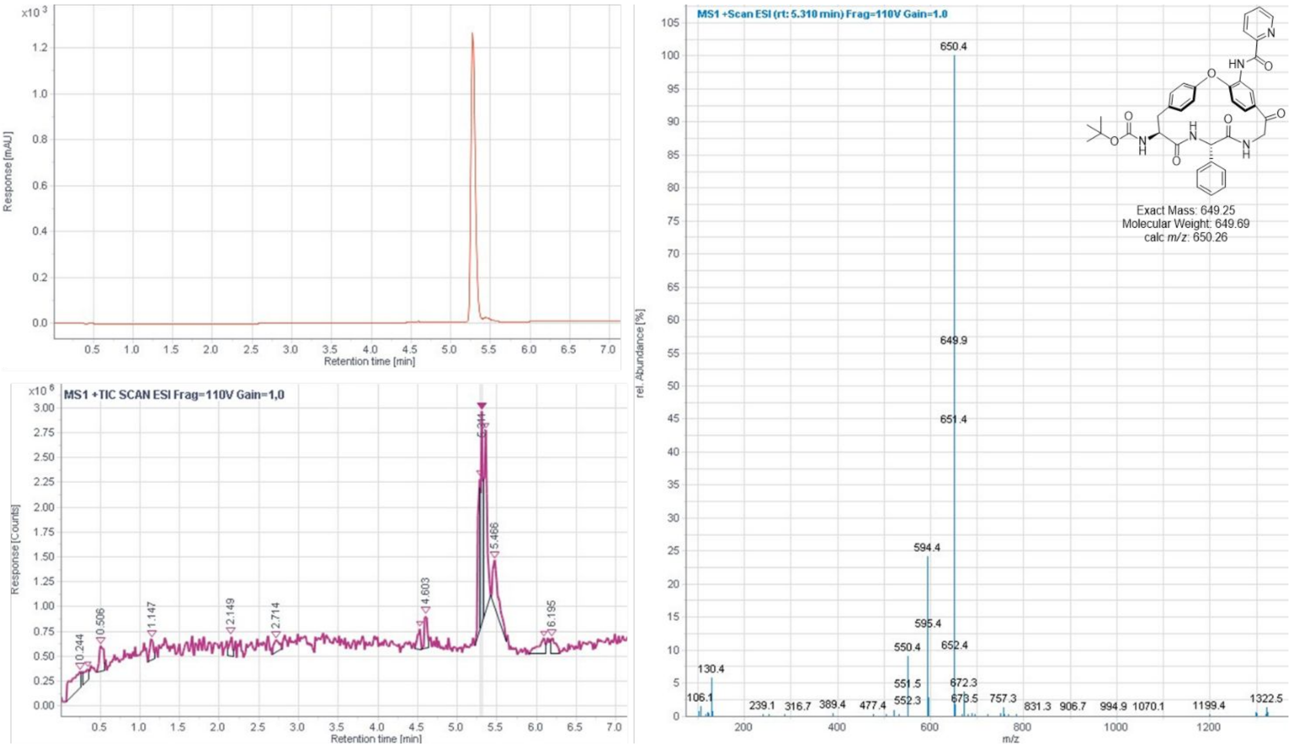

LC-MS after purification by HPLC: Compound 10

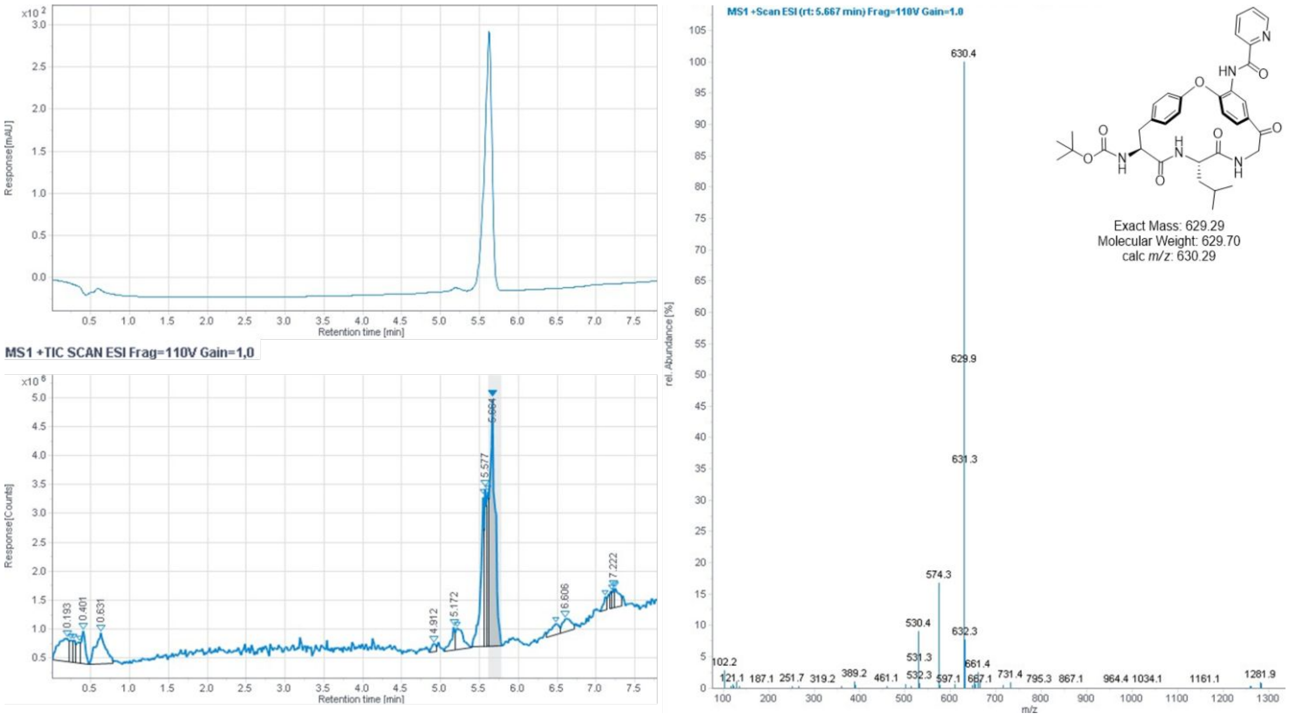

LC-MS after purification by HPLC: Compound 11

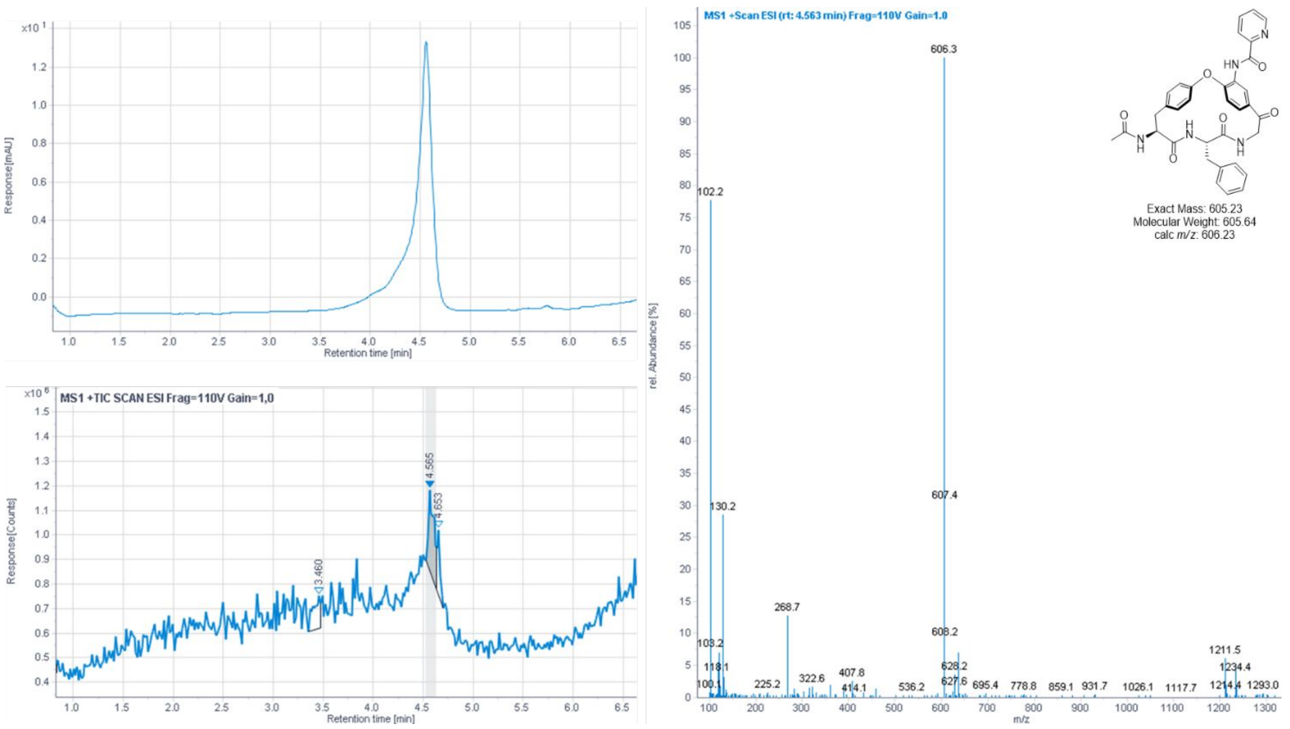

LC-MS after purification by HPLC: Compound 12

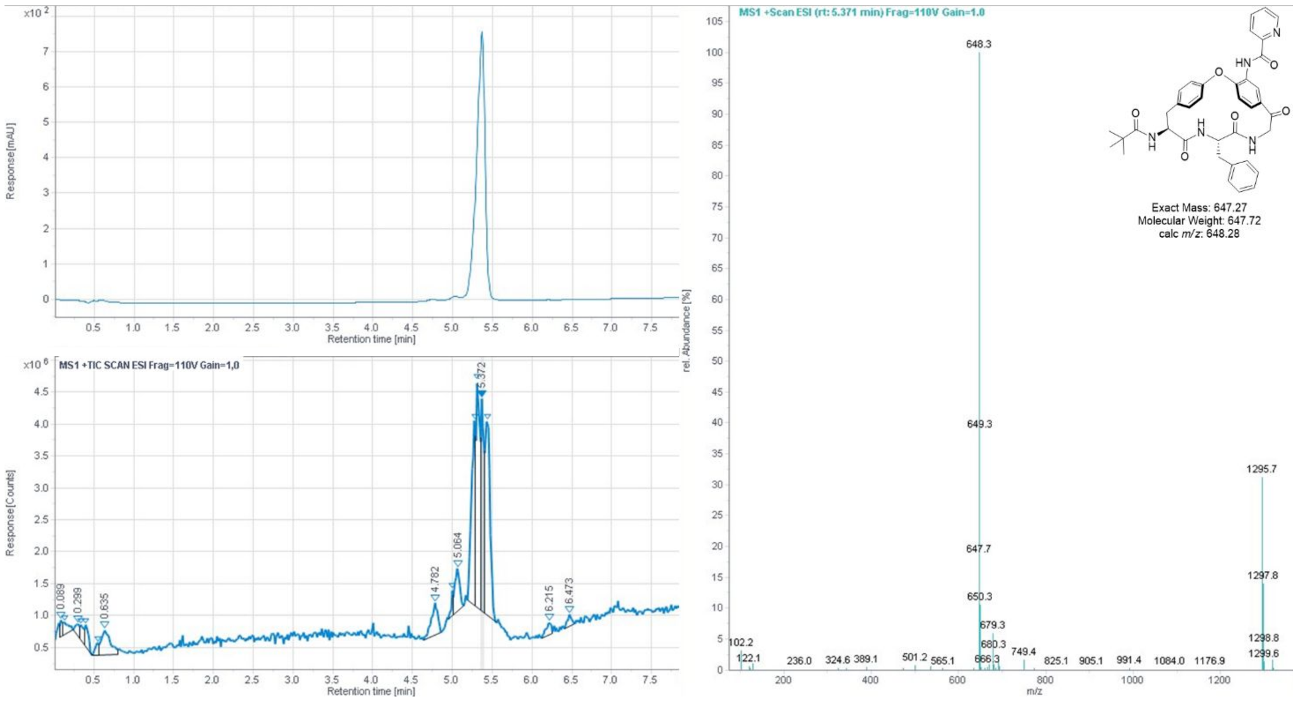

Supplement: Supplementary file 1 — jm4c01370_si_001.pdf [file jm4c01370_si_001.pdf]
